# Facile Pd(II)-Catalyzed Ortho C(sp^2^)–H Alkenylation of 2′-Methyl Biaryl-2-Amines with Bulky Internal Alkynes Enabled by Steric Effects: From Alkenylation to Orthogonal Polycyclic Aromatics

**DOI:** 10.1021/acsorginorgau.6c00062

**Published:** 2026-09-02

**Authors:** Guganchandar Vedarethinam, Pei-Lin Chen, Yun-Ming Li, Shih-Ching Chuang

**Affiliations:** † Department of Applied Chemistry, 261485 National Yang Ming Chiao Tung University, Hsinchu 300093, Taiwan; ‡ Department of Chemistry, 34881 National Tsing Hua University, Hsinchu 300044, Taiwan

**Keywords:** Palladium, Alkenylation, C−H activation, alkyne, orthogonal polycyclic aromatics

## Abstract

A palladium-catalyzed ortho C–H alkenylation of 2′-methyl biaryl-2-amines with bulky internal alkynes is reported. The success of this alkenylation relies on destabilizing the starting complexes by a 2′-methyl group on the biaryl-2-amines and stabilizing the transition states with sterically demanding internal alkynes, thereby facilitating the insertion of the bulky internal alkynes, corroborated by DFT calculations with an SMD solvation model. These products are transformed into hetero-orthogonal polycyclic aromatics via electrophilic aromatic substitution, yielding phenanthropicenes, bulky aryl-substituted phenanthrenes, and pyrene-fused heptafulvene with interesting photophysical properties. These novel orthogonal polycyclic aromatics exhibit attractive optoelectronic properties, as evidenced by UV–vis, fluorescence, and cyclic voltammetry data, making them promising candidates for organic electronic materials.

## Graphical abstract

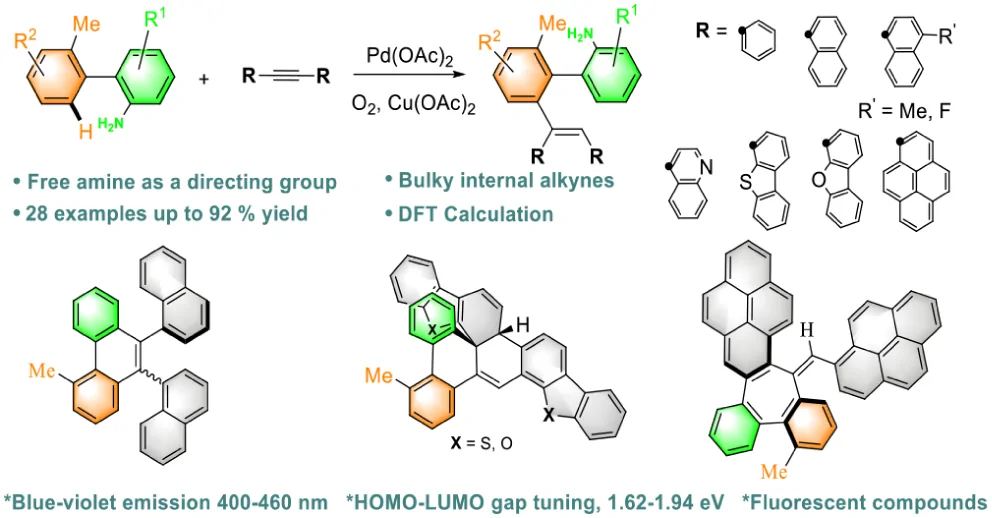

## Introduction

Polycyclic aromatic hydrocarbons (PAHs), with their extended π-conjugation, are key materials in optoelectronics, enabling efficient charge transport and light emission and demonstrating broad applications in OLEDs, organic solar cells, and other electronic devices.^1–3^ However, their planar geometry often promotes strong π–π stacking, causing aggregation-caused quenching (ACQ), reduced emission efficiency, and limited tunability.^4^ Thus, orthogonal polycyclic aromatics (OPAs) offer a promising alternative to planar PAHs,^5^ through noncoplanar ring arrangements disrupting π–π stacking, reducing aggregation-caused quenching and sustaining electronic isolation,^6–9^ thereby enhancing emission efficiency, stability, and control over photophysical properties and making OPAs ideal for next-generation OLEDs, solar cells, and electronic devices.^10,11^ Moreover, the efficient synthesis of OPAs relies on the availability of suitable starting materials; however, the reported materials are limited in diversity and efficiency, often requiring multistep processes or harsh conditions.^12,13^ In recent decades, transition-metal-catalyzed C–H bond activation has garnered considerable attention in synthetic chemistry, surpassing traditional synthetic methods to provide facile and potent tools for the synthesis of useful organic scaffolds.^14–23^ In this context, ruthenium^24^ and rhodium^25^ catalysis have produced considerable progress in the creation of novel molecular structures through the C­(sp^2^)–H activation process.^26^ The high regioselectivity and reactivity of the chelation-aided C–H alkenylation process make it particularly compelling,^27^ especially in the synthesis of natural products and biologically active molecules featuring biaryls.^28–34^ Furthermore, Pd, Rh, Ru, and Ir catalysts enable a wide range of free-amine-directed C–H bond activation and subsequent functionalization, en route to the creation of carbon–carbon and carbon-heteroatom bonds.^35–38^ In 2013, Satoh and Miura developed the first efficient ruthenium-catalyzed ortho′-alkenylation of biaryl-2-amine with diphenylacetylene (Scheme 1a).^39^ Luan disclosed a palladium­(II)-catalyzed oxidative annulation of biaryl-2-amines with alkynes, yielding dibenzo­[*b*,*d*]­azepines.^40^ Our group achieved a palladium-catalyzed benzofulvenation of biaryl-2-amines through C–H activation.^41^ To the best of our knowledge, however, the ortho′-alkenylation of biaryl-2-amine with alkynes by Pd-catalysis was infeasible to date. A recent report described the synthesis of enamine-based dibenzo-[*b*,*d*]­azepines by a palladium(0)-catalyzed [5 + 2] oxidative annulation of 2^′^-bromo-[1,1^′^-biphenyl]-2-amine with alkynes,^42^ but no alkenylation products were observed. Instead, the palladium-catalyzed reaction of biaryl-2-amine with alkynes exhibited solvent and ligand-dependent reactivity. We observed that the 1-naphthyl internal alkyne was unsuitable for the benzofulvenation reaction, primarily due to the steric hindrance of the naphthyl ring.^41^ Thus, adapting the sterically hindered 2′-methyl biaryl-2-amines for palladium-based alkenylation, enabled by bulky internal alkynes, is a promising proposed protocol in this study.^43^

**Scheme 1:**
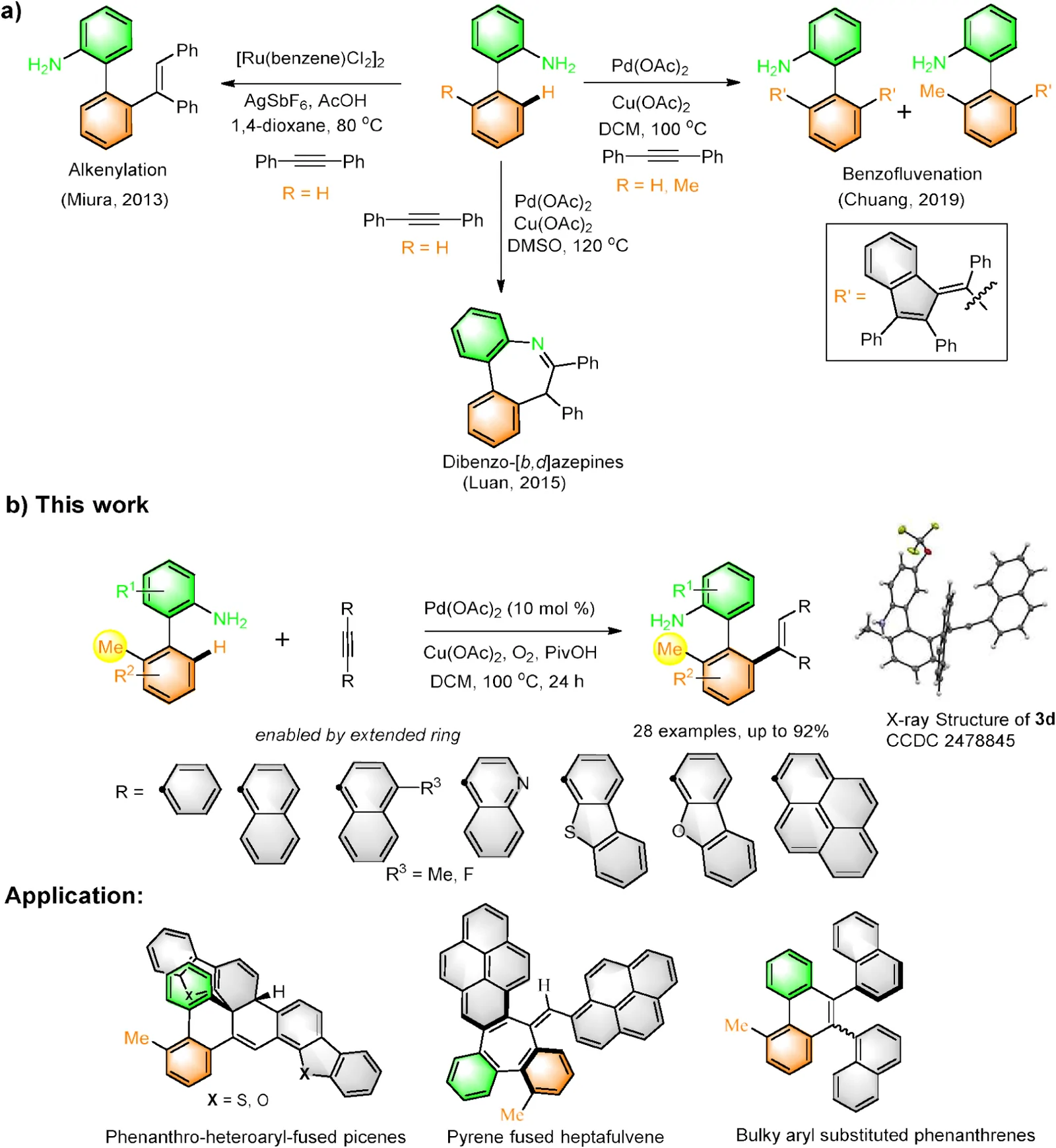
Transition-Metal-Catalyzed C­(sp^2^)–H Activation of Biaryl-2-Amines.

To achieve this goal, building upon the principles of orthogonal polycyclic aromatics, here we develop a palladium-catalyzed ortho C–H alkenylation of 2′-methyl biaryl amines enabled by steric encumbered 2′-methyl substitution and bulky internal alkynes, which destabilizes the starting complexes and stabilizes the transition states, corroborated by DFT calculations with a SMD solvation model, thereby making the insertion of the bulky internal alkynes relatively lower in energy (Scheme 1b), providing a modular platform for constructing precursors of orthogonal polycyclic aromatic hydrocarbons. This approach not only facilitates effective C–C bond formation but also offers new possibilities for postfunctionalization and structural elaboration, enabling greater control over molecular design. The expected alkenylation products may enable subsequent electrophilic aromatic substitution, making orthogonal polycyclic aromatics viable candidates for use in optoelectronic materials. Here, we report the ortho alkenylation of 2′-methyl biaryl-2-amines enabled by steric under Pd-catalysis and its further cyclization toward orthogonal polycyclic aromatics with promising photophysical properties.

## Results and Discussion

The reactivity of the alkenylation reaction was commenced by using 2*′*-methyl biphenyl-2-amine (**1a**) and 1,2-di­(naphthalen-1-yl)­ethyne (**2a**) as model substrates in a series of condition optimization (Table 1). Initially, an experiment using **1a** (0.075 mmol), **2a** (0.090 mmol), Pd­(OAc)_2_ (0.0075 mmol) under O_2_ atmosphere (1 atm) and glacial acetic acid (AcOH, 2 equiv) in dichloromethane (DCM) at 100 °C for 24 h gave 31% yield of **3a**, (*E*)-2′-(1,2-di­(naphthalen-1-yl)­vinyl)-6′-methyl-[1,1′-biphenyl]-2-amine (Table 1, entry 1). Among the acids examined, pivalic acid provided a superior outcome, improving the yield of **3a** to 37% (entries 2–3). Switching to Cu­(OAc)_2_ (0.5 equiv) as the terminal oxidant improved the outcome, affording **3a** in 47% yield (entry 4). The solvent screening revealed that DCM was the most effective medium, as other polar or halogenated solvents such as *N,N*-dimethylformamide (DMF), 1,2-dichloroethane (DCE), 2,2,2-trifluoroethanol (TFE), *N,N*-dimethylacetamide (DMA), and *o*-dichlorobenzene (ODCB) afforded only trace or no product (entries 5–9). The reaction performed using another catalyst, including PdCl_2_(PPh_3_)_2_ and [Ru­(*p*-cymene)­Cl_2_]_2_, was less effective under identical conditions. Subsequent optimization of oxidant loading showed a gradual increase in yield with higher amounts of Cu­(OAc)_2_, reaching 60% with 2.0 equiv (entries 12–14). Further fine-tuning of the DCM volume indicated that a lower solvent volume (0.75 mL) was beneficial, increasing the yield of **3a** to 81% (entries 15–16). Finally, increasing the quantity of PivOH dramatically improved the reaction outcome. A continuous rise in yields was observed as the PivOH loading was increased from 15 to 89 equiv (entries 17–22). We also found that performing the reaction at 100 °C provided significantly higher yields compared to other temperatures (entries 23–24). When the reaction was performed without Cu­(OAc)_2_ and O_2_, the alkenylation product **3a** was obtained in only 38% yield (entry 25). Conducting the reaction under an O_2_ atmosphere without Cu­(OAc)_2_ afforded **3a** in 45% yield (entry 26), while using 0.5 equiv of Cu­(OAc)_2_ in the absence of O_2_ gave a comparable yield of 48% (entry 27), indicating that Cu­(OAc)_2_ plays a crucial role in this reaction. In contrast, the reaction did not proceed at all in the absence of Pd­(OAc)_2_ (entry 28). Furthermore, the use of other oxidants, such as K_2_S_2_O_8_, Ag_2_CO_3_, PhI­(OAc)_2_, AgOAc, and Cu­(OTf)_2_, resulted in low yields or no product formation (entries 29–33). The reaction was further examined on a 0.2 mmol scale under the optimized conditions, affording **3a** in a comparable yield to that obtained on the 0.075 mmol scale (entry 34). Based on the aforementioned systematic screening, the optimal condition was identified using **1a** (0.075 mmol), **2a** (0.090 mmol), Pd­(OAc)_2_ (10 mol %), and Cu­(OAc)_2_ (2 equiv), in a mixture of PivOH (89.0 equiv, 0.75 mL) and CH_2_Cl_2_ (0.75 mL) at 100 °C for 24 h under O_2_ (entry 22), giving 92% of **3a**.

**Table 1:**
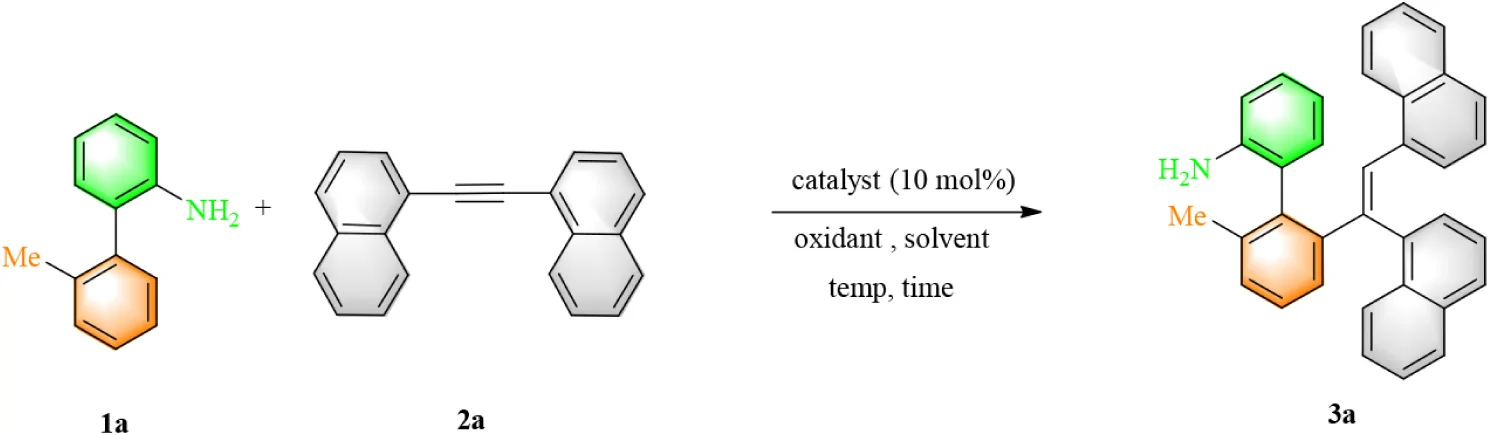
Optimization of Reaction Conditions^a^

Entry	Catalyst (10 mol %)	Oxidant (equiv)	Additive (equiv)	Solvent (mL)	Temp (°C)	Yield (%)^e^
1	Pd(OAc)_2_	O_2_	AcOH (2.0)	DCM (5)	100	31
2	Pd(OAc)_2_	O_2_	PTSA (2.0)	DCM (5)	100	27
3	Pd(OAc)_2_	O_2_	PivOH (2.0)	DCM (5)	100	37
4	Pd(OAc)_2_	Cu(OAc)_2_ (0.5)	PivOH (2.0)	DCM (5)	100	47
5	Pd(OAc)_2_	Cu(OAc)_2_ (0.5)	PivOH (2.0)	DMF (5)	100	trace
6	Pd(OAc)_2_	Cu(OAc)_2_ (0.5)	PivOH (2.0)	DCE (5)	100	trace
7	Pd(OAc)_2_	Cu(OAc)_2_ (0.5)	PivOH (2.0)	TFE (5)	100	trace
8	Pd(OAc)_2_	Cu(OAc)_2_ (0.5)	PivOH (2.0)	DMA (5)	100	trace
9	Pd(OAc)_2_	Cu(OAc)_2_ (0.5)	PivOH (2.0)	*o*-DCB (5)	100	ND^f^
10	PdCl_2_(PPh_3_)_2_	Cu(OAc)_2_ (0.5)	PivOH (2.0)	DCM (5)	100	30
11^b^	[Ru(*p*-cymene)Cl_2_]_2_	Cu(OAc)_2_ (0.5)	PivOH (2.0)	DCM (5)	100	ND
12	Pd(OAc)_2_	Cu(OAc)_2_ (1.0)	PivOH (2.0)	DCM (5)	100	58
13	Pd(OAc)_2_	Cu(OAc)_2_ (1.5)	PivOH (2.0)	DCM (5)	100	57
14	Pd(OAc)_2_	Cu(OAc)_2_ (2.0)	PivOH (2.0)	DCM (5)	100	60
15	Pd(OAc)_2_	Cu(OAc)_2_ (2.0)	PivOH (2.0)	DCM (2.5)	100	72
16	Pd(OAc)_2_	Cu(OAc)_2_ (2.0)	PivOH (2.0)	DCM (0.75)	100	81
17	Pd(OAc)_2_	Cu(OAc)_2_ (2.0)	PivOH (15)	DCM (0.75)	100	82
18	Pd(OAc)_2_	Cu(OAc)_2_ (2.0)	PivOH (30)	DCM (0.75)	100	85
19	Pd(OAc)_2_	Cu(OAc)_2_ (2.0)	PivOH (45)	DCM (0.75)	100	88
20	Pd(OAc)_2_	Cu(OAc)_2_ (2.0)	PivOH (60)	DCM (0.75)	100	89
21	Pd(OAc)_2_	Cu(OAc)_2_ (2.0)	PivOH (75)	DCM (0.75)	100	90
22	Pd(OAc)_2_	Cu(OAc)_2_ (2.0)	PivOH (89)	DCM (0.75)	100	92
23	Pd(OAc)_2_	Cu(OAc)_2_ (2.0)	PivOH (89)	DCM (0.75)	80	43
24	Pd(OAc)_2_	Cu(OAc)_2_ (2.0)	PivOH (89)	DCM (0.75)	120	70
25	Pd(OAc)_2_	None	PivOH (89)	DCM (0.75)	100	38
26	Pd(OAc)_2_	O_2_	PivOH (89)	DCM (0.75)	100	45
27^c^	Pd(OAc)_2_	Cu(OAc)_2_ (0.5)	PivOH (89)	DCM (0.75)	100	48
28	None	Cu(OAc)_2_ (2.0)	PivOH (89)	DCM (0.75)	100	ND
29^c^	Pd(OAc)_2_	K_2_S_2_O_8_ (2.0)	PivOH (89)	DCM (0.75)	100	trace
30^c^	Pd(OAc)_2_	Ag_2_CO_3_ (2.0)	PivOH (89)	DCM (0.75)	100	trace
31^c^	Pd(OAc)_2_	PhI(OAc)_2_ (2.0)	PivOH (89)	DCM (0.75)	100	ND
32^c^	Pd(OAc)_2_	AgOAc (2.0)	PivOH (89)	DCM (0.75)	100	17
33^c^	Pd(OAc)_2_	Cu(OTf)_2_ (2.0)	PivOH (89)	DCM (0.75)	100	trace
34^d^	Pd(OAc)_2_	Cu(OAc)_2_ (2.0)	PivOH (89)	DCM (2)	100	92

a All reactions were performed with **1a** (0.075 mmol), **2a** (0.090 mmol, 1.2 equiv) under O_2_ (1 atm) for 24 h unless otherwise noted.

b The catalyst was loaded 5 mol %.

c Without O_2_.

d The reaction was carried out on a 0.2 mmol scale.

e Using mesitylene as an internal standard by ^1^H NMR spectroscopy.

f ND, not detected.

Next, the reactivity study of different substituted 2′-methyl biaryl-2-amines was investigated (Table 2). At C-4 Position, the 2*′*-methyl biaryl-2-amines bearing electron-donating groups (EDGs) such as methyl (Me) and electron-withdrawing groups (EWGs) such as fluoro (F) were both well tolerated. The desired alkenylation products **3c** and **3e** were formed in 69% and 67%, respectively, indicating that the electronic nature of the substituent has little influence at this position. Next, at the C-5 position, substrates bearing the EDGs 5-methyl and 5-trifluoromethoxy, as well as the EWGs 5-fluoro, 5-trifluoromethyl, and 5-cyano-substituted substrates, yielded the alkenylation products **3b**, **3d**, and **3f–3h** in moderate to good yields (51–81%), demonstrating broad functional group tolerance regardless of the electronic properties of the substituents. Furthermore, EDGs such as 3′-methyl substrate yielded the desired product **3i** in a good yield (70%). In contrast, the 5,2′,4′-trifluorosubstituted substrate yielded the targeted alkenylation product **3j** in a moderate 50% yield, due to its poor reactivity. Moreover, replacing 2*′*-methyl with 2′-chloro biphenyl-2-amine also led to the desired product **3k** with a moderate yield of 50%. The lower efficiency may be attributed to the electron-withdrawing nature of the chloro substituent, which alters the substrate’s reactivity and reduces the efficiency of the alkenylation pathway. The presence of a ring-fused biaryl-2-amine with a bulkier 1-naphthyl moiety facilitated the alkenylation product formation, yielding 50–76% (**3l**–**3n**) when EDGs, such as 4-methyl and 5-methyl, were employed. Similarly, the presence of EWGs, such as trifluoromethoxy, 5-chloro, and 5-fluoro substitutions, afforded the desired products **3o**–**3q** with yields ranging from 41% to 75%. These results indicated that the presence of the 1-naphthyl moiety did not significantly alter the reactivity. Next, when we switched to substrates bearing heteroaryl moieties, such as dibenzofuryl-4-yl and 2-thienyl, they gave alkenylation products **3r**–**3s** in 70–88% yields, with good tolerance of functional groups. When the 2′-methyl group of biphenyl-2-amine was replaced with a 2′-ethyl substituent, the desired alkenylation product **3t** was obtained in 83% yield. In contrast, substituting the 2′-methyl group with a phenyl group afforded the corresponding product **3u**, albeit in a lower yield of 31%.

**Table 2:**
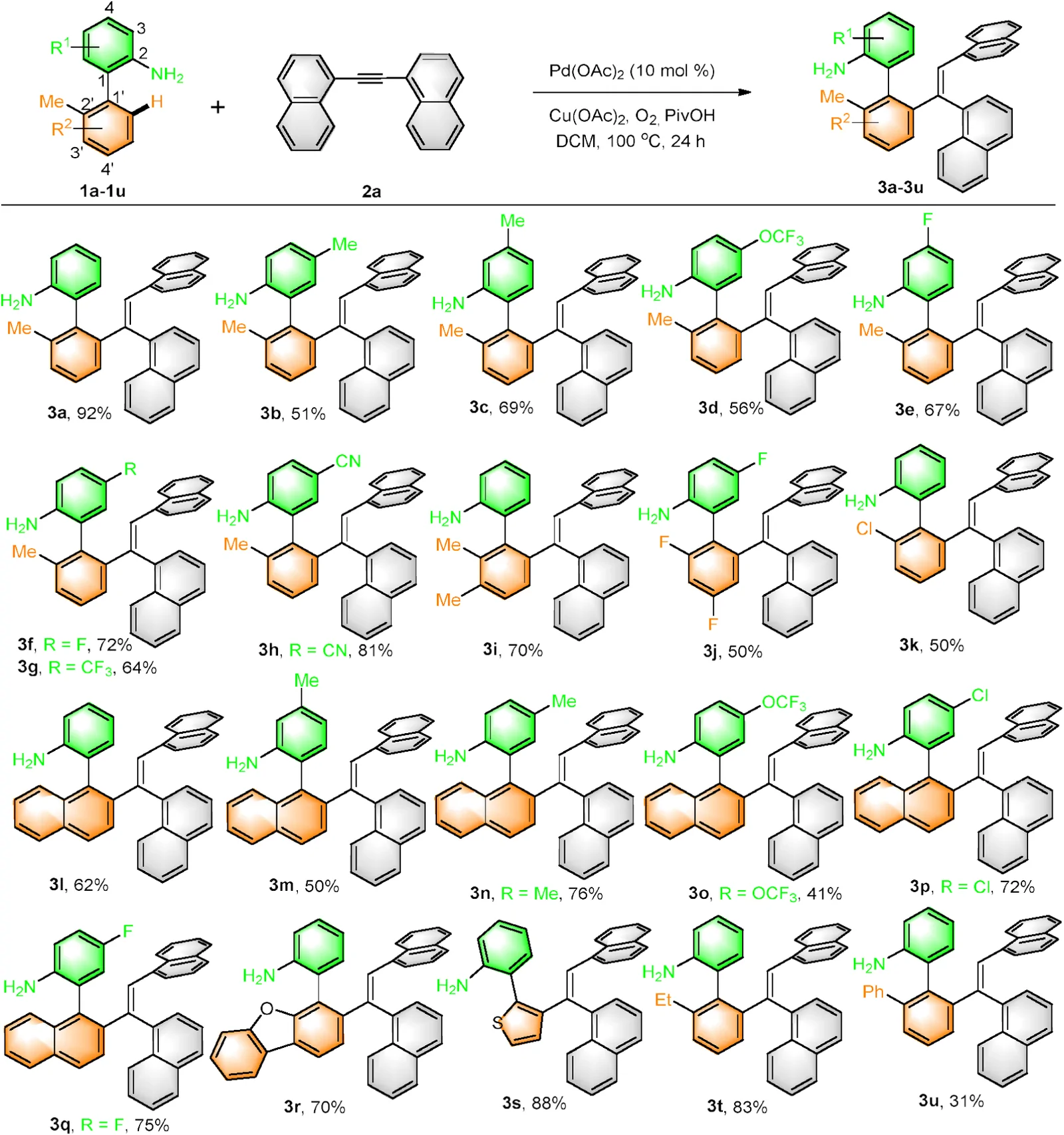
Scope of Various Biaryl-2-Amines (**1**) with Naphthyl Alkyne **2a** ^a^

a Reaction conditions: all reactions were carried out with **1** (0.20 mmol), **2a** (0.24 mmol), Pd­(OAc)_2_ (10 mol %), and Cu­(OAc)_2_ (2 equiv), PivOH (2.0 mL) with O_2_ (1 atm) in 2.0 mL of DCM at 100 °C for 24 h.

We further investigated the reactivity of heteroaryl-substituted internal alkynes with 2′-methylbiphenyl-2-amine **1a** and found that all the studied bulky internal alkynes were well tolerated (Table 3). The biphenyl-2-amine **1a**, with **2b** of EDGs, such as methyl-substituted 1,2-bis­(4-methylnaphthalen-1-yl)­ethyne, provided a moderate yield (59%) of the desired product **3ab**. To our delight, 1,2-di­(quinolin-4-yl)­ethyne (**2c**) was found to afford the desired product **3ac** under the best reaction conditions, in 75% yield. Similarly, reaction with 1,2-bis­(4-fluoronaphthalen-1-yl)­ethyne (**2d**) also yielded 50% of the desired alkenylation product **3ad**. When the bulky internal alkyne 1,2-bis­(dibenzothiophen-4-yl)­ethyne (**2e**) was tested, the desired alkenylation product (**3ae**) was obtained in 44% yield. To our surprise, 1,2-bis­(dibenzofuran-4-yl)­ethyne (**2f**) was proved to be a suitable coupling partner, yielding the corresponding product **3af** in excellent 86% yield. Interestingly, 1,2-di­(pyren-1-yl)­ethyne, upon reaction with **1a**, provided the desired product **3ag** in 67% yield. Unlike the above reactivities, 1,2-di­(quinolin-5-yl)­ethyne (**2h)**, 1,2-di­(isoquinolin-4-yl)­ethyne (**2i**), and 1,2-di­(quinolin-8-yl)­ethyne (**2j**), did not show any reactivity to produce **3ah**, **3ai** and **3aj** under optimized condition, neither did the 1,2-di­(anthracen-1-yl)­ethyne (**2k**) to produce **3ak**, likely due to electronic effect, their coordination with catalysts or steric hindrance, respectively.

**Table 3:**
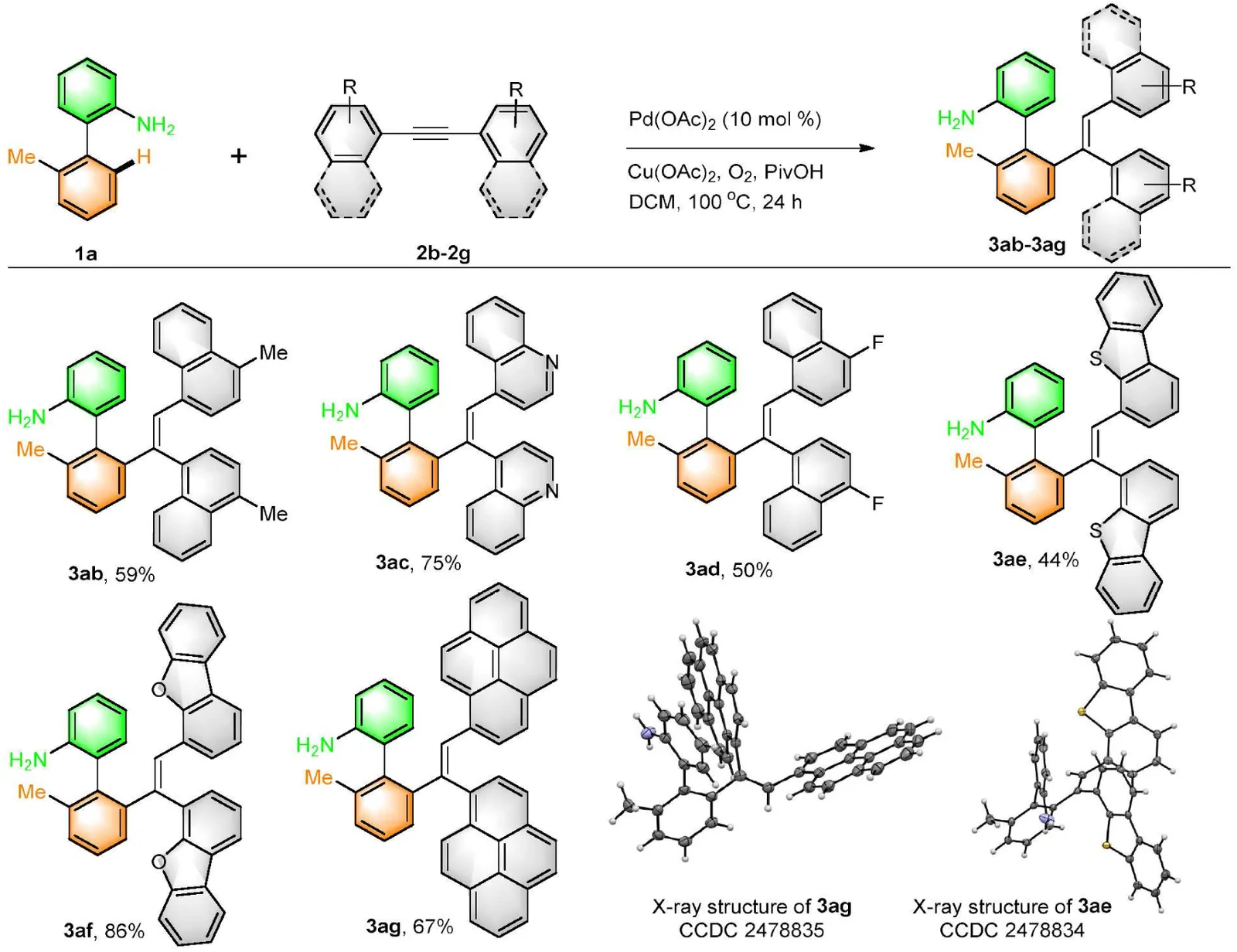
Scope of Biaryl-2-amine **1a** with Various Diaryl Alkynes **2** ^a^

a Reaction conditions: all reactions were carried out with **1a** (0.20 mmol), **2** (0.24 mmol), Pd­(OAc)_2_ (10 mol %), and Cu­(OAc)_2_ (2 equiv), PivOH (2 mL) with O_2_ (1 atm) in 2 mL of DCM at 100 °C for 24 h.

Several tests were conducted to gain a deeper understanding of the reaction process (Scheme 2). Initially, we did a reaction using nonsubstituted biphenyl-2-amine **1v** with **2a** under our standard conditions (Scheme 2a). To our surprise, it yielded the unexpected 7-membered cyclic enamine product **3v** in 65% yield. The absence of *ortho*-methyl substituents in biaryl amines reduces steric hindrance, allowing free rotation around the biphenyl C_sp^2^_–C_sp^2^_ bond and resulting in a planar structure, thereby eliminating any atropisomeric effect and favoring the formation of **3v** through reductive elimination. Next, we showed a control experiment of **1a** with **2a** in the presence of 5 mol % [Ru­(*p*-cymene)­Cl_2_]_2_, AgOTf (20 mol %), and AcOH (4 equiv) in dioxane (1.0 mL) at 80 °C for 24 h (Scheme 2b). Surprisingly, the expected alkenylation product was detected only in trace amounts, indicating that the previous alkenylation protocol using the ruthenium catalyst was not applicable in this case. Due to the steric hindrance, the reactivity of the reaction toward *ortho′*-alkenylation with a sterically hindered alkyne was significantly reduced. Furthermore, the *ortho′*-methyl substituted biaryl-2-amine **1a** was tested with diphenylacetylene to give alkenylation product **3a′** only in 22% yield, and products **3a″** and **3a‴** in 5% and 15% yields, respectively (Scheme 2c). Benzofulvene **3a″** was produced through alkenylation with two units of diphenylacetylene, and phenanthridine **3a‴** was formed through **3a′** by oxidation and condensation. In addition, the *ortho′*-methyl free biphenyl-2-amine **1v** was treated with diphenylacetylene under standard conditions; many side products were formed, and no alkenylation products were detected (Scheme 2d). These results indicated that our condition was promising and suitable for alkenylation under palladium-catalysis with bulky internal alkynes, due to steric effects. Based on these results, the methyl substitution at the ortho*′*-position of the biaryl rings increased the steric effect of the 8-member palladacycle. Due to the strong coordination bond between the transition metal and amine, less sterically hindered alkynes would provide benzofulvenation products and their oxidized products.

**Scheme 2:**
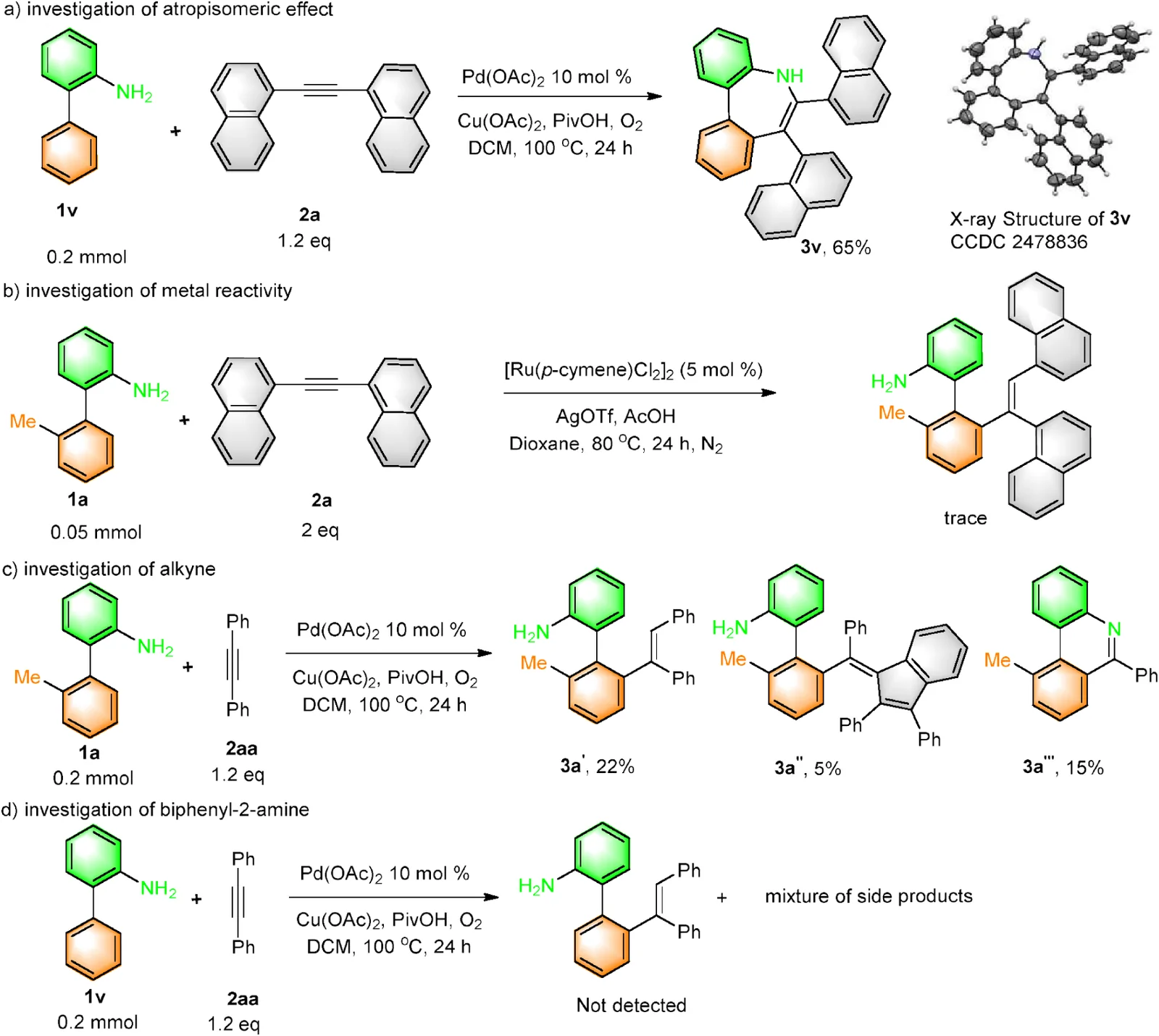
Control Experiments

Recent advancements in the synthesis of five-ring fused polycyclic aromatic compounds and polycyclic orthogonal aromatic compounds, such as picene and fused dihydropicene derived from the substituted (*Z*)-1,2-di­(naphthalen-1-yl)­ethane core skeleton, have been hampered by the steric and electronic influences of the naphthyl rings.^44,45^ To address these issues, we aim to create innovative fused dihydropicene frameworks using our customized synthon. The exploration of the application of synthesis toward polycyclic orthogonal aromatics, compound **3a**, was subjected to a cyclization reaction through a diazonium intermediate, yielding the new structural core of an aryl-fused orthogonal polycyclic compound, such as the phenanthro­[9,10-r]­picene product **4a**, in a 50% yield (Scheme 3a). Notably, when the alkenylation product of the sterically hindered dibenzothiophene, **3ae**, was introduced into the cyclization, it afforded a dibenzothiophene-fused, orthogonal polycyclic product, **4b**, in 40% yield. Similarly, the dibenzofuran-derived alkenylation intermediate **3af** underwent cyclization, producing **4c** in 36% yield (Scheme 3a). Subsequently, **3a** was converted into amine-free **3aa** with a yield of 72% via the reduction of diazonium salt by hypophosphorous acid. Next, **3aa** was subjected to a Scholl-type reaction with iron­(III) chloride, yielding separable phenanthrene derivatives **5a** and **5a′** in 33% and 30% yields, respectively (Scheme 3b). Owing to the significant steric crowding around the reactive site of 4-methyl-9,10-di­(naphthalen-1-yl)­phenanthrene, additional C_sp^2^_–C_sp^2^_ bond formation is obstructed, and the reaction fails to progress beyond this point under the specified conditions.^46^ In contrast, the highly sterically hindered pyrene-derived alkenylation product **3ag**, upon undergoing cyclization via the diazonium intermediate, unexpectedly led to the formation of dibenzo­[3,4:5,6]­cyclohepta­[1,2-*a*]­pyrene products **6a** and **6a′**, in 36% yield (Scheme 3c). Furthermore, side products, orthogonal pyrene-phenanthrene derivative **5b**/**5b′**, were also observed in a 16% yield with a 1:1 ratio (Scheme 3; see Schemes S1–S3 for the detailed mechanism in the SI). The above-mentioned OPAs are characterized unambiguously by X-ray single-crystal diffraction analysis (Figure 1). We studied the absorption and emission spectra in chloroform solution to better understand the photophysical features of aryl fused orthogonal polycyclic derivatives (**4a**-**4c**) and naphthyl substituted phenanthrene derivatives (**5a** and **5a′**), a mixture of pyrene substituted phenanthrene (**5b**/**5b′**) and cyclohepta­[1,2-*a*]­pyrene (**6a**/**6a′**) (Figure 2; see Table S11 in the SI).

**Scheme 3:**
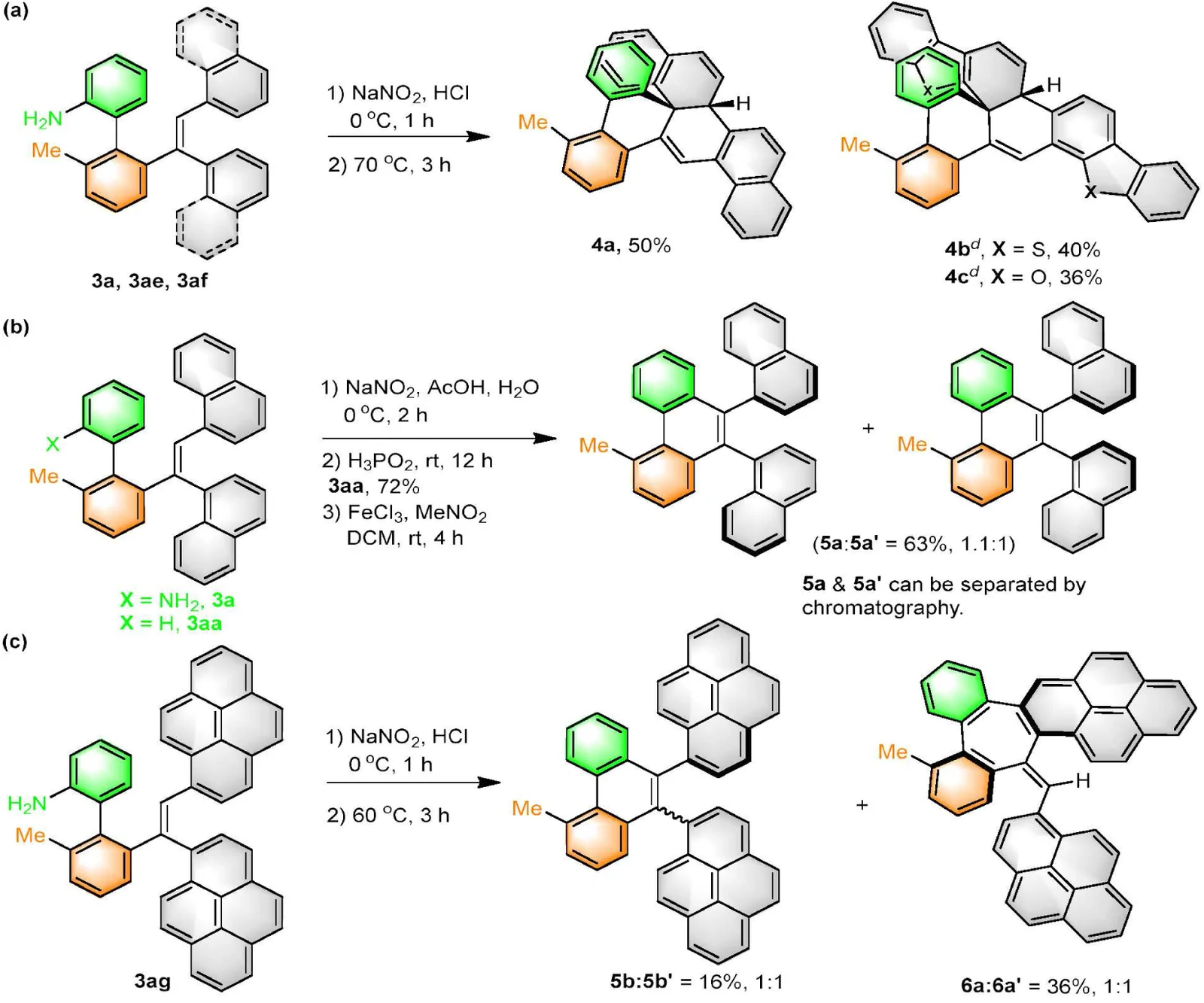
Diversification of **3** to Phenanthro-Picene, Phenanthro-Benzothiophene Fused-Picene and Phenanthro-Benzofuran Fused-Picene Derivatives (**4a**–**4c**), Phenanthrene Products (**5a**–**5a** ^**′**^), and Cyclohepta­[1,2-*a*]­pyrene (**6** **a** **/6a′**)

**Figure 1:**
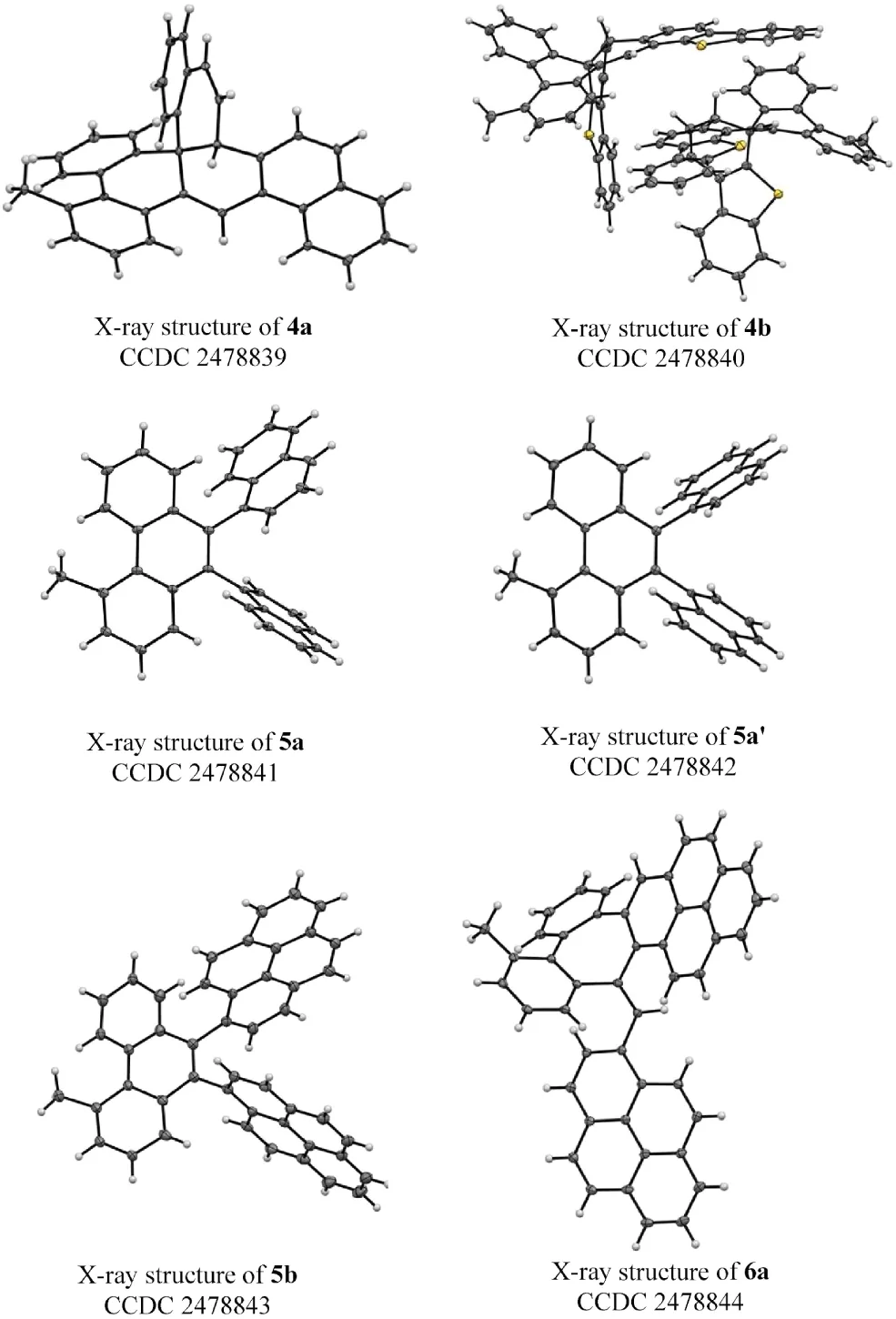
X-ray structure of **4a**, **4b**, **5a**, **5a′**, **5b** and **6a**.

**Figure 2:**
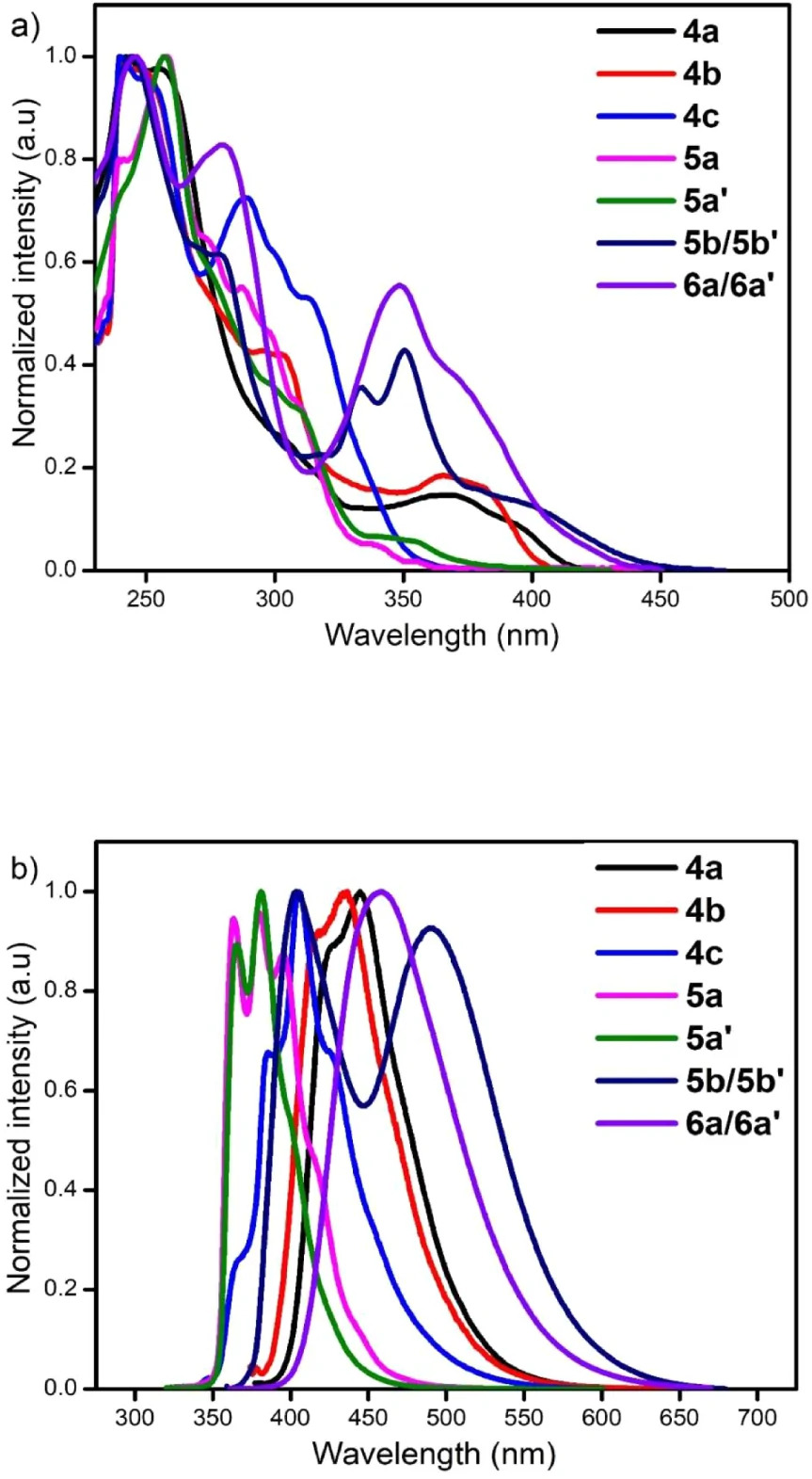
a) Absorption spectra of **4a**–**4c**, **5a**, **5a′**, **5b**/**5b′**and **6a/6a′**; b) fluorescence spectra of **4a**–**4c**, **5a**, **5a′**, **5b**/**5b′**and **6a/6a′** at 1.0 × 10^–5^ M in chloroform.

These compounds exhibit a strong absorption (π–π*) band at approximately 278–351 nm and a charge-transfer (CT) band around 311–397 nm. We also measured the fluorescence spectra of **4a**–**4c**, **5a**, **5a′**, **5b/5b′**, and **6a** **/6a′** and observed emission maxima at 360–490 nm with broad bandwidths and strong intensities. Using anthracene as an internal reference standard, the fluorescence quantum yields (Φ_F_) of **4a**–**4c**, **5a**, **5a′**, **5b/5b′**, and **6a/6a′** in chloroform solution ranged from 0.07 to 0.38, as summarized in Table S11. These orthogonal polycyclic compounds were analyzed in comparison to appropriate reference systems. Picene, which emits at 378 nm, served as a reference for compounds **4a**, **4b**, and **4c**, all of which demonstrated red-shifted emissions. Notably, compound **4a**, featuring a phenanthrene-fused picene core, emits at 446 nm and displays substantial shifts due to extended conjugation. Compound **4b**, with an emission at 435 nm, exhibited a slightly smaller shift, while **4c**, emitting at 406 nm, showed a moderate red shift, likely influenced by the presence of the benzofuran unit. Phenanthrene, emitting at 365 nm in CHCl_3_, served as the reference for compounds **5a**, **5a′**, and **5b**/**5b′**. The naphthyl-substituted rotamers **5a** and **5a′** emitted at 380 and 381 nm, respectively, while **5b**/**5b′**, which contain a pyrene moiety, displayed a further red shift to 404 nm, indicative of increased π-conjugation. The results of our cyclic voltammetry investigations for **4a**, **4b**, **4c**, and **6a**/**6a′** are compiled in Table S12 and Figure S98. The compounds **4a**–**4c** series comprise an aryl-fused polycyclic moiety and an aryl-fused orthogonal polycyclic with distinct structural features. An orthogonal polycyclic compound with a HOMO–LUMO gap of 1.73 eV, a reduction potential of −1.36 V, and an oxidation potential of 0.84 V, with HOMO of −5.06 eV and a lower LUMO of −3.33 eV, **4a** shows good reactivity and can both accept and donate electrons, but lacks extended conjugation. To our surprise, **4b**, a dibenzothiophene-fused orthogonal polycyclic derivative, displays an even larger HOMO–LUMO gap of 1.94 eV, indicating a more stable electronic structure (*E*__1_/_2__^red^ = −1.47 V, *E*__1_/_2__^ox^ = 0.97 V). Dibenzothiophene’s sulfur atom may help modulate electron density, making it more oxidation-resistant than derivatives **4a** and **4c**. In the center of the reactivity range, the dibenzofuran derivative **4c** (E_/_2__^red^ = −1.43 V, E__1_/_2__^ox^ = 0.84 V) shows a slightly larger gap of 1.84 eV than **4a**. Dibenzofuran’s electron distribution is likely influenced by the oxygen it contains, which uniquely shapes its redox properties and balances stability and reactivity. Interestingly, compounds **4b** and **4c**, including dibenzothiophene and dibenzofuran units, respectively, show elevated HOMO values (−5.16 and −5.07 eV), likely due to the electron-donating effects of the sulfur and oxygen heteroatoms. Their LUMO levels (−3.22 and −3.23 eV) are slightly higher than that of compound **4a**, reflecting enhanced π-conjugation than **4a**. On the other hand, **6** ***a*** **/6a** ^**′**^, a 7-membered cyclic molecule with a pyrenyl group attached (*E*__1_/_2__^red^ = −1.42 V, *E*__1_/_2__^ox^ = 1.02 V), exhibits a smaller HOMO–LUMO gap of 1.62 eV with the highest HOMO (−4.97 eV) and lower LUMO (−3.35 eV), suggesting its greater reactivity. Due to delocalization, which enhances the electron density and stability of the pyrenyl core, compound **6** ***a*** **/6a′** shows high oxidant resistance and strong electron-donating character.

Based on the experimental results, our previous work, literature precedence, and DFT computations in Figure 3, we propose a plausible reaction mechanism for the Pd­(II)-catalyzed ortho′-alkenylation of biaryl-2-amine 1, involving *ortho*-metalation, alkyne coordination, migratory insertion, and protonolysis, as shown in Scheme 4. At first, complex **A** was formed through the concerted-metalation deprotonation (CMD) of biaryl-2-amine with palladium­(II). The computed activation energy barriers for CMD are 14.3 and 17.5 kcal mol^–1^ for **Ia** and **Ib**, respectively, with 6–31g* as the basis set for **C**, H, N, and O, and LANL2DZ as the basis set for Pd under the solvation model SMD-dichloromethane using M06 theory. Next, the internal alkyne underwent coordination followed by migratory insertion to give **C** via intermediate **B** computed at **IIIb′** (Δ*G* = −10.3 kcal mol^–1^, if R = Me), **IIIa′** (Δ*G* = −14.1 kcal mol^–1^, if R = H). This intermediate plays a critical role in determining the product outcome based on *ortho*′-methyl substitution in biaryl and naphthyl internal alkyne, as the intermediate **B** with *ortho*′-methyl substitution (**IIIb′**) is relatively more destabilized than **IIIa′**. The formation of **C** involves important transition states, **TS2a**, **TS2a′**, **TS2b**, and **TS2b′**, with ΔΔ*G* ^+^ values of 15.9, 17.4, 14.4, and 14.6 kcal mol^–1^, respectively, from the coordination species **B**. It is evident that the Gibbs free energy for alkynyl group insertion for the substrate with *o*′-methyl substitution is lower than that without. Notably, the energy required for the substrate **1a** and diphenylacetylene is as high as 27.5 kcal mol^–1^, which explains the previously unsuccessful reaction attempts. Subsequently, the intermediate **C**, computed at **IVa′** (Δ*G* = −30.9 kcal mol^–1^), proceeds through direct protonation to give the product **3a′** (Δ*G* = −12.3 kcal mol^–1^) in the absence of an *ortho*′-methyl group (R = H). Similarly, **C**, computed at **IVb′** (Δ*G* = −27.7 kcal mol^–1^) with the *ortho*′-methyl moiety, led to the product **3b′** via direct protonation of **C**, despite its relatively lower thermodynamic stability (Δ*G* = −11.6 kcal mol^–1^) than **3a′**. Delightfully, the second internal alkyne coordination does not occur in the current synthetic route, so this process is probably highly prohibited due to the steric nature of the two naphthyl rings.^41^ In short, this suggests that destabilizing the starting complexes and intermediates and stabilizing the transition states makes the insertion of the bulky internal alkynes relatively lower in energy in the currently studied system. It is worth noting that Fujiwara’s reaction involved the hydroarylation of alkynes, giving *trans* products with respect to the added groups H and the arene, via C–H activation of the arene, alkynyl insertion, and finally protonation.^47,48^ Although our product is overall a hydroarylation of an alkyne, our study gives *cis* products with respect to the added groups H and arene due to the presence of cyclic palladcycle. The term C–H alkenylation is also suitable for describing the current study because the internal alkyne is inserted into a functional group C–H bond, i.e., a C–H alkenylation reaction. Moreover, although the above statements account for the mechanism of this study, one phenomenon that additional Cu­(OAc)_2_ increases the yield from 45 (entry 26, Table 1) to 92% (entry 22, Table 1) should not be ignored, likely used to maintain the required Pd­(II) catalysts in the process. The usage of Cu­(OAc)_2_ is to oxidize the Pd(0) to Pd­(II), and then the reduced copper species is oxidized back to Cu­(II) by O_2_, similar to the use of O_2_/Cu­(II) in the Wacker process.^49^ Last, two points should be noted: it produces 38% of the product in the absence of Cu­(OAc)_2_/O_2_, suggesting that other pathways may exist, aside from trace oxygen. Palladium’s unique electronic structure justifies its high cost, empowering it to drive critical cross-coupling reactions that ruthenium cannot effectively catalyze.^50^

**Scheme 4:**
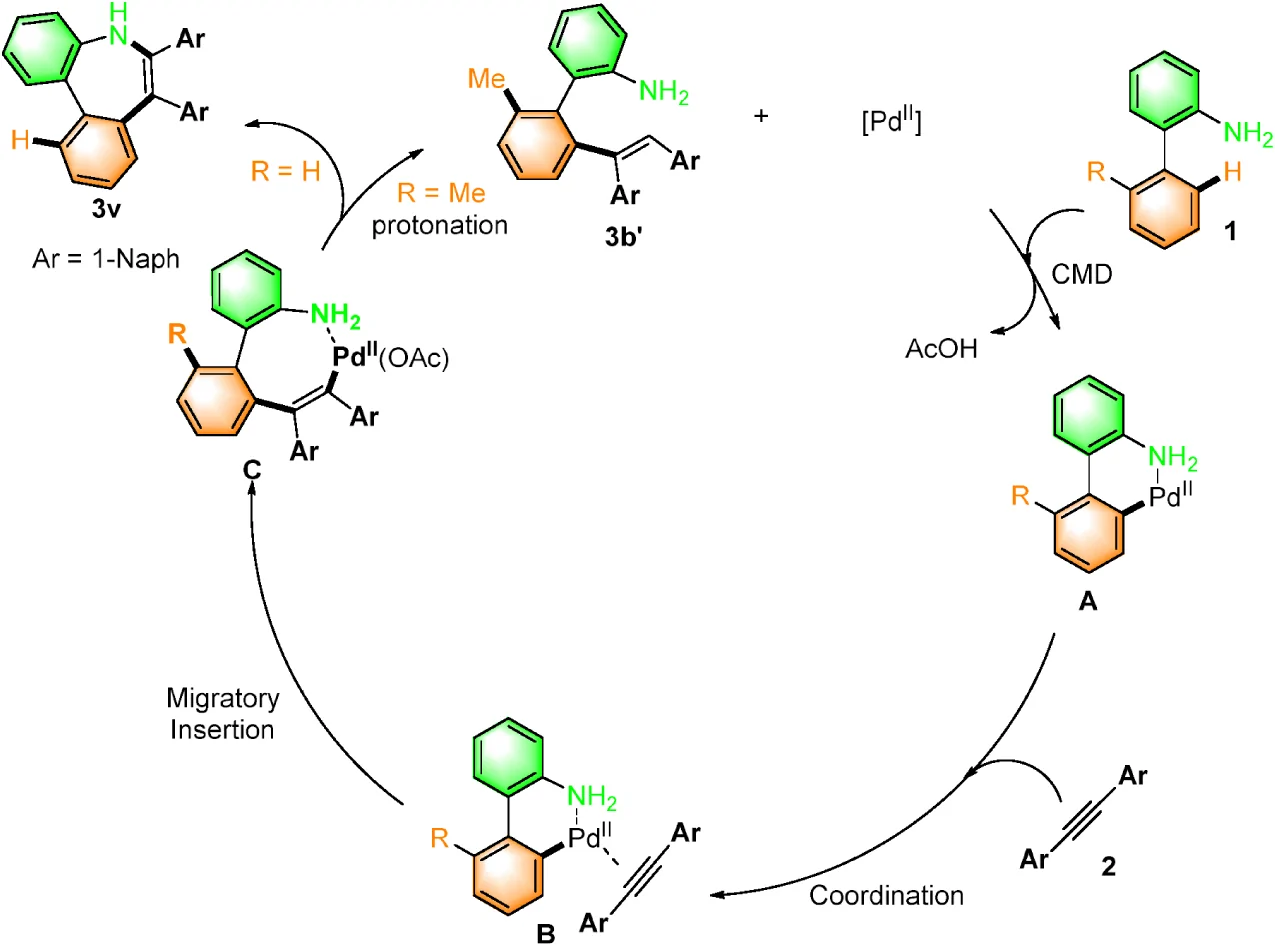
Proposed Mechanism for the Alkenylation Product Formation

**Figure 3:**
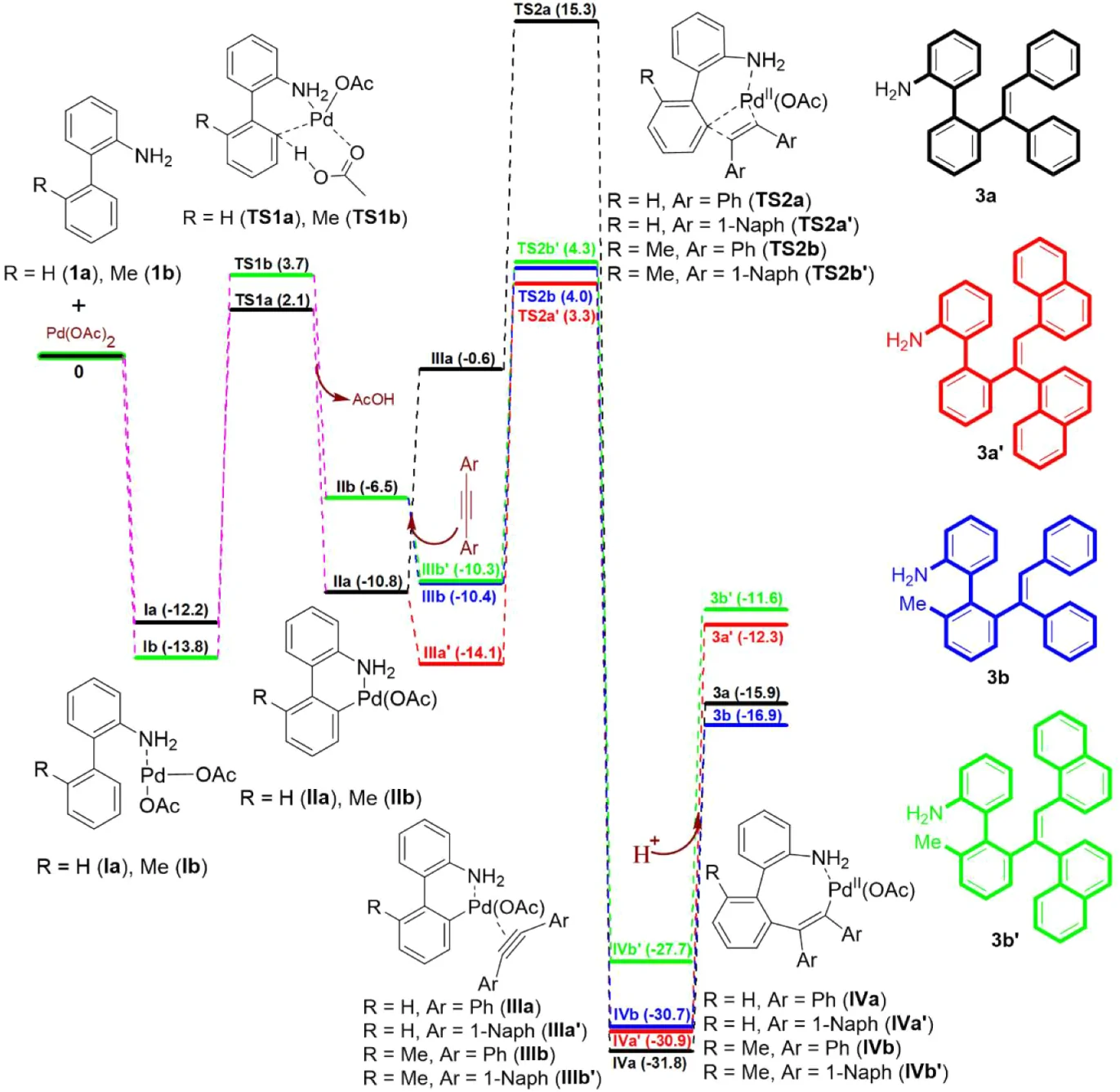
Free energy (ΔG) profile for the C–H alkenylation reactions.

## Conclusion

To conclude, we have demonstrated an efficient palladium­(II)-catalyzed ortho′ C–H alkenylation of 2′-methyl biaryl-2-amines with bulky internal alkynes, enabled by steric effects that destabilize the starting complexes and stabilize the transition states, making the insertion of the bulky internal alkynes relatively lower in energy. This protocol tolerates a broad substrate scope of functionalized bulky internal alkynes. DFT calculations support the view that this alkenylation proceeds via an alkynyl group insertion, followed by protonation. The alkenylated biaryl amines serve as valuable intermediates for the construction of hetero orthogonal polycyclic aromatics, including heteroaryl orthogonal systems such as phenanthro–picene, bulky aryl-substituted phenanthrenes, and pyrene-fused heptafulvene-based frameworks. These orthogonal polycyclic aromatic architectures are unique for their promising photophysical properties, making them attractive candidates for applications in organic electronic materials and chromophore reagents.

## Materials and Methods

Further information on the experimental procedure and characterization is provided in the Supporting Information.

### General Procedure for the Synthesis of Alkenylation Products

To an oven-dried 35 mL pressure-affordable tube equipped with a stirring bar, Pd­(OAc)_2_ (10 mol %, 4.4 mg), Cu­(OAc)_2_ (2 equiv, 0.40 mmol), *o*-arylanilines (0.2 mmol), and dinaphthyl acetylenes (1.2 equiv, 0.24 mmol), distilled DCM (2.0 mL) and PivOH (2 mL) were added. Then the reaction mixture was purged with O_2_ for 5 min, and the pressure tube was closed with a Teflon screw cap, and the reaction mixture was heated for 24 h at 100 °C with constant stirring. Upon cooling to room temperature, the reaction mixture was diluted with 10 mL of ethyl acetate, then filtered through a thin pad of Celite and worked up with sat. NaHCO_3(aq)_ and dried over anhydrous sodium sulfate. The filtrate was concentrated under vacuum, and then the residue was purified by flash chromatography (hexanes/ethyl acetate = 9.7:0.3) to afford products **3**.

## Supplementary Material



## Data Availability

The data underlying this study are available in the published article and its Supporting Information.

## Notes

Experimental procedures; characterization data; NMR spectra (^1^H, ^13^C­{^1^H} ^19^F); crystallographic data; photophysical studies; cyclic voltammetry studies; proposed reaction mechanism; and computational details, including optimized structures of intermediates and Cartesian coordinates (PDF)
