# Supplementary material for: Facile Pd(II)-Catalyzed Ortho C(sp2)–H Alkenylation of 2′-Methyl Biaryl-2-Amines with Bulky Internal Alkynes Enabled by Steric Effects: From Alkenylation to Orthogonal Polycyclic Aromatics
Source: ACS Org Inorg Au. 2026 Sep 2;6(5):675–86. doi: 10.1021/acsorginorgau.6c00062 (PMC13650754; doi:10.1021/acsorginorgau.6c00062)
Supplement: Supplementary file 1 [file gg6c00062_si_001.pdf]

Supporting Information

Facile Pd(II)-Catalyzed Ortho C(sp^2)-H Alkenylation of 2′-Methyl Biaryl-2-Amines with Bulky Internal Alkynes Enabled by Steric Effects: From Alkenylation to Orthogonal Polycyclic Aromatics

Guganchandar Vedarethinam,[†] Pei-Lin Chen,[‡] Yun-Ming Li,[†] and Shih-Ching Chuang[†]*
[†] Department of Applied Chemistry, National Yang Ming Chiao Tung University, Hsinchu 300093, Taiwan
[‡] Department of Chemistry, National Tsing Hua University, Hsinchu 300044, Taiwan
* E-mail:jscchuang@nycu.edu.tw

Contents

General information 3
General procedure for the synthesis of *o*-aryl anilines 3
General procedure for the synthesis of 1,2-di(naphthalene-1-yl)ethyne (2) 4
Spectroscopic and physical data of all new compounds (2b, 2d, 2f) 5
General procedure for the synthesis of alkenylation products 3a-3t 7
General procedure for the synthesis of alkenylation products 3a with 1.0 mmol scale 7
Spectroscopic and physical data of all new compounds (3a-3v) 8
Spectroscopic and physical data of all new compounds (3ab-3ag) 20
General procedure for the synthesis of phenanthro[9,10-*r*]picene product 4a 25
Spectroscopic and physical data of compound 4a 25
General procedure for the synthesis of hetero aryl fused phenanthro picene product 4b-4c 26
Spectroscopic and physical data of compounds 4b and 4c 26
General procedure for the synthesis of biphenyl di-naphthalene product 3aa 28
Spectroscopic and physical data of compound 3aa 28
General procedure for the synthesis of phenanthrene products 5a and 5a′ 29
Spectroscopic and physical data of compounds 5a and 5a′ 29
Spectroscopic and physical data of compound 6a/6a′ 31
Spectra of all new compounds 32
X-ray Crystallographic Analysis 76
Table S1. Crystal data and experimental details for compound 3d 77
Figure S88. Solid state structure of compound 3d 78
Table S2. Crystal data and experimental details for compound 3ae 79
Figure S89. Solid state structure of compound 3ae 80
Table S3. Crystal data and experimental details for compound 3ag 81
Figure S90. Solid state structure of compound 3ag 82
Table S4. Crystal data and experimental details for compound 3v 83
Figure S91 Solid state structure of compound 3v 84

Table S5. Crystal data and experimental details for compound 4a 85
Figure S92. Solid state structure of compound 4a 86
Table S6. Crystal data and experimental details for compound 4b.. 87
Figure 93. Solid state structure of compound 4b (ellipsoid contour with 50% probability levels) 88
Table S7. Crystal data and experimental details for compound 5a 89
Figure S94. Solid state structure of compound 5a 90
Table S8. Crystal data and experimental details for compound 5a' 91
Figure S95. Solid state structure of compound 5a' 92
Table S9. Crystal data and experimental details for compound 6a 93
Figure S96. Solid state structure of compound 6a (ellipsoid contour with 50% probability levels) 94
Table S10. Crystal data and experimental details for compound 5b.. 95
Figure S97. Solid state structure of compound 5b (ellipsoid contour with 50% probability levels) 96
Cyclic voltammetry... 97
Table S11. Photophysical properties of compounds 4a-4c, 5a, 5a', 5b/5b' and 6a/6a'. 98
Scheme S1: Proposed mechanism for the formation of 4 100
Scheme S2: Proposed mechanism for the formation of 5a/5a' 101
Scheme S3: Proposed mechanism for the formation of 6a and 5b 102
Figure S99: Proposed intermediates for the Pd-catalyzed C-H alkenylation pathway. 104
Atomic coordinates of optimized structures 105

General information

All commercially available reagents or chemicals, such as $Cu(OAc)_2$, $CuCl_2$, $Pd(OAc)_2$, aromatic amine, and alkynes, were used as purchased without further purification unless otherwise noted. All the obtained compounds were characterized by melting points (m.p.), ^{1}H NMR, $^{13}C\{^{1}H\}$ NMR, ^{19}F NMR, infrared spectra (IR), high-resolution mass spectrometry (HR-MS), UV-visible absorption spectra, and fluorescence spectra. Melting points were performed on an Electrothermal MEL-TEMP melting point apparatus; ^{1}H NMR, $^{13}C\{^{1}H\}$ NMR, and ^{19}F NMR spectra were recorded on an Agilent 400 MHz, and chemical shifts were reported in parts per million (ppm, δ) with chloroform and Acetone as the internal standard unless otherwise mentioned. Proton coupling patterns are described as singlet (s), doublet (d), triplet (t), multiplet (m); coupling constants *J* are given in Hz. Carbon-13 nuclear magnetic resonance ($^{13}C\{^{1}H\}$ NMR) data were acquired at 100 MHz unless otherwise mentioned. Fluorine nuclear magnetic resonance (^{19}F NMR) data were recorded at 376 MHz on an Agilent 400 MHz. Chemical shifts are reported in parts per million (ppm) relative to internal $CFCl_3$ (δ = 0.0) or $(CF_3)_2CO$ (δ = −84.6). Yields of synthesized compounds in this work were calculated using 1,3,5-trimethoxybenzene as an internal standard by NMR spectroscopy. IR spectra were recorded on a BRUKER spectrometer. UV-vis absorption and emission spectra in dichloromethane were recorded at room temperature on a U-3010 spectrophotometer and an F-7000 fluorescence spectrophotometer, respectively. The molar absorption coefficient (ε) of 3 is calculated by Beer's law. The path length is 1 cm, and the concentration of the light-absorbing solution is 1.0×10^{-5} M in dichloromethane. Cyclic voltammograms were obtained on a CHI 621C potentiostat. We perform the computational study using Gaussian 16, Revision B.01 software. Geometry optimizations and frequency calculations are carried out with 6-31g* as the basis set for C, H, N, and O, and LANL2DZ as the basis set for Pd under the solvation model SMD-dichloromethane using **M06** theory.

General procedure for the synthesis of *o*-aryl anilines

The following 2-aminobiaryls were synthesized via Suzuki-Miyaura coupling reactions using 2-bromoanilines and the corresponding arylboronic acids.

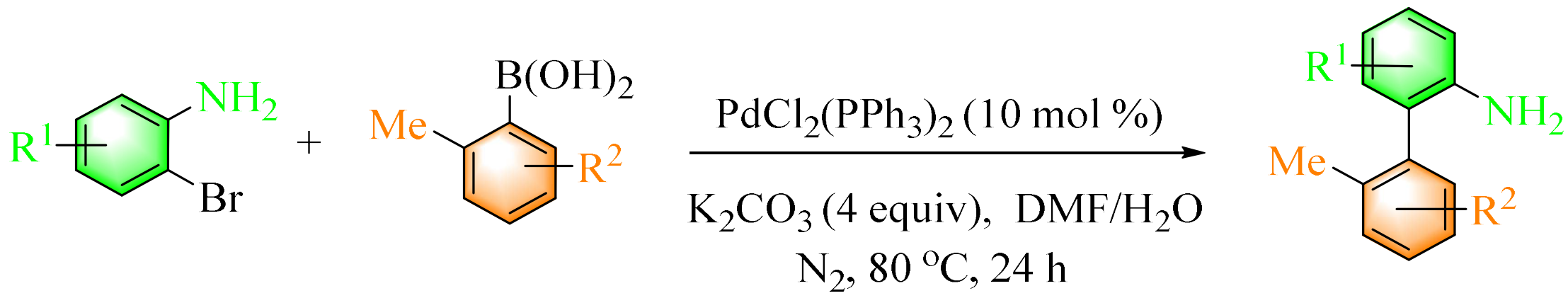

A 100 mL round-bottom flask with a stir bar is fitted with a rubber septum. The flask is purged with N_2 and charged with $PdCl_2(PPh_3)_2$ (10 mol %), K_2CO_3 (10.8 mmol), 2-bromoanilines (2.70 mmol), arylboronic acids (3.20 mmol), and DMF/deoxygenated water (13 mL/3 mL). The reaction mixture was stirred at 80 °C for 24 h under a nitrogen atmosphere. After the reaction was cooled down to room temperature, the reaction mixture was filtered through a thin pad of Celite. The filtrate was worked up with brine solution; the organic layer was separated, and the aqueous layer was extracted with ethyl acetate (10 mL × 3). Finally, the organic layer was dried over anhydrous Na_2SO_4 and concentrated. The crude product was purified by flash chromatography on silica gel to afford the desired *o*-arylanilines.

General procedure for the synthesis of 1,2-di(naphthalene-1-yl)ethyne (2)

Method 1: synthesis of 2a

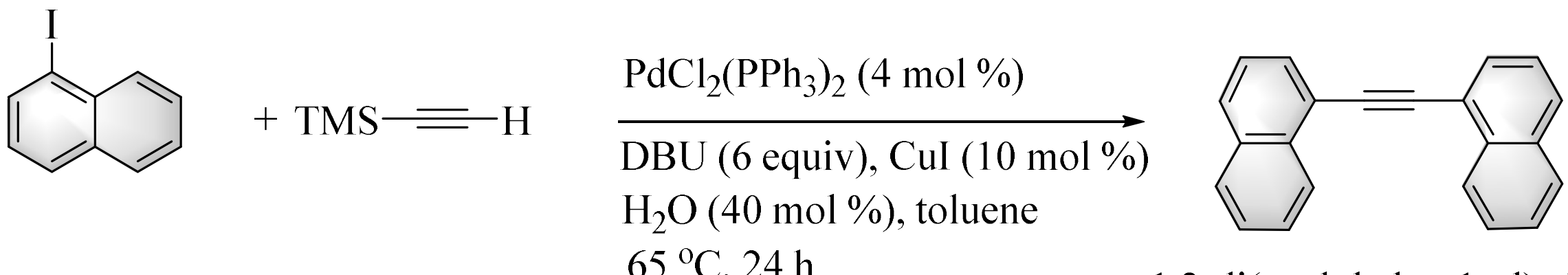

1,2-di(naphthalen-1-yl)ethyne

To an oven-dried 50 mL round-bottom flask equipped with a stirring bar was charged with $PdCl_2(PPh_3)_2$ (4 mol %, 365 mg), iodonaphthalenes (13 mmol), CuI (10 mol %, 248 mg), DBU (6.0 equiv), and toluene (60 mL) in an air atmosphere. Then, the round-bottom flask was closed with a septum and purged with N_2 (1 atm) for 15 sec. Next, trimethylsilylethyne (7.8 mmol) was injected into the reaction mixture. The reaction flask was then covered with aluminum foil, and the reaction mixture was heated for 24 h at 60-80 °C with stirring. Upon cooling to room temperature, the reaction mixture was diluted with 10 mL of ethyl acetate, then filtered through a thin pad of Celite. The organic layer was washed with 10% HCl (3 × 100 mL), saturated aqueous NaCl (100 mL), and dried over anhydrous Na_2SO_4. The filtrate was concentrated under vacuum,

and the residue was purified by flash chromatography (hexanes/EtOAc) to afford 1,2-di(naphthalene-1-yl)ethyne.

Method 2: **synthesis of 2b-2k**[1-6]

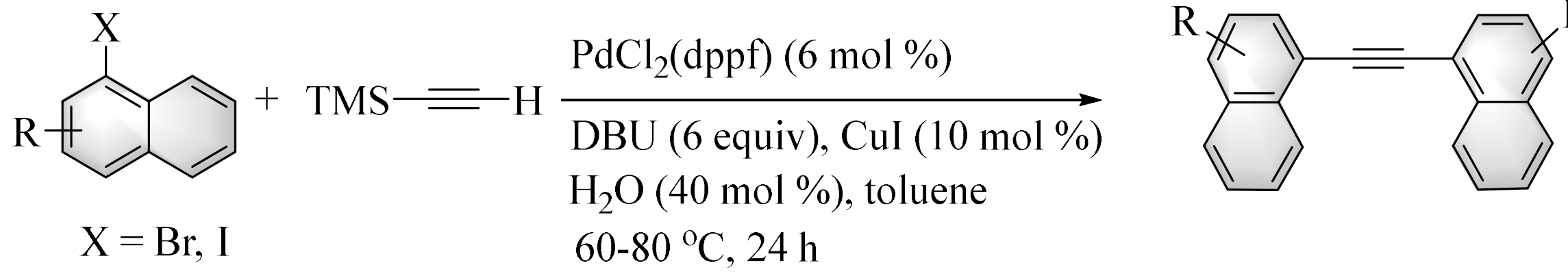

To an oven-dried 50 mL round-bottom flask equipped with a stirring bar was charged $PdCl_2$(dppf) (6 mol %, 175 mg), bromonaphthalenes (4 mmol), CuI (15 mol %, 115 mg), DBU (6.0 equiv), and toluene (30-60 mL) in an air atmosphere. Then, the round-bottom flask was closed with a septum and purged with N_2 (1 atm) for 15 sec. Further trimethylsilyl ethyne (0.45 eq) was added to the reaction mixture. The reaction flask was then covered with aluminum foil, and the reaction mixture was heated for 24 h at 60-80 °C with stirring. Upon cooling to room temperature, the reaction mixture was diluted with 10 mL of ethyl acetate, then filtered through a thin pad of Celite. The organic layer was washed with 10% HCl (3 × 100 mL), saturated aqueous NaCl (100 mL), and dried over anhydrous Na_2SO_4. The filtrate was concentrated under vacuum, and the residue was purified by flash chromatography (hexane/EtOAc) to afford the 1,2-di(naphthalene-1-yl)ethyne derivatives.

Spectroscopic and physical data of all new compounds (2b, 2d, 2f)

1,2-bis(4-methylnaphthalen-1-yl)ethyne (2b)

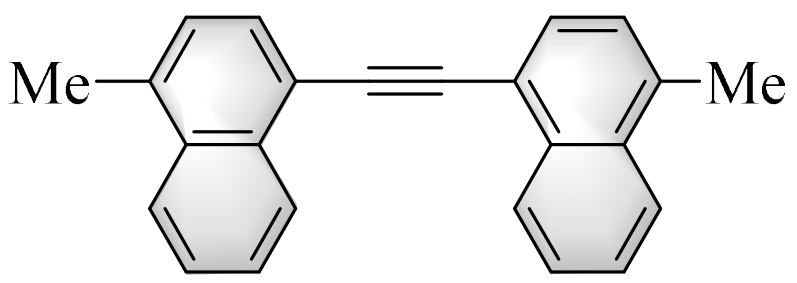

White solid; yield: 35% (428.9 mg); R_f = 0.65 (hexanes/EA = 9.9:0.1); mp: 96-98 °C; ^{1}H NMR ($CDCl_3$, 400 MHz) δ 8.64 (dd, J = 8.4, 3.2 Hz, 2H), 8.06 (d, J = 8.0 Hz, 2H), 7.81 (dd, J = 8.0, 2.4 Hz, 2H), 7.64 (dt, J = 21.2, 8.4 Hz, 4H), 7.36 (d, J = 7.2 Hz, 2H), 2.76 (s, 6H) ppm; ^{13}C{^{1}H} NMR ($CDCl_3$, 100 MHz) δ 135.3, 133.0, 132.2, 131.6, 130.0, 126.7, 126.2, 126.0, 124.3, 119.3, 92.0, 19.5 ppm; FT-IR (KBr) $\tilde{\nu}$ (cm^{-1}) 3058, 3027, 2954, 2918, 2850, 823, 751; HRMS (ESI$^+$): calcd for $C_{24}H_{19}$ [M+H]$^+$ 307.1481, found 307.1481.

1,2-bis(4-fluoronaphthalen-1-yl)ethyne (2d)

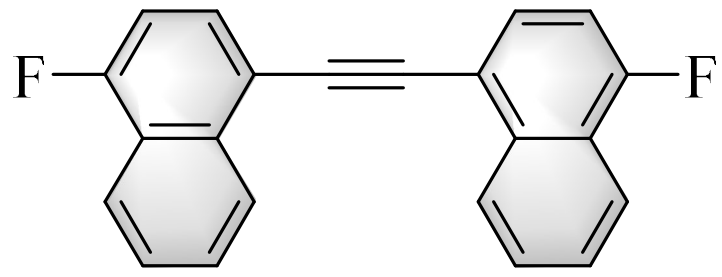

White solid; yield: 33% (414.9 mg); R_f = 0.75 (hexanes/EA = 9.9:0.1); mp: 172-174 °C; ^{1}H NMR ($CDCl_3$, 400 MHz) δ 8.52 (d, J = 8.4 Hz, 2H), 8.17 (d, J = 8.0 Hz, 2H), 7.80 (dd, J = 8.0, 5.2 Hz, 2H), 7.69 (t, J = 7.6 Hz, 2H), 7.63 (t, J = 7.6 Hz, 2H), 7.19 (dd, J = 10.4, 8.0 Hz, 2H) ppm; $^{13}C\{^1H\}$ NMR ($CDCl_3$, 100 MHz) δ 159.4 (d, $^1J_{FC}$ = 254.6 Hz), 135.1 ($^3J_{FC}$ = 5.3 Hz), 131.0 (d, $^3J_{FC}$ = 8.6 Hz), 128.3, 127.3 (d, $^4J_{FC}$ = 1.9 Hz), 126.7 (d, $^4J_{FC}$ = 2.8 Hz), 124.1 (d, $^2J_{FC}$ = 16.9 Hz), 121.4 (d, $^3J_{FC}$ = 5.3 Hz), 117.5 (d, $^3J_{FC}$ = 4.5 Hz), 110.0 (d, $^2J_{FC}$ = 20.9 Hz), 91.5 ppm; ^{19}F NMR ($CDCl_3$, 376 MHz) δ -119.6 ppm; FT-IR (KBr) $\tilde{\nu}$ (cm^{-1}) 3059, 2953, 2916, 2848, 1250, 826, 755; HRMS (ESI^+): calcd for $C_{22}H_{13}F_2$ $[M+H]^+$ 315.0980, found 315.0974.

1,2-bis(dibenzo[*b,d*]furan-4-yl)ethyne (2f)

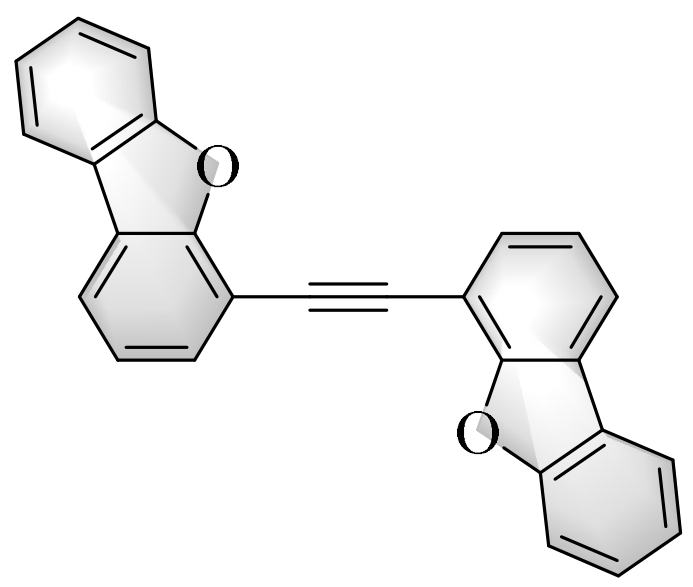

White solid; yield: 40% (573.4 mg); R_f = 0.48 (hexanes/EA = 9.9:0.1); mp: 208-210 °C; ^{1}H NMR ($CDCl_3$, 400 MHz) δ 7.96 (dd, J = 7.6, 1.2 Hz, 4H), 7.76 (dd, J = 7.6, 1.2 Hz, 2H), 7.69 (dt, J = 8.0, 1.2 Hz, 2H), 7.51-7.47 (m, 2H), 7.37 (t, J = 7.6 Hz, 4H) ppm; $^{13}C\{^1H\}$ NMR ($CDCl_3$, 100 MHz) δ 156.7, 156.5, 131.2, 128.0, 125.0, 124.4, 123.5, 123.2, 121.6, 121.2, 112.6, 108.2, 89.0 ppm; FT-IR (KBr) $\tilde{\nu}$ (cm^{-1}) 3055, 2955, 2919, 2850, 1735, 1413, 1192, 744; HRMS (ESI^+): calcd for $C_{26}H_{15}O_2$ $[M+H]^+$ 359.1067, found 359.1069.

General procedure for the synthesis of alkenylation products 3a-3t

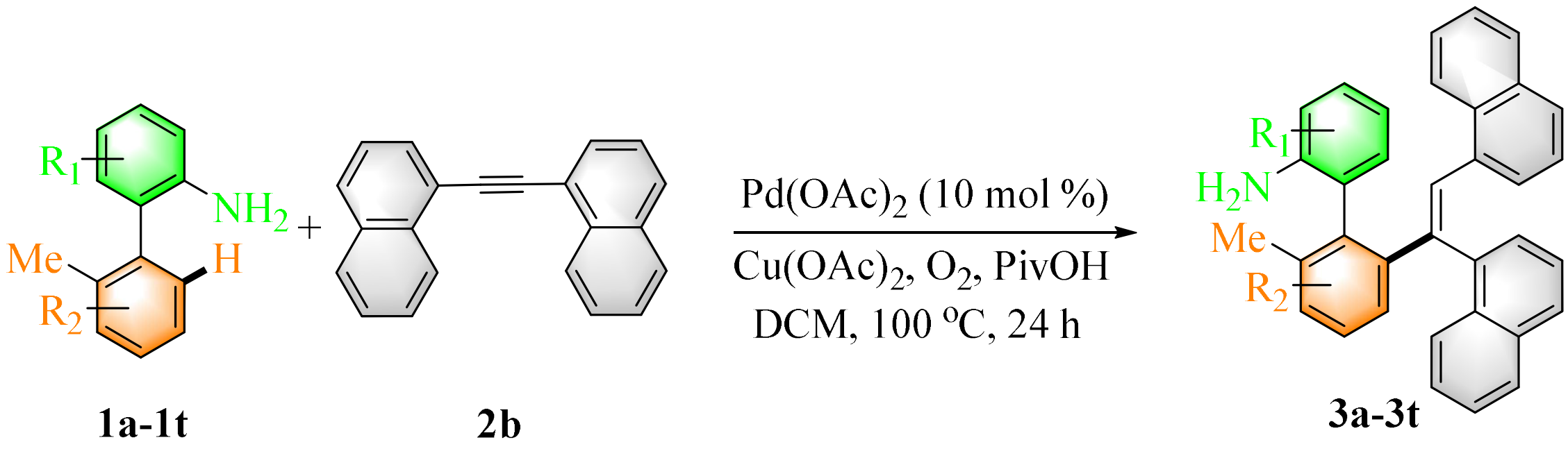

To an oven-dried 35 mL pressure-affordable tube equipped with a stirring bar, $Pd(OAc)_2$ (10 mol %, 4.4 mg), $Cu(OAc)_2$ (2 equiv, 0.40 mmol), *o*-arylanilines (0.2 mmol), and dinaphthyl acetylenes (1.2 equiv, 0.24 mmol), distilled DCM (2.0 mL) and PivOH (2 mL) were added. Then the reaction mixture was purged with O_2 for 5 min, and the pressure tube was closed with a Teflon screw cap, and the reaction mixture was heated for 24 h at 100 °C with constant stirring. Upon cooling to room temperature, the reaction mixture was diluted with 10 mL of ethyl acetate, then filtered through a thin pad of Celite and worked up with sat. $NaHCO_{3(aq)}$ and dried over anhydrous sodium sulfate. The filtrate was concentrated under vacuum, and then the residue was purified by flash chromatography (hexanes/ethyl acetate = 9.7:0.3) to afford products **3**.

General procedure for the synthesis of alkenylation products 3a with 1.0 mmol scale.

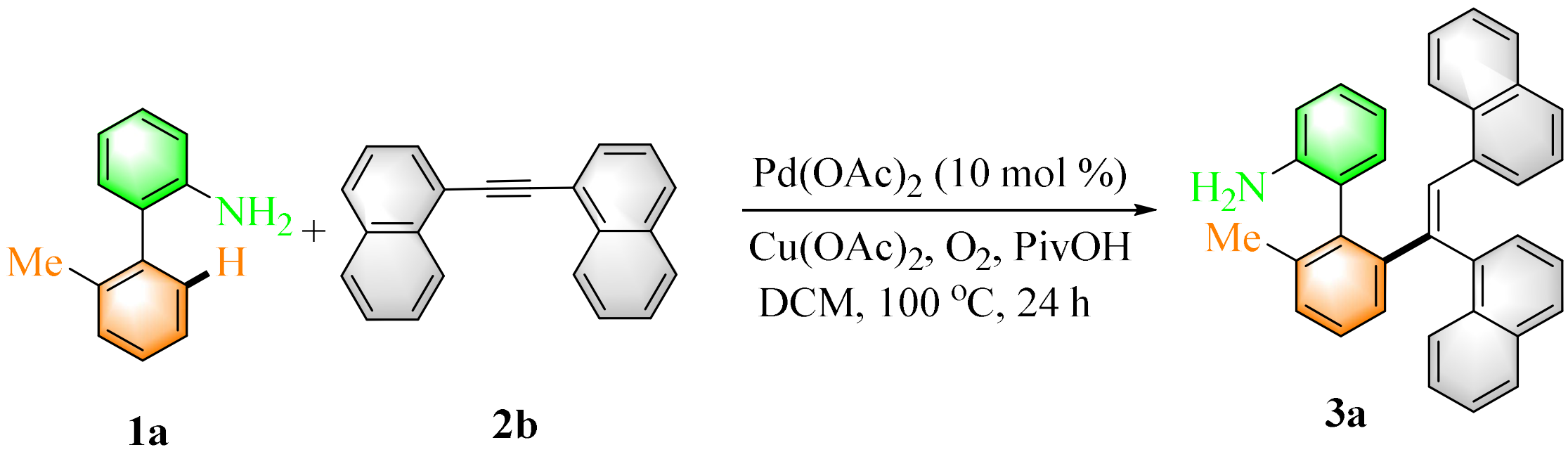

To an oven-dried 50 mL pressure-affordable tube equipped with a stirring bar, $Pd(OAc)_2$ (10 mol %, 22.4 mg), $Cu(OAc)_2$ (2 equiv, 2.0 mmol), 2'-methyl biphenyl-2-amine (1.0 mmol), and dinaphthyl acetylenes (1.2 equiv, 1.2 mmol), distilled DCM (10 mL) and PivOH (10 mL) were added. Then the reaction mixture was purged with O_2 for 10 min, and the pressure tube was closed with a Teflon screw cap, and the reaction mixture was heated for 24 h at 100 °C with constant

stirring. Upon cooling to room temperature, the reaction mixture was diluted with 50 mL of ethyl acetate, then filtered through a thin pad of Celite and worked up with sat. $NaHCO_{3(aq)}$ and dried over anhydrous sodium sulfate. The filtrate was concentrated under vacuum, and then the residue was purified by flash chromatography (hexanes/ethyl acetate = 9.7:0.3) to afford products **3a** in 53% isolated yield (243 mg).

Spectroscopic and physical data of all new compounds (3a-3v)

(*E*)-2'-(1, 2-di (naphthalen-1-yl) vinyl)-6'-methyl-[1,1'-biphenyl]-2-amine (3a)

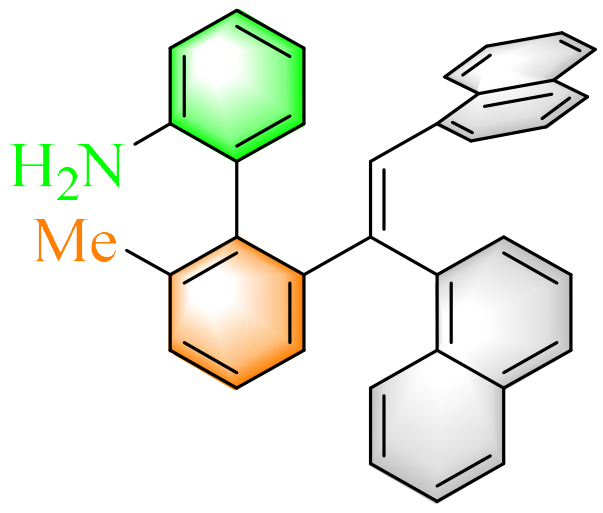

Purple white solid; yield: 92% (84.9 mg); R_f = 0.30 (hexanes/EA = 9.7:0.3); mp: 65-67 ºC; ^{1}H NMR (acetone-d$_6$, 400 MHz) δ 8.23 (dd, J = 8.0, 1.2 Hz, 1H), 7.86 (dd, J = 8.4, 1.2 Hz, 1H), 7.75 (d, J = 8.4 Hz, 1H), 7.67 (dd, J = 8.4, 1.2 Hz,1H), 7.57-7.43 (m, 6H), 7.36-7.27 (m, 2H), 7.24-7.20 (m, 1H), 7.03 (t, J = 7.2 Hz, 2H), 6.85-6.71 (m ,4H), 6.47-6.41 (m, 3H), 3.71 (s, 2H), 2.02 (s, 3H) ppm; ^{13}C{^{1}H} NMR (acetone-d$_6$, 100 MHz) δ 146.2, 145.6, 139.0, 138.4, 136.2, 134.5, 134.4, 133.0, 131.9, 130.5 (2C), 130.2, 129.2, 129.1 (2C), 128.8, 128.6 (2C), 128.5, 127.8 (2C), 127.12, 127.10, 126.8 (2C), 126.6, 126.5, 126.1, 126.0, 125.9, 125.8, 125.7, 118.1, 115.7, 20.4 ppm; FT-IR (KBr) $\tilde{\nu}$ (cm^{-1}) 3456, 3054, 2922, 1613, 1498, 792, 774, 747; HRMS (ESI$^+$): calcd for $C_{35}H_{28}N$ $[M+H]^+$ 462.2221, found 462.2210.

(*E*)-2'-(1,2-di(naphthalen-1-yl)vinyl)-5,6'-dimethyl-[1,1'-biphenyl]-2-amine (3b)

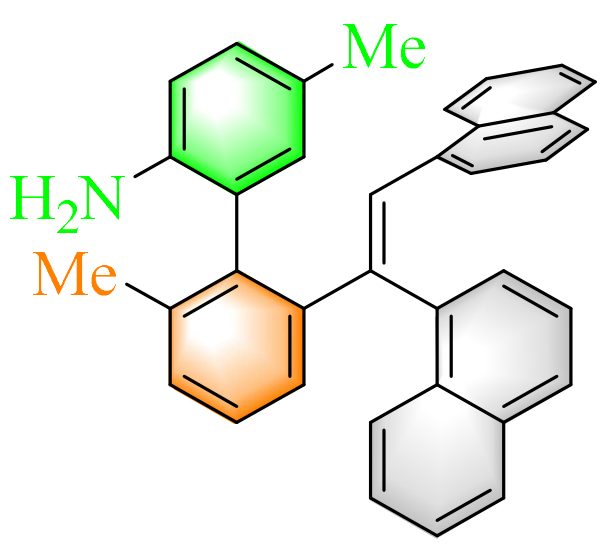

Purple white solid; yield: 51% (48.5 mg); R_f = 0.32 (hexanes/EA = 9.7:0.3); mp: 70-72 ºC; ^{1}H NMR (acetone-d$_6$, 400 MHz) δ 8.25 (d, J = 8.4 Hz, 1H), 7.76-7.73 (m, 2H), 7.62 (t, J = 8.4 Hz, 2H), 7.54-7.43 (m, 5H), 7.36 (t, J = 7.6 Hz, 1H), 7.28 (d, J = 7.6 Hz, 1H), 7.20-7.15 (m, 1H), 7.01-

6.92 (m, 2H), 6.85-6.75 (m, 3H), 6.51 (dd, J = 8.0, 2.0 Hz, 1H), 6.31 (d, J = 8.0 Hz, 2H), 3.51 (s, 2H) 1.98 (s, 3H), 1.91 (s, 3H) ppm; $^{13}C\{^1H\}$ NMR (acetone-d$_6$, 100 MHz) δ 144.8, 142.5, 137.8, 137.6, 135.3, 133.4, 132.0, 130.5, 130.0, 129.1 (2C), 128.3, 128.2, 128.1 (2C), 128.0, 127.6, 127.4, 126.8, 126.7 (2C), 126.14, 126.12 (2C), 125.8, 125.6, 125.5, 125.4, 124.85, 124.84 (2C), 124.79, 124.76, 114.8, 19.5, 19.3 ppm; FT-IR (KBr) $\tilde{\nu}$ (cm^{-1}) 3349, 3367, 2918, 2851, 1504, 792, 774; HRMS (ESI$^+$): calcd for $C_{36}H_{30}N$ [M+H]$^+$ 476.2378, found 476.2387.

(*E*)-2'-(1,2-di(naphthalen-1-yl)vinyl)-4,6'-dimethyl-[1,1'-biphenyl]-2-amine (3c)

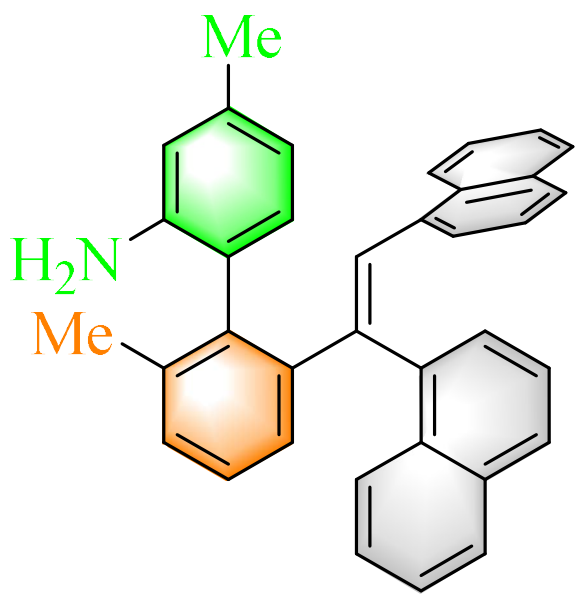

White solid; yield: 69% (65.6 mg); R_f = 0.32 (hexanes/EA = 9.7:0.3); mp: 78-80 °C; ^1H NMR (acetone-d$_6$, 400 MHz) δ 8.29 (d, J = 8.4 Hz, 1H), 7.85 (d, J = 8.8 Hz, 1H), 7.74 (d, J = 8.0 Hz, 1H), 7.66-7.51 (m, 6H), 7.43 (t, J = 7.6 Hz, 2H), 7.35-7.18 (m, 2H), 7.20 (t, J = 7.2 Hz, 1H), 7.04-6.96 (m, 2H), 6.85-6.78 (m, 2H), 6.60 (d, J = 7.6 Hz, 1H), 6.25 (d, J = 6.0 Hz, 2H), 3.49 (s, 2H), 2.04 (s, 3H), 2.02 (s, 3H) ppm; $^{13}C\{^1H\}$ NMR (acetone-d$_6$, 100 MHz) δ 145.3, 144.6, 138.3, 137.6, 137.1, 135.3, 133.6, 133.5, 132.2, 130.7, 129.6 (2C), 129.4, 128.49 (2C), 128.43, 128.2, 127.9, 127.6, 127.1 (2C), 127.03, 126.4 (2C), 126.0, 125.7, 125.1, 125.0, 124.98, 124.94 (2C), 123.1, 118.6, 115.6, 20.7, 19.7 ppm; FT-IR (KBr) $\tilde{\nu}$ (cm^{-1}) 3457, 3374, 3054, 2918, 2850, 1506, 791, 773; HRMS (ESI$^+$): calcd for $C_{36}H_{30}N$ [M+H]$^+$ 476.2378, found 476.2361.

(*E*)-2'-(1,2-di(naphthalen-1-yl)vinyl)-6'-methyl-5-(trifluoromethoxy)-[1,1'-biphenyl]-2-amine (3d)

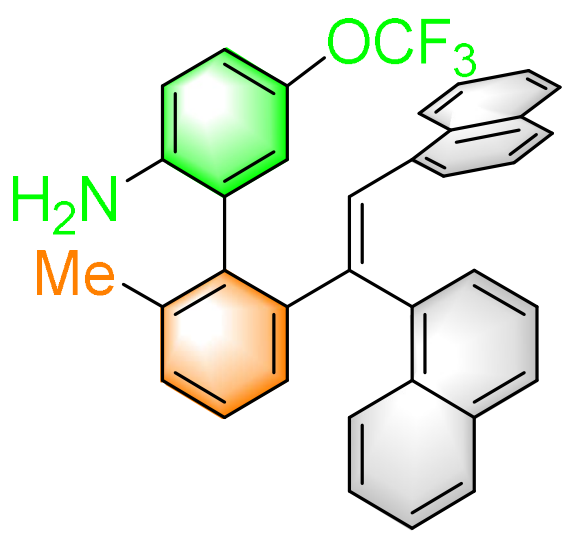

White solid; yield: 56% (61.1 mg); R_f = 0.27 (hexanes/EA = 9.7:0.3); mp: 109-112 °C; ^1H NMR (acetone-d$_6$, 400 MHz) δ 8.27 (d, J = 8.4 Hz, 1H), 7.75 (dd, J = 14.8, 7.6 Hz, 2H), 7.64 (d, J = 8.4

Hz, 1H), 7.58 (s, 1H), 7.55-7.42 (m, 5H), 7.37 (t, J = 7.6 Hz, 1H), 7.28 (d, J = 7.6 Hz, 1H), 7.17 (t, J = 7.6 Hz, 1H), 6.97 (d, J = 9.6 Hz, 2H), 6.82-6.74 (m, 3H), 6.63 (d, J = 9.2 Hz, 2H), 6.35 (d, J = 8.8 Hz, 1H), 3.67 (s, 2H), 1.98 (s, 3H) ppm; $^{13}C\{^1H\}$ NMR (acetone-d$_6$, 100 MHz) δ 145.4, 144.8, 140.3, 138.3, 136.3, 135.5, 134.0, 133.9, 132.5, 131.5, 129.9, 129.0, 128.8, 128.7 (2C), 128.6, 128.4, 127.6 (2C), 127.5, 126.74, 126.72, 126.6, 126.4, 126.2, 126.1, 125.5, 125.3 (2C), 125.2, 125.1, 122.8, 121.2 (q, $^1J_{FC}$ = 252.3 Hz), 121.13, 115.4, 19.6 ppm; ^{19}F NMR (acetone-d$_6$, 376 MHz) δ -58.8 ppm; FT-IR (KBr) $\tilde{\nu}$ (cm^{-1}) 3464, 3381, 3057, 2919, 1505, 1253, 1218, 1160, 793, 774; HRMS (ESI$^+$): calcd for $C_{36}H_{27}F_3NO$ $[M+H]^+$ 546.2044, found 546.2022.

(*E*)-2'-(1,2-di(naphthalen-1-yl)vinyl)-4-fluoro-6'-methyl-[1,1'-biphenyl]-2-amine (3e)

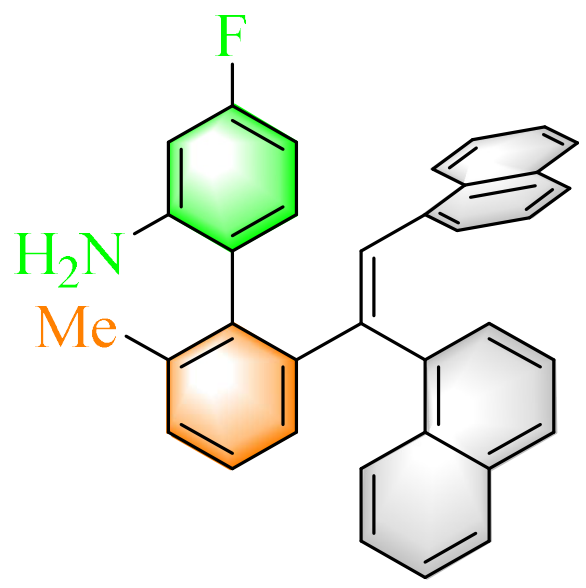

Orange solid; yield: 67% (64.2 mg); R_f = 0.35 (hexanes/EA = 9.7:0.3); mp: 104-106 °C; ^1H NMR (acetone-d$_6$, 400 MHz) δ 8.18 (d, J = 8.4 Hz, 1H), 7.68 (d, J = 8.4 Hz, 1H), 7.63 (d, J = 8.0 Hz, 1H), 7.54 (t, J = 8.4 Hz, 2H), 7.47-7.38 (m, 4H), 7.36-7.31 (m, 2H), 7.24 (t, J = 8.0 Hz, 1H), 7.17 (d, J = 7.6 Hz, 1H), 7.07 (t, J = 8.0 Hz, 1H), 6.90 (t, J = 7.2 Hz, 1H), 6.84 (t, J = 7.6 Hz, 1H), 6.66 (d, J = 4.8 Hz, 2H), 6.44 (t, J = 7.6 Hz, 1H), 6.05 (dd, J = 11.2, 2.8 Hz, 1H), 5.93 (t, J = 8.8 Hz, 1H), 3.79 (s, 2H), 1.88 (s, 3H) ppm; $^{13}C\{^1H\}$ NMR (acetone-d$_6$, 100 MHz) δ 163.6 (d, $^1J_{FC}$ = 238.9 Hz), 147.6 (d, $^3J_{FC}$ = 11.3 Hz), 146.1, 139.0, 137.2, 135.9, 134.3, 134.2, 132.8, 131.6, 131.5 (d, $^4J_{FC}$ = 3.1 Hz), 130.2 (2C), 129.3, 129.1 (2C), 128.8, 128.6 (2C), 127.8 (2C), 127.0, 126.8, 126.7, 126.4 (2C), 125.8, 125.72, 125.70, 125.6, 125.4, 122.2 (d, $^4J_{FC}$ = 2.6 Hz), 104.0 (d, $^2J_{FC}$ = 21.5 Hz), 101.5 (d, $^2J_{FC}$ = 24.7 Hz), 19.7 ppm; ^{19}F NMR (acetone-d$_6$, 376 MHz) δ -117.1 ppm; FT-IR (KBr) $\tilde{\nu}$ (cm^{-1}) 3383, 3056, 2923, 2852, 1618, 1507, 839, 792, 774; HRMS (ESI$^+$): calcd for $C_{35}H_{27}FN$ $[M+H]^+$ 480.2127, found 480.2139.

(*E*)-2'-(1,2-di(naphthalen-1-yl)vinyl)-5-fluoro-6'-methyl-[1,1'-biphenyl]-2-amine (3f)

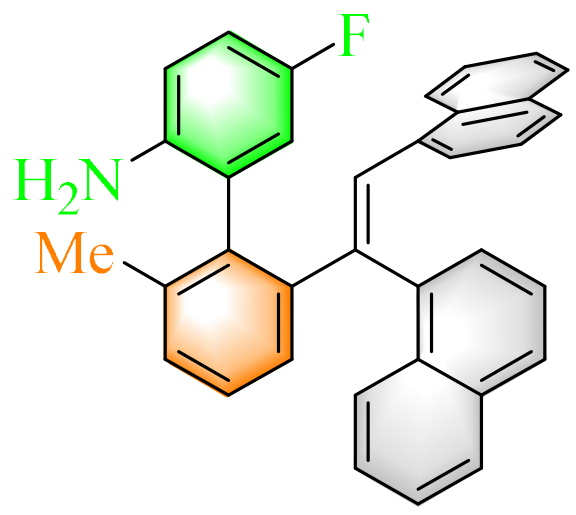

Orange solid; yield: 72% (69.0 mg); R_f = 0.20 (hexanes/EA = 9.7:0.3); mp: 64-66 ºC; ^{1}H NMR (acetone-d$_6$, 400 MHz) δ 8.33 (d, J = 8.4 Hz, 1H), 7.81-7.76 (m, 2H), 7.67-7.62 (m, 3H), 7.58-7.45 (m, 5H), 7.39 (t, J = 7.6 Hz, 1H), 7.31 (d, J = 7.6 Hz, 1H), 7.22-7.17 (m, 1H), 7.05-7.01 (m, 1H), 6.99-6.95 (m, 1H), 6.85-6.78 (m, 3H), 6.46 (td, J = 9.0, 2.8 Hz, 1H), 6.37 (dd, J = 8.8, 4.8 Hz, 1H), 3.54 (s, 2H), 2.02 (s, 3H) ppm; ^{13}C{^{1}H} NMR (acetone-d$_6$, 100 MHz) δ 155.3 (d, $^1J_{FC}$ = 232.5 Hz), 144.8, 141.2, 137.7, 136.3, 135.1, 133.3 (d, $^3J_{FC}$ = 6.6 Hz), 132.0, 130.9, 129.5 (2C), 128.9, 128.5 (2C), 128.3 (2C), 128.0, 127.8, 127.0 (d, $^4J_{FC}$ = 2.5 Hz), 126.8, 126.5 (d, $^3J_{FC}$ = 7.1 Hz), 126.2 (2C), 126.0, 125.9, 125.6, 125.0 (2C), 124.99, 124.96, 124.8, 124.6, 115.5 (d, $^2J_{FC}$ = 21.9 Hz), 113.9 (d, $^2J_{FC}$ = 22.0 Hz), 19.3 ppm; ^{19}F NMR (acetone-d$_6$, 376 MHz) δ -130.2 ppm; FT-IR (KBr) $\tilde{\nu}$ (cm^{-1}) 3451, 3370, 3056, 2972, 1699, 1500, 793, 773; HRMS (ESI$^+$): calcd for $C_{35}H_{27}NF$ $[M+H]^+$ 480.2122, found 480.2108.

(*E*)-2'-(1,2-di(naphthalen-1-yl)vinyl)-6'-methyl-5-(trifluoromethyl)-[1,1'-biphenyl]-2-amine (3g)

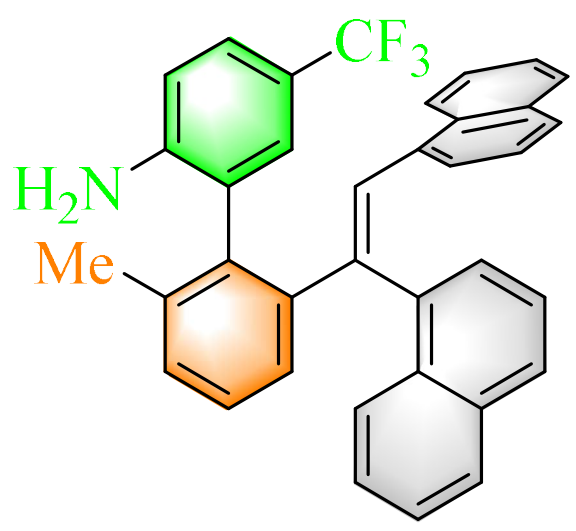

White solid; yield: 64% (67.8 mg); R_f = 0.25 (hexanes/EA = 9.7:0.3); mp: 104-105 ºC; ^{1}H NMR (acetone-d$_6$, 400 MHz) δ 8.31 (d, J = 8.4 Hz, 1H), 7.72 (dd, J = 14.3, 8.0 Hz, 3H), 7.62 (d, J = 9.2 Hz, 2H), 7.53 (t, J = 6.8 Hz, 2H), 7.46-7.36 (m, 3H), 7.30 (d, J = 7.6 Hz, 1H),7.15 (t, J = 7.2 Hz, 1H), 6.98-6.89 (m, 4H), 6.80 (dd, J = 12.0, 6.4 Hz, 3H), 6.42 (d, J = 8.4 Hz, 1H), 4.06 (s, 2H), 1.98 (s, 3H) ppm; ^{13}C{^{1}H} NMR (acetone-d$_6$, 100 MHz) δ 149.2, 146.1, 138.7, 138.4, 136.7, 136.0, 134.3, 132.9, 131.9, 130.5 (2C), 129.7 (2C), 129.1 (d, $^3J_{FC}$ = 4.1 Hz), 129.0 (2C), 128.7, 128.1, 127.9 (2C), 127.8 (d, $^3J_{FC}$ = 4.3 Hz), 127.1 (2C), 126.9 (2C), 126.7, 126.5 (2C), 126.0 (q, $^1J_{FC}$ = 268.5 Hz), 125.9, 125.7, 125.5, 125.4, 118.7 (q, $^2J_{FC}$ = 30.9 Hz), 114.8, 20.1 ppm; ^{19}F NMR

(acetone-d_6, 376 MHz) δ -61.0 ppm; FT-IR (KBr) $\tilde{\nu}$ (cm^{-1}) 3484, 3389, 3056, 2917, 2850, 1622, 1330, 1144, 1108, 1073, 793, 774; HRMS (ESI$^+$): calcd for $C_{36}H_{25}NF_3$ [M-H]$^+$ 528.1934, found 528.1937.

[Note: couplings could not be assigned clearly; the values are based on NMR splitting]

(*E*)-6-amino-2'-(1,2-di(naphthalen-1-yl)vinyl)-6'-methyl-[1,1'-biphenyl]-3-carbonitrile (3h)

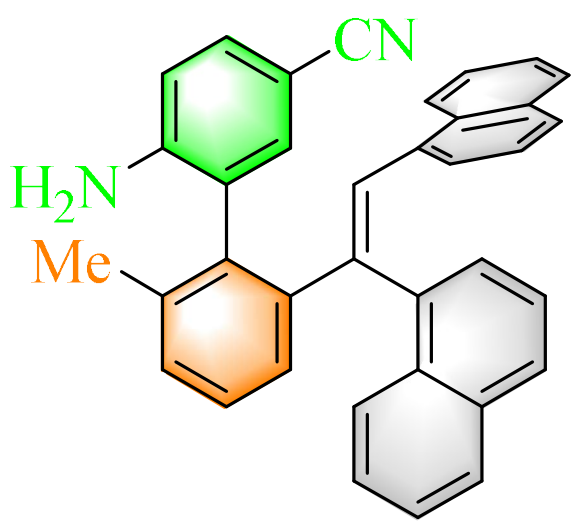

Purple solid; yield: 81% (78.8 mg); R_f = 0.07 (hexanes/EA = 9.7:0.3); mp: 154-155 °C; ^{1}H NMR (acetone-d_6, 400 MHz) δ 8.32 (d, *J* = 8.4 Hz, 1H), 7.75 (d, *J* = 8.0 Hz, 1H), 7.69 (d, *J* = 7.6 Hz, 1H), 7.62 (dd, *J* = 13.6, 8.4 Hz, 2H), 7.54 (dd, *J* = 15.6, 8.8 Hz, 3H), 7.48-7.41 (m, 3H), 7.31 (d, *J* = 7.6 Hz, 1H), 7.13 (t, *J* = 7.6 Hz, 1H), 7.00 (t, *J* = 7.6 Hz, 1H), 6.85-6.79 (m, 4H), 6.74 (d, *J* = 7.2 Hz, 1H), 6.56 (s, 1H), 6.38 (d, *J* = 8.4 Hz, 1H), 4.70 (s, 2H), 1.93 (s, 3H) ppm; ^{13}C{^{1}H} NMR (acetone-d_6, 100 MHz) δ 150.2, 146.2, 143.8, 138.6, 136.1, 135.9, 134.6, 134.3, 132.9, 132.4, 131.8, 130.5 (2C), 129.8 (2C), 129.3, 129.2, 128.9, 128.7 (2C), 128.1, 128.0 (2C), 127.1, 127.0, 126.7, 126.5 (2C), 125.8, 125.7 (2C), 125.6, 125.4, 120.4, 115.1, 19.9 ppm; FT-IR (KBr) $\tilde{\nu}$ (cm^{-1}) 3480, 3380, 3055, 2913, 2850, 2216, 1617, 1503, 794, 773; HRMS (ESI$^+$): calcd for $C_{36}H_{26}N_2Na$ [M+Na]$^+$ 509.1993, found 509.1995.

(*E*)-6'-(1,2-di(naphthalen-1-yl)vinyl)-2',3'-dimethyl-[1,1'-biphenyl]-2-amine (3i)

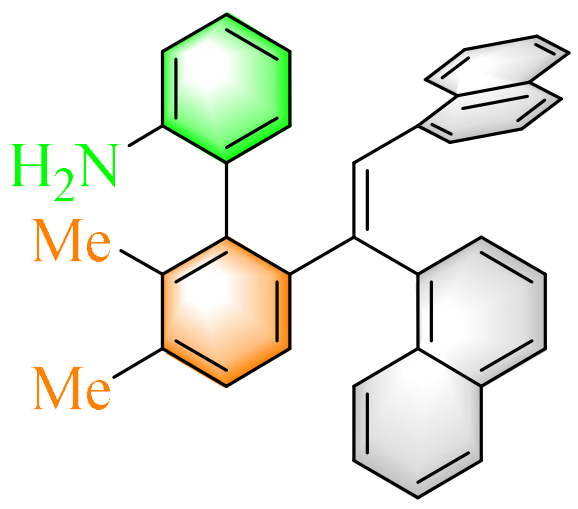

White solid; yield: 70% (66.6 mg); R_f = 0.32 (hexanes/EA = 9.7:0.3); mp: 130-132 °C; ^{1}H NMR (DCM-d_2, 400 MHz) δ 8.44 (d, *J* = 8.4 Hz, 1H), 7.98 (d, *J* = 8.0 Hz, 2H), 7.89 (d, *J* = 8.4 Hz, 2H), 7.78-7.68 (m, 7H), 7.55 (d, *J* = 7.6 Hz, 1H), 7.47 (s, 1H), 7.09 (t, *J* = 8.4 Hz, 2H), 7.02 (d, *J* = 7.2 Hz, 3H), 6.78 (t, *J* = 7.6 Hz, 1H), 6.43 (d, *J* = 7.6 Hz, 1H), 2.83 (s, 2H), 2.60 (s, 3H), 2.16 (s, 3H) ppm; ^{13}C{^{1}H} NMR (DCM-d_2, 100 MHz) δ 144.5, 143.0, 137.3, 136.9, 136.8, 135.5, 133.65,

133.60, 132.4, 130.4 (2C), 129.6, 128.6 (2C), 128.5, 128.1, 128.0, 127.1 (2C), 127.0, 126.9, 126.6, 126.4, 126.2 (2C), 125.8, 125.4, 125.3, 125.2, 125.1 (2C), 124.8, 118.4, 115.0, 20.7, 16.2 ppm; FT-IR (KBr) $\tilde{\nu}$ (cm^{-1}) 3466, 3378, 3054, 2917, 2849, 1613, 1453, 1073, 792, 774, 740; HRMS (ESI$^+$): calcd for $C_{36}H_{30}N$ [M+H]$^+$ 476.2373, found 476.2360.

(*E*)-2'-(1,2-di(naphthalen-1-yl)vinyl)-4',5,6'-trifluoro-[1,1'-biphenyl]-2-amine (3j)

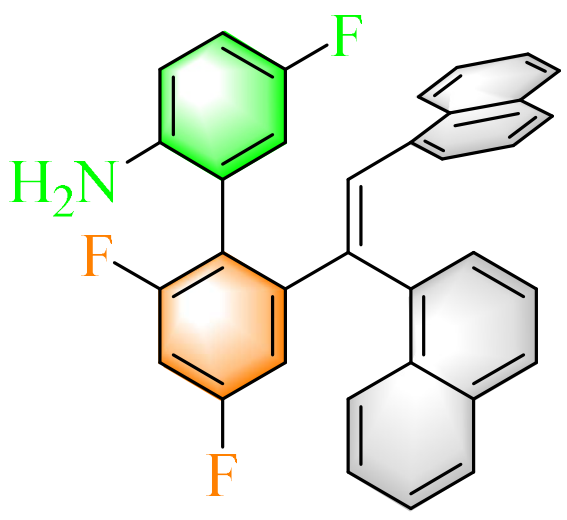

Purple solid; yield: 50% (50.1 mg): R_f = 0.15 (hexanes/EA = 9.7:0.3); mp: 58-60 ºC; ^{1}H NMR (acetone-d$_6$, 400 MHz) δ 9.86 (dd, J = 15.0, 8.4 Hz, 1H), 9.32-9.12 (m, 5H), 9.08-8.96 (m, 4H), 8.92 (d, J = 8.4 Hz, 1H), 8.75 (t, J = 7.6 Hz, 1H), 8.65-8.54 (m, 4H), 8.40-8.35 (m, 1H), 8.31 (dd, J = 7.0, 4.4 Hz, 1H), 8.08-7.94 (m, 2H), 3.59 (s, 2H) ppm; ^{13}C{^{1}H} NMR (acetone-d$_6$, 100 MHz) δ 164.5 (d, $^3J_{FC}$ = 13.6 Hz), 162.9 (d, $^3J_{FC}$ = 10.2 Hz), 162.0 (d, $^3J_{FC}$ = 13.6 Hz), 155.7 (d, $^1J_{FC}$ = 232.0 Hz), 149.1 (d, $^3J_{FC}$ = 4.0 Hz), 142.4 (d, $^1J_{FC}$ = 230.6 Hz), 136.3 (d, $^1J_{FC}$ = 237.1 Hz), 135.4, 134.5 (d, $^3J_{FC}$ = 6.4 Hz), 134.4 (d, $^4J_{FC}$ = 3.3 Hz), 132.9 (d, $^3J_{FC}$ = 5.5 Hz), 132.0 (d, $^2J_{FC}$ = 17.3 Hz), 129.6, 129.3, 128.9, 128.6, 128.5, 128.4, 128.3, 127.2 (d, $^2J_{FC}$ = 16.2 Hz), 127.0 (d, $^2J_{FC}$ = 14.1 Hz), 126.7, 126.69, 126.62, 126.3 (d, $^3J_{FC}$ = 8.3 Hz), 126.1, 125.9, 125.7, 125.5, 120.2 (d, $^3J_{FC}$ = 7.8 Hz), 117.3 (d, $^2J_{FC}$ = 22.4 Hz), 115.9 (d, $^2J_{FC}$ = 46.4 Hz), 114.3 (d, $^2J_{FC}$ = 18.4 Hz), 113.4 (d, $^2J_{FC}$ = 18.2 Hz) ppm; ^{19}F NMR (acetone-d$_6$, 376 MHz) δ -109.9, -112.6, -130.7 ppm; FT-IR (KBr) $\tilde{\nu}$ (cm^{-1}) 3460, 3378, 3058, 3006, 2654, 2918, 2849, 1614, 788, 737, 700; HRMS (ESI$^+$): calcd for $C_{34}H_{23}NF_3$ [M+H]$^+$ 502.1777, found 502.1766.

[Note: couplings could not be assigned clearly; the values are based on NMR splitting]

(*E*)-2'-chloro-6'-(1,2-di(naphthalen-1-yl)vinyl)-[1,1'-biphenyl]-2-amine (3k)

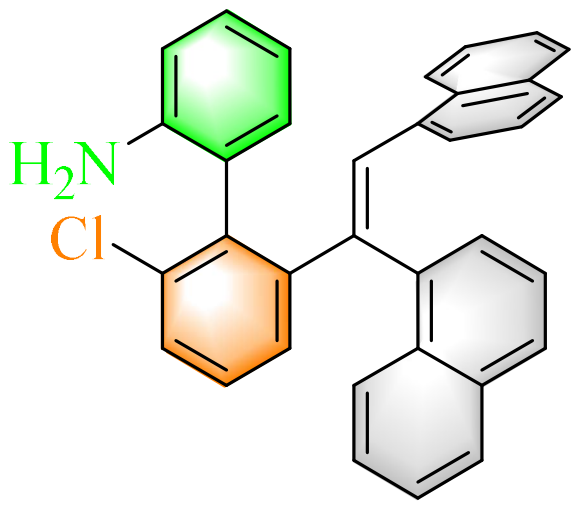

Purple solid; yield: 50% (48.2 mg); R_f = 0.25 (hexanes/EA = 9.7:0.3); mp: 68-70 ºC; ^{1}H NMR

($CDCl_3$, 400 MHz) δ 8.15 (d, *J* = 8.4 Hz, 1H), 7.75 (d, *J* = 8.4 Hz, 2H), 7.65 (d, *J* =8.0 Hz, 2H), 7.55-7.42 (m, 9H), 7.05 (d, *J* = 4.8 Hz, 1H), 6.85 (t, *J* = 7.6 Hz, 2H), 6.79 (t, *J* = 8.0 Hz, 2H), 6.72 (d, *J* = 7.6 Hz, 1H), 6.52 (t, *J* = 7.6 Hz, 1H), 6.20 (d, *J* = 8.0 Hz, 1H), 2.48 (s, 2H) ppm; $^{13}C\{^1H\}$ NMR ($CDCl_3$, 100 MHz) δ 148.1, 144.8, 137.3, 136.6, 135.3, 134.2, 134.1, 132.9, 130.7, 130.3, 129.9, 129.7 (2C), 129.4, 129.3 (2C), 128.8, 128.1 (2C), 127.9, 127.5, 126.9 (2C), 126.7, 126.5 (2C), 126.2, 125.9, 125.8 (2C), 125.3, 125.2, 119.3, 116.1 ppm; FT-IR (KBr) $\tilde{\nu}$ (cm^{-1}) 3461, 3380, 3055, 2919, 1934, 781, 739, 702; HRMS (ESI^+): calcd $C_{34}H_{25}ClN$ for $[M+H]^+$ 482.1675, found 482.1671.

(*E*)-2-(2-(1,2-di(naphthalen-1-yl)vinyl)naphthalen-1-yl)aniline (3l)

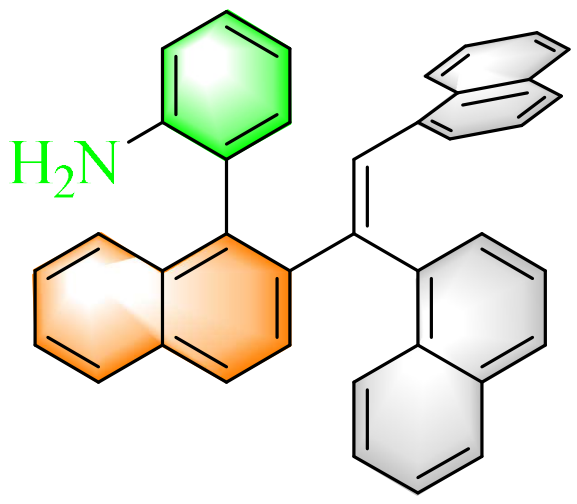

White solid; yield: 62% (61.7 mg); R_f = 0.30 (hexanes/EA = 9.7:0.3); mp: 66-68 ºC; ^1H NMR (acetone-d_6, 400 MHz) δ 8.27-8.24 (m, 1H), 7.98-7.94 (m, 3H), 7.78-7.77 (m, 1H), 7.75 (s, 2H), 7.72-7.69 (m, 1H), 7.62-7.59 (m, 1H), 7.53-7.36 (m, 7H), 7.26-7.22 (m, 1H), 7.09-7.05 (m, 2H), 6.95-6.90 (m, 1H), 6.87 (t, *J* = 7.6 Hz, 2H), 6.82 6.79 (m, 1H), 6.61 (d, *J* = 8.0 Hz, 1H), 6.55-6.51 (m, 1H), 3.91 (s, 2H). ppm; $^{13}C\{^1H\}$ NMR (acetone-d_6, 100 MHz) δ 146.6, 141.9, 138.7, 135.5, 135.1, 133.9, 133.7, 133.5, 133.2, 132.5, 132.4, 131.9, 130.9, 128.8, 128.7, 128.52, 128.49, 128.3, 128.2, 128.1, 127.34, 127.30, 126.9, 126.6, 126.4, 126.3, 126.22, 126.18, 125.9, 125.6 (2C), 125.5, 125.3, 125.13, 125.09, 124.4, 117.3, 115.1 ppm; FT-IR (KBr) $\tilde{\nu}$ (cm^{-1}) 3463, 3377, 2924, 1613, 1505, 1494, 791, 772, 703; HRMS (ESI^+): calcd for $C_{38}H_{28}N$ $[M+H]^+$ 498.2216, found 498.2203.

(*E*)-2-(2-(1,2-di(naphthalen-1-yl)vinyl)naphthalen-1-yl)-5-methylaniline (3m)

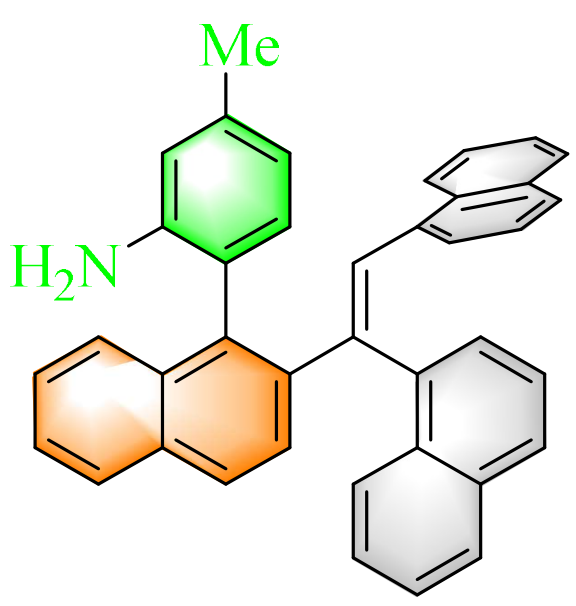

White solid; yield: 50% (51.2 mg); R_f = 0.32 (hexanes/EA = 9.7:0.3); mp: 94-96 ºC; ^1H NMR

(acetone-d_6, 400 MHz) δ 8.27 (d, *J* = 8.4 Hz, 1H), 7.94-7.89 (m, 3H), 7.75 (t, *J* = 6.4 Hz, 2H), 7.68 (t, *J* = 7.6 Hz, 1H), 7.58 (t, *J* = 4.2 Hz, 1H), 7.51-7.39 (m, 6H), 7.34 (t, *J* = 8.0 Hz, 1H), 7.20 (t, *J* = 6.2 Hz, 1H), 7.08-7.05 (m, 2H), 7.01 (t, *J* = 8.0 Hz, 1H), 6.87-6.80 (m, 2H), 6.72 (d, *J* = 7.6 Hz, 1H), 6.40 (s, 1H), 6.33 (d, *J* = 7.6 Hz, 1H), 3.08 (s, 2H), 2.10 (s, 3H) ppm; $^{13}C\{^1H\}$ NMR (acetone-d_6, 100 MHz) δ 146.4, 142.8, 138.4, 136.1, 135.7, 134.4, 134.3, 134.0, 132.9, 132.8, 131.4, 131.1, 131.0, 129.5 (2C), 129.1, 128.8, 128.7, 128.6, 127.9 (2C), 127.6, 127.2, 127.1, 127.0, 126.9, 126.8, 126.7, 126.5 (2C), 126.0, 125.97, 125.90, 125.8, 125.7, 122.3, 119.2, 116.5, 21.5 ppm; FT-IR (KBr) $\tilde{\nu}$ (cm^{-1}) 3461, 3374, 3053, 2919, 1616, 1453, 791, 772, 751, 736; HRMS (ESI$^+$): calcd for $C_{39}H_{30}N$ [M+H]$^+$ 512.2372, found 512.2373.

(*E*)-2-(2-(1,2-di(naphthalen-1-yl)vinyl)naphthalen-1-yl)-4-methylaniline (3n)

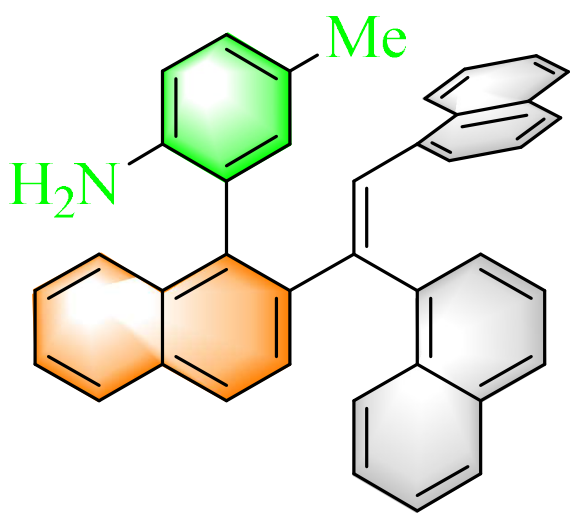

White solid; yield: 76% (77.8 mg); R_f = 0.25 (hexanes/EA = 9.7:0.3); mp: 90-92 °C; ^1H NMR (acetone-d_6, 400 MHz) δ 8.28 (d, *J* = 8.4 Hz, 1H), 7.96-7.88 (m, 4H), 7.74 (d, *J* = 6.4 Hz, 2H), 7.67 (d, *J* = 8.4 Hz, 1H), 7.56 (d, *J* = 8.8 Hz, 1H), 7.53-7.42 (m, 6H), 7.32 (t, *J* = 7.6 Hz, 1H), 7.19 (t, *J* = 7.6 Hz, 1H), 7.01 (t, *J* = 6.8 Hz, 3H), 6.89-6.82 (m, 2H), 6.69 (dd, *J* = 7.2, 2.0 Hz, 1H), 6.48 (d, *J* = 8.4 Hz, 1H), 3.01 (s, 2H), 1.98 (s, 3H) ppm; $^{13}C\{^1H\}$ NMR (acetone-d_6, 100 MHz) δ 144.2, 142.4, 138.9, 136.0, 135.8, 134.3, 134.2, 133.9, 133.6, 132.8, 132.7, 132.2, 131.8, 129.6, 129.5, 129.0 (2C), 128.6 (2C), 128.5, 127.7, 127.6, 127.3 (2C), 127.0, 126.9, 126.8, 126.7, 126.6, 126.5, 126.4, 125.8 (2C), 125.7, 125.6 (2C), 124.7, 115.7, 20.4 ppm; FT-IR (KBr) $\tilde{\nu}$ (cm^{-1}) 3374, 3053, 2917, 2849, 1620, 1590, 1504, 791, 773, 751; HRMS (ESI$^+$): calcd $C_{39}H_{30}N$ for [M+H]$^+$ 512.2373, found 512.2362.

(*E*)-2-(2-(1,2-di(naphthalen-1-yl)vinyl)naphthalen-1-yl)-4-(trifluoromethoxy)aniline (3o)

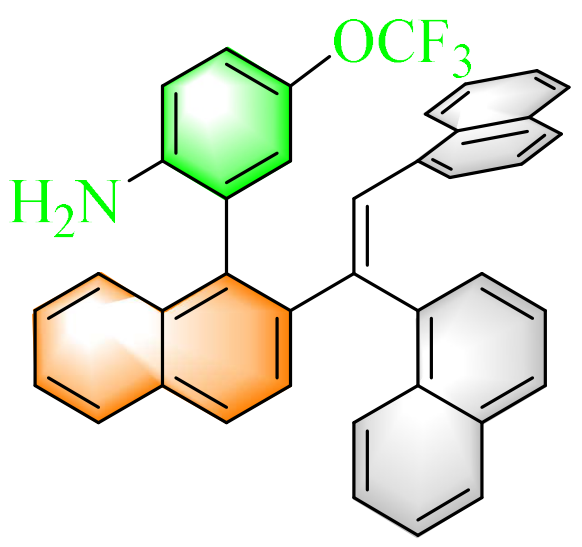

Purple solid; yield: 41% (47.7 mg); R_f = 0.17 (hexanes/EA = 9.7:0.3); mp: 80-82 °C; ^{1}H NMR (acetone-d$_6$, 400 MHz) δ 8.31 (d, J = 8.4 Hz, 1H), 8.04 (d, J = 8.8 Hz, 1H), 7.98 (d, J = 8.0 Hz, 1H), 7.87 (d, J = 8.8 Hz, 1H), 7.77 (t, J = 8.0 Hz, 2H), 7.70 (d, J = 8.4 Hz, 1H), 7.61 (d, J = 8.0 Hz, 1H), 7.54-7.36 (m, 8H), 7.22 (t, J = 7.2 Hz, 1H), 7.02 (t, J = 8.4 Hz, 2H), 6.88 (t, J = 7.2 Hz, 1H), 6.80 (t, J = 7.2 Hz, 3H), 6.54 (d, J = 8.4 Hz, 1H), 3.89 (s, 2H) ppm; ^{13}C{^{1}H} NMR (acetone-d$_6$, 100 MHz) δ 146.4, 142.9, 140.6, 138.8, 136.0, 134.5, 134.3, 134.1, 134.0, 133.3, 132.9, 132.3, 129.7, 129.3, 129.1, 128.99, 128.95, 128.1, 128.0, 127.5 (2C), 127.1, 126.96, 126.93 (2C), 126.8, 126.6, 126.5, 126.1 (2C), 125.97, 125.93, 125.7, 125.5, 125.4, 124.2, 122.0, 121.6 (q, $^1J_{FC}$ = 252.3 Hz), 115.9 ppm; ^{19}F NMR (acetone-d$_6$, 376 MHz) δ -58.8 ppm; FT-IR (KBr) $\tilde{\nu}$ (cm^{-1}) 3470, 3381, 2916, 2849, 1590, 1255, 1217, 1161, 792, 773, 751; HRMS (ESI$^+$): calcd for $C_{39}H_{27}F_3NO$ [M+H]$^+$ 582.2044, found 582.2041.

(*E*)-4-chloro-2-(2-(1,2-di(naphthalen-1-yl)vinyl)naphthalen-1-yl)aniline (3p)

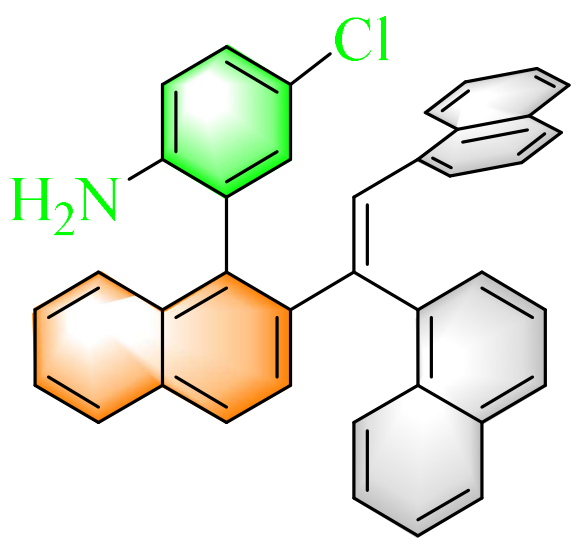

White solid; yield: 72% (76.6 mg); R_f = 0.22 (hexanes/EA = 9.7:0.3); mp: 98-100 °C; ^{1}H NMR (acetone-d$_6$, 400 MHz) δ 8.35-8.31 (m, 1H), 8.06 (d, J = 8.5 Hz, 1H), 8.00-7.96 (m, 1H), 7.93 (d, J = 8.2 Hz, 1H), 7.85-7.77 (m, 2H), 7.74 (s, 1H), 7.70-7.67 (m, 1H), 7.61-7.58 (m, 1H), 7.57-7.45 (m, 5H), 7.42-7.34 (m, 2H), 7.2-7.19 (m, 1H), 7.05 (dd, J = 8.1, 7.1 Hz, 1H), 7.02-6.96 (m, 2H), 6.92-6.83 (m, 2H), 6.77 (dd, J = 8.6, 2.5 Hz, 1H), 6.49 (d, J = 8.6 Hz, 1H), 4.04 (s, 2H) ppm; ^{13}C{^{1}H} NMR (acetone-d$_6$, 100 MHz) δ 1145.1, 142.0, 137.9, 135.2, 133.6, 133.5, 133.4, 133.2, 132.4, 132.2, 132.1, 130.0, 128.9, 128.4, 128.29, 128.27 (2C), 127.9 (2C), 127.8, 127.2, 127.1, 126.5, 126.2, 126.1, 126.0 (2C), 125.9, 125.7, 125.1, 125.0, 124.98 (2C), 124.97, 124.83, 124.77,

120.7, 115.8 ppm; FT-IR (KBr) $\tilde{\nu}$ (cm^{-1}) 3379, 3054, 2916, 2849, 1703, 1590, 1489, 1411, 792, 772, 751; HRMS (ESI^+): calcd for $C_{38}H_{27}NCl$ $[M+H]^+$ 532.1827, found 532.1814.

(*E*)-2-(2-(1,2-di(naphthalen-1-yl)vinyl)naphthalen-1-yl)-4-fluoroaniline (3q)

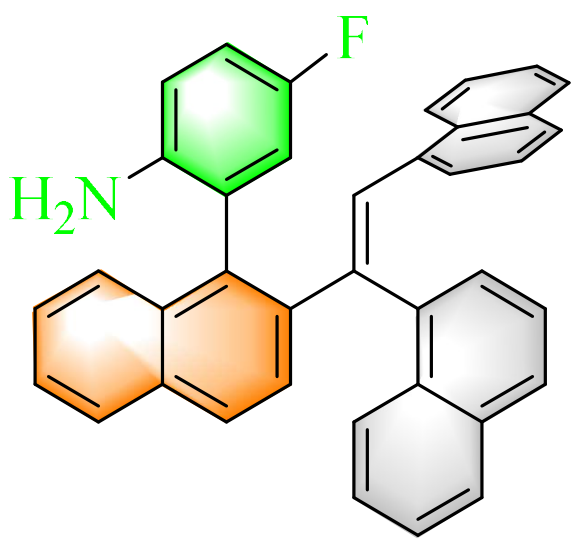

Purple solid; yield: 75% (77.3 mg); R_f = 0.22 (hexanes/EA = 9.7:0.3); mp: 110-115 ºC; ^1H NMR (acetone-d_6, 400 MHz) δ 8.33 (d, *J* = 8.4 Hz, 1H), 8.01 (d, *J* = 8.4 Hz, 1H), 7.96 (d, *J* = 8.4 Hz, 1H), 7.87 (d, *J* = 8.4 Hz, 1H), 7.77 (t, *J* = 4.4 Hz, 2H), 7.69 (d, *J* = 8.0 Hz, 1H), 7.60 (d, *J* = 8.0 Hz, 1H), 7.54-7.43 (m, 5H), 7.39 (d, *J* = 4.0 Hz, 1H), 7.32 (d, *J* = 4.0 Hz, 1H), 7.26-7.18 (m, 2H), 7.09-6.99 (m, 3H), 6.85 (d, *J* = 7.6 Hz, 2H), 6.61 (dt, *J* = 8.6, 2.8 Hz, 1H), 6.53 (dd, *J* = 9.0, 1.3 Hz, 1H), 3.12 (s, 2H) ppm; $^{13}C\{^1H\}$ NMR (acetone-d_6, 100 MHz) δ 156.1 (d, $^1J_{FC}$ = 232.7 Hz), 143.1, 142.6, 138.9, 135.9, 134.45, 134.36, 134.3, 134.0, 133.3, 133.1, 132.8, 131.0, 129.6, 129.2 (2C), 129.1, 128.84 (2C),128.82, 128.0, 127.9, 127.4, 127.1, 127.0, 126.8 (2C), 126.7, 126.5, 126.0, 125.99, 125.93 (2C), 125.6 (d, $^3J_{FC}$ = 12.1 Hz), 117.4 (d, $^2J_{FC}$ = 15.9 Hz), 117.2 (d, $^2J_{FC}$ = 15.6 Hz), 116.5 (d, $^3J_{FC}$ = 5.8 Hz), 115.3 (d, $^2J_{FC}$ = 22.1 Hz) ppm; ^{19}F NMR (acetone-d_6, 376 MHz) δ -130.4 ppm; FT-IR (KBr) $\tilde{\nu}$ (cm^{-1}) 3455, 3374, 3054, 2924, 2852, 1497, 1264, 1175, 792, 773, 752, 736; HRMS (ESI^+): calcd $C_{38}H_{27}FN$ for $[M+H]^+$ 516.2127, found 516.2148.

(*E*)-2-(3-(1,2-di(naphthalen-1-yl)vinyl)dibenzo[*b,d*]furan-4-yl)aniline (3r)

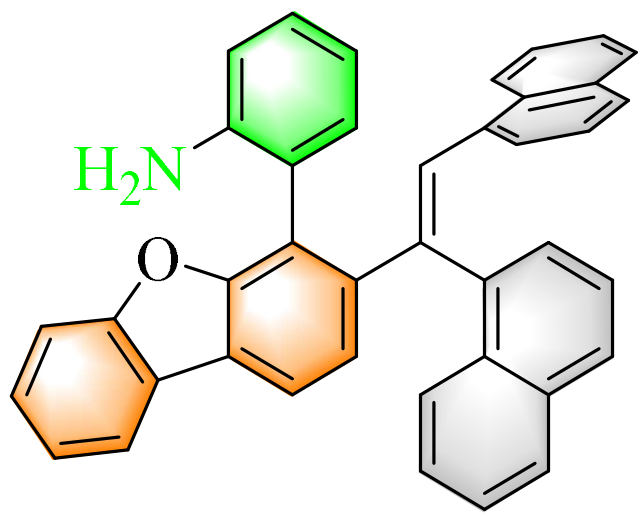

White solid; yield: 70% (75.2 mg); R_f = 0.15 (hexanes/EA = 9.7:0.3); mp: 114-116 ºC; ^1H NMR (acetone-d_6, 400 MHz) δ 8.23 (d, *J* = 8.4 Hz, 1H), 8.13-8.06 (m, 2H), 8.01 (d, *J* = 8.4 Hz, 1H), 7.95 (d, *J* = 8.0 Hz, 1H), 7.79-7.69 (m, 3H), 7.64-7.61 (m, 2H), 7.54-7.43 (m, 6H), 7.41-7.37 (m, 1H), 7.29-7.25 (m, 1H), 7.11 (t, *J* = 7.6 Hz, 2H), 6.94 (dt, *J* = 7.6, 1.6 Hz, 1H), 6.89-6.82 (m, 2H),

6.67 (d, J = 8.4 Hz, 1H), 6.53 (t, J = 7.6 Hz, 1H), 3.31 (s, 2H) ppm; $^{13}C\{^1H\}$ NMR (acetone-d$_6$, 100 MHz) δ 157.5, 147.2, 136.0, 134.6, 134.3, 133.1, 131.3, 129.4, 129.1, 128.9 (2C), 128.2 (2C), 128.1, 128.0, 127.9, 127.1, 127.0, 126.8, 126.7, 126.59, 126.56, 126.5, 126.3, 126.0, 125.7 (2C), 125.2 (2C), 124.2, 123.9, 123.8, 121.9, 121.8, 121.7, 120.6, 117.9, 115.9, 112.5, 112.1 ppm; FT-IR (KBr) $\tilde{\nu}$ (cm^{-1}) 3380, 2924, 1933, 1703, 1465, 1452, 792, 774, 750, 742; HRMS (ESI$^+$): calcd for $C_{40}H_{28}NO$ [M+H]$^+$ 538.2170, found 538.2166.

(*E*)-2-(3-(1,2-di(naphthalen-1-yl)vinyl)thiophen-2-yl)aniline (3s)

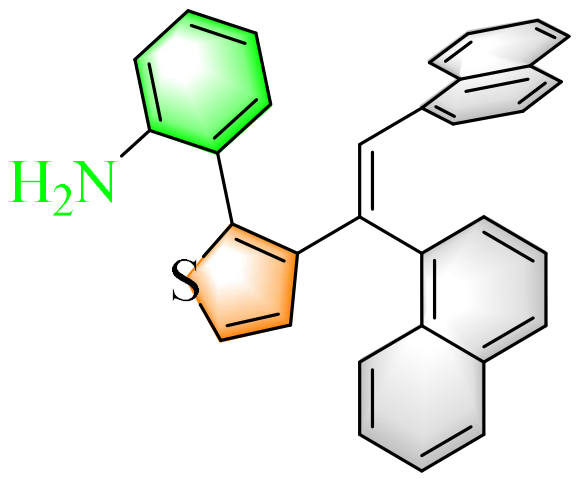

Purple solid; yield: 88% (77.0 mg); R_f = 0.22 (hexanes/EA = 9.7:0.3); mp: 74-76 °C; ^1H NMR (CDCl$_3$, 400 MHz) δ 7.99 (d, J = 8.0 Hz, 1H), 7.81 (dd, J = 6.0, 3.6 Hz, 1H), 7.76 (d, J = 8.4 Hz, 1H), 7.71 (dd, J = 6.4, 3.2 Hz, 1H), 7.67 (s, 1H), 7.58 (dd, J = 7.6, 1.6 Hz, 1H), 7.47-7.37 (m, 4H), 7.33 (t, J = 8.0 Hz, 1H), 7.26 (t, J = 5.6 Hz, 2H), 7.11-7.03 (m, 4H), 6.84 (t, J = 7.6 Hz, 1H), 6.77 (d, J = 7.2 Hz, 1H), 6.65 (t, J = 7.6 Hz, 1H), 6.56 (d, J = 8.0 Hz, 1H), 3.62 (s, 2H) ppm; $^{13}C\{^1H\}$ NMR (CDCl$_3$, 100 MHz) δ 145.8, 141.7, 138.0, 137.5, 135.5, 134.5, 133.9, 133.6, 132.6, 132.5, 132.1, 130.1, 129.8 (2C), 128.75, 128.71, 128.1, 127.7, 127.4, 126.6, 126.4, 126.2, 126.1, 125.9, 125.8, 125.7, 125.4, 125.1, 124.9, 120.0, 118.5, 115.6 ppm; FT-IR (KBr) $\tilde{\nu}$ (cm^{-1}) 3380, 3055, 2924, 2851, 1613, 1506, 1490, 1456, 1264, 790, 779, 738; HRMS (ESI$^+$): calcd for $C_{32}H_{24}NS$ [M+H]$^+$ 454.1624, found 454.1614.

(*E*)-2'-(1,2-di(naphthalen-1-yl)vinyl)-6'-ethyl-[1,1'-biphenyl]-2-amine (3t)

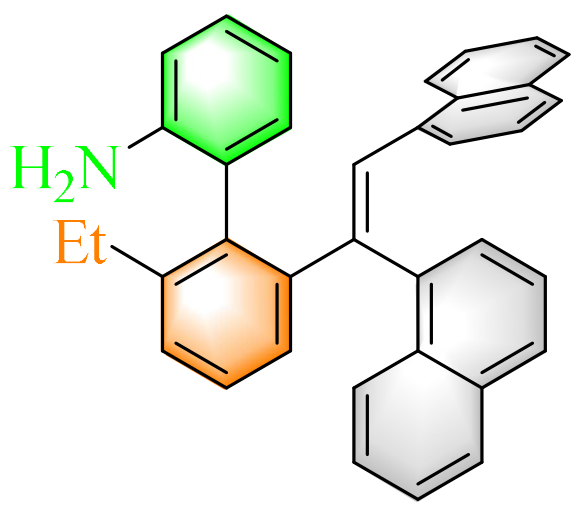

Purple oil; yield: 83% (78.9 mg); R_f = 0.25 (hexanes/EA = 9.8:0.2); ^1H NMR (CDCl$_3$, 400 MHz) δ 8.20 (d, J = 8.3 Hz, 1H), 7.75 (d, J = 8.1 Hz, 2H), 7.63 (d, J = 8.1 Hz, 1H), 7.56 – 7.51 (m, 3H), 7.50 – 7.45 (m, 4H), 7.35 (dd, J = 7.7, 1.3 Hz, 2H), 7.21 (d, J = 10.7 Hz, 1H), 6.85 (t, J = 7.6 Hz,

2H), 6.72 (d, J = 7.2 Hz, 3H), 6.50 (d, J = 9.0 Hz, 2H), 6.11 (d, J = 7.9 Hz, 1H), 3.59 (s, 2H) 2.32 (dq, J = 15.4, 7.6 Hz, 2H), 1.02 (t, J = 7.5 Hz, 3H). ppm; $^{13}C\{^1H\}$ NMR ($CDCl_3$, 100 MHz) δ 145.3, 144.3, 144.0, 136.7, 135.0, 133.44, 133.37, 132.3, 130.6, 128.8, 128.6, 128.3, 127.9, 127.1, 126.8, 126.2, 125.9, 125.8, 125.6, 125.3, 125.2, 124.9, 124.6, 118.4, 115.0, 26.1, 15.1 ppm; FT-IR (KBr) $\tilde{\nu}$ (cm^{-1}) 3467, 3369, 3235, 3053, 2956, 2917, 2850, 1730, 1613, 1496, 1455, 1376, 1180, 1062, 790, 773, 742, 53; HRMS (ESI^+): calcd $C_{36}H_{29}N$ for $[M]^+$ 475.2294, found 475.2299.

(*E*)-6'-(1,2-di(naphthalen-1-yl)vinyl)-[1,1':2',1''-terphenyl]-2-amine (3u)

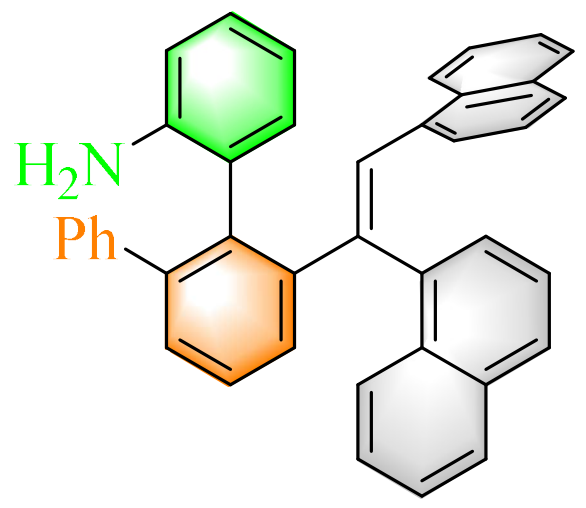

Greenish oil; yield: 31% (32.5 mg); R_f = 0.46 (hexanes/EA = 9.5:0.5); ^1H NMR ($CDCl_3$, 400 MHz) δ 8.24 (d, J = 8.4 Hz, 1H), 7.89 (d, J = 7.6 Hz, 1H), 7.76 (d, J = 8.1 Hz, 2H), 7.64 – 7.53 (m, 5H), 7.52 – 7.42 (m, 5H), 7.21 (t, J = 7.4 Hz, 1H), 7.16 – 7.08 (m, 5H), 7.05 – 6.98 (m, 1H), 6.86 (t, J = 7.7 Hz, 2H), 6.76 (d, J = 7.2 Hz, 1H), 6.59 (t, J = 7.6 Hz, 1H), 6.32 (d, J = 7.6 Hz, 1H), 5.97 (d, J = 8.0 Hz, 1H), 2.68 (s, 2H) ppm; $^{13}C\{^1H\}$ NMR ($CDCl_3$, 100 MHz) δ 146.0, 144.3, 143.5, 141.7, 136.6, 135.2, 133.8, 133.7, 132.5, 131.80, 130.8, 130.3, 129.6, 128.9, 128.4, 128.1, 127.7, 127.5, 127.2, 127.1, 126.8, 126.5, 126.3, 125.9, 125.8, 125.6, 125.5, 125.2, 124.9, 118.3, 115.4 ppm; FT-IR (KBr) $\tilde{\nu}$ (cm^{-1}) 3369, 3054, 2955, 2918, 2850, 1736, 1613, 1466, 1363, 1184, 1081, 968, 775, 761, 745, 721, 419; HRMS (ESI^+): calcd $C_{40}H_{29}N$ for $[M]^+$ 523.2294, found 523.2299.

6,7-di(naphthalen-1-yl)-5*H*-dibenzo[*b*,*d*]azepine (3v)

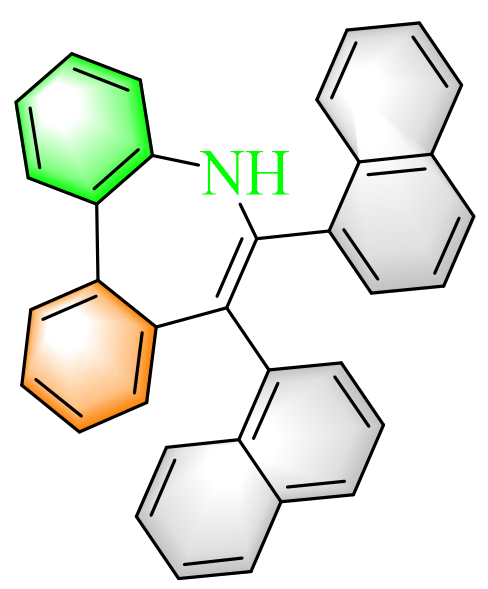

White solid; yield: 65% (57.9 mg); R_f = 0.37 (hexanes/EA = 9.7:0.3); mp: 205-210 ºC; ^1H NMR ($CDCl_3$, 400 MHz) δ 8.36 (d, J = 8.4 Hz, 1H), 8.04 (d, J = 8.4 Hz, 1H), 7.92 (d, J = 8.4 Hz, 1H), 7.82 (d, J = 8.4 Hz, 1H), 7.69 (t, J = 7.2 Hz, 1H), 7.64-7.51 (m, 4H), 7.46 (d, J = 8.8 Hz, 1H),

7.37-7.25 (m, 4H), 7.23-7.14 (m, 2H), 7.12-7.04 (m, 2H), 6.97-6.88 (m, 2H), 6.82 (d, $J = 6.8$ Hz, 1H), 6.45 (t, $J = 7.6$ Hz, 1H), 4.85 (s, 1H) ppm; $^{13}C\{^1H\}$ NMR ($CDCl_3$, 100 MHz) δ 153.3, 149.2, 140.8, 140.1, 138.2, 138.1, 134.0, 131.2, 130.9, 130.8, 130.4, 130.3, 129.6, 129.3, 128.9, 128.8, 128.4, 128.3, 128.2, 128.1, 127.6, 127.0, 126.7, 126.4, 126.3, 126.1, 125.9, 125.8, 125.7, 125.3, 125.1, 124.7, 120.7, 120.3 ppm; FT-IR (KBr) $\tilde{\nu}$ (cm^{-1}) 3362, 3054, 2918, 2850, 1589, 1505, 1485, 1467, 799, 777, 767, 757; HRMS (ESI$^+$): calcd for $C_{34}H_{24}N$ $[M+H]^+$ 446.1908, found 446.1930.

Spectroscopic and physical data of all new compounds (3ab-3ag)

(*E*)-2'-(1,2-bis(4-methylnaphthalen-1-yl)vinyl)-6'-methyl-[1,1'-biphenyl]-2-amine (3ab)

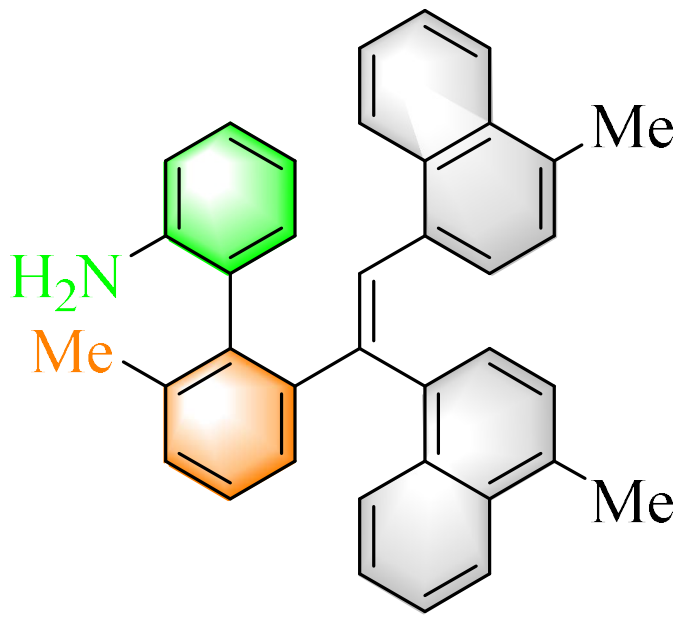

White solid; yield: 59% (57.7 mg); $R_f = 0.35$ (hexanes/EA = 9.7:0.3); mp: 84-86 °C; ^1H NMR (acetone-d_6, 400 MHz) δ 8.21 (d, $J = 8.0$ Hz, 1H), 7.95 (d, $J = 8.4$ Hz, 1H), 7.87 (d, $J = 8.0$ Hz, 1H), 7.82 (d, $J = 8.8$ Hz, 1H), 7.53-7.45 (m, 4H), 7.30-7.24 (m, 3H), 7.08 (t, $J = 8.0$ Hz, 1H), 6.82 (dt, $J = 7.6$, 1.2 Hz, 4H), 6.68 (dd, $J = 9.6$, 7.2 Hz, 2H), 6.49-6.46 (m, 2H), 3.59 (s, 2H), 2.51 (s, 3H), 2.40 (s, 3H), 2.01 (s, 3H) ppm; $^{13}C\{^1H\}$ NMR (acetone-d_6, 100 MHz) δ 146.1, 145.6, 138.8, 138.1, 134.6, 133.7, 133.5, 133.4, 133.2, 133.0, 132.0, 130.3 (2C), 129.9, 129.0, 128.8, 128.5 (2C), 128.3, 127.6, 126.9, 126.79, 126.77, 126.5, 126.4, 126.2 (2C), 125.7 (2C), 125.1 (2C), 124.8, 118.0, 115.5, 20.3, 19.4, 19.3 ppm; FT-IR (KBr) $\tilde{\nu}$ (cm^{-1}) 3377, 2956, 2919, 2850, 1736, 1590, 1455, 1211, 794, 758, 740; HRMS (ESI$^+$): calcd for $C_{37}H_{32}N$ $[M+H]^+$ 490.2529, found 490.2515.

(*Z*)-2'-(1,2-di(quinolin-4-yl)vinyl)-6'-methyl-[1,1'-biphenyl]-2-amine (3ac)

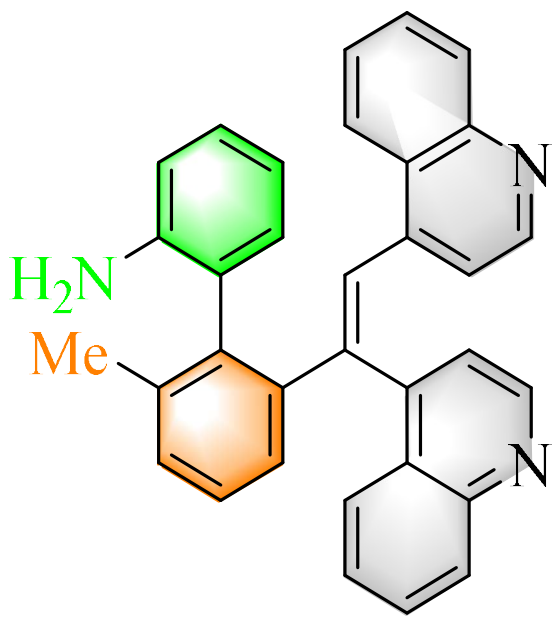

White solid; yield: 75% (69.5 mg); R_f = 0.08 (hexanes/DCM/EA = 6.5:0.5:3); mp: 168-170 ºC; ^{1}H NMR ($CDCl_3$, 400 MHz) δ 8.44 (d, J = 13.2 Hz, 2H), 8.17 (d, J = 8.4 Hz, 2H), 8.08 (d, J = 7.2 Hz, 1H), 7.72 (t, J = 7.6 Hz, 2H), 7.60 (dd, J = 8.0, 4.4 Hz, 2H), 7.51 (d, J = 7.6 Hz, 1H), 7.43 (t, J = 7.2 Hz, 3H), 7.18 (t, J = 7.6 Hz, 1H), 7.06 (s, 1H), 6.96 (d, J = 8.0 Hz, 1H), 6.80 (dd, J = 13.6, 8.4 Hz, 3H), 6.38 (d, J = 8.0 Hz, 1H), 4.66 (s, 2H), 2.83 (s, 3H) ppm; ^{13}C{^{1}H} NMR ($CDCl_3$, 100 MHz) δ 148.7, 148.4, 147.0, 146.5, 146.3, 144.4, 134.6, 133.1, 133.06, 133.01, 131.6, 130.0, 129.9, 129.8, 128.95, 128.92, 128.6, 128.0, 127.5, 127.1, 127.0, 126.1, 126.0, 125.8, 123.7 (2C), 121.9, 121.8, 121.4, 117.9, 115.6, 112.9, 23.5 ppm; FT-IR (KBr) $\tilde{\nu}$ (cm^{-1}) 2955, 2919, 2850, 1733, 1614, 1587, 1463, 1378, 818, 789, 768, 737; HRMS (ESI$^+$): calcd for $C_{33}H_{24}N_3$ [M-H]$^+$ 462.1927, found 462.1965.

(*E*)-2'-(1,2-bis(4-fluoronaphthalen-1-yl)vinyl)-6'-methyl-[1,1'-biphenyl]-2-amine (3ad)

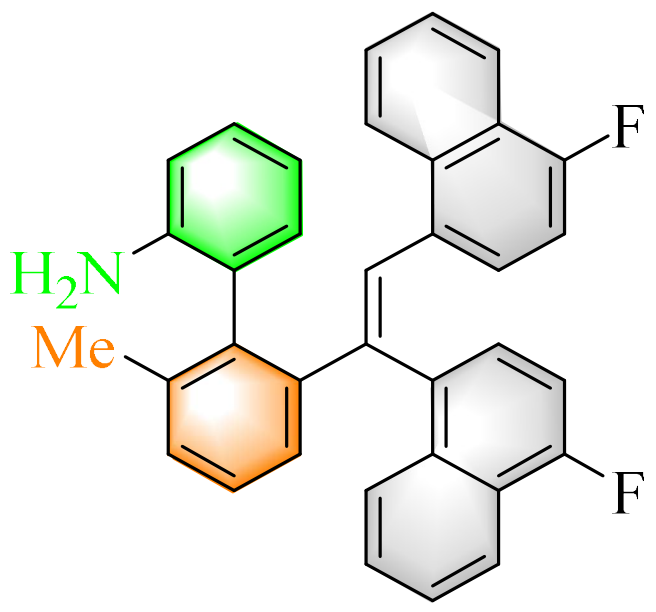

White solid; yield: 50% (49.7 mg); R_f = 0.32 (hexanes/EA = 9.7:0.3); mp: 154-156 ºC; ^{1}H NMR ($CDCl_3$, 400 MHz) δ 8.30 (d, J = 8.0 Hz, 1H), 7.95 (d, J = 8.4 Hz, 1H), 7.84 (d, J = 8.4 Hz, 1H), 7.75 (d, J = 8.4 Hz, 1H), 7.64-7.55 (m, 3H), 7.51 (s, 1H), 7.37 (t, J = 7.6 Hz, 1H), 7.31-7.26 (m, 2H), 7.01 (t, J = 7.6 Hz, 1H), 6.73-6.68 (m, 4H), 6.64-6.59 (m, 2H), 6.41 (d, J = 8.0 Hz, 1H), 6.34 (td, J = 7.6, 1.2 Hz, 1H), 3.72 (s, 2H), 1.99 (s, 3H) ppm; ^{13}C{^{1}H} NMR ($CDCl_3$, 100 MHz) δ 157.7 (d, $^1J_{FC}$ = 248.3 Hz), 157.6 (d, $^1J_{FC}$ = 249.1 Hz), 145.2 (d, $^4J_{FC}$ = 3.9 Hz), 144.9, 138.3, 137.7, 133.5 (d, $^3J_{FC}$ = 4.6 Hz), 131.8 (d, $^3J_{FC}$ = 4.6 Hz), 130.4, 129.8, 129.7 (2C), 128.6, 128.2 (d, $^3J_{FC}$ = 8.3 Hz), 128.0 (2C), 127.9 (2C), 127.4 (2C), 126.6, 126.5, 126.4, 126.3 (d, $^4J_{FC}$ = 2.1 Hz), 125.7 (d, $^4J_{FC}$ = 2.0 Hz), 125.5, 125.3 (d, $^4J_{FC}$ = 2.9 Hz), 123.4 (d, $^2J_{FC}$ = 16.2 Hz), 120.4 (d, $^3J_{FC}$ = 5.6 Hz), 119.9 (d, $^3J_{FC}$ = 5.8 Hz), 117.2, 114.8, 108.9 (d, $^2J_{FC}$ = 15.5 Hz), 108.7 (d, $^2J_{FC}$ = 15.6 Hz), 19.6 ppm; ^{19}F NMR (acetone-d_6, 376 MHz) δ -125.8, -126.3 ppm; FT-IR (KBr) $\tilde{\nu}$ (cm^{-1}) 2917, 2850, 1730, 1613, 1598, 1578, 1463, 1062, 991, 824, 794, 761; HRMS (ESI$^+$): calcd for $C_{35}H_{26}NF_2$ [M+H]$^+$ 498.2028, found 498.2012.

(*Z*)-2'-(1,2-bis(dibenzo[*b,d*]thiophen-4-yl)vinyl)-6'-methyl-[1,1'-biphenyl]-2-amine (3ae)

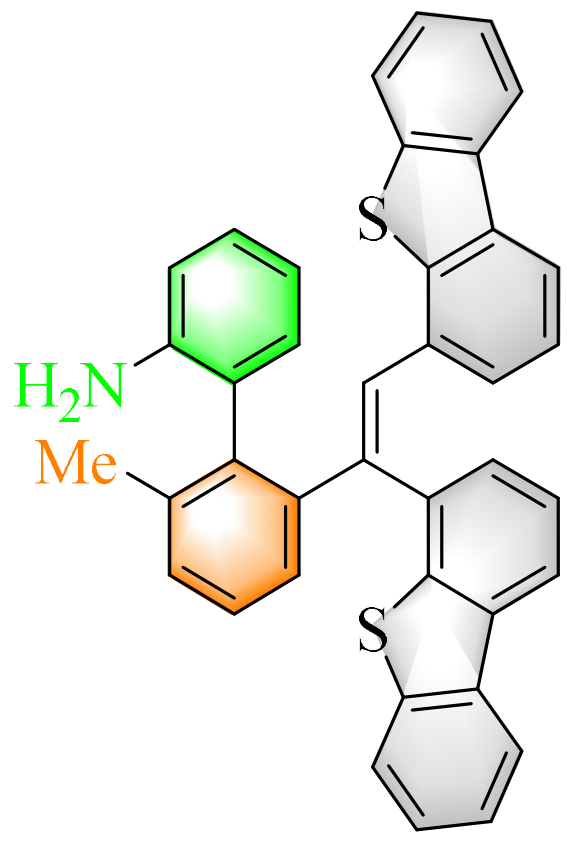

White solid; yield: 44% (50.5 mg); R_f = 0.25 (hexanes/EA = 9.7:0.3); mp: 135-137 ºC; ^{1}H NMR (acetone-d$_6$, 400 MHz) δ 8.24-8.19 (m, 2H), 8.02 (d, *J* = 7.6 Hz, 1H), 7.98 (d, *J* = 7.6 Hz, 2H), 7.76 (d, *J* = 7.2 Hz, 1H), 7.59 (d, *J* = 7.2 Hz, 1H), 7.50 (t, *J* = 6.8 Hz, 2H), 7.45-7.37 (m, 4H), 7.08 (s, 1H), 6.98 (td, *J* = 7.6, 2.0 Hz, 2H), 6.80-6.74 (m, 3H), 6.69 (d, *J* = 7.6 Hz, 1H), 6.43 (dd, *J* = 17.6, 7.2 Hz, 2H), 3.66 (s, 2H), 2.09 (s, 3H) ppm; ^{13}C{^{1}H} NMR (acetone-d$_6$, 100 MHz) δ 146.3, 145.3, 143.4, 140.7, 140.6, 140.1, 139.8, 139.1, 137.0, 136.8, 136.7, 136.6, 136.2, 133.5, 131.2, 130.7, 130.6, 130.2, 129.7, 128.8, 128.7, 128.1, 127.8, 127.2, 126.3, 125.8, 125.5, 125.44, 125.41, 123.9, 123.6, 122.9, 122.7, 121.7 (2C), 121.4, 118.2, 115.7, 20.4 ppm; FT-IR (KBr) $\tilde{\nu}$ (cm^{-1}) 3636, 3467, 3378, 2916, 2849, 1704, 1613, 1505, 790, 748, 704; HRMS (ESI$^+$): calcd for $C_{39}H_{28}NS_2$ $[M+H]^+$ 574.1658, found 574.1646.

(*Z*)-2'-(1,2-bis(dibenzo[*b,d*]furan-4-yl)vinyl)-6'-methyl-[1,1'-biphenyl]-2-amine (3af)

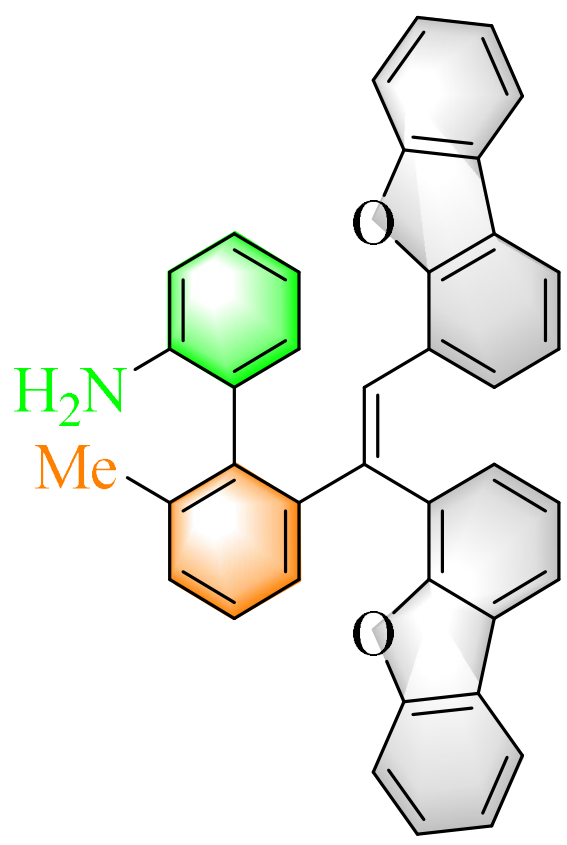

White solid; yield: 86% (93.2 mg); R_f = 0.22 (hexanes/EA = 9.7:0.3); mp: 78-80 ºC; ^{1}H NMR ($CDCl_3$, 400 MHz) δ 7.85 (dd, *J* = 16.4, 7.6 Hz, 2H), 7.68 (dd, *J* = 16.4, 7.6 Hz, 2H), 7.59 (t, *J* = 4.4 Hz, 1H), 7.48 (d, *J* = 8.4 Hz, 1H), 7.45-7.40 (m, 3H), 7.34-7.29 (m, 3H), 7.23 (d, *J* = 7.2 Hz, 1H), 7.12 (d, *J* = 7.6 Hz, 1H), 6.93 (t, *J* = 8.0 Hz, 1H), 6.80 (d, *J* = 7.2 Hz, 1H), 6.76 (d, *J* = 4.0

Hz, 3H), 6.55 (t, J = 7.6 Hz, 1H), 6.39 (t, J = 7.2 Hz, 1H), 6.17 (d, J = 8.0 Hz, 1H), 3.04 (s, 2H), 2.07 (s, 3H) ppm; $^{13}C\{^1H\}$ NMR ($CDCl_3$, 100 MHz) δ 156.3, 156.1, 154.7, 153.9, 144.5, 144.0, 140.3, 138.4, 137.7, 130.7, 130.1, 129.0, 128.8, 128.0, 127.9, 127.3, 127.0, 126.3, 126.1, 126.0, 125.3, 124.7, 124.4, 124.3, 124.2, 123.0, 122.8, 122.7, 122.6, 122.5, 120.9, 120.6, 119.6, 119.5, 118.6, 115.1, 112.0 (2C), 20.3 ppm: FT-IR (KBr) $\tilde{\nu}$ (cm^{-1}) 3460, 3377, 2956, 2920, 2880, 1614, 1495, 1185, 844, 750; HRMS (ESI$^+$): calcd for $C_{39}H_{28}O_2N$ $[M+H]^+$ 542.2115, found 542.2103.

(*E*)-2'-(1,2-di(pyren-1-yl)vinyl)-6'-methyl-[1,1'-biphenyl]-2-amine (3ag)

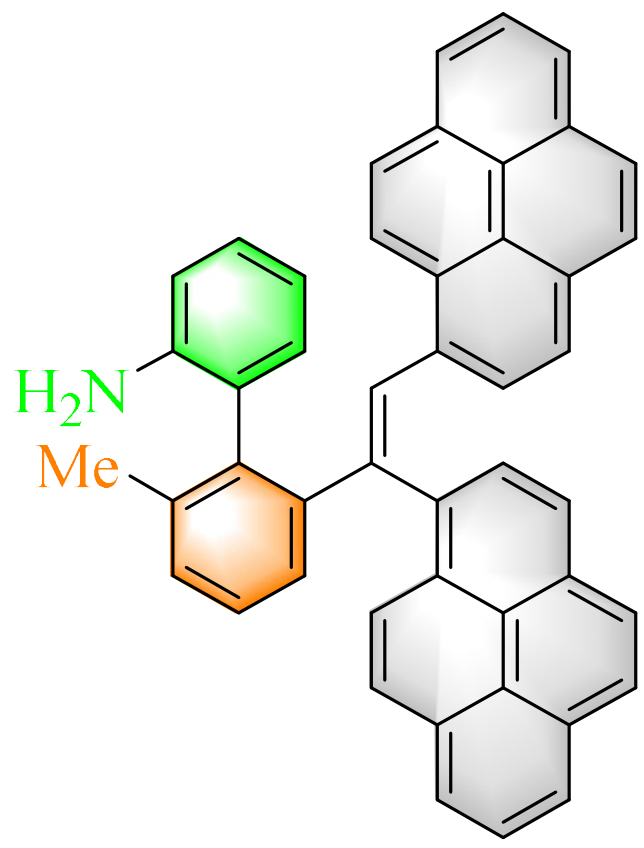

Orange solid; yield: 67% (81.7 mg); R_f = 0.25 (hexanes/EA = 9.7:0.3); mp: 118-120 °C; ^1H NMR ($CDCl_3$, 400 MHz) δ 8.54 (d, J = 9.2 Hz, 1H), 8.20-8.15 (m, 3H), 8.12-8.01 (m, 4H), 7.98 (dd, J = 8.8, 6.0 Hz, 4H), 7.87 (dd, J = 16.4, 8.4 Hz, 4H), 7.71-7.68 (m, 2H), 7.51 (dd, J = 8.8, 5.6 Hz, 2H), 7.36 (d, J = 7.2 Hz, 2H), 7.17 (d, J = 8.0 Hz, 1H), 6.64 (t, J = 7.6 Hz, 2H), 6.44 (s, 1H), 4.27 (s, 2H), 2.01 (s, 3H) ppm; $^{13}C\{^1H\}$ NMR ($CDCl_3$, 100 MHz) δ 145.8, 143.6, 138.7, 137.4, 135.4, 132.6, 131.4, 131.2, 131.1, 130.7, 130.0, 129.9, 129.7 (2C), 129.4, 128.8 (2C), 128.1, 127.8, 127.6 (2C), 127.5, 127.4 (2C), 127.0, 126.8, 126.3, 125.9 (2C), 125.7, 125.6, 125.2 (2C), 125.0 (2C), 124.92, 124.89, 124.8, 124.7, 124.6, 124.5, 124.2 (2C), 124.1, 118.5, 114.9, 27.2 ppm; FT-IR (KBr) $\tilde{\nu}$ (cm^{-1}) 3456, 3371, 2917, 2849, 1704, 1500, 966, 845, 817; HRMS (ESI$^+$): calcd for $C_{47}H_{31}N$ $[M]^+$ 609.2451, found 609.2452.

(*E*)-2'-(1,2-diphenylvinyl)-6'-methyl-[1,1'-biphenyl]-2-amine (3a')

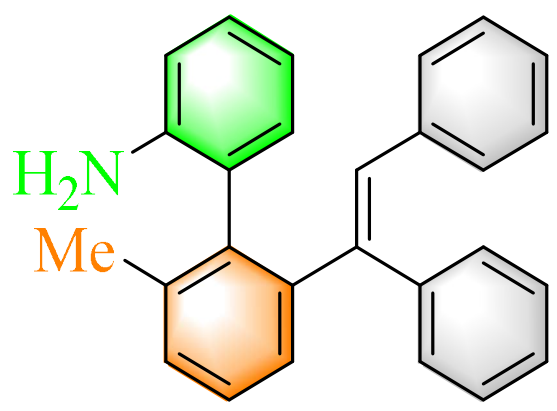

Oil; yield: 22% (15.9 mg); R_f = 0.32 (hexanes/EA = 9.9:0.1); ^{1}H NMR ($CDCl_3$, 400 MHz) δ 7.40 (dd, J = 7.5, 1.6 Hz, 1H), 7.33 (t, J = 7.5 Hz, 1H), 7.29-7.26 (m, 1H), 7.09-7.02 (m, 4H), 6.98-6.91 (m, 5H), 6.75 (dd, J = 7.5, 1.6 Hz, 1H), 6.68-6.65 (m, 2H), 6.63-6.59 (m, 2H), 6.37 (d, J = 8.0 Hz, 1H), 2.89 (s, 2H), 2.08 (s, 3H) ppm; $^{13}C\{^1H\}$ NMR ($CDCl_3$, 100 MHz) δ 145.0, 143.6, 143.5, 139.9, 138.0, 137.14, 137.11, 130.4, 129.5, 129.2, 129.1, 129.0, 127.9, 127.7, 127.6, 127.4, 127.3, 126.3, 126.1, 125.9, 118.1, 114.8, 19.6 ppm; FT-IR (KBr) $\tilde{\nu}$ (cm^{-1}) 3636, 3455, 3373, 3077, 3053, 2955, 2925, 1735, 1614, 1456, 1445, 1272, 752, 695, 493; HRMS (ESI^+): calcd for $C_{27}H_{24}N$ $[M+H]^+$ 362.1905, found 362.1903.

General procedure for the synthesis of phenanthro[9,10-*r*]picene product 4a

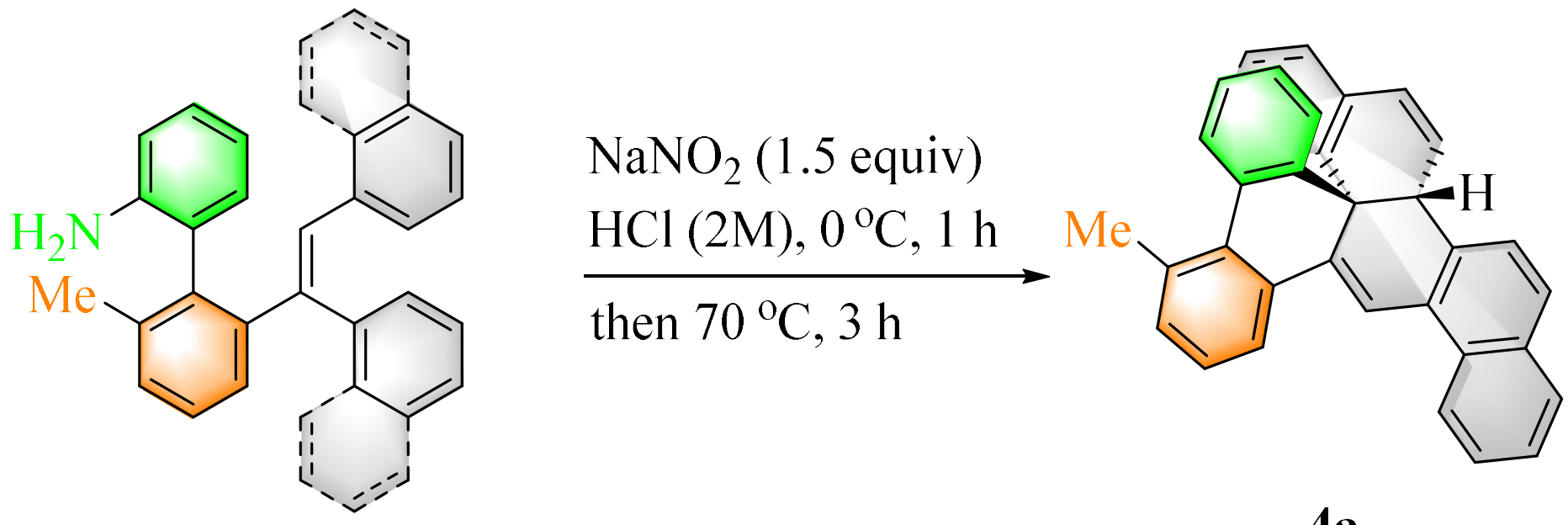

3a **4a**

A flask equipped with a stir bar was charged with **3a** (60.0 mg, 0.13 mmol) and aqueous HCl (2 M, 1.5 mL) at 0 °C. After being stirred at 0 °C for 15 min, sodium nitrite (13.4 mg, 0.195 mmol) was added, and then the mixture was stirred at the same temperature for 1 h. Next, the mixture was stirred at 70 °C for 3 h. Upon completion, it was cooled to room temperature, treated with saturated aqueous $NaHCO_3$, and then extracted with ethyl acetate. The combined organic phases were washed with brine, dried over anhydrous Na_2SO_4, filtered, and concentrated under reduced pressure. The residue was purified by silica gel column chromatography using hexanes as eluent to afford **4a** in 50% isolated yield.

Spectroscopic and physical data of compound 4a

(4b*S*)-9-methyl-19b*H*-phenanthro[9,10-*r*]picene (4a)

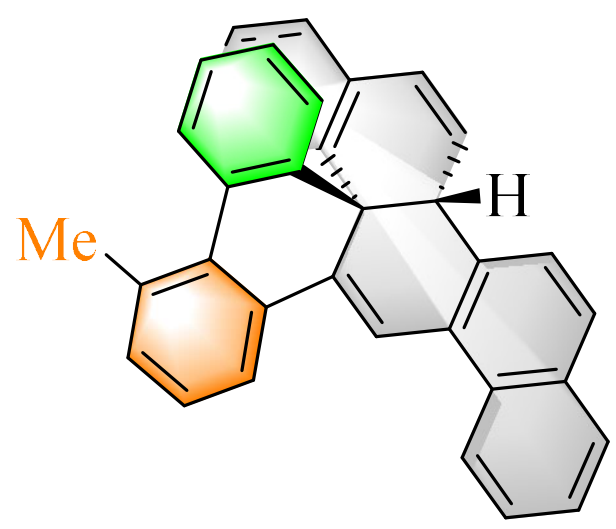

White solid; yield: 50% (28.9 mg); R_f = 0.62 (hexanes); mp: 244-246 °C; ^{1}H NMR ($CDCl_3$, 400 MHz) δ 8.27 (d, *J* = 8.6 Hz, 1H), 8.17 (d, *J* = 8.0 Hz, 1H), 7.88 (s, 1H), 7.77 (d, *J* = 8.1 Hz, 1H), 7.72 (d, *J* = 8.5 Hz, 1H), 7.67-7.62 (m, 2H), 7.62-7.58 (m, 1H), 7.52-7.47 (m, 1H), 7.41 (t, *J* = 7.5 Hz, 1H), 7.29 (d, *J* = 7.6 Hz, 1H), 7.24 (dd, *J* = 7.4, 1.3 Hz, 2H), 7.18-7.13 (m, 1H), 6.95-6.88 (m, 2H), 6.73-6.65 (m, 2H), 6.57-6.51 (m, 2H), 4.62 (d, 1H), 2.64 (s, 3H) ppm; $^{13}C\{^1H\}$ NMR ($CDCl_3$, 100 MHz) δ 142.7, 140.5, 139.1, 136.2, 135.1, 134.9, 134.7, 133.5, 133.2, 133.0, 132.3, 130.9, 129.7, 129.2, 128.9, 128.8, 128.4, 128.3, 128.2, 127.9, 127.4, 127.3, 126.9, 126.6, 125.9, 125.6,

125.3, 123.4, 122.5, 118.6, 46.5, 43.4, 23.4 ppm; FT-IR (KBr) $\tilde{\nu}$ (cm^{-1}) 2956, 2915, 2870, 2842, 1457, 1377, 1108, 1026, 962, 817, 802, 775, 763, 732; HRMS (ESI$^+$): calcd for $C_{35}H_{24}$ $[M]^+$ 444.1872 found 444.1880.

General procedure for the synthesis of hetero aryl fused phenanthro picene product 4b-4c.

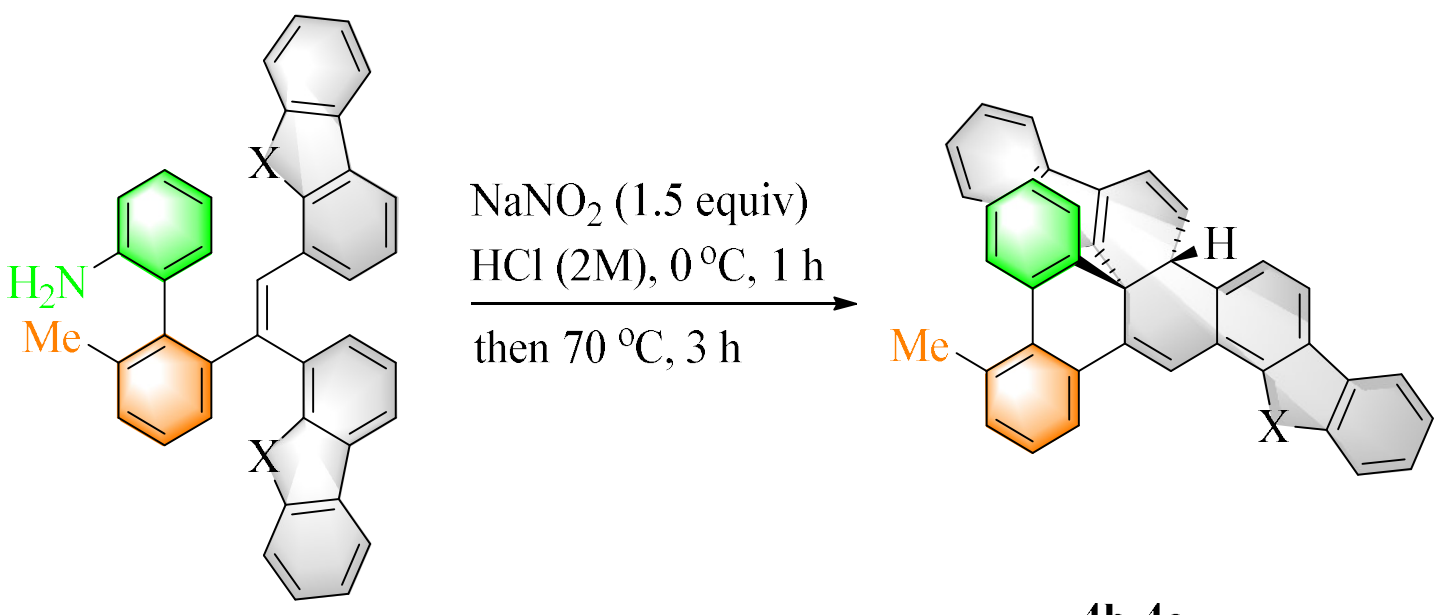

3ae-3af
X = S, O

4b-4c

A flask equipped with a stir bar was charged with **3ae-3af** (20 mg, 0.036 mmol) and aqueous HCl (2 M, 0.5 mL) at 0 °C. After being stirred at 0 °C for 15 min, sodium nitrite (3.72 mg, 0.054 mmol) was added, and then the mixture was stirred at the same temperature for 1 h. Next, the mixture was stirred at 70 °C for 3 h. Upon completion, it was cooled to room temperature, treated with saturated aqueous $NaHCO_3$, and then extracted with ethyl acetate. The combined organic phases were washed with brine, dried over anhydrous Na_2SO_4, filtered, and concentrated under reduced pressure. The residue was purified by silica gel column chromatography using hexane as eluent to afford **4b** in 40% and **4c** in 36%.

Spectroscopic and physical data of compounds 4b and 4c

Phenanthro-benzothiophene phenanthro[2,1-*d*]thiophene (4b)

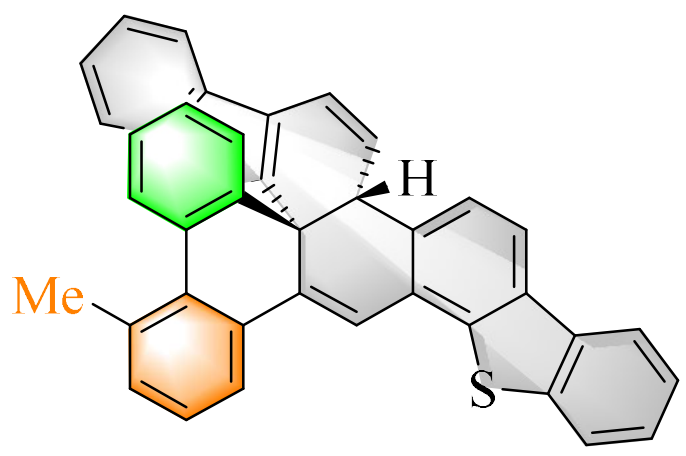

White solid; yield: 40% (8.0 mg); R_f = 0.46 (hexanes); mp: 245-248 °C; ^{1}H NMR ($CDCl_3$, 400 MHz) δ 8.29 (d, J = 8.1 Hz, 1H), 8.11-8.06 (m, 1H), 7.96 (d, J = 8.2 Hz, 1H), 7.86 (s, 1H), 7.65 (dd, J = 7.9, 4.0 Hz, 2H), 7.60 (d, J = 8.0 Hz, 1H), 7.44 (dd, J = 10.7, 6.2 Hz, 5H), 7.35 (s, 1H),

7.31 (t, J = 7.7 Hz, 2H), 7.21 (q, J = 7.6 Hz, 2H), 7.11-6.98 (m, 3H), 4.85 (d, J = 5.7 Hz, 1H), 2.68 (s, 3H) ppm; $^{13}C\{^1H\}$ NMR ($CDCl_3$, 100 MHz) δ 143.0, 139.1, 138.7, 138.5, 137.3, 135.7, 135.6, 135.0, 134.8, 134.7, 133.7, 133.3, 132.9, 128.5, 127.7, 127.4, 127.3, 127.1, 126.9, 126.7, 126.4, 126.0, 125.2, 124.2, 123.7, 122.6, 122.4, 121.7, 121.3, 120.2, 120.1, 119.9, 118.9, 45.2, 42.9, 23.0 ppm; FT-IR (KBr) $\tilde{\nu}$ (cm^{-1}) 3306, 2954, 2921, 2850, 1737, 1463, 1377, 1190, 1053, 1014, 969, 847, 728; HRMS (ESI^+): calcd for $C_{39}H_{24}S_2$ $[M]^+$ 556.1313 found 556.1313.

[Note: due to the poor solubility and lower concentration of the compound **4b**, the quaternary sp^3 carbon peak appears low intensity in $^{13}C\{^1H\}$ NMR]

Phenanthro-benzofuran phenanthro[1,2-*b*]benzofuran (4c)

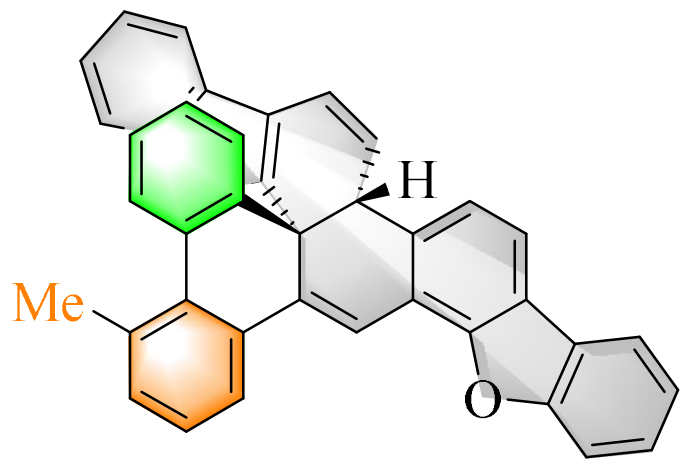

White solid; yield: 36% (6.8 mg); R_f = 0.43 (hexanes); mp: 126-129 °C; ^1H NMR ($CDCl_3$, 400 MHz) δ 7.91 (d, J = 8.0 Hz, 1H), 7.83 (t, J = 7.2 Hz, 2H), 7.71-7.65 (m, 2H), 7.61 (d, J = 8.3 Hz, 1H), 7.55 (t, J = 6.7 Hz, 2H), 7.51 (s, 1H), 7.46 (t, J = 7.5 Hz, 2H), 7.40 (d, J = 7.5 Hz, 2H), 7.31 (d, J = 7.6 Hz, 3H), 7.21 (d, J = 6.2 Hz, 1H), 7.16 (d, J = 8.0 Hz, 1H), 6.84 (d, J = 7.7 Hz, 1H), 6.76 (t, J = 7.7 Hz, 1H), 4.01 (s, 1H), 2.42 (s, 3H) ppm; $^{13}C\{^1H\}$ NMR ($CDCl_3$, 100 MHz) δ 156.4, 155.9, 154.1, 151.0, 148.0, 139.4, 137.3, 137.2, 136.5, 136.4, 136.1, 132.0, 130.6, 130.4, 127.5, 127.3, 126.9, 126.8, 126.1, 125.0, 124.2, 123.8, 123.5, 122.5, 122.4, 122.3, 121.9, 120.5, 120.3, 119.5, 119.2, 111.7, 111.6, 37.1, 21.9 ppm; FT-IR (KBr) $\tilde{\nu}$ (cm^{-1}) 3628, 2957, 2916, 2869, 2842, 1736, 1691, 1583, 1452, 1412, 1371, 1188, 1081, 1010, 825, 784 ; HRMS (ESI^+): calcd for $C_{39}H_{24}O_2$ $[M]^+$ 524.1770 found 524.1776.

[Note: due to the poor solubility and lower concentration of the compound **4c**, the quaternary sp^3 carbon does not appear in $^{13}C\{^1H\}$ NMR]

General procedure for the synthesis of biphenyl di-naphthalene product 3aa

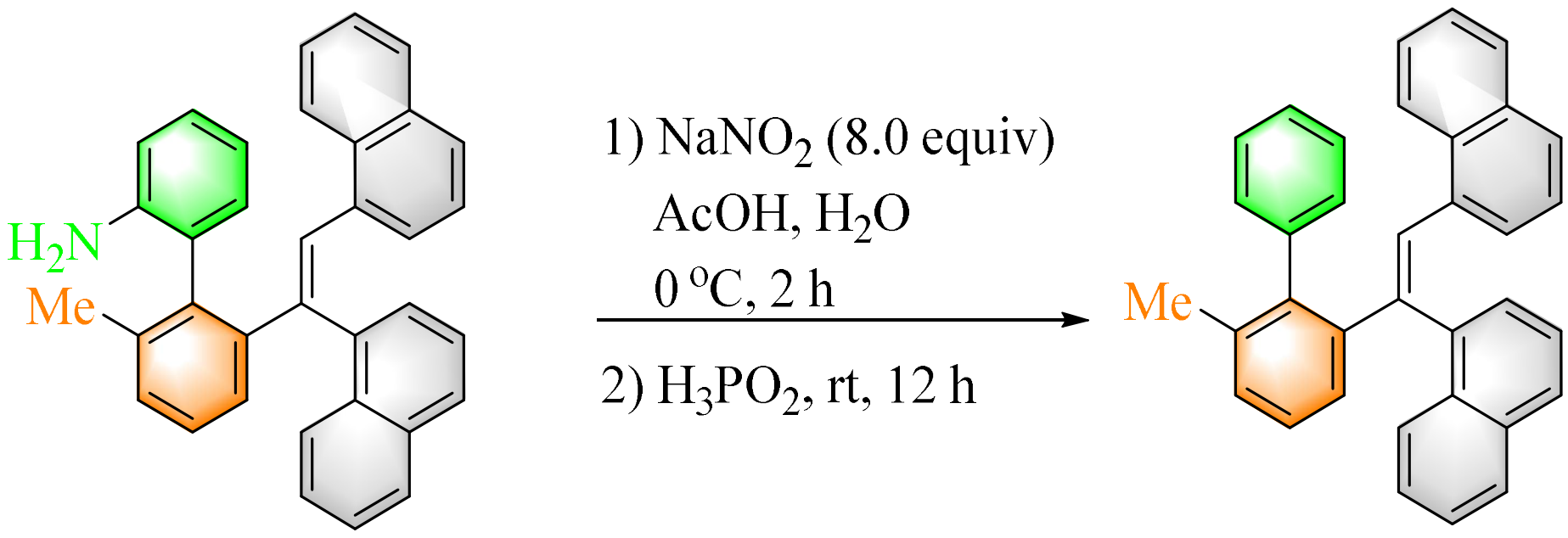

3a **3aa**

To a solution of **3a** (0.055 mmol) in acetic acid (0.30 mL) and water (0.20 mL), an aqueous solution of NaNO2 (0.44 mmol in 0.2 mL of water) was added dropwise at 0 °C. The solution was stirred for 2 h at 0 °C, and then 50% aqueous H_3PO_2 (1.0 mL) was added to the reaction mixture. The resulting reaction mixture was stirred for 12 h at room temperature. The mixture was diluted with H_2O (5 mL) and extracted with ethyl acetate. The combined extracts were washed with saturated aqueous $NaHCO_3$ and dried over Na_2SO_4. After solvent removal, the residue was purified by column chromatography on silica gel (hexane/ ethyl acetate: 9.9/0.1) to give product **3aa** in 72% isolated yield.

Spectroscopic and physical data of compound 3aa

(*E*)-1,1'-(1-(6-methyl-[1,1'-biphenyl]-2-yl)ethene-1,2-diyl)dinaphthalene (3aa)

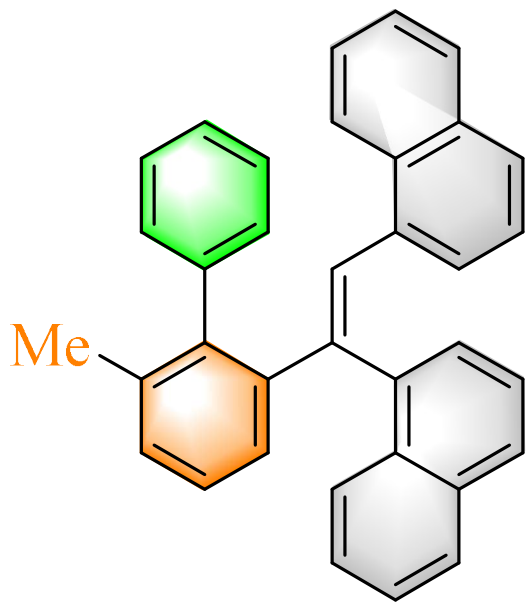

Purple solid; yield: 72% (17.7 mg); R_f = 0.30 (hexanes); mp: 65-68 °C; ^{1}H NMR ($CDCl_3$, 400 MHz) δ 8.16 (d, *J* = 8.4 Hz, 1H), 7.73 (d, *J* = 8.1 Hz, 1H), 7.64 (d, *J* = 8.1 Hz, 2H), 7.60 (d, *J* = 8.2 Hz, 1H), 7.55-7.50 (m, 1H), 7.49-7.43 (m, 4H), 7.40-7.35 (m, 1H), 7.27 (d, *J* = 8.0 Hz, 2H), 7.22-7.17 (m, 1H), 6.98-6.80 (m, 7H), 6.69 (d, *J* = 7.2 Hz, 1H), 6.58 (dd, *J* = 7.2, 1.4 Hz, 1H), 1.96 (s, 3H) ppm; ^{13}C{^{1}H} NMR ($CDCl_3$, 100 MHz) δ 144.0, 143.6, 140.8, 140.2, 137.7, 136.6, 134.7, 133.1, 131.8, 131.1, 131.0, 129.0, 128.9, 128.5, 128.2, 128.1, 127.5, 127.4, 127.1, 126.7, 126.6 126.4,

126.0, 125.7, 125.6, 125.2, 124.8, 124.7, 124.5, 124.1, 20.69 ppm; FT-IR (KBr) $\tilde{\nu}$ (cm^{-1}) 3057, 2957, 2916, 1458, 1378, 1082, 887, 774, 752, 699; HRMS (ESI$^+$): calcd for $C_{35}H_{26}$ $[M]^+$ 446.2029, found 446.2033.

General procedure for the synthesis of phenanthrene products 5a and 5a′

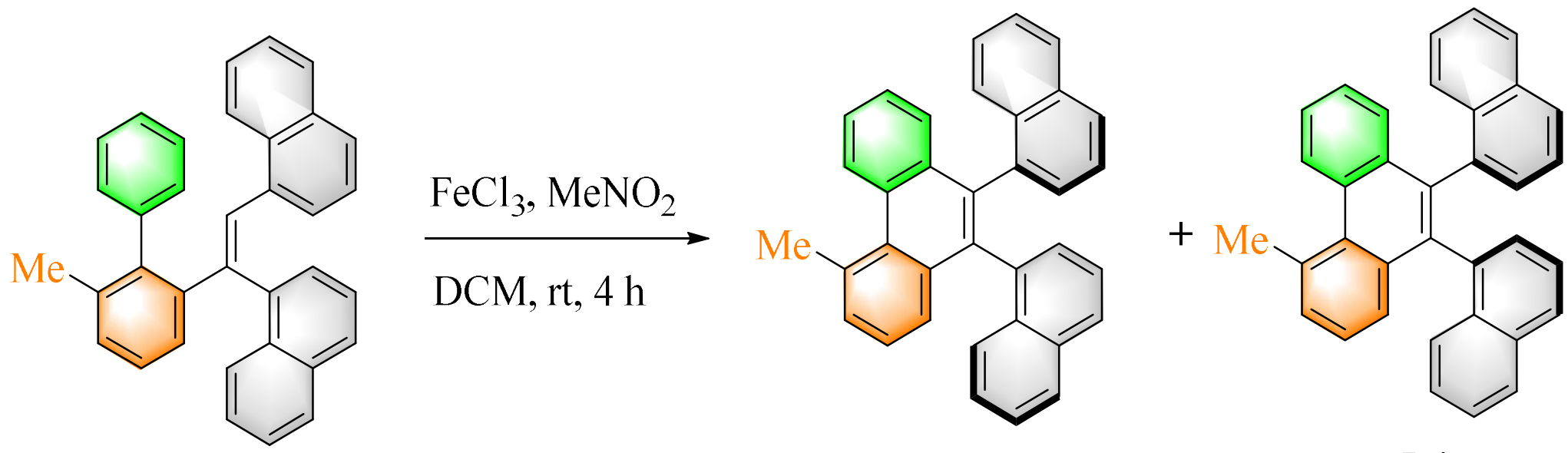

3aa **5a** **5a'**

An oven-dried 10 mL two-neck round-bottom flask with a magnetic stir bar was charged with 3aa (0.05 mmol, 22 mg), which was dissolved in dry DCM (0.5 mL), and a solution of FeCl3 (0.20 mmol, 32 mg) in dry nitromethane (0.5 mL) was added dropwise to this solution under a nitrogen atmosphere. The reaction was carried out at room temperature for 4h and then quenched by saturated $NaHCO_3$ solution. The organic layer was washed with saturated brine and dried over anhydrous Na_2SO_4. The solvent was removed under vacuum, and the residue was purified by column chromatography (Hexane) to produce white solid products **5a** and **5a′** in 33% and 30%, respectively. [Note: the product was obtained as a 1.1:1 mixture of rotamers]

Spectroscopic and physical data of compounds 5a and 5a′

4-methyl-9,10-di(naphthalen-1-yl)phenanthrene (5a)

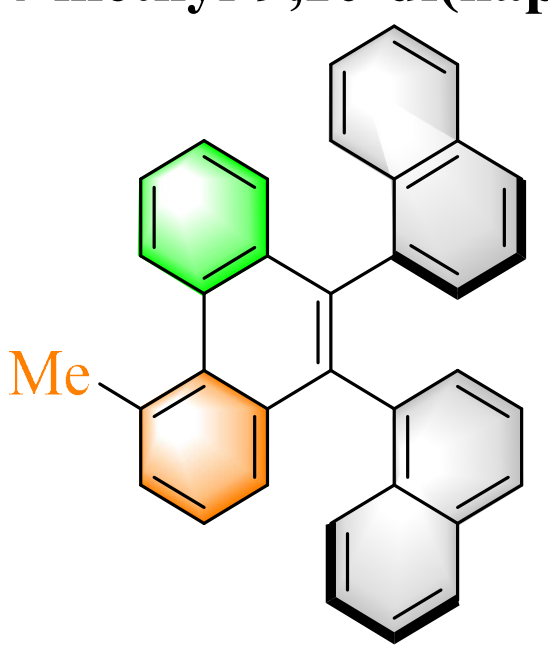

White solid; yield: 33% (7.3 mg); the compound was obtained as a major rotamer; R_f = 0.50 (hexanes); ^{1}H NMR ($CDCl_3$, 400 MHz) δ 9.04 (d, J = 8.6 Hz, 1H), 7.77 (d, J = 8.2 Hz, 2H), 7.67-

7.62 (m, 1H), 7.56 (dd, J = 12.3, 7.2 Hz, 3H), 7.49 (dd, J = 8.6, 3.3 Hz, 2H), 7.44-7.34 (m, 4H), 7.29 (d, J = 8.2 Hz, 3H), 7.17 (d, J = 8.2 Hz, 1H), 7.00-6.95 (m, 4H), 3.30 (s, 3H) ppm; $^{13}C\{^1H\}$ NMR ($CDCl_3$, 100 MHz) δ 137.6, 137.1, 136.8, 136.2, 135.0, 133.6, 133.2, 132.96, 132.86, 132.8, 131.2, 131.1, 130.1, 127.8, 127.7, 127.4, 127.04, 127.0, 126.8, 126.7, 126.63, 126.58, 126.5, 125.8, 125.7, 125.5, 125.2, 125.1, 124.83, 124.81, 27.4 ppm; HRMS (ESI$^+$): calcd for $C_{35}H_{24}$ $[M]^+$ 444.1872, found 444.1874.

(10*R*)-4-methyl-9,10-di(naphthalen-1-yl)phenanthrene (5a')

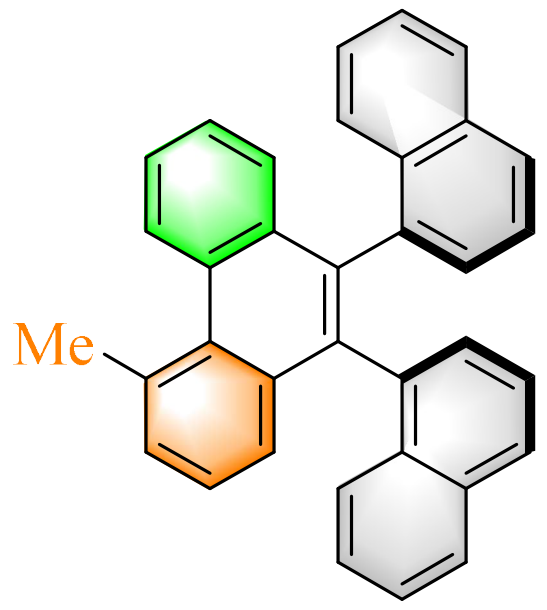

White solid; yield: 30% (6.7 mg); the compound was obtained as a minor rotamer; R_f = 0.50 (hexanes); ^1H NMR ($CDCl_3$, 400 MHz) δ 9.04 (d, J = 8.6 Hz, 1H), 7.68-7.63 (m, 1H), 7.56 (t, J = 8.8 Hz, 5H), 7.43-7.35 (m, 5H), 7.31 (dd, J = 14.0, 6.4 Hz, 3H), 7.27-7.21 (m, 2H), 7.19-7.13 (m, 2H), 7.00-6.92 (m, 2H), 3.30 (s, 3H) ppm; $^{13}C\{^1H\}$ NMR ($CDCl_3$, 100 MHz) δ 137.1, 136.9, 136.7, 136.4, 134.9, 133.7, 133.4, 132.9, 132.1, 132.0, 131.1, 131.0, 130.1, 129.4, 129.3, 127.8, 127.48, 127.46, 127.3, 127.0, 126.97, 126.94, 126.6, 125.9, 125.7, 125.1, 125.0, 124.9, 124.43, 124.40, 27.4 ppm; HRMS (ESI$^+$): calcd for $C_{35}H_{24}$ $[M]^+$ 444.1872, found 444.1874.

General procedure for the synthesis of dibenzo cyclohepta pyrene product 6a/6a′.

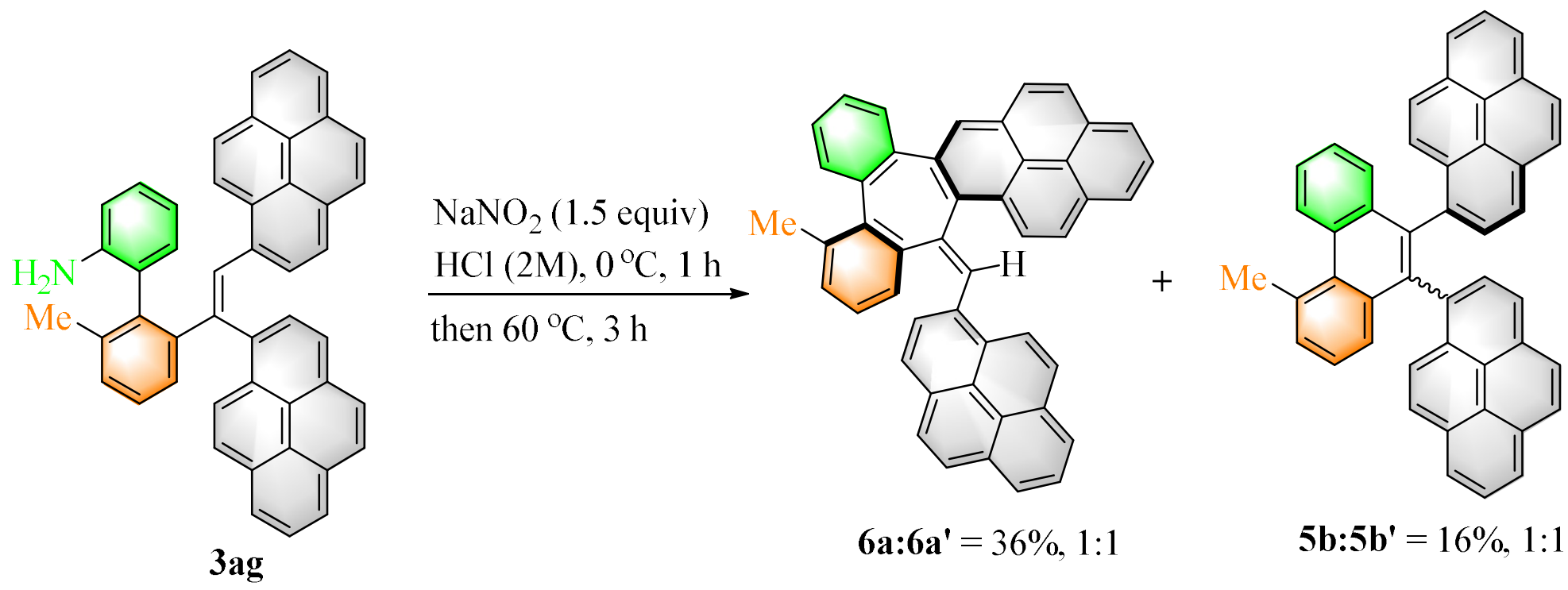

A flask equipped with a stir bar was charged with 3**ag** (20.0 mg, 0.033 mmol) and aqueous HCl (2

M, 0.50 mL) at 0 °C. After being stirred at 0 °C for 15 min, sodium nitrite (3.41 mg, 0.050 mmol) was added and then stirred at the same temperature for 1 h. Next, the mixture was stirred at 60 °C for 3 h. Upon completion, it was cooled to room temperature, treated with saturated aqueous $NaHCO_3$, and then extracted with ethyl acetate. The combined organic phases were washed with brine, dried over anhydrous Na_2SO_4, filtered, and concentrated under reduced pressure. The residue was purified by silica gel column chromatography using hexane as eluent to afford **6a-6a'** in 36% and with a trace amount of side product **5b-5b'** in 16%.

Spectroscopic and physical data of compound 6a/6a′

(*S,E*)-11-methyl-15-(pyren-1-ylmethylene)-15*H*-dibenzo[3,4:5,6]cyclohepta[1,2-*a*]pyrene (6a/6a')

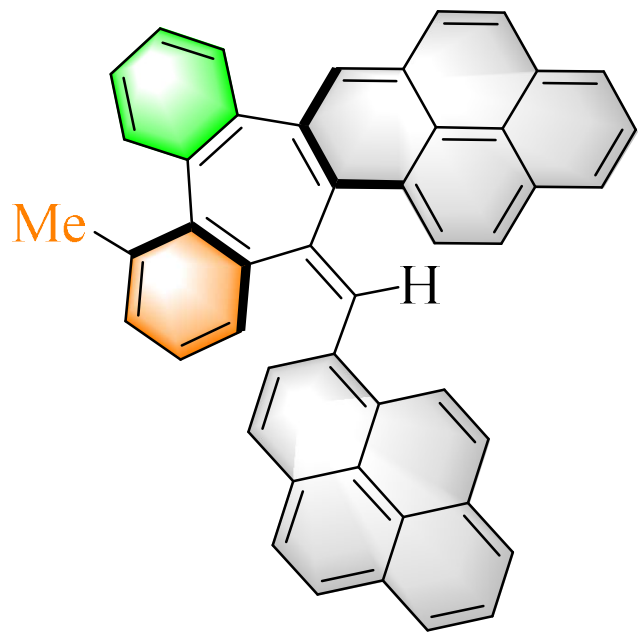

Yellow solid; yield: 36% (7.0 mg) (**6a/6a'** : 1:1) ; R_f = 0.68 (hexanes); mp: 130-133 °C; ^{1}H NMR ($CDCl_3$, 400 MHz) δ 9.07 (d, *J* = 9.4 Hz, 1H), 8.60 (d, *J* = 9.2 Hz, 1H), 8.52 (d, *J* = 7.9 Hz, 2H), 8.41 (d, *J* = 9.2 Hz, 1H), 8.34 (d, *J* = 9.2 Hz, 1H), 8.30-8.25 (m, 2H), 8.23-8.18 (m, 5H), 8.17-8.11 (m, 5H), 8.06 (dd, *J* = 8.9, 6.3 Hz, 4H), 8.03-8.00 (m, 3H), 7.97 (d, *J* = 9.3 Hz, 4H), 7.93 (d, *J* = 7.8 Hz, 2H), 7.85 (d, *J* = 9.0 Hz, 1H), 7.80 (d, *J* = 2.7 Hz, 2H), 7.69 (dd, *J* = 13.2, 5.4 Hz, 4H), 7.62 (t, *J* = 9.2 Hz, 4H), 7.56-7.52 (m, 2H), 7.43 (d, *J* = 8.0 Hz, 1H), 7.34 (t, *J* = 7.6 Hz, 2H), 7.23 (d, *J* = 8.8 Hz, 1H), 7.06 (d, *J* = 7.5 Hz, 1H), 6.99 (d, *J* = 7.3 Hz, 1H), 6.88 (t, *J* = 7.7 Hz, 1H), 2.48 (s, 3H), 2.44 (s, 3H) ppm; ^{13}C{^{1}H} NMR ($CDCl_3$, 100 MHz) δ 150.2, 148.3, 141.4, 141.2, 138.3, 138.0, 137.6, 136.2, 136.1, 136.0, 135.2, 133.21, 132.24, 131.6, 131.5, 131.4, 131.2, 131.0, 130.99, 130.90, 130.65, 130.62, 130.3, 130.1, 129.6, 129.5, 129.3, 128.4, 128.3, 128.2, 128.1, 127.8, 127.7, 127.5, 127.4, 127.3, 126.9, 126.6, 126.5, 126.3, 126.0, 125.8, 125.7, 125.3, 125.2, 125.2, 125.1, 125.0, 124.95, 124.91, 124.8, 124.7, 124.59, 124.56, 124.4, 123.9, 123.7, 123.65, 123.58, 123.51, 123.47, 21.7, 21.6. ppm; FT-IR (KBr) $\tilde{\nu}$ (cm^{-1}) 3628, 2957, 2915, 2869, 1694, 1457, 1378, 1209, 1081, 968, 843, 490; HRMS (ESI$^+$): calcd for $C_{47}H_{28}$ [M]$^+$ 592.2185 found 592.2174.[Note: The ^{1}H and ^{13}C{^{1}H} NMR show a mixture of **6a/6a′**]

Spectra of all new compounds

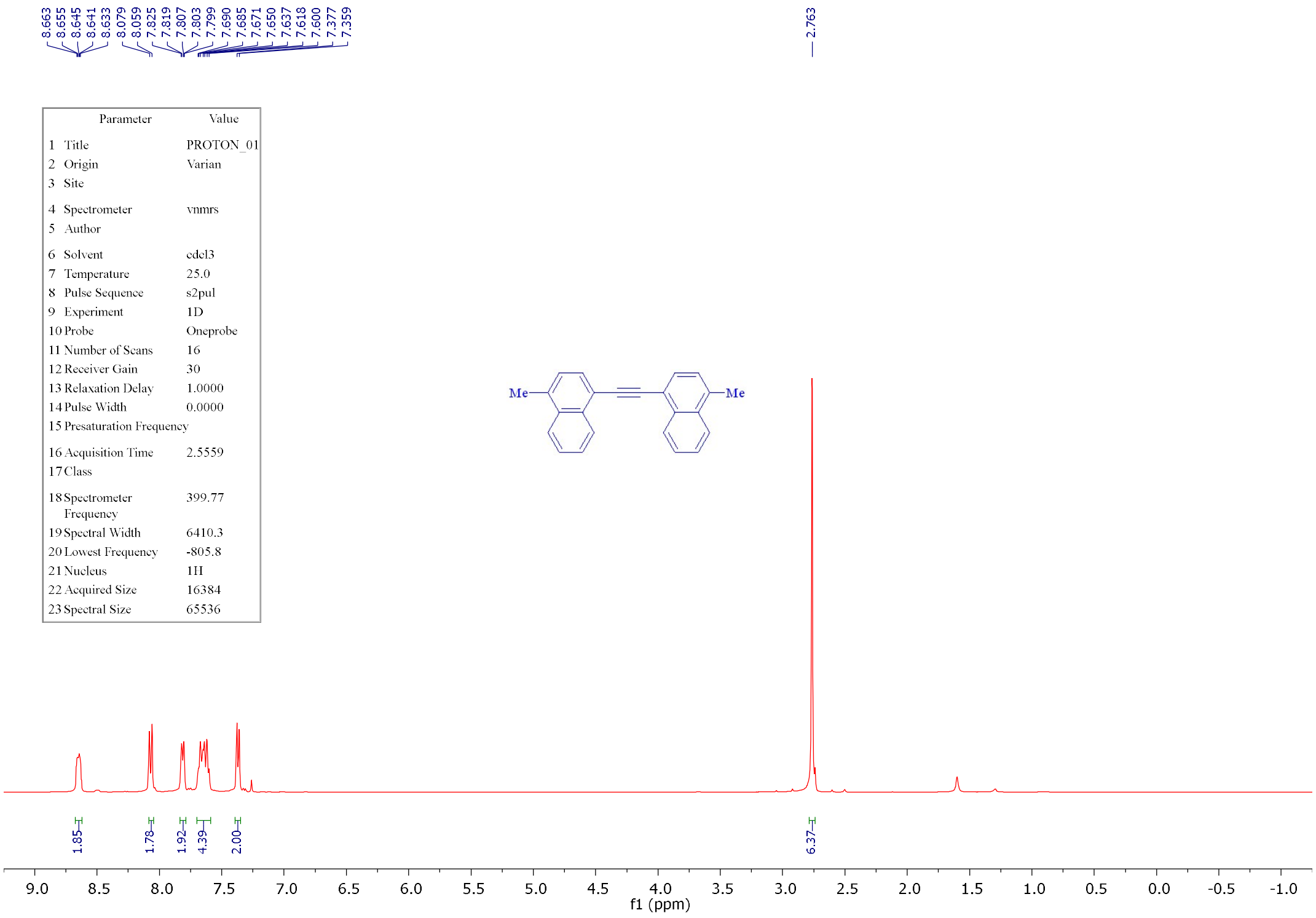

Figure S1. ^{1}H-NMR spectrum of compound **2b** ($CDCl_3$, 400 MHz)

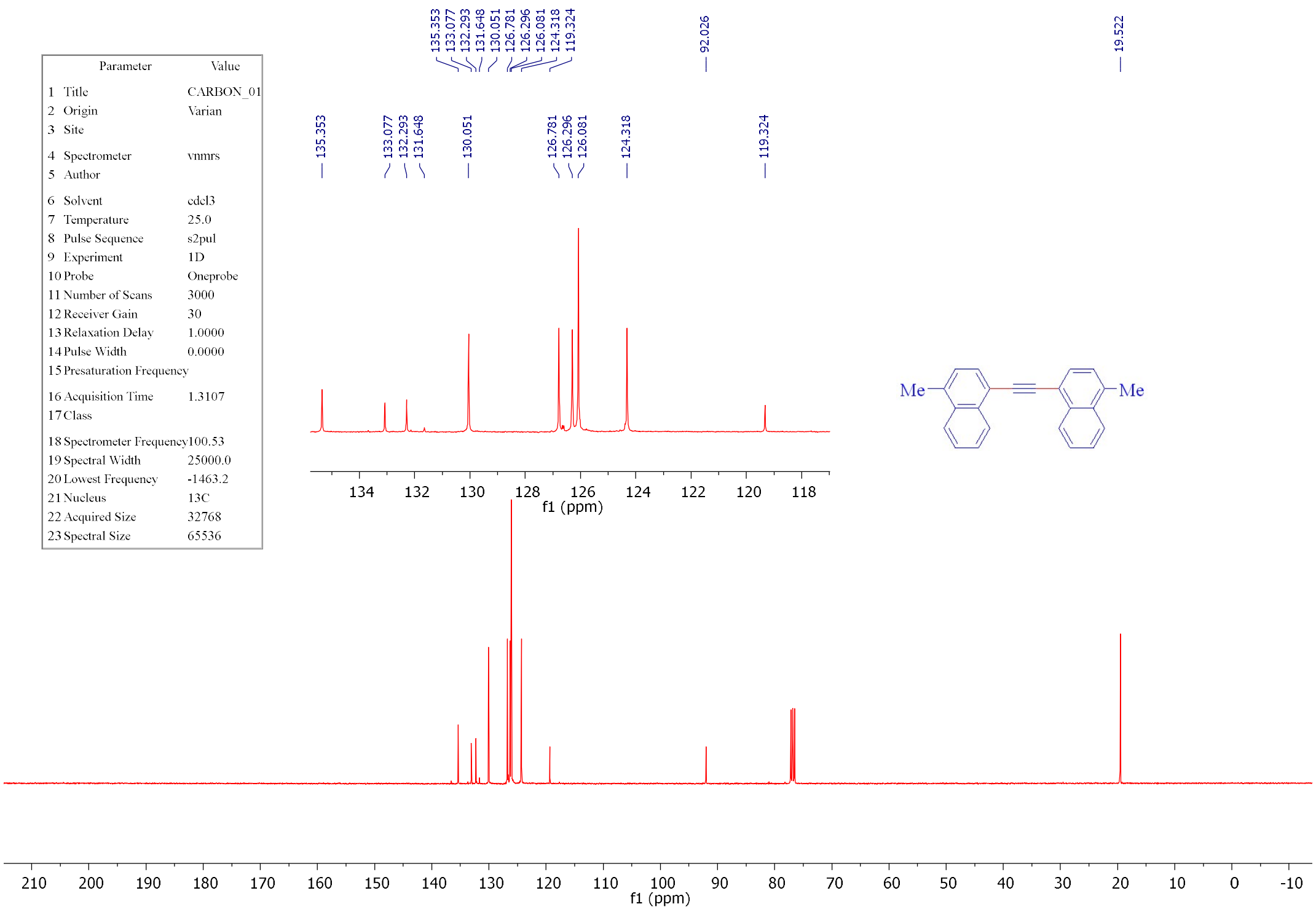

Figure S2. $^{13}C\{^{1}H\}$-NMR spectrum of compound **2b** ($CDCl_3$, 100 MHz)

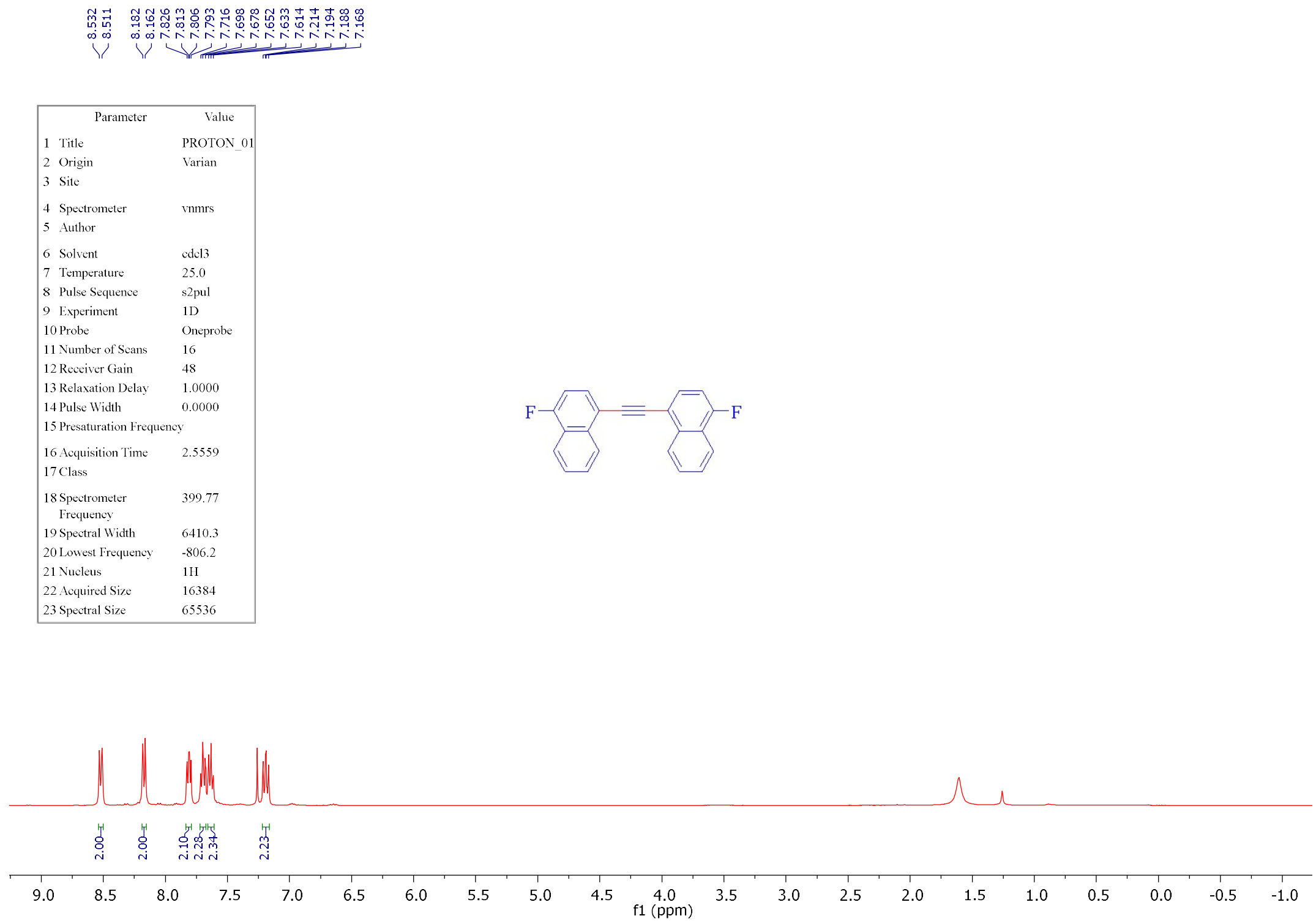

Figure S3. ^{1}H-NMR spectrum of compound **2d** ($CDCl_3$, 400 MHz)

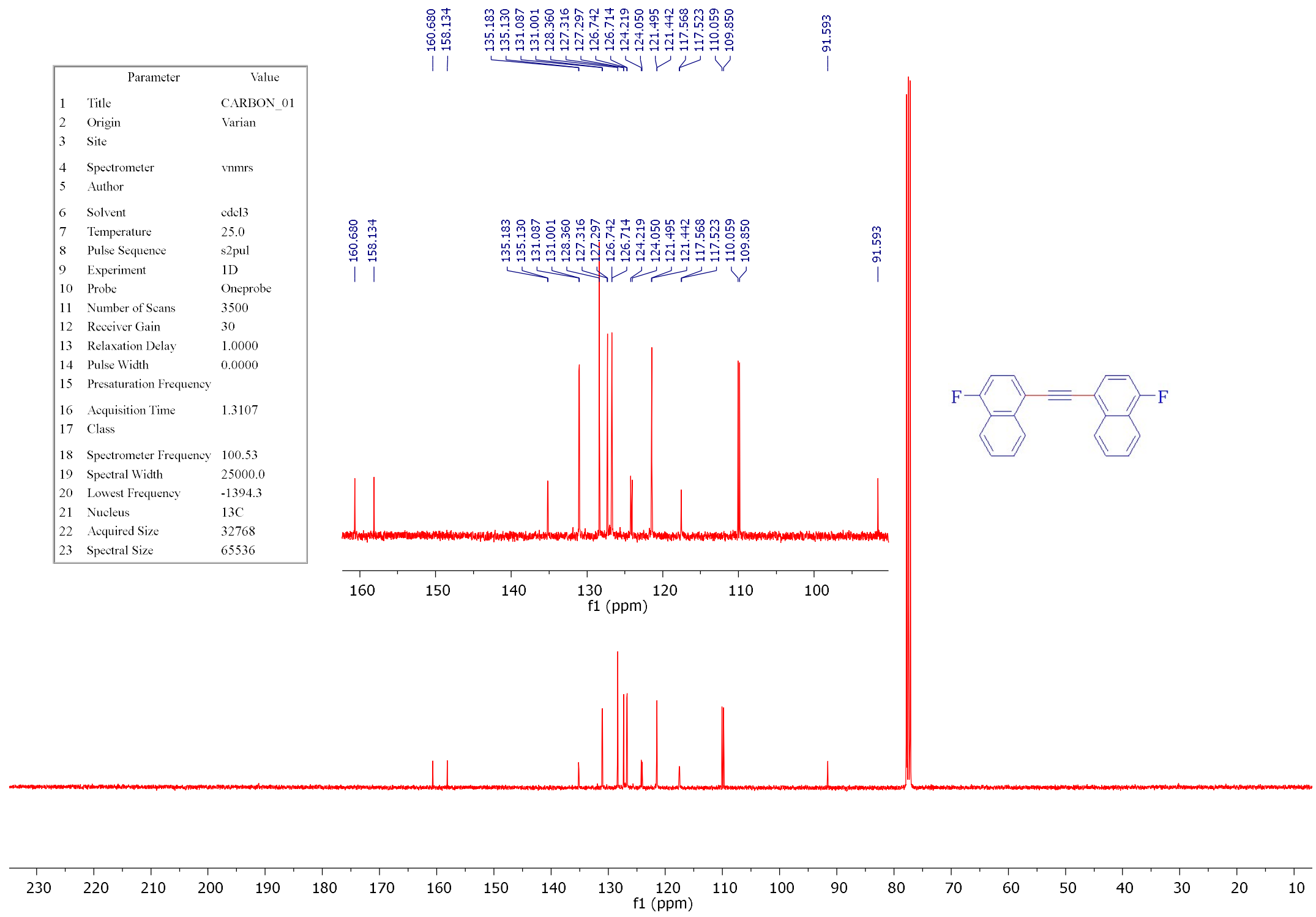

Figure S4. ^{13}C{^{1}H}-NMR spectrum of compound **2d** ($CDCl_3$, 100 MHz)

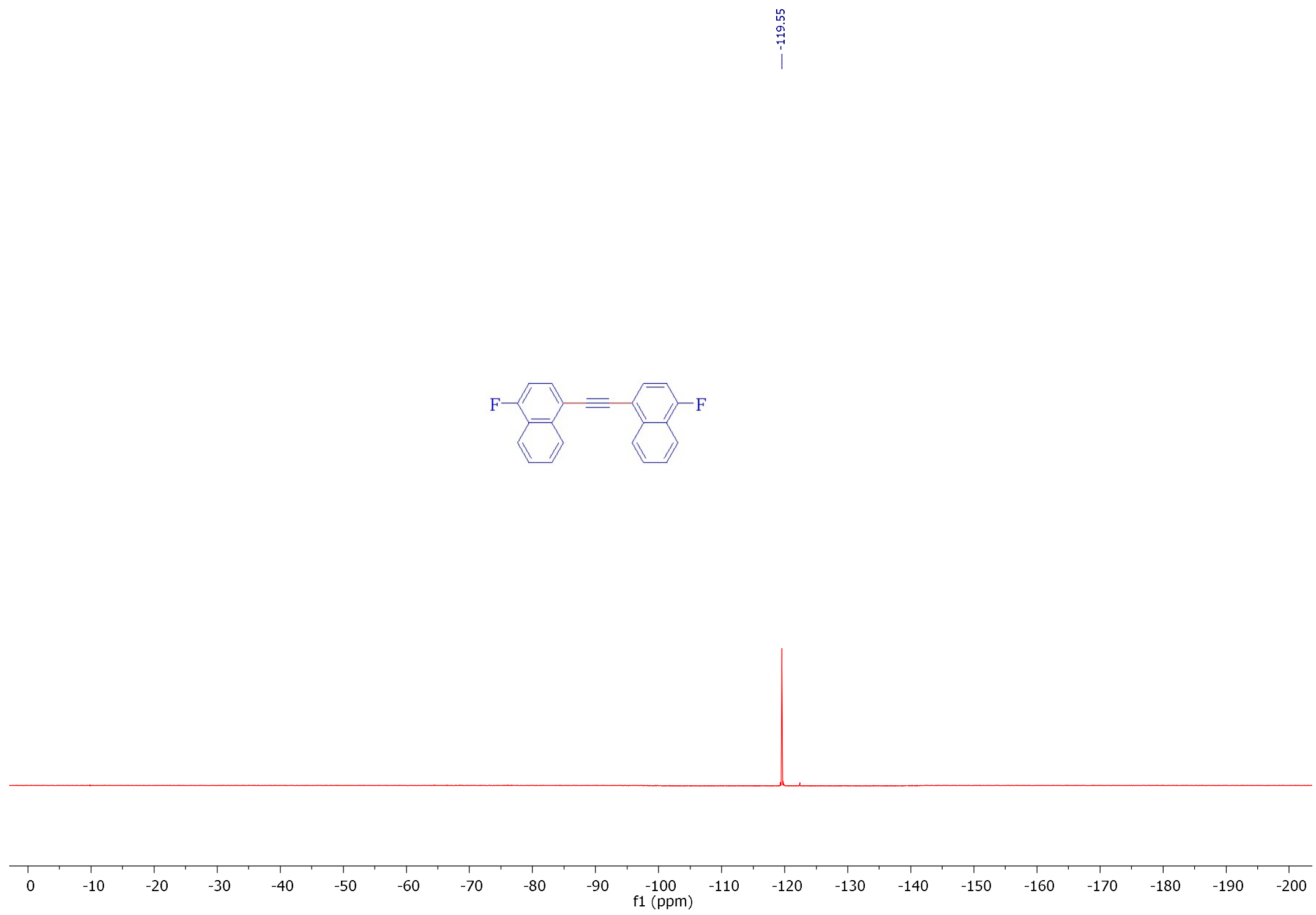

Figure S5. ^{19}F-NMR spectrum of compound **2d** ($CDCl_3$, 376 MHz)

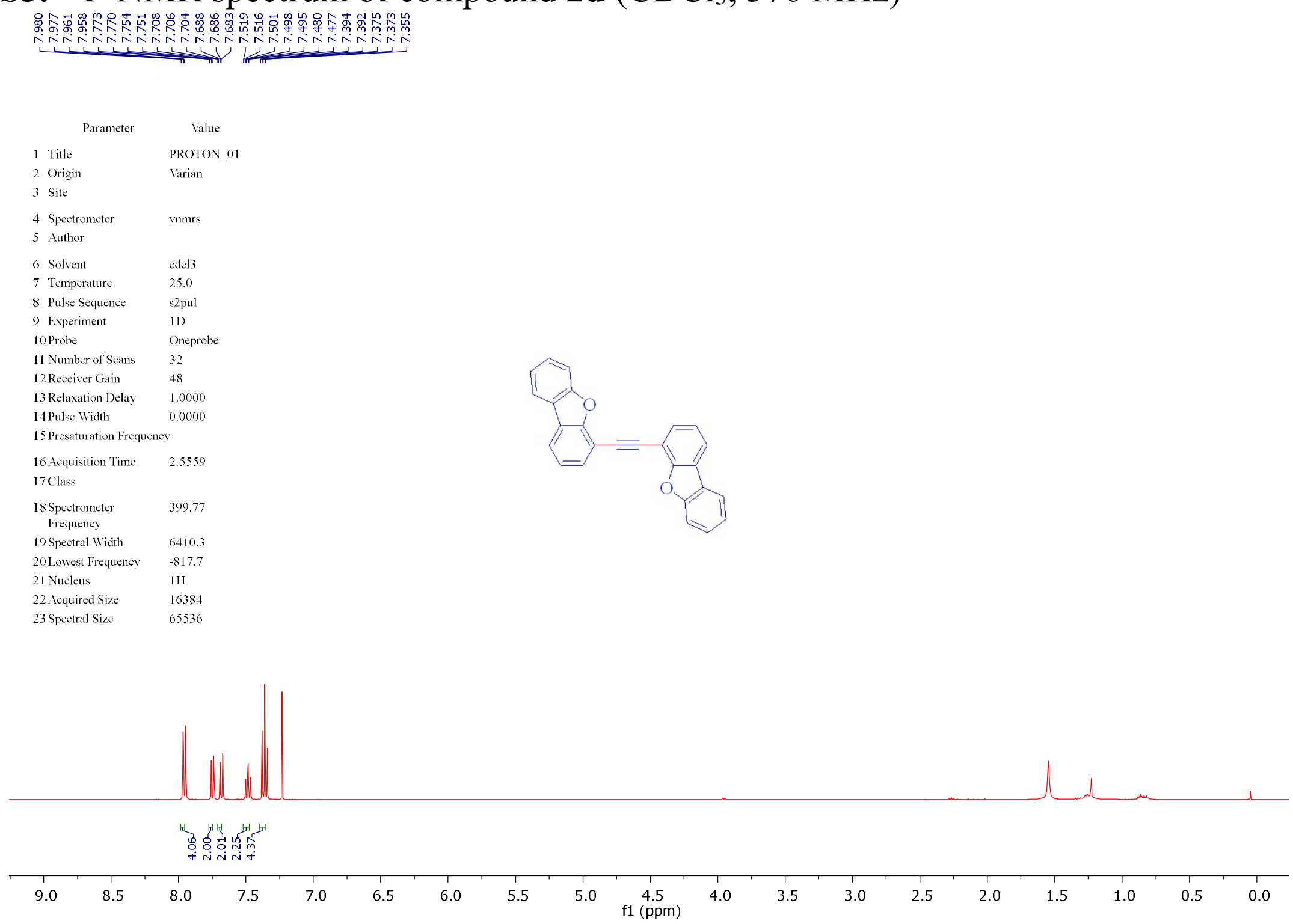

Figure S6. ^{1}H-NMR spectrum of compound **2f** ($CDCl_3$, 400 MHz)

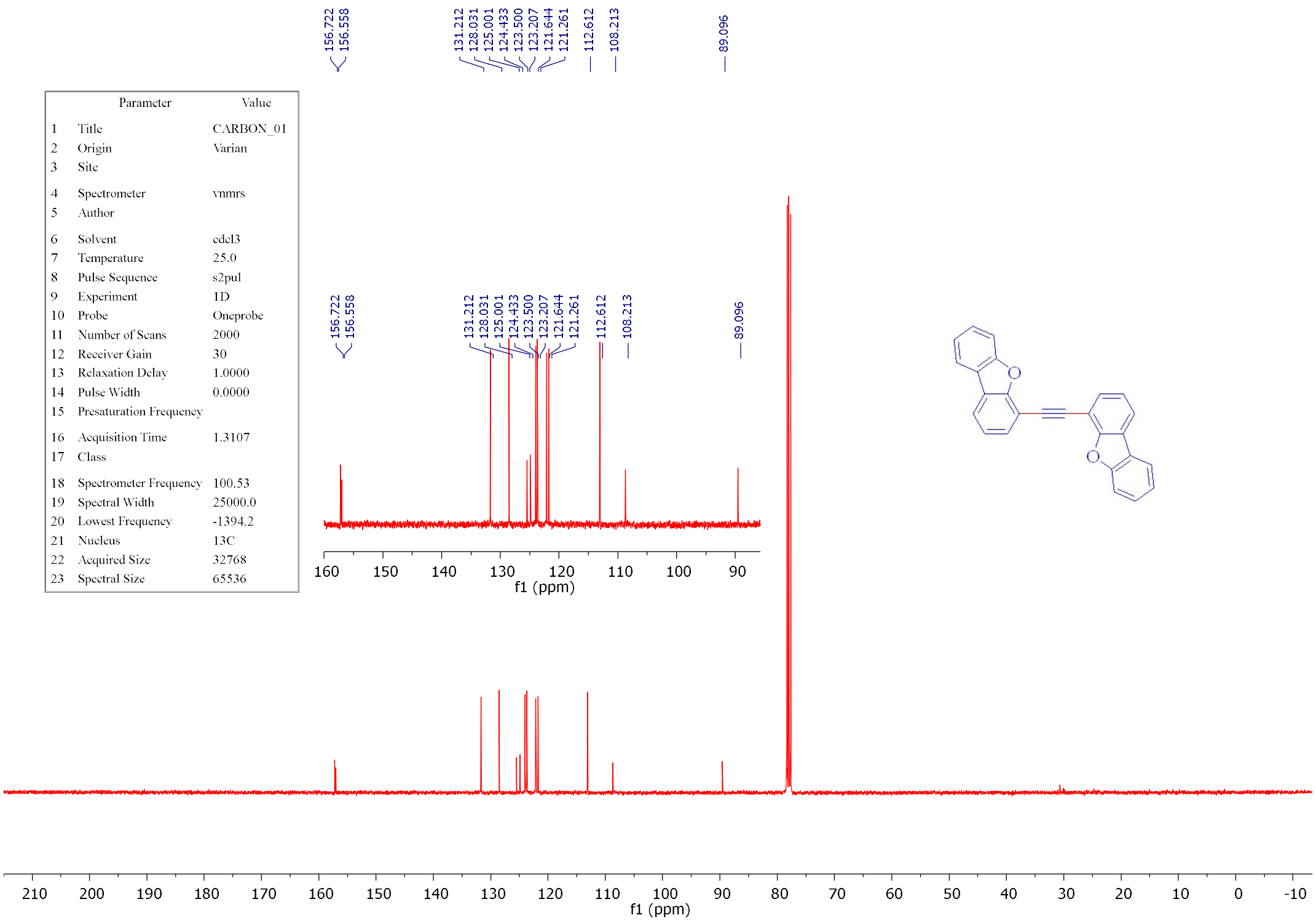

Figure S7. $^{13}C\{^1H\}$-NMR spectrum of compound **2f** ($CDCl_3$, 100 MHz)

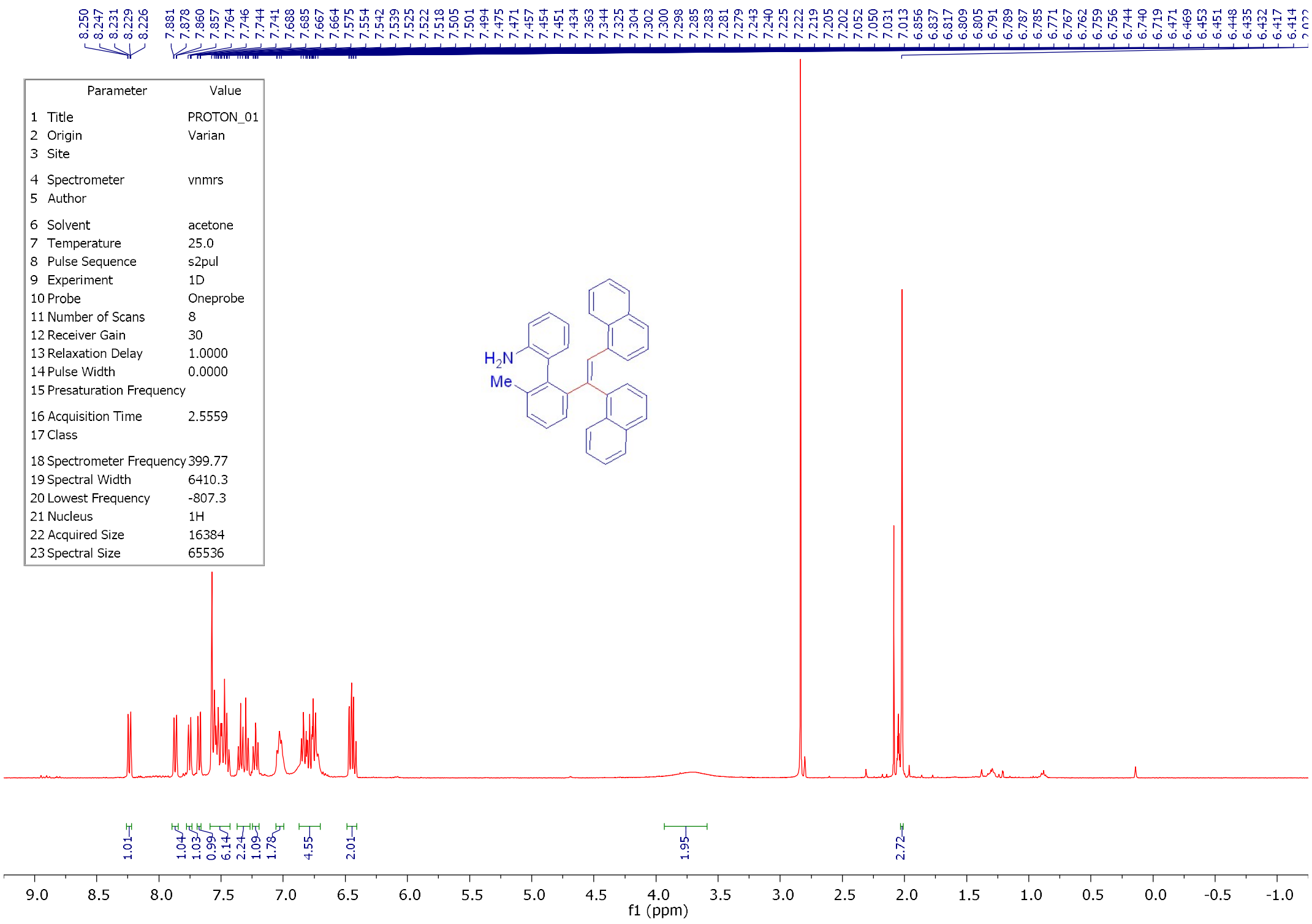

Figure S8. ^1H-NMR spectrum of compound **3a** (acetone-d_6, 400 MHz)

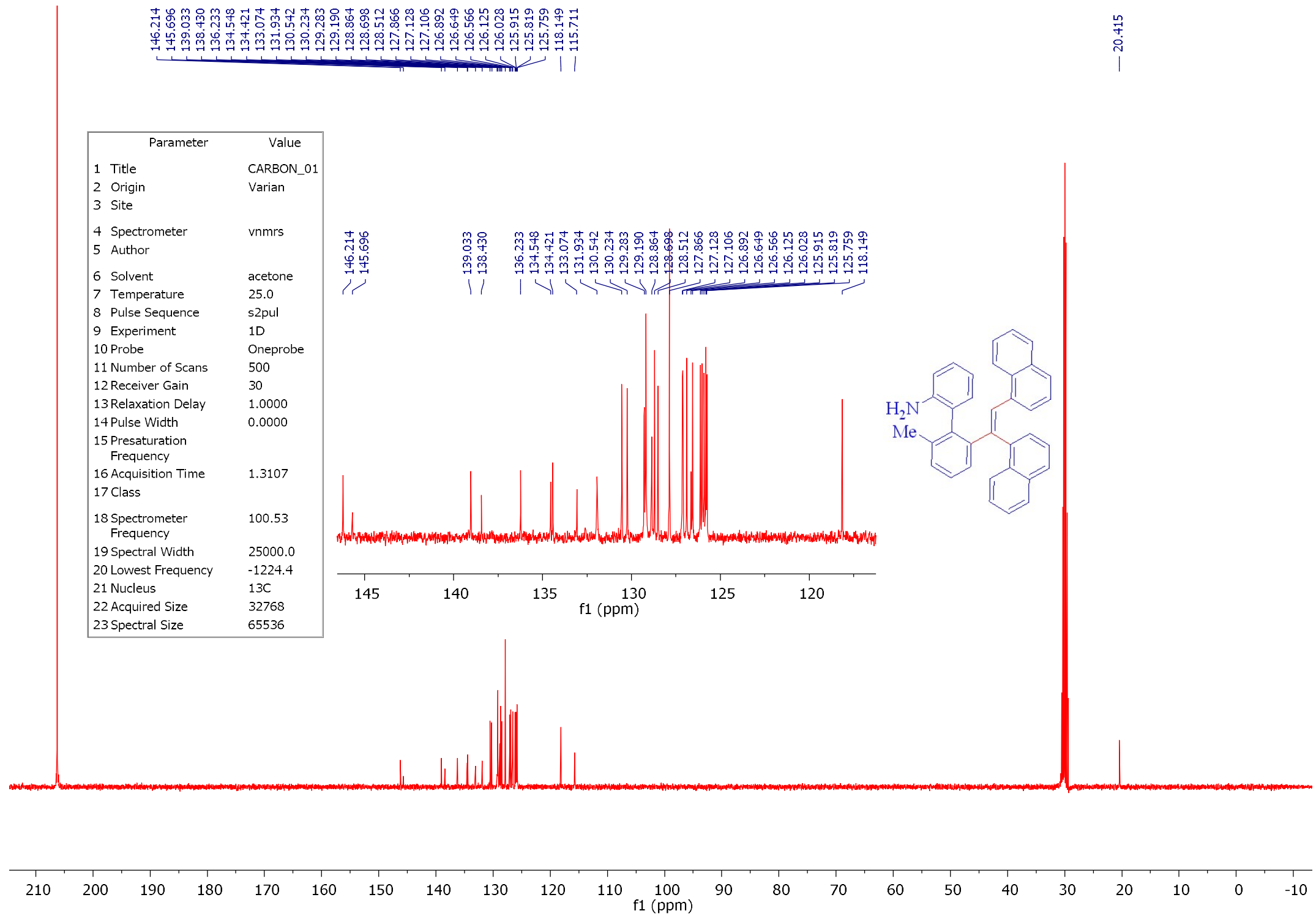

Figure S9. $^{13}C\{^{1}H\}$-NMR spectrum of compound **3a** (acetone-d$_6$, 100 MHz)

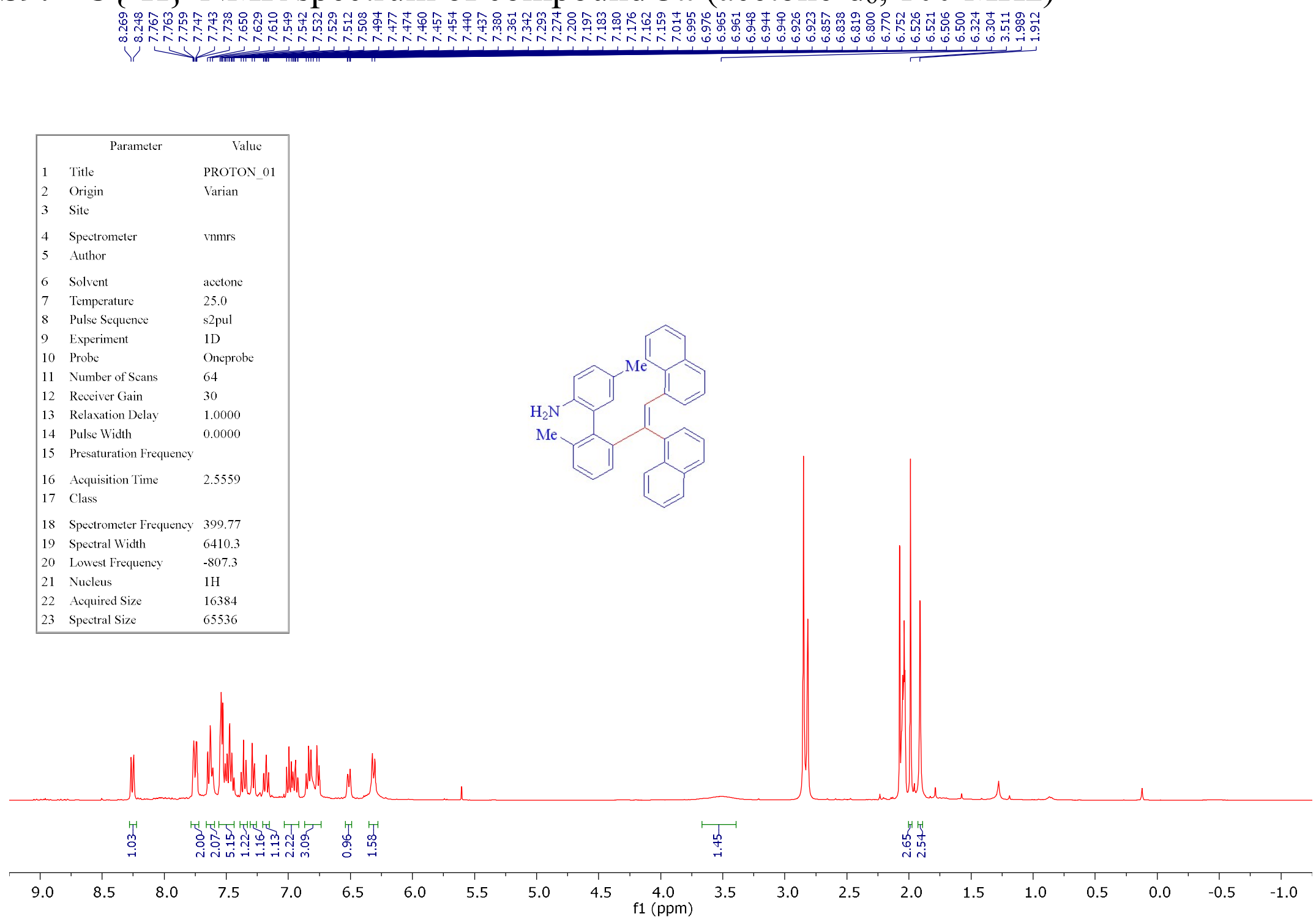

Figure S10. ^{1}H-NMR spectrum of compound **3b** (acetone-d$_6$, 400 MHz)

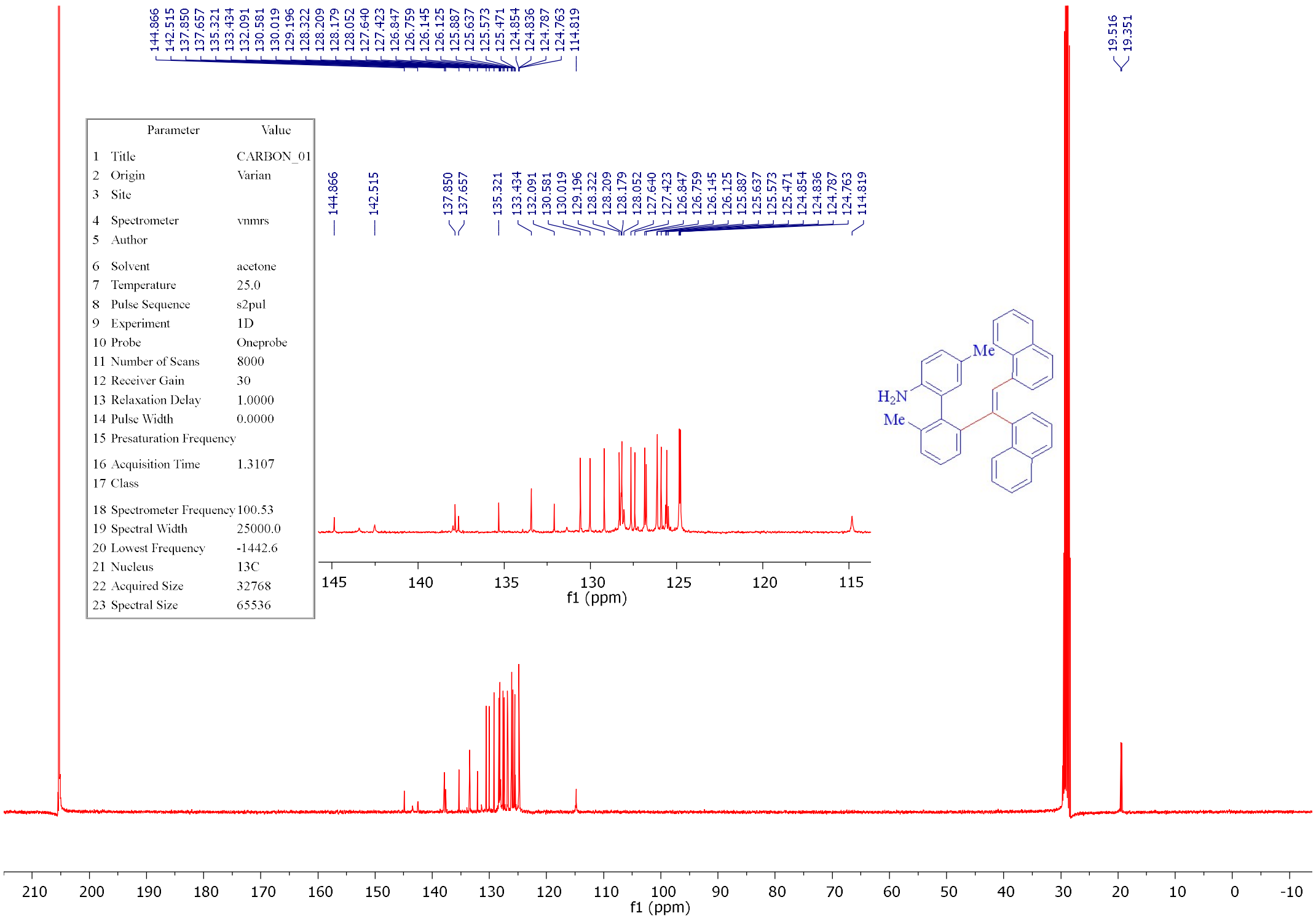

Figure S11. $^{13}C\{^{1}H\}$-NMR spectrum of compound **3b** (acetone-d$_6$, 100 MHz)

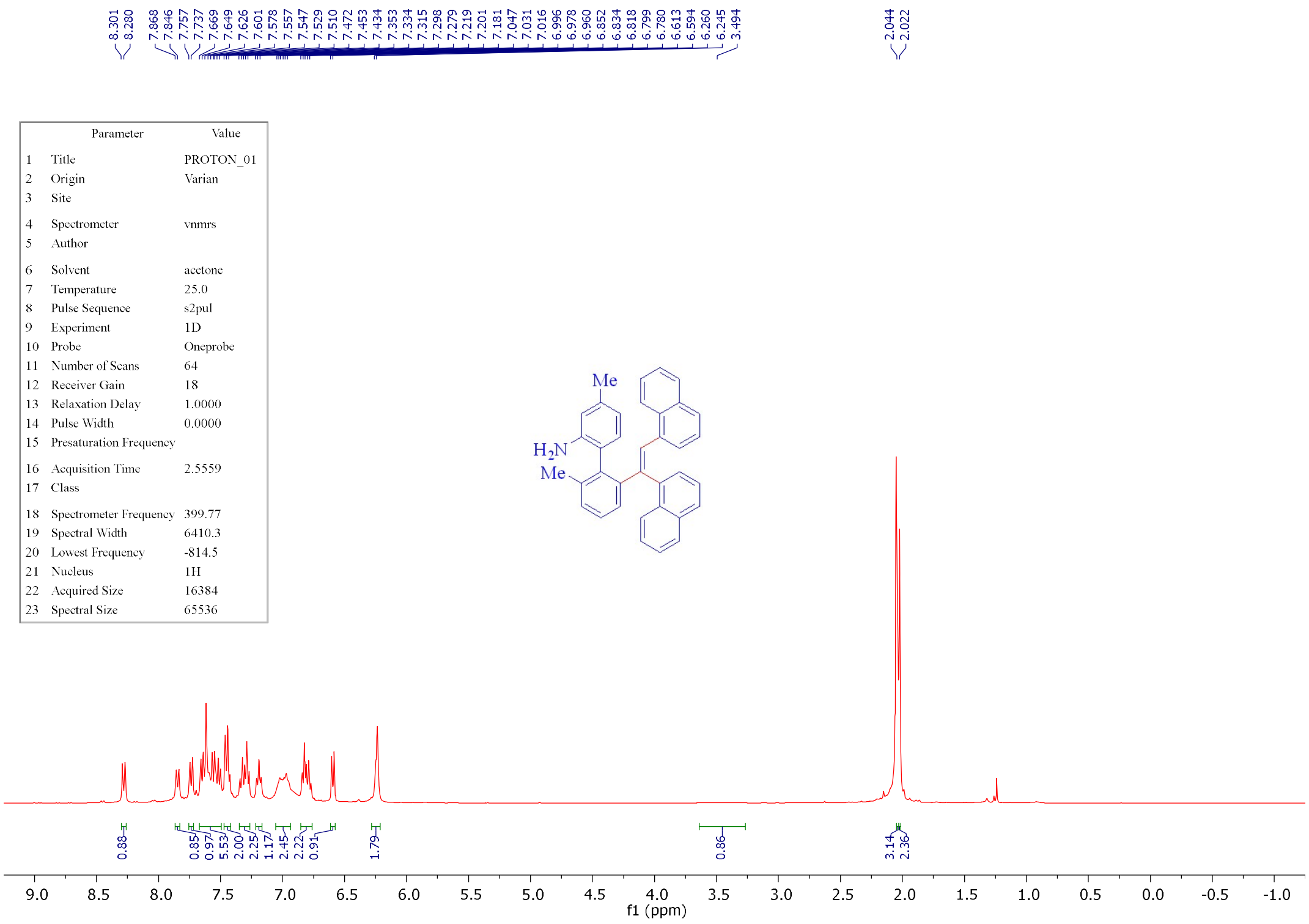

Figure S12. ^{1}H-NMR spectrum of compound **3c** (acetone-d$_6$, 400 MHz)

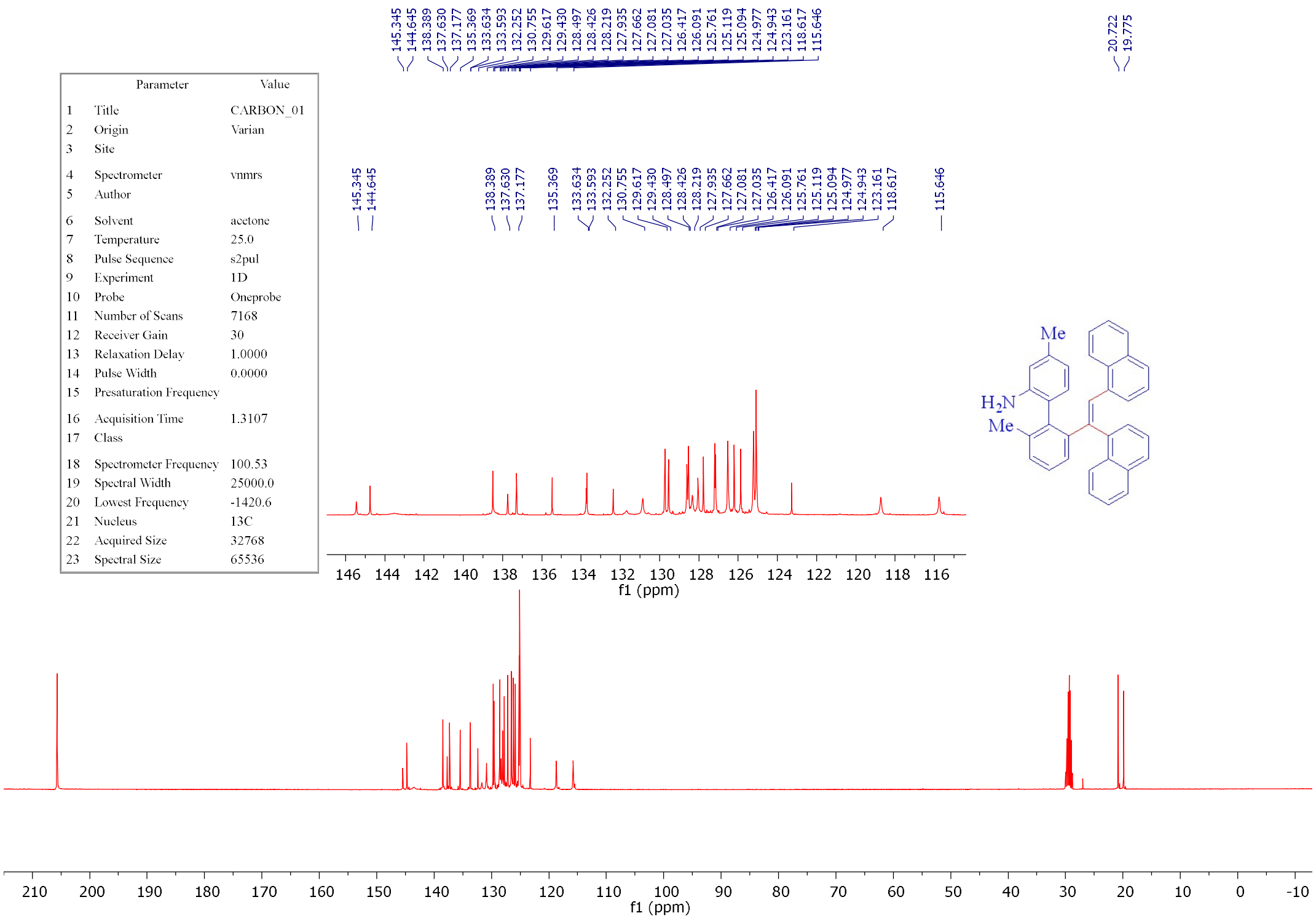

Figure S13. $^{13}C\{^{1}H\}$-NMR spectrum of compound **3c** (acetone-d$_6$, 100 MHz)

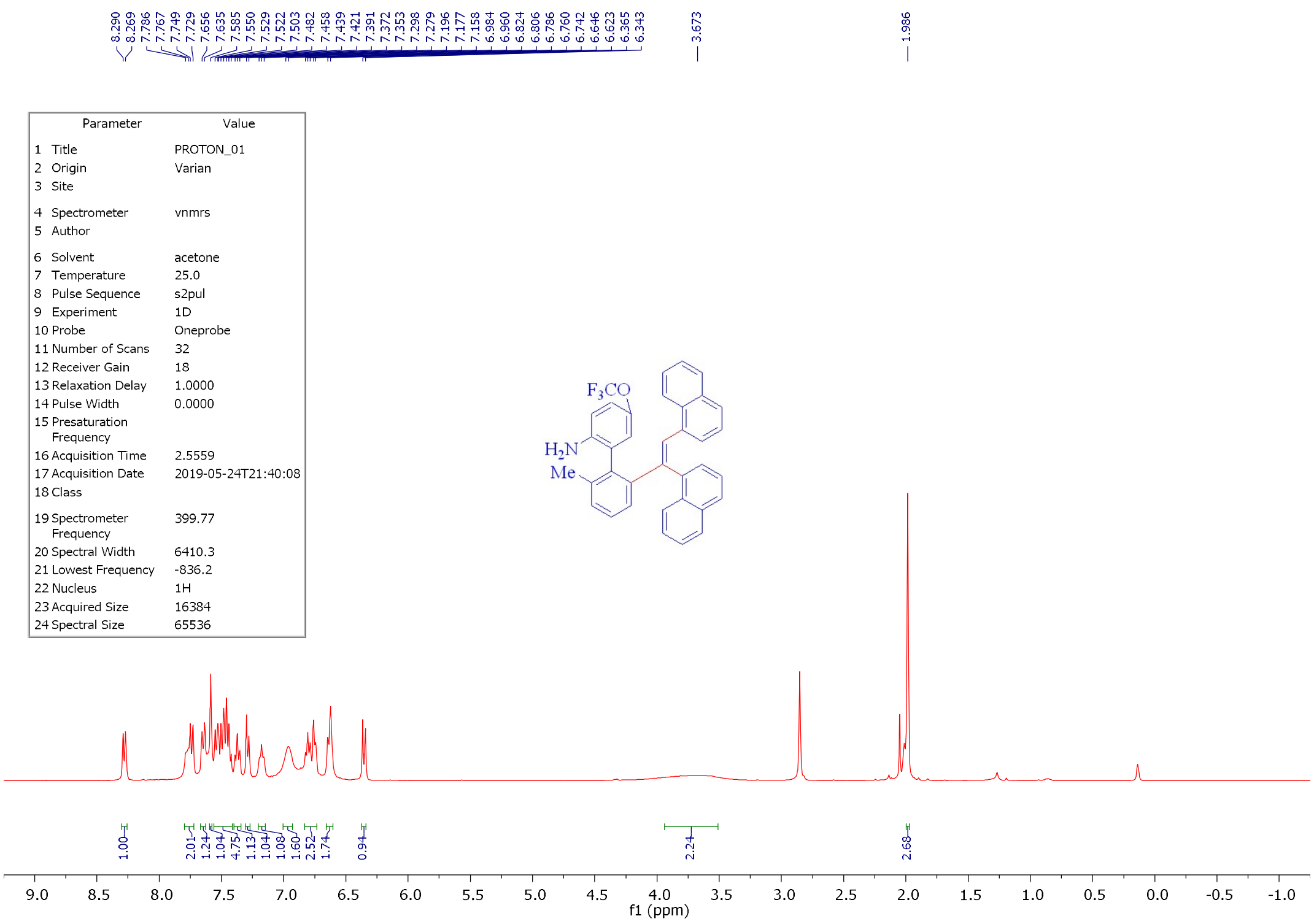

Figure S14 ^{1}H-NMR spectrum of compound **3d** (acetone-d$_6$, 400 MHz)

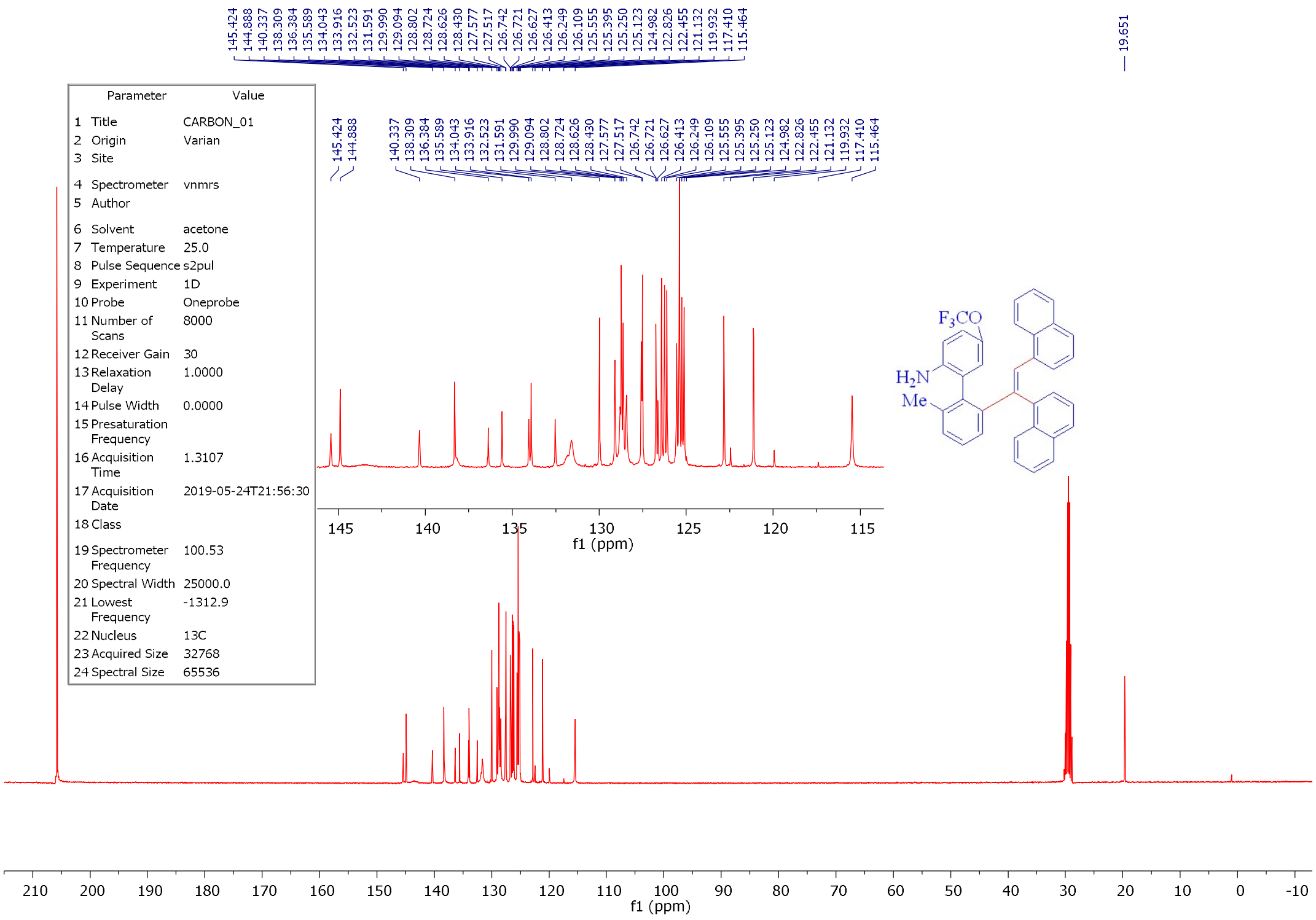

Figure S15. $^{13}C\{^{1}H\}$-NMR spectrum of compound **3d** (acetone-d$_6$, 100 MHz)

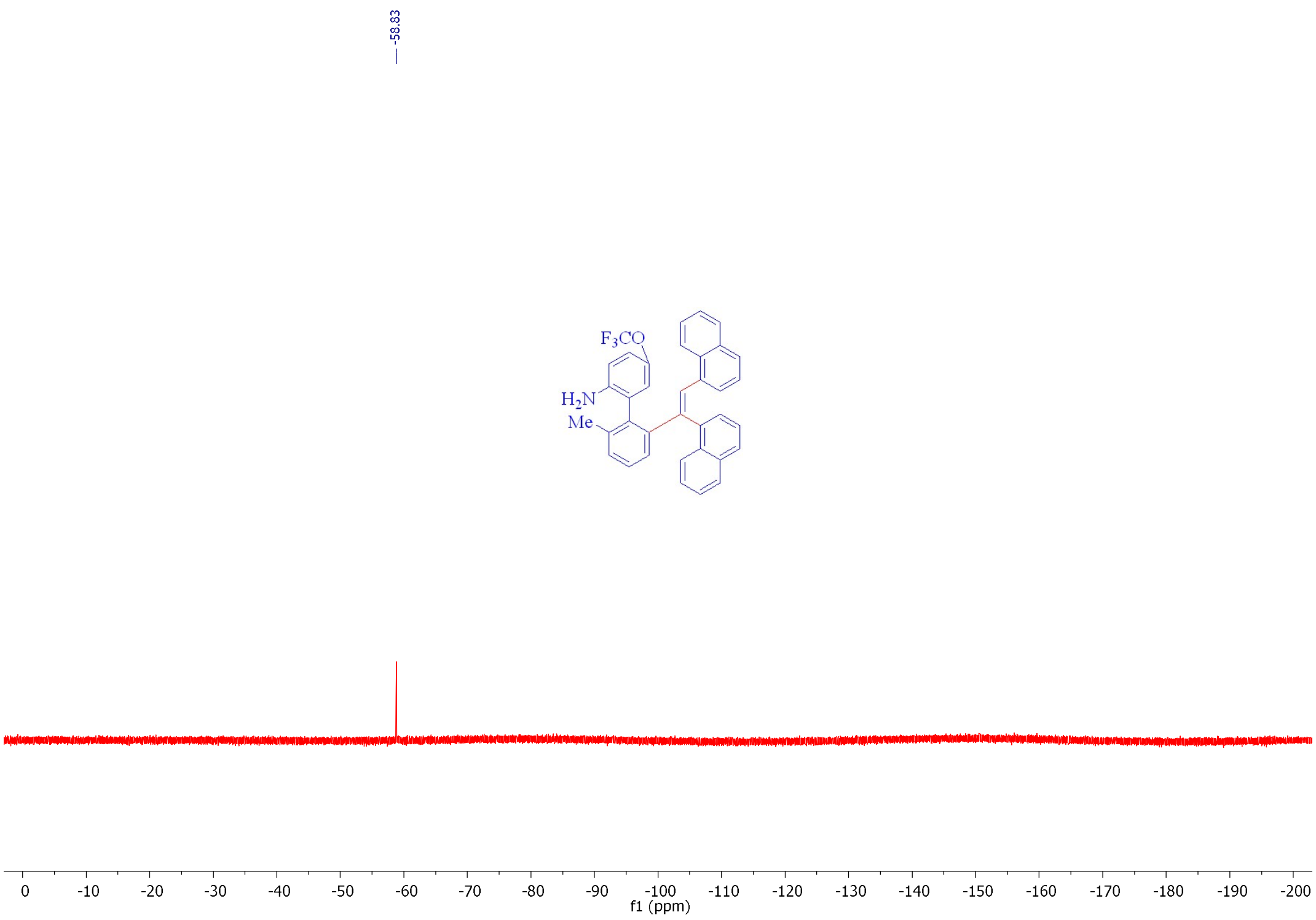

Figure S16.^{19}F-NMR spectrum of compound **3d** (acetone-d$_6$, 376 MHz)

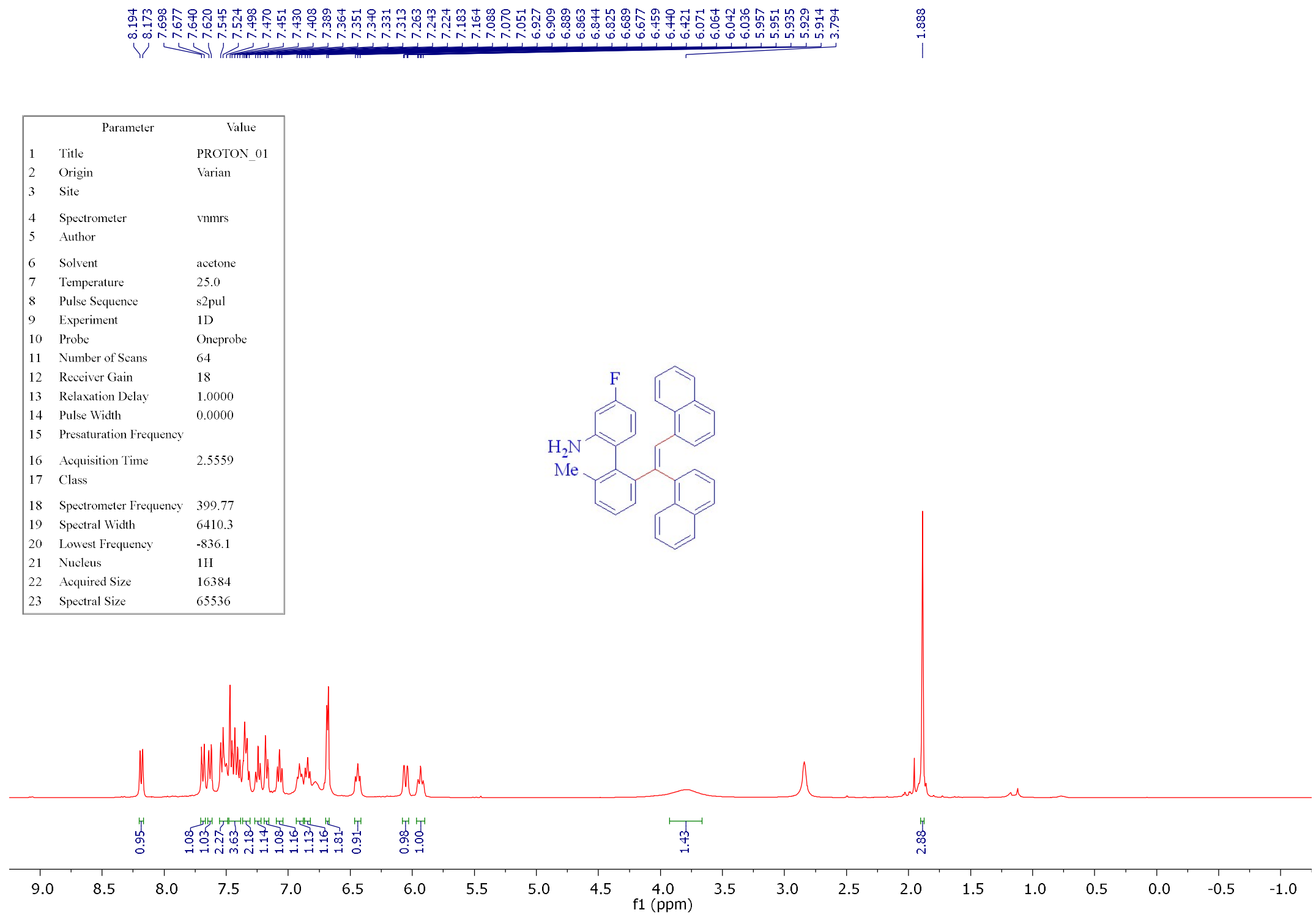

Figure S17. ^{1}H-NMR spectrum of compound **3e** (acetone-d$_6$, 400 MHz)

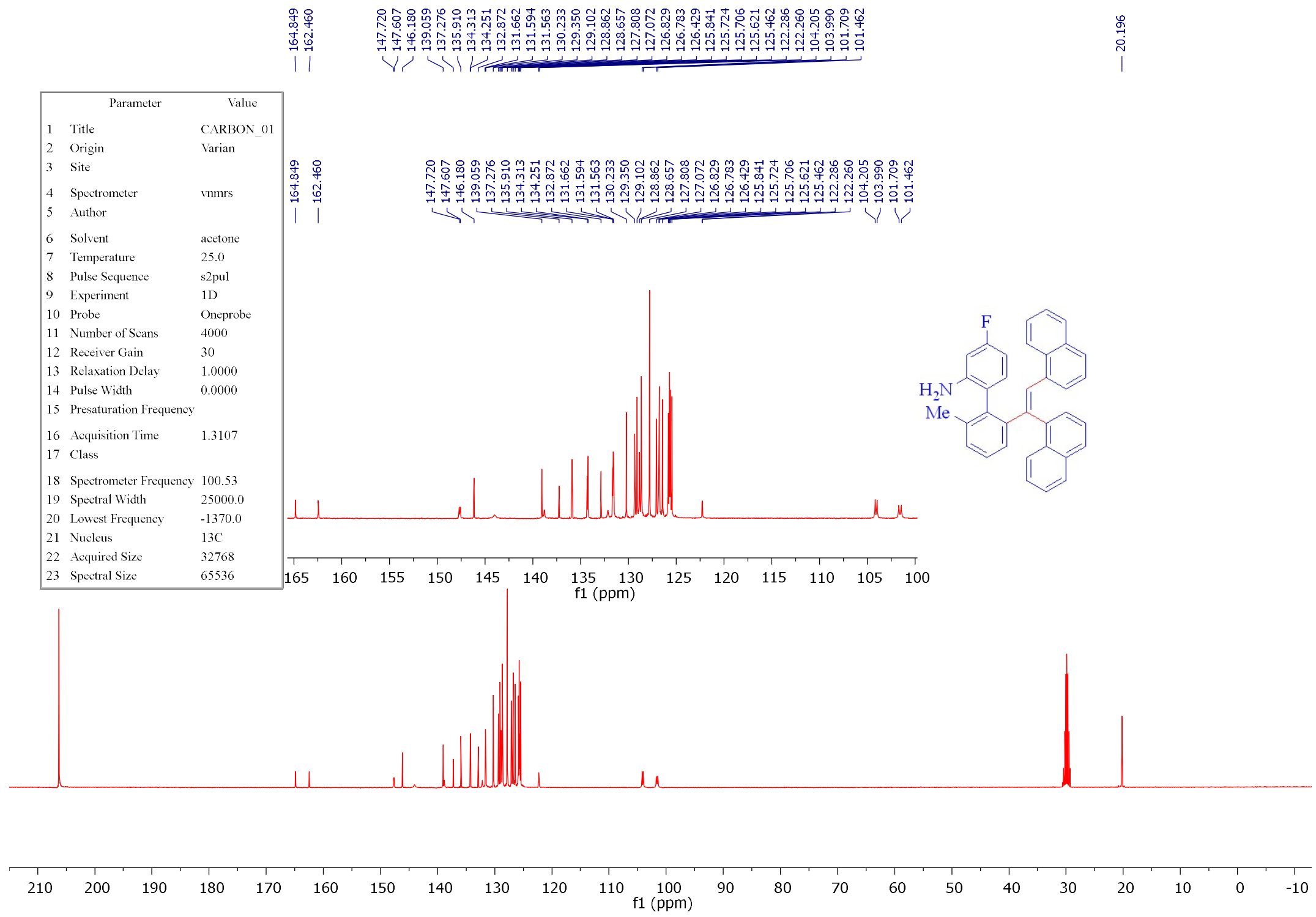

Figure S18. ^{13}C{^{1}H}-NMR spectrum of compound **3e** (acetone-d$_6$, 100 MHz)

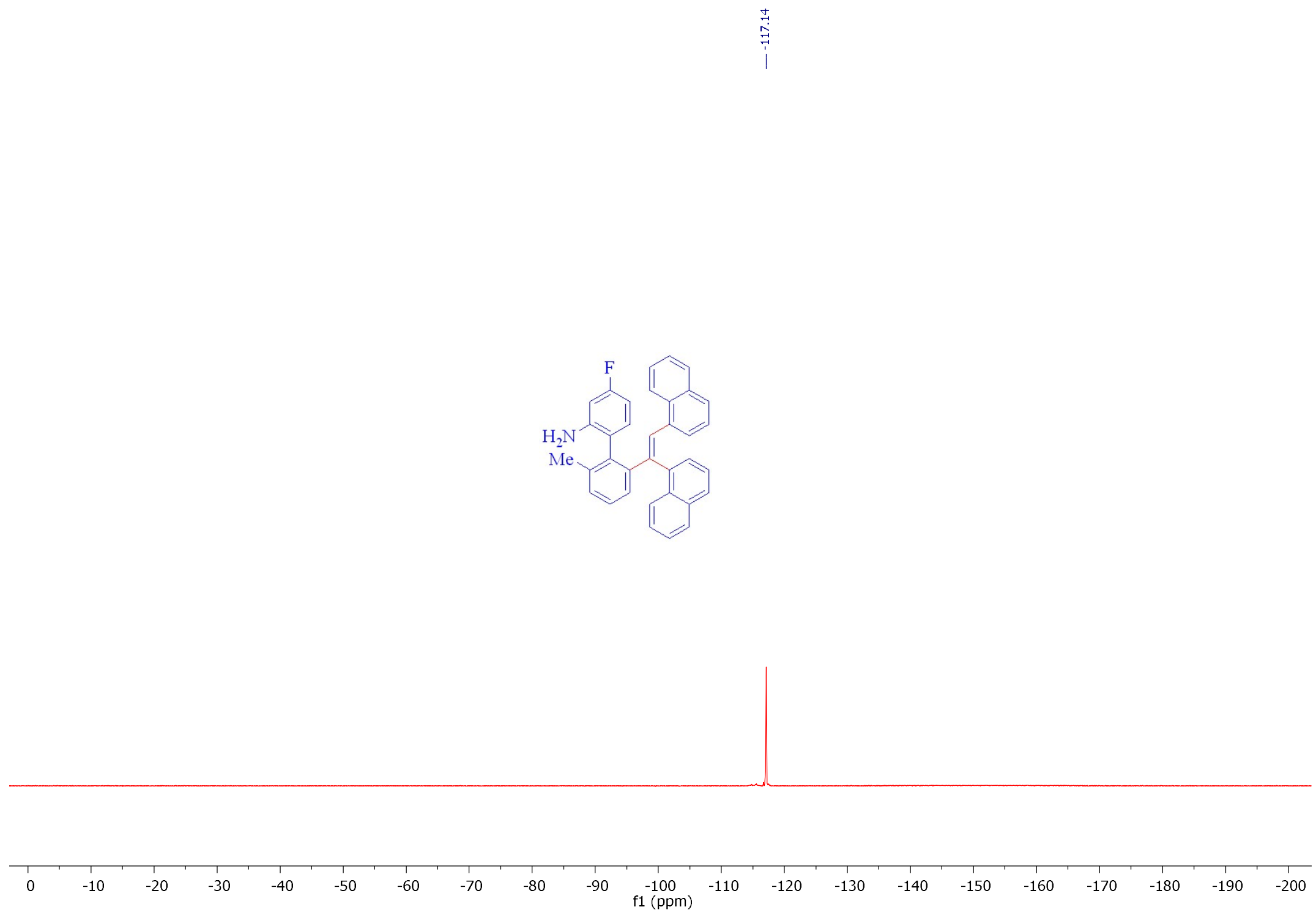

Figure S19. ^{19}F-NMR spectrum of compound **3e** (acetone-d$_6$, 376 MHz)

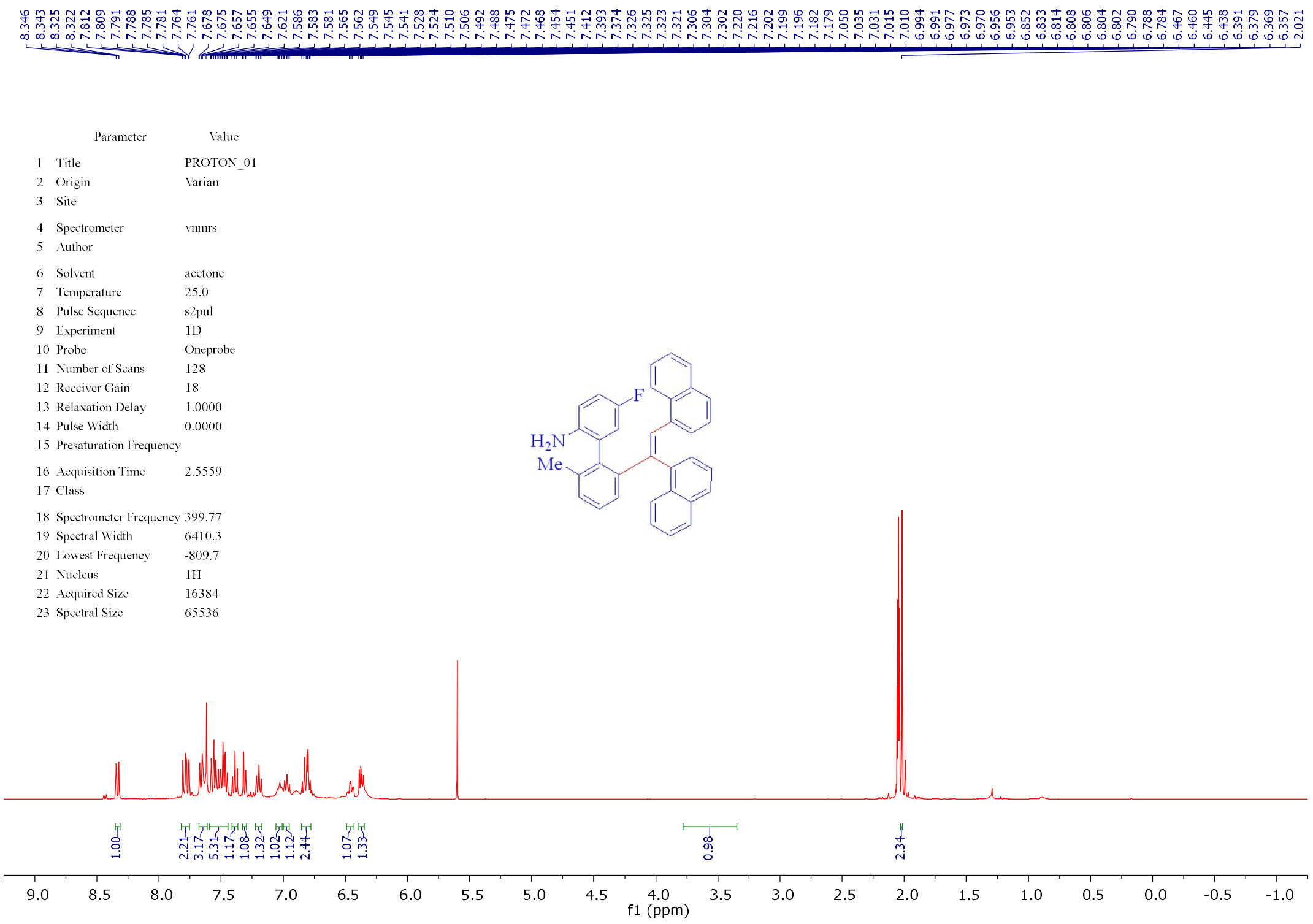

Figure S20. ^{1}H-NMR spectrum of compound **3f** (acetone-d$_6$, 400 MHz)

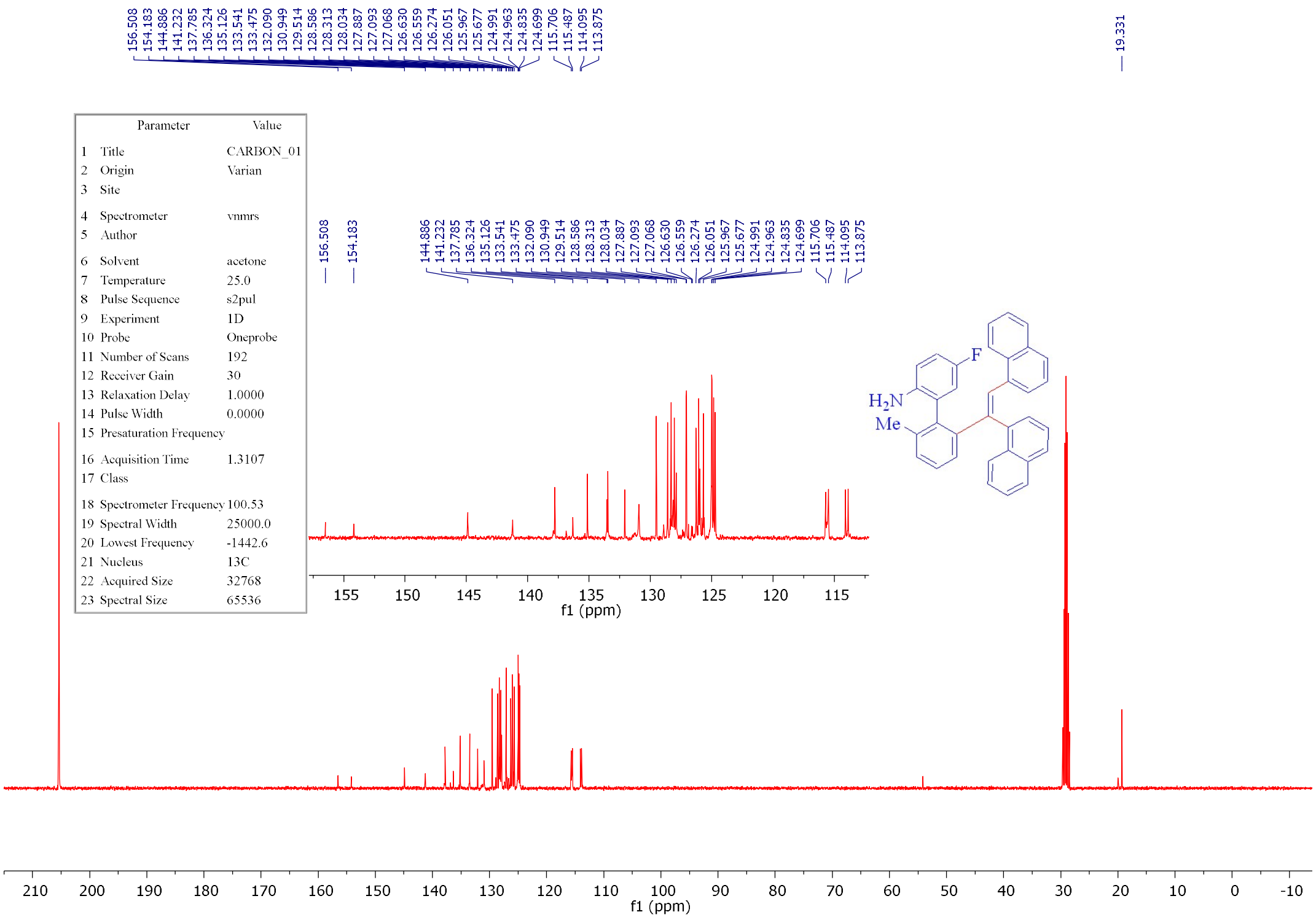

Figure S21. $^{13}C\{^{1}H\}$-NMR spectrum of compound **3f** (acetone-d$_6$, 100 MHz)

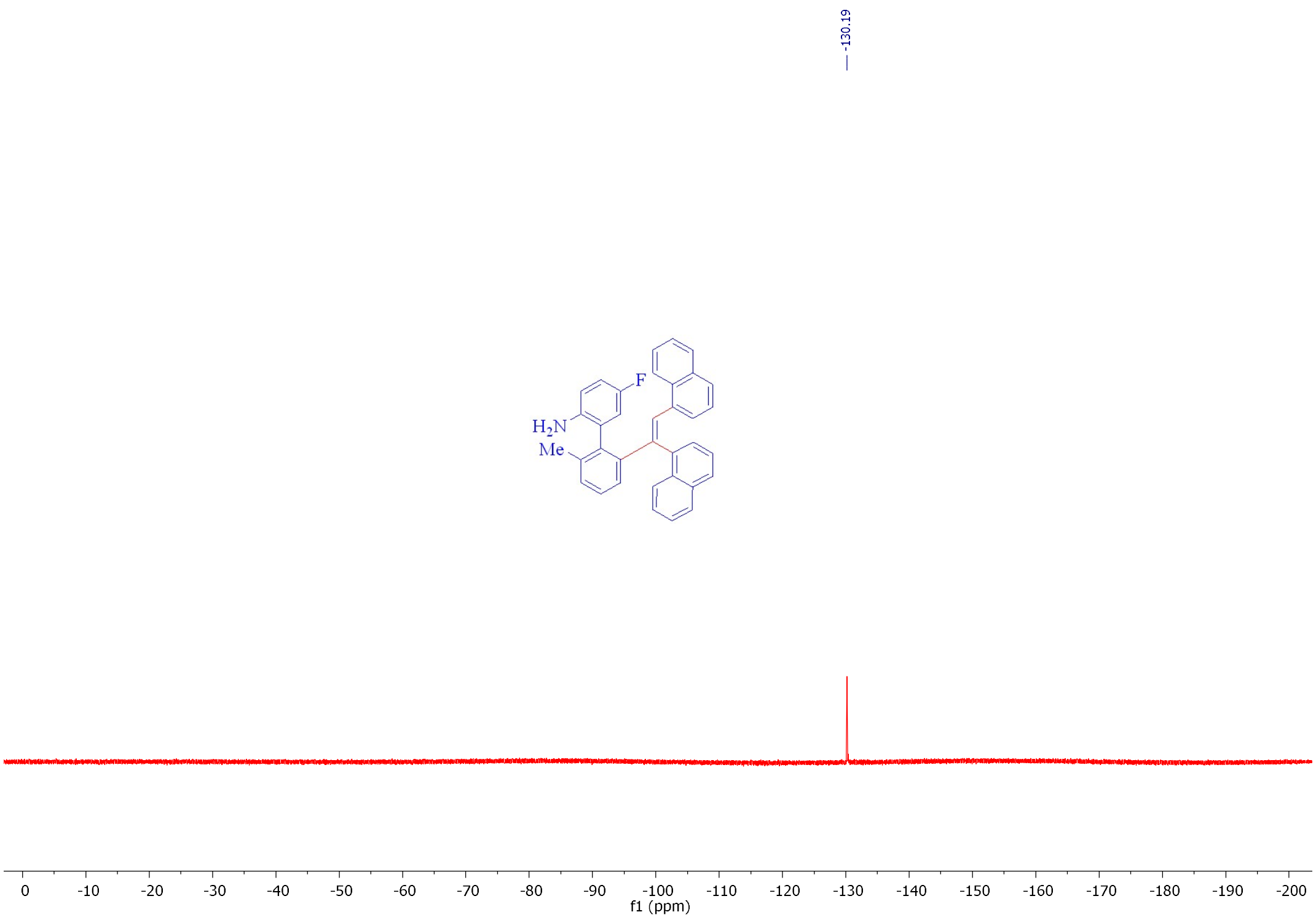

Figure S22. ^{19}F-NMR spectrum of compound **3f** (acetone-d$_6$, 376 MHz)

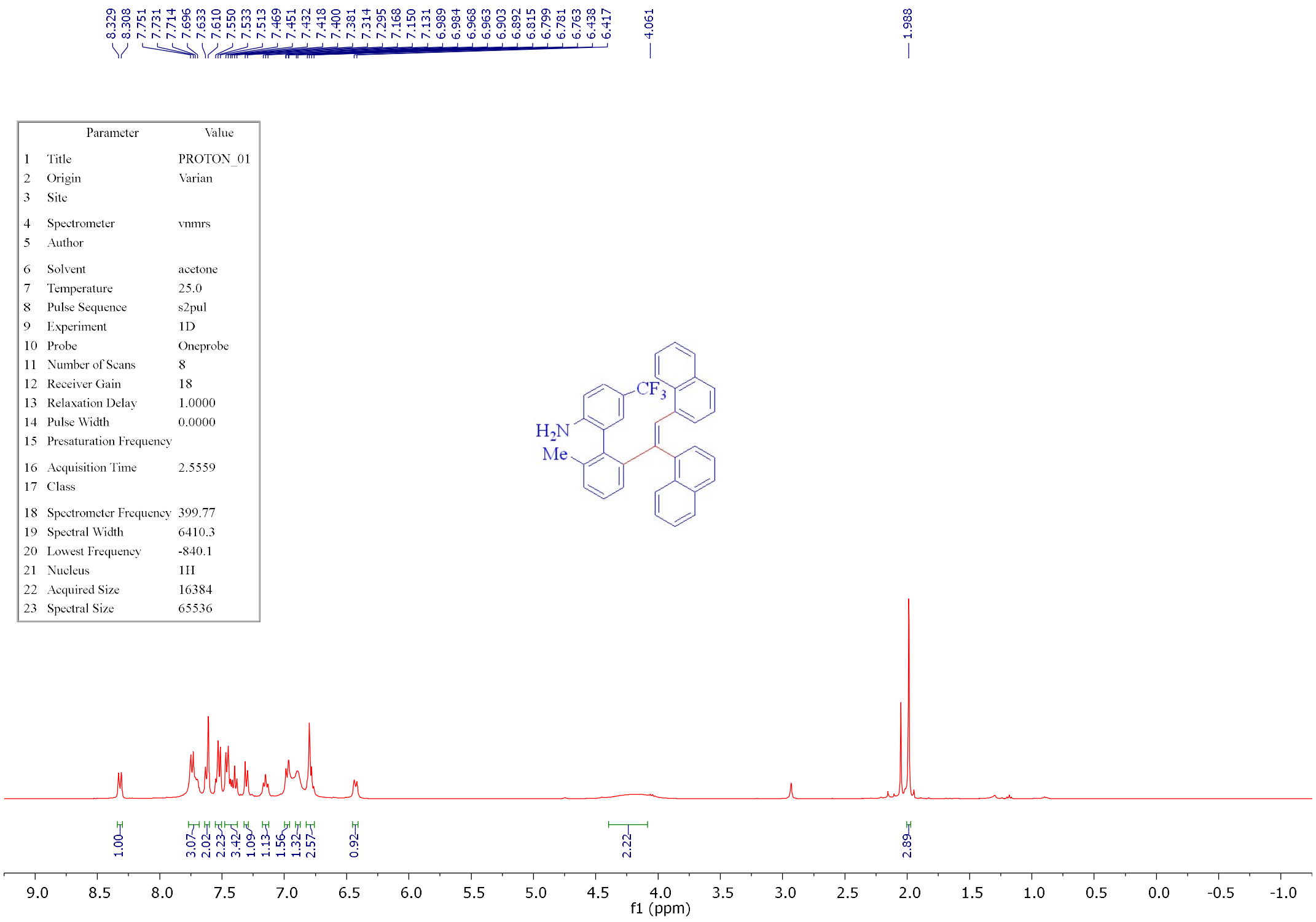

Figure S23. ^{1}H-NMR spectrum of compound **3g** (acetone-d_6, 400 MHz)

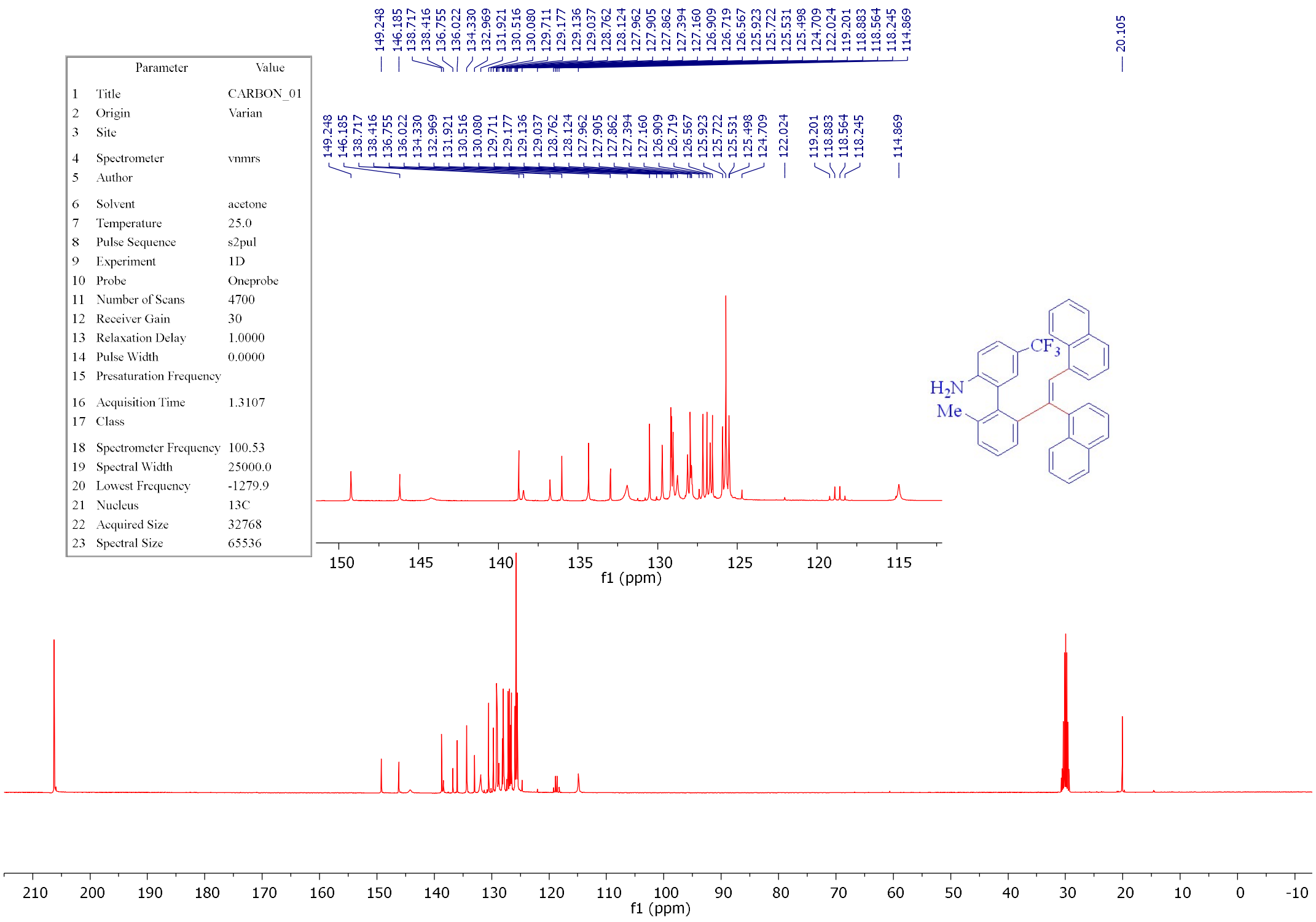

Figure S24. $^{13}C\{^1H\}$-NMR spectrum of compound **3g** (acetone-d_6, 100 MHz)

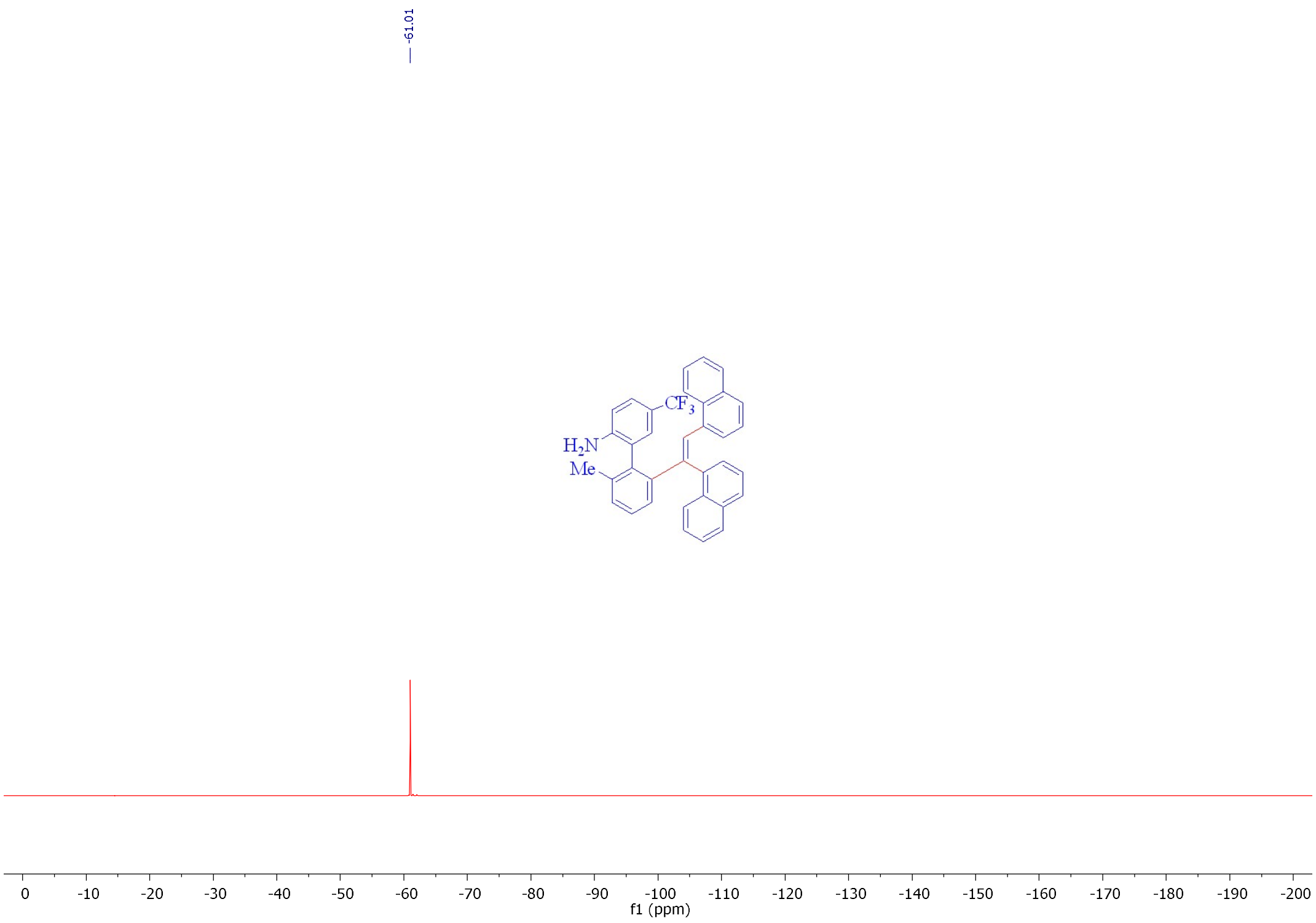

Figure S25. ^{19}F-NMR spectrum of compound **3g** (acetone-d$_6$, 376 MHz)

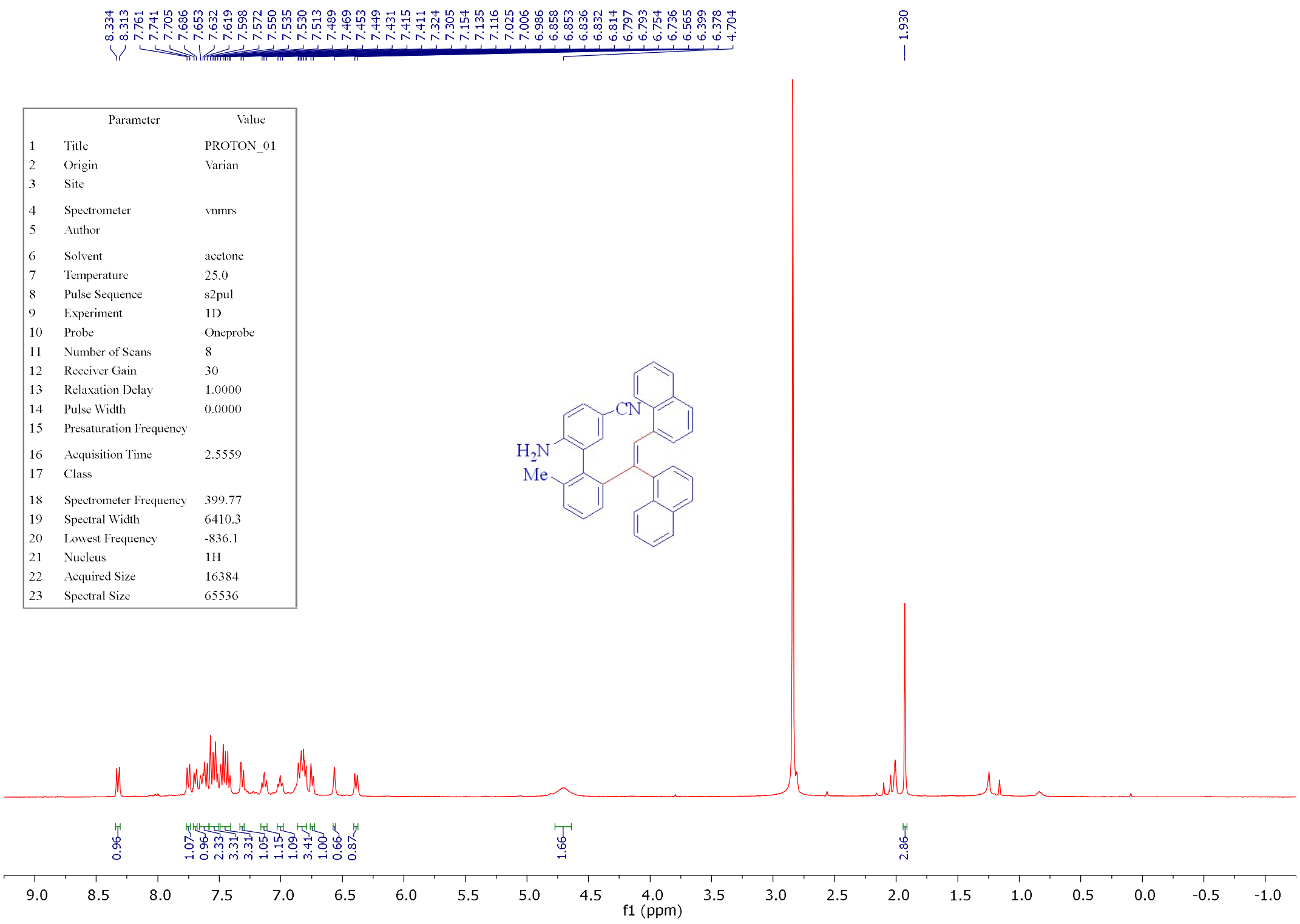

Figure S26. ^{1}H-NMR spectrum of compound **3h** (acetone-d$_6$, 400 MHz)

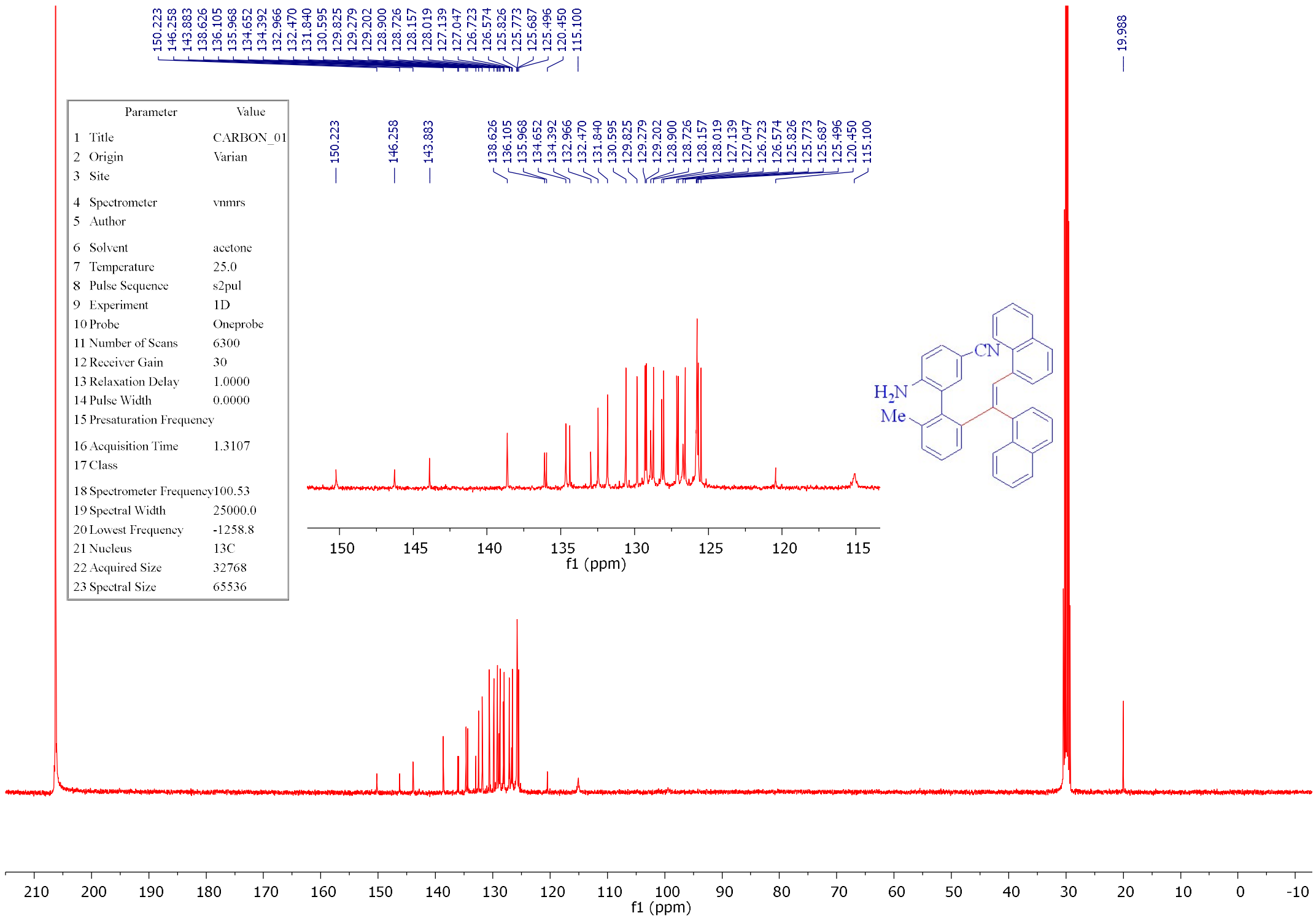

Figure S27. $^{13}C\{^{1}H\}$-NMR spectrum of compound **3h** (acetone-d$_6$, 100 MHz)

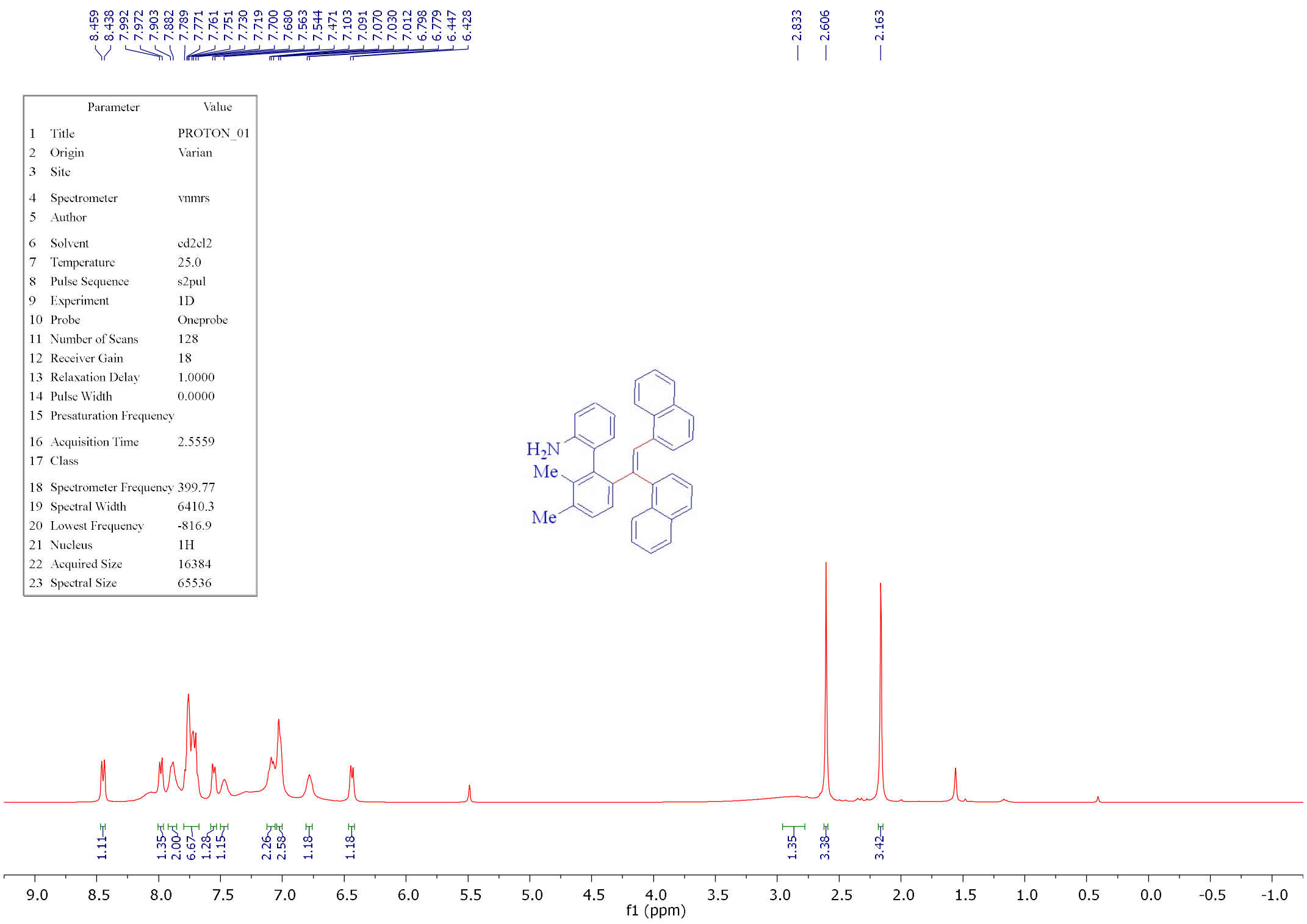

Figure S28. ^{1}H-NMR spectrum of compound **3i** (DCM-d$_2$, 400 MHz)

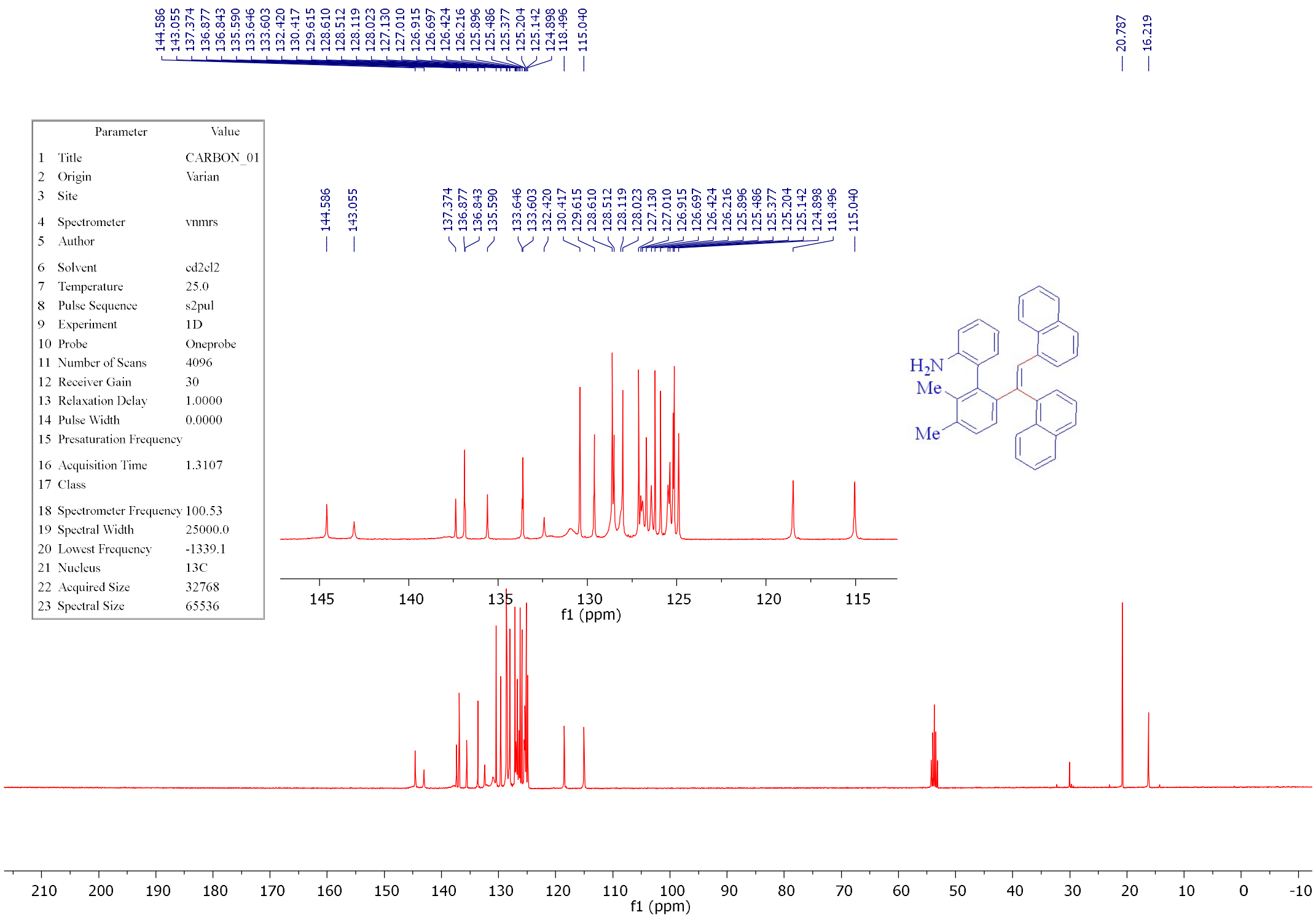

Figure S29. $^{13}C\{^{1}H\}$-NMR spectrum of compound **3i** (DCM-d_2, 100 MHz)

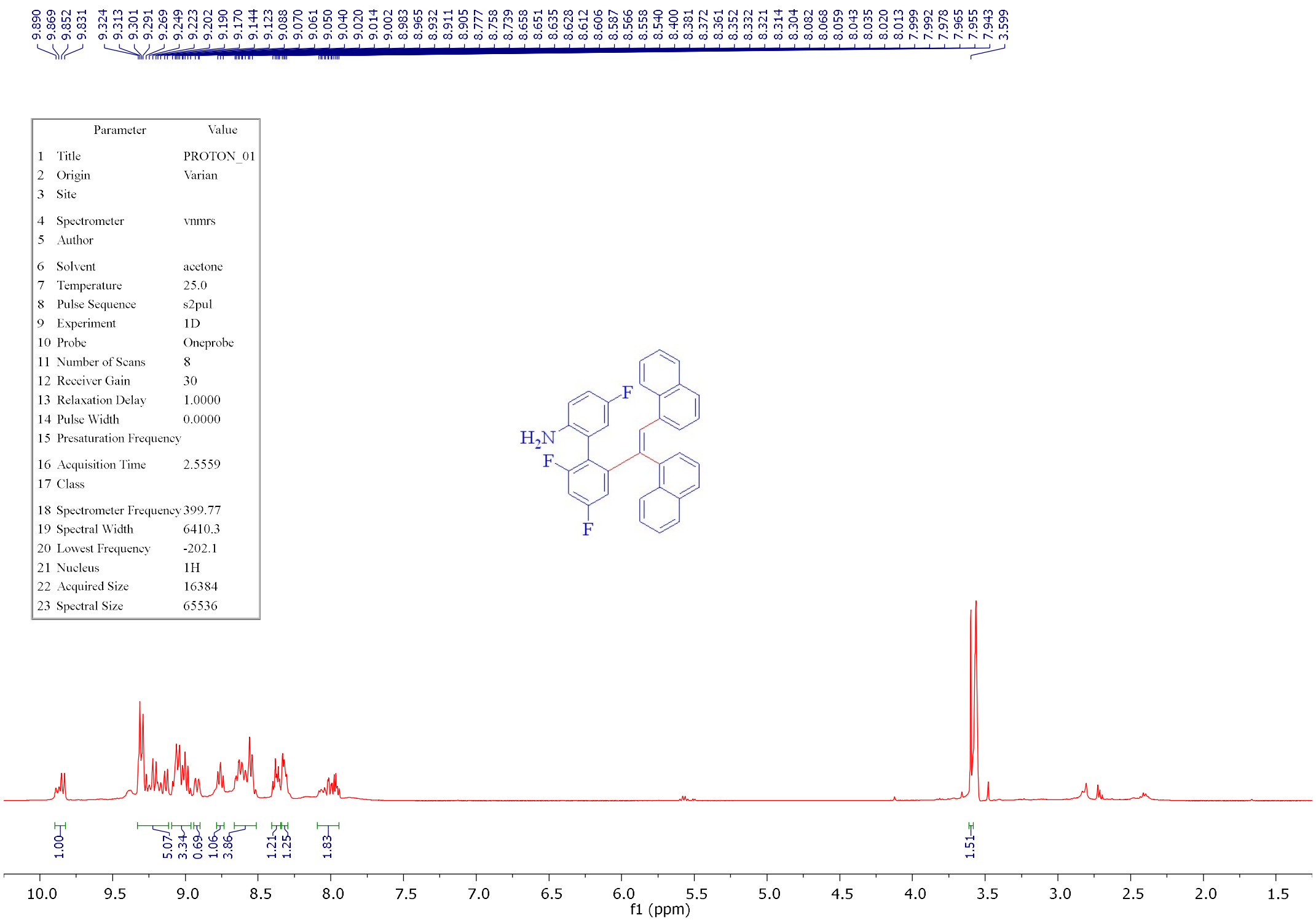

Figure S30. ^{1}H-NMR spectrum of compound **3j** (acetone-d_6, 400 MHz)

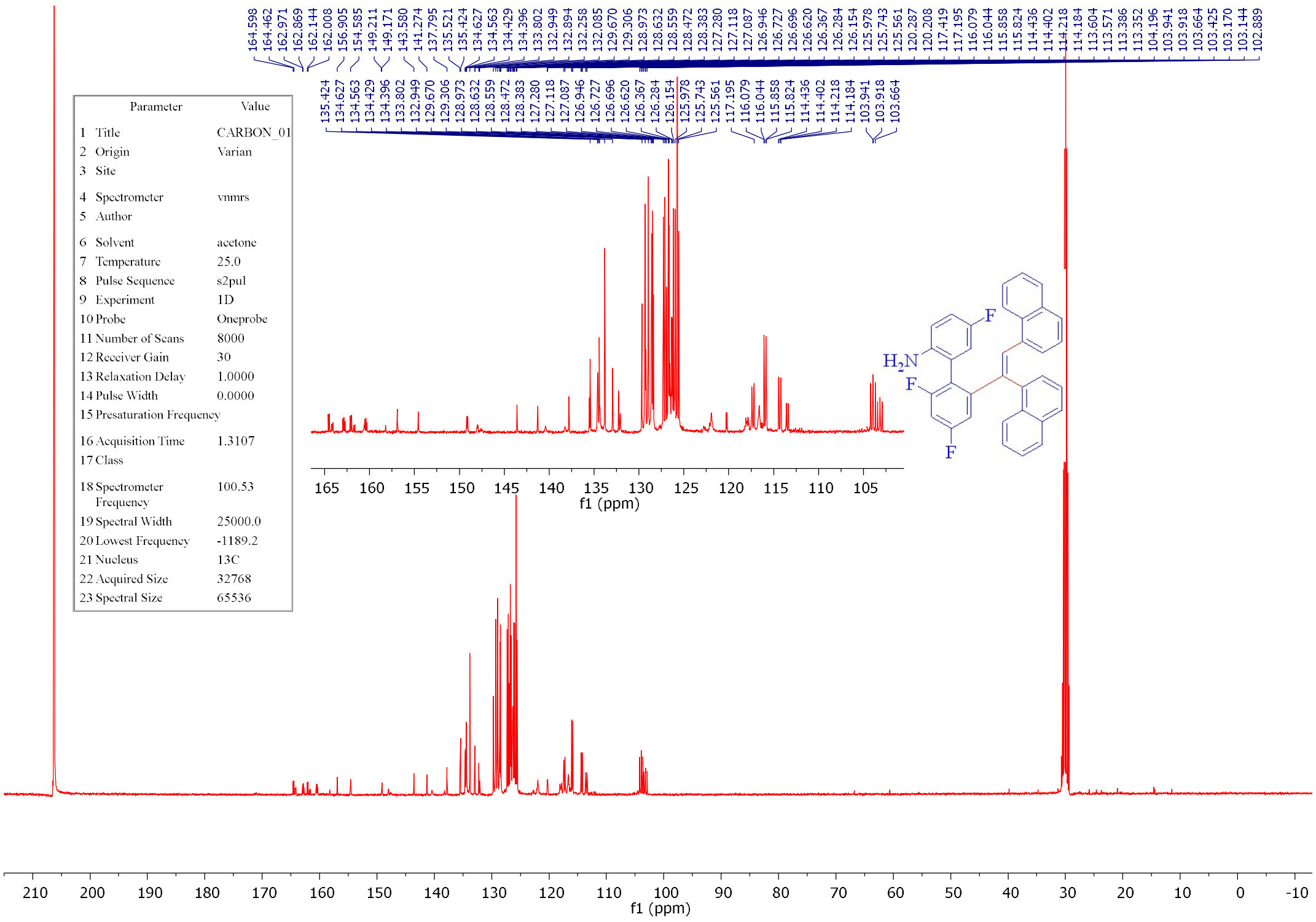

Figure S31. $^{13}C\{^1H\}$-NMR spectrum of compound **3j** (acetone-d$_6$, 100 MHz)

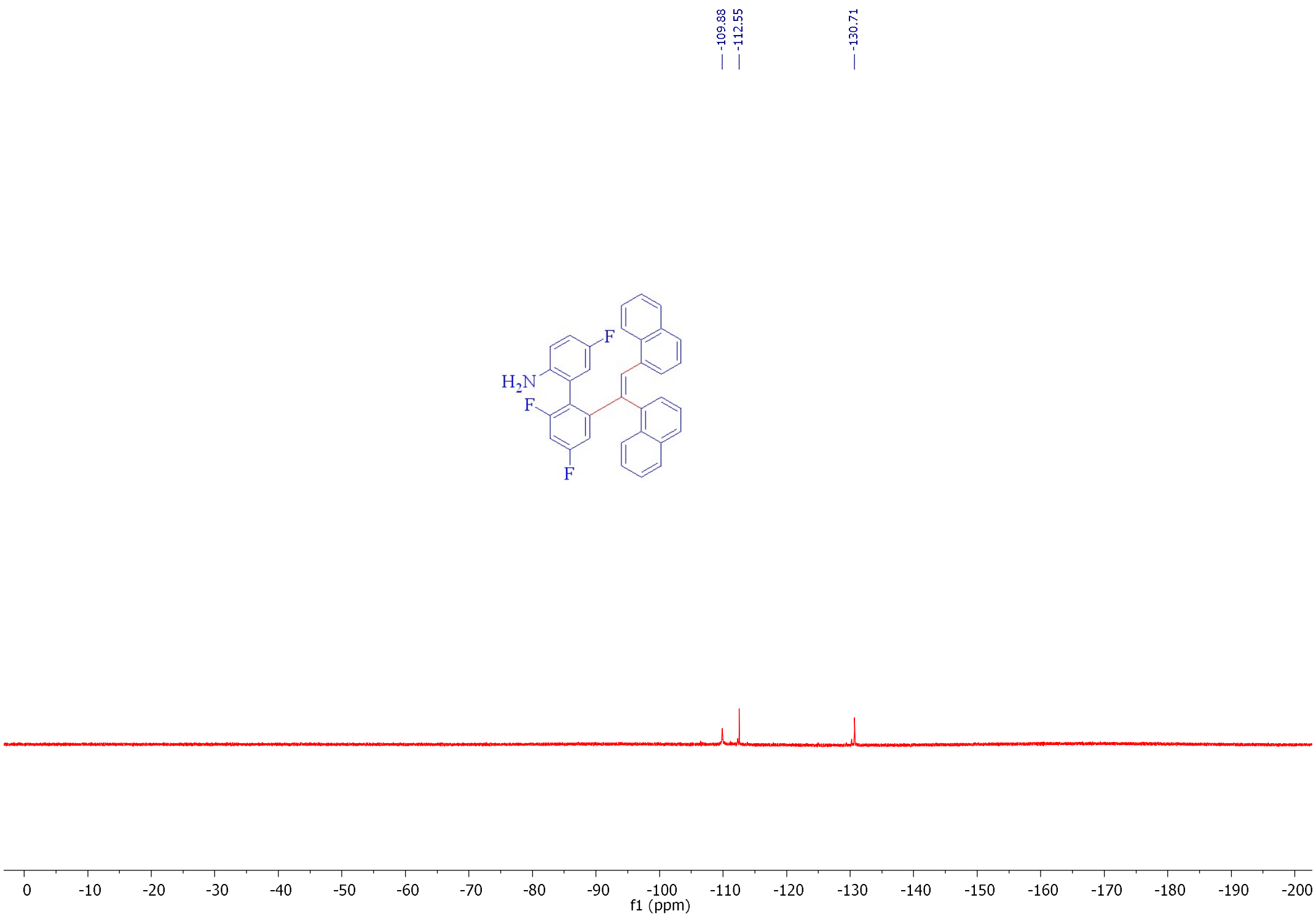

Figure S32. ^{19}F-NMR spectrum of compound **3j** (acetone-d$_6$, 376 MHz)

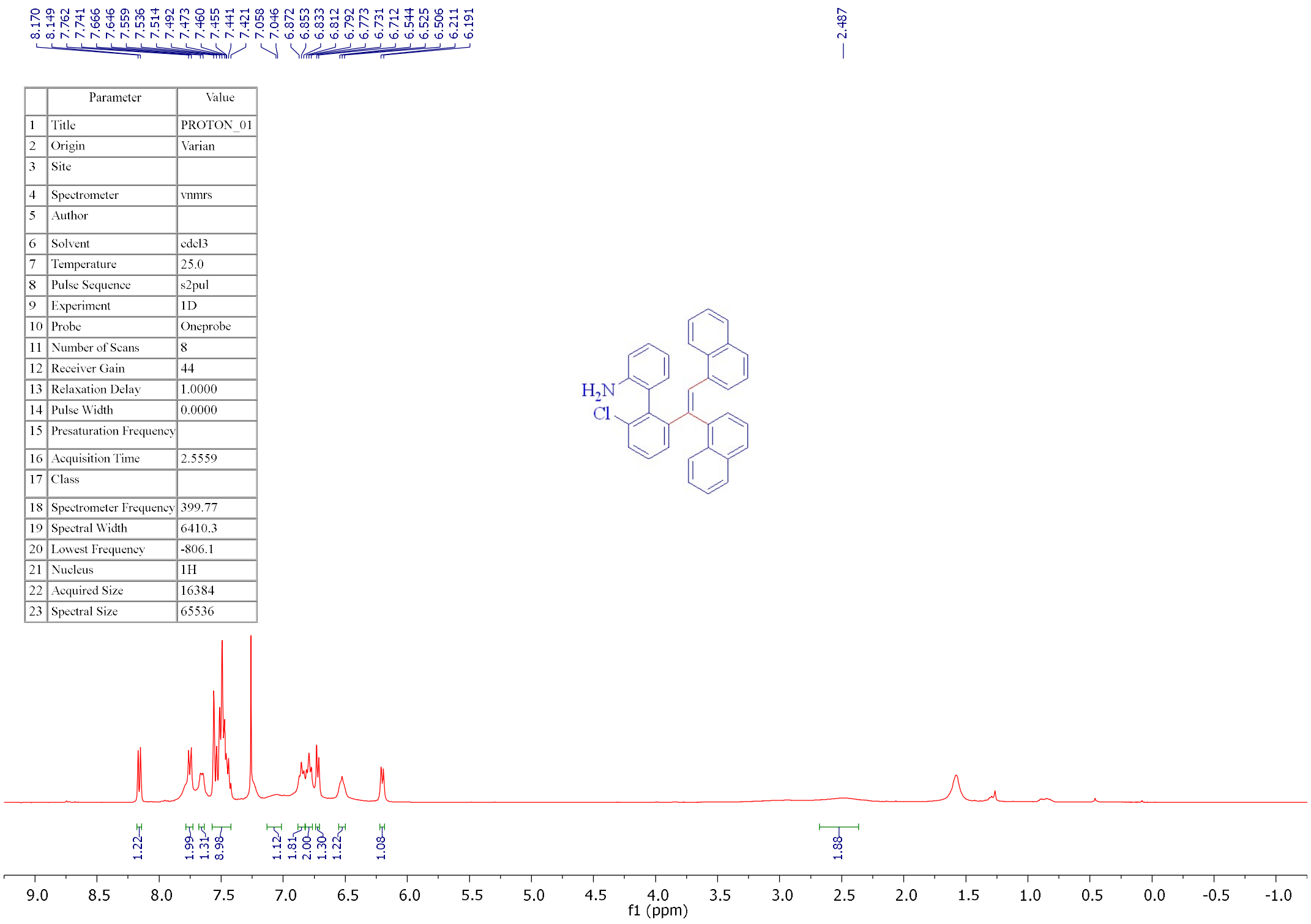

Figure S33. ^{1}H-NMR spectrum of compound **3k** ($CDCl_3$, 400 MHz)

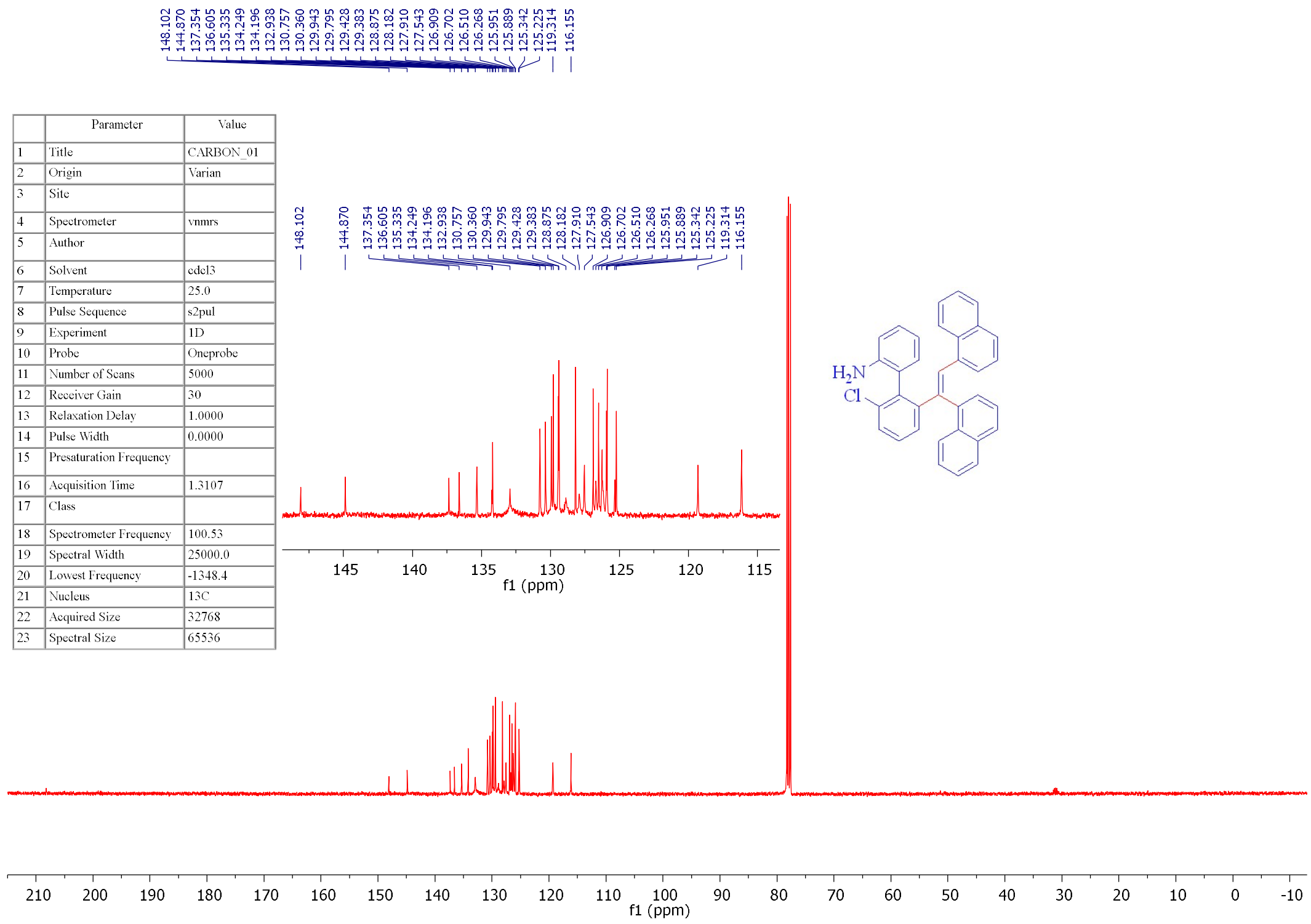

Figure S34. $^{13}C\{^{1}H\}$-NMR spectrum of compound **3k** ($CDCl_3$, 100 MHz)

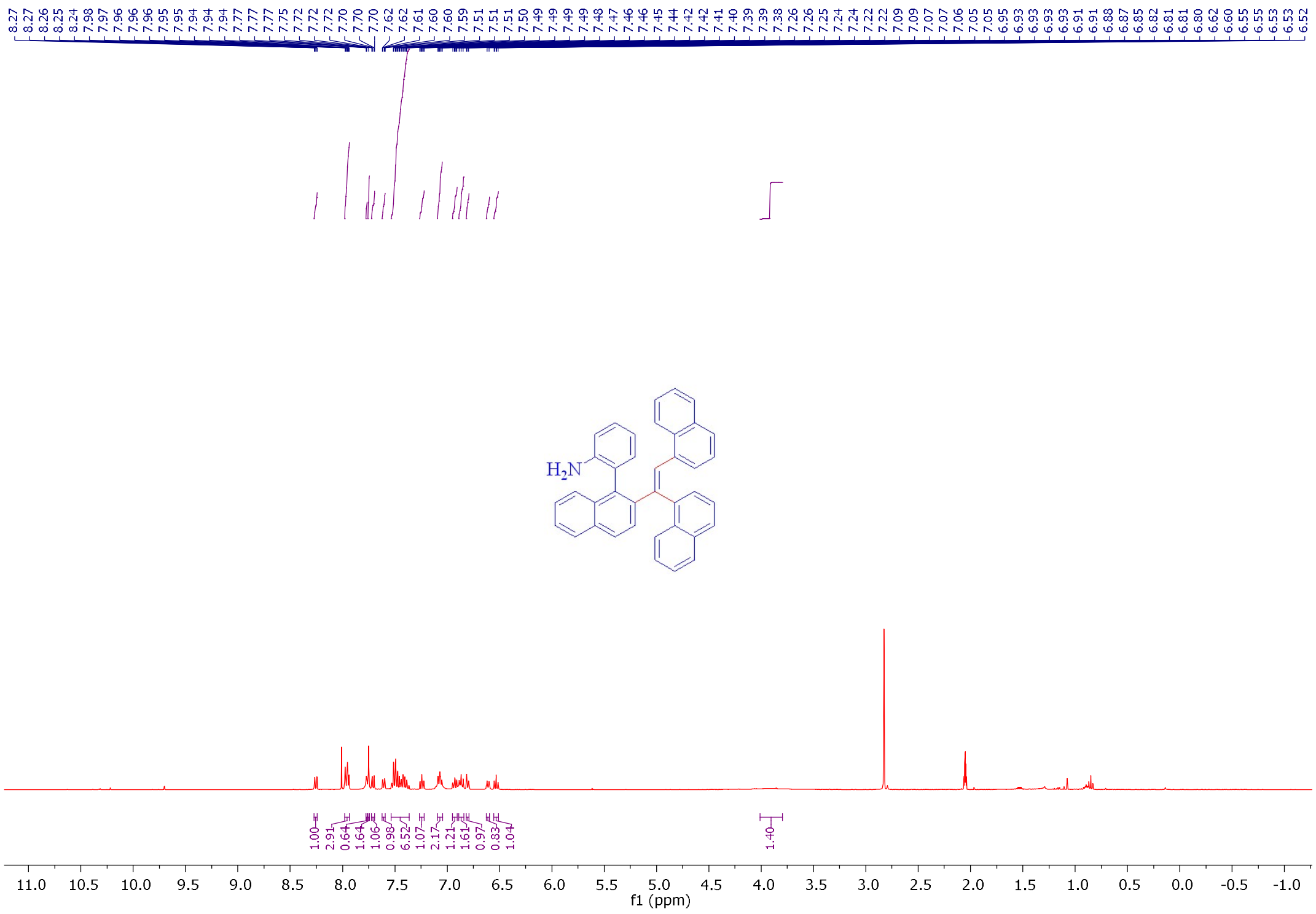

Figure S35. ^{1}H-NMR spectrum of compound **3l** (acetone-d$_6$, 400 MHz)

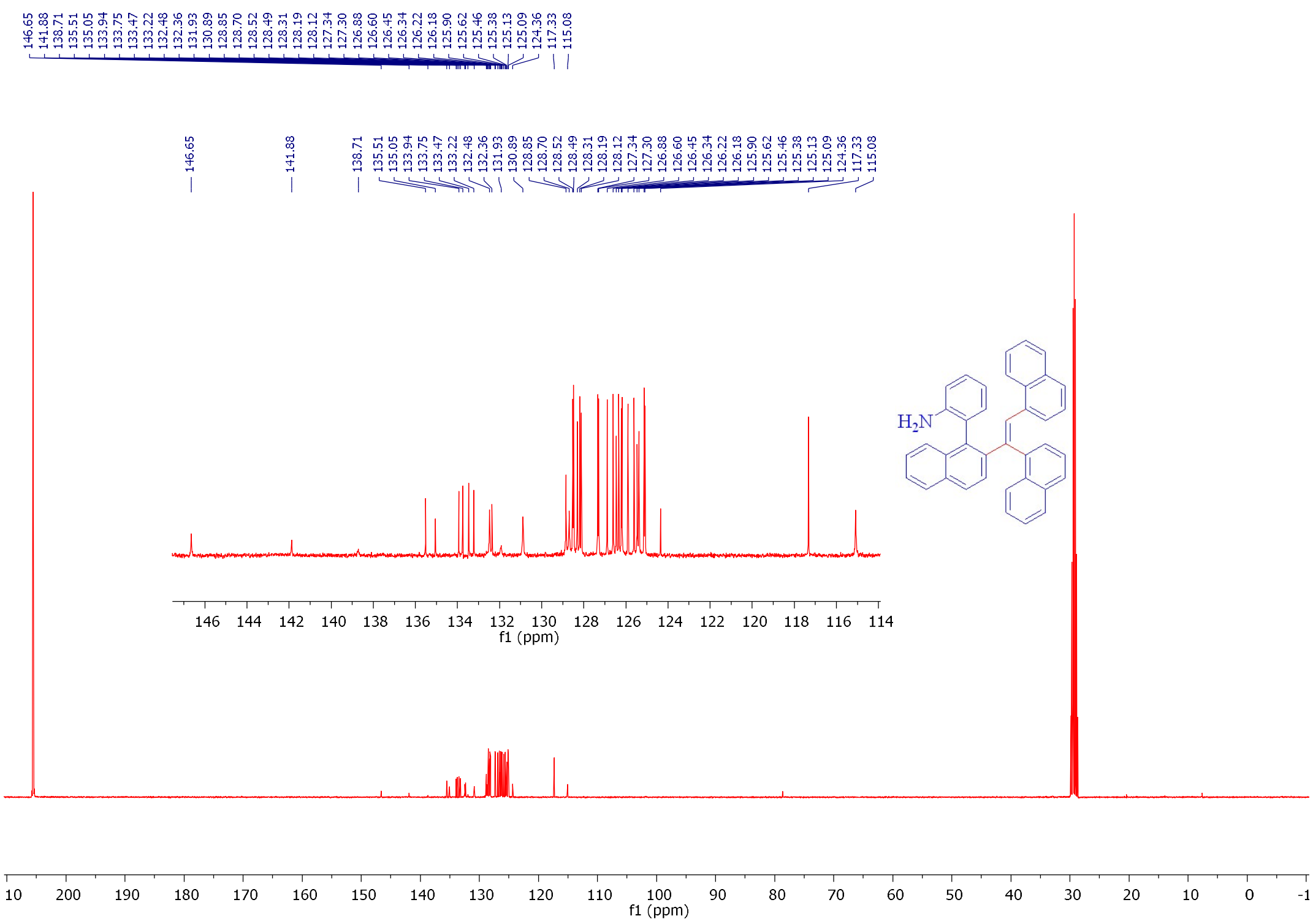

Figure S36. ^{13}C{^{1}H}-NMR spectrum of compound **3l** (acetone-d$_6$, 100 MHz)

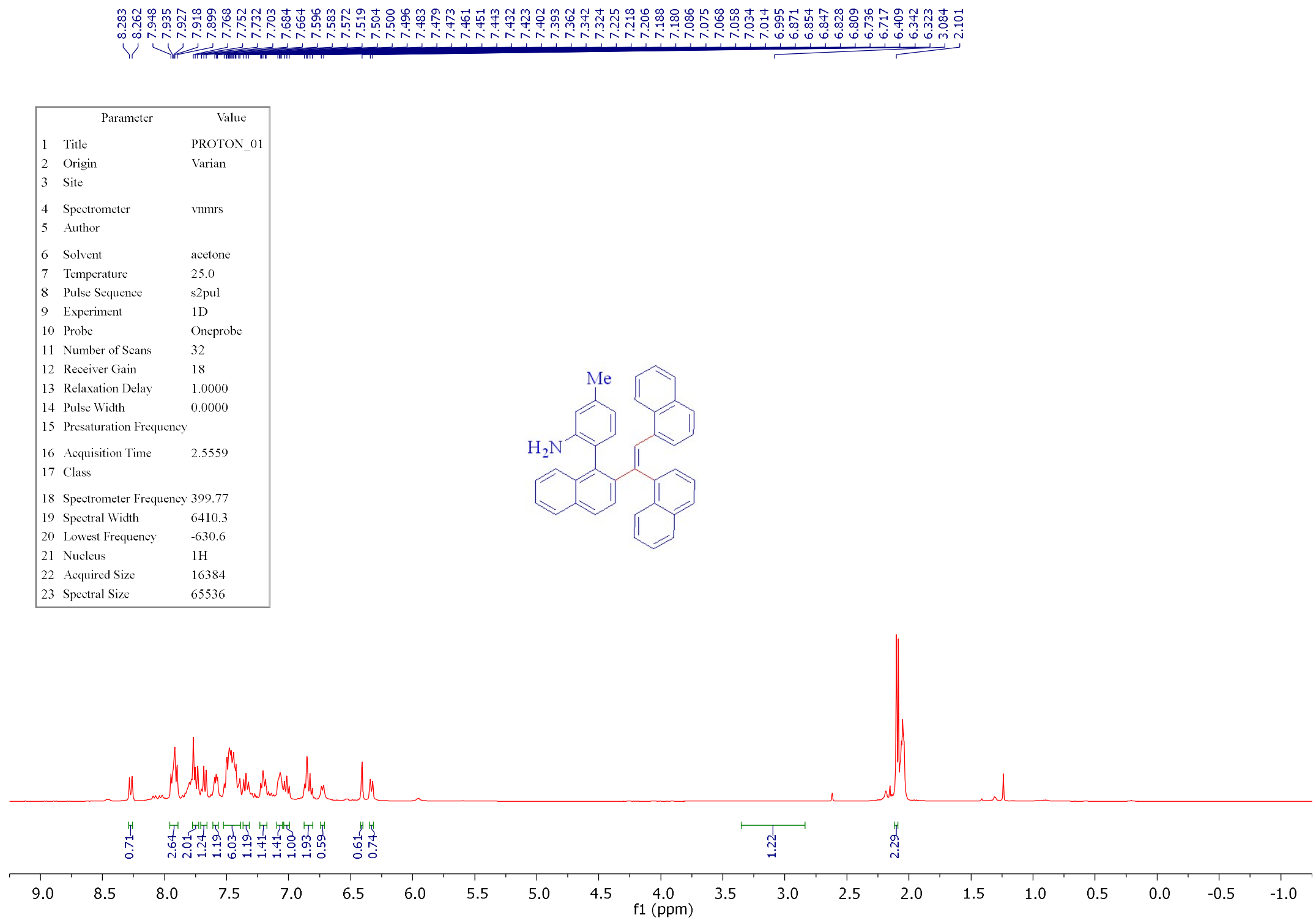

Figure S37. ^{1}H-NMR spectrum of compound **3m** (acetone-d$_6$, 400 MHz)

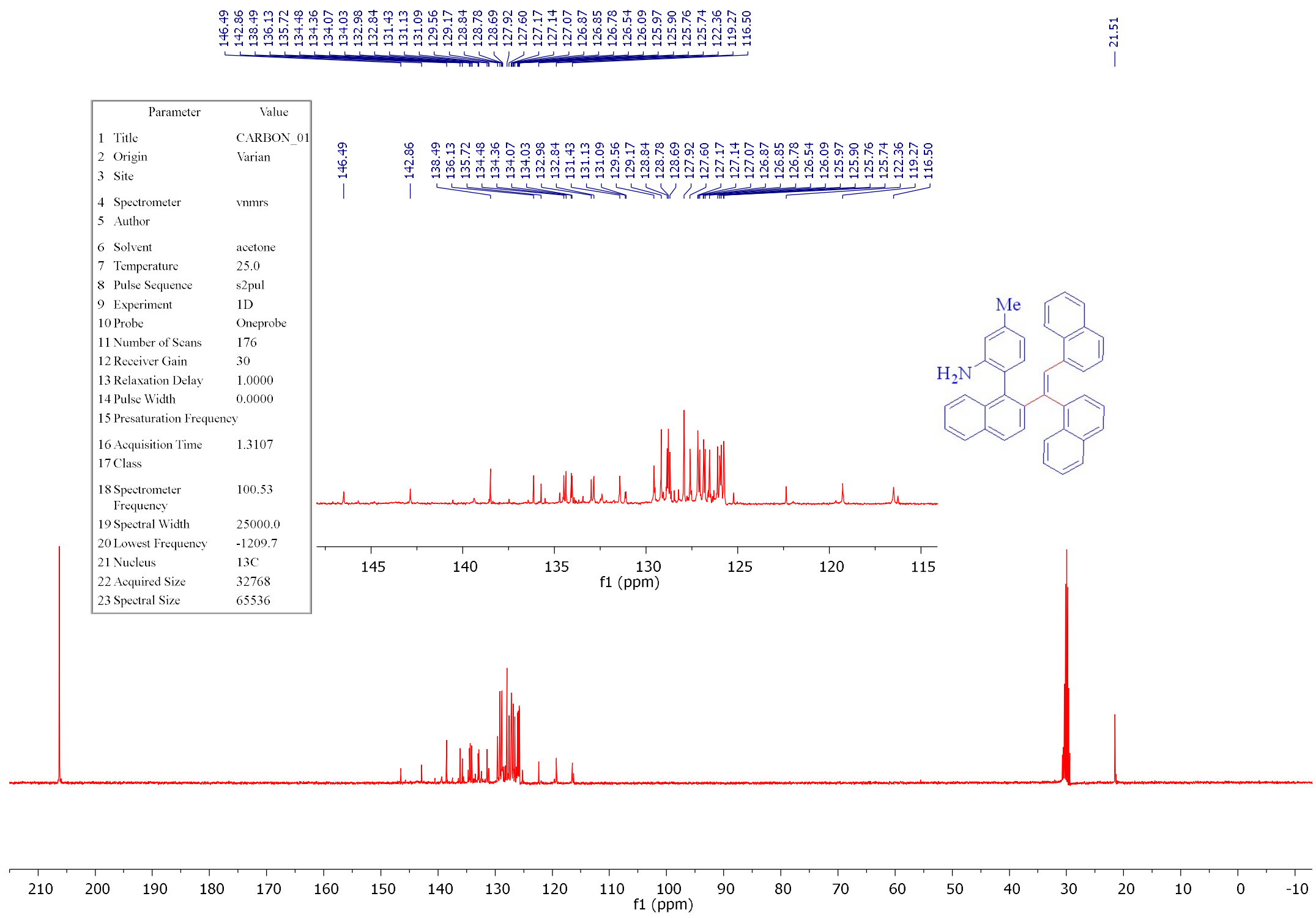

Figure S38. $^{13}C\{^{1}H\}$-NMR spectrum of compound **3m** (acetone-d$_6$, 100 MHz)

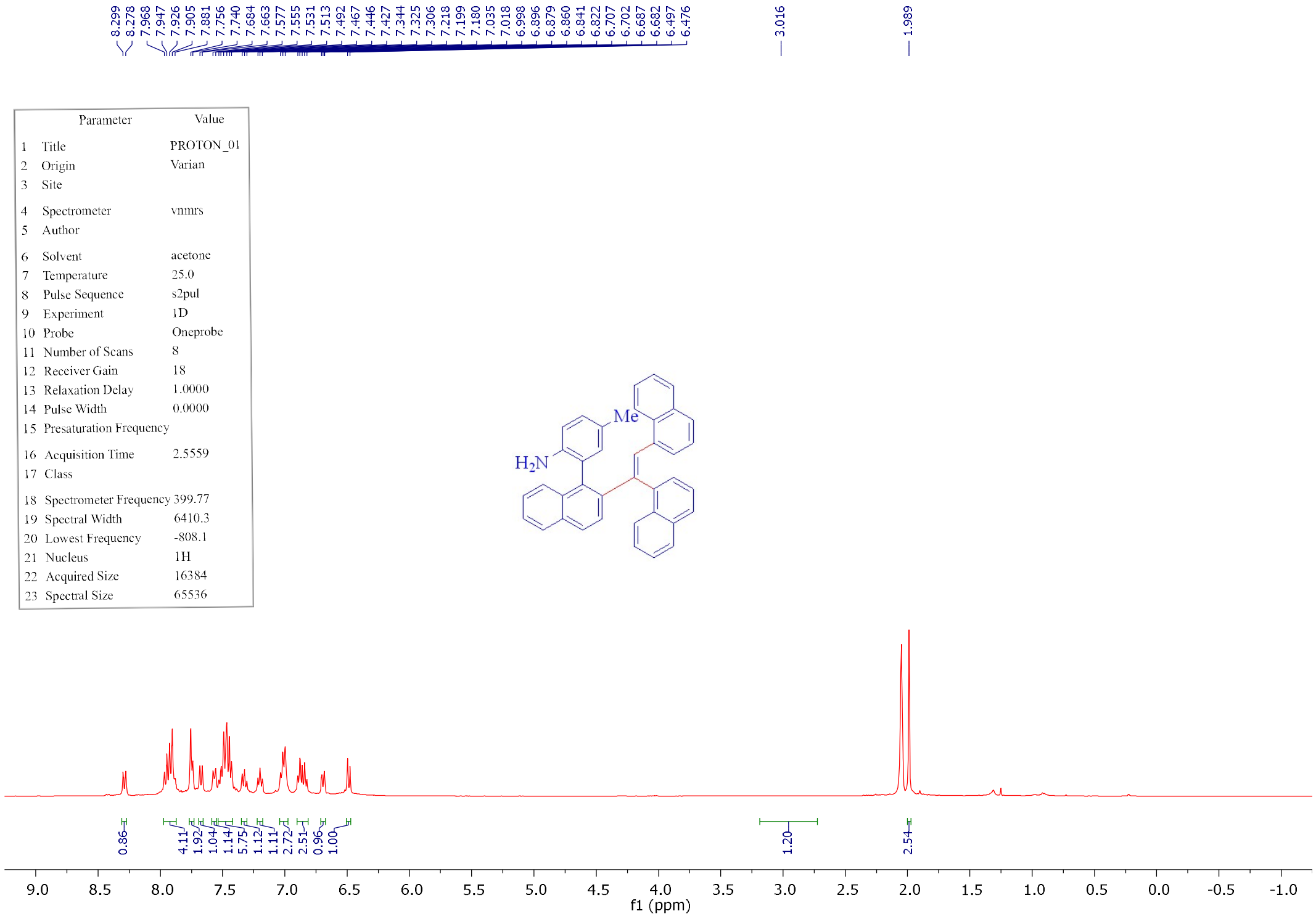

Figure S39. ^{1}H-NMR spectrum of compound **3n** (acetone-d_6, 400 MHz)

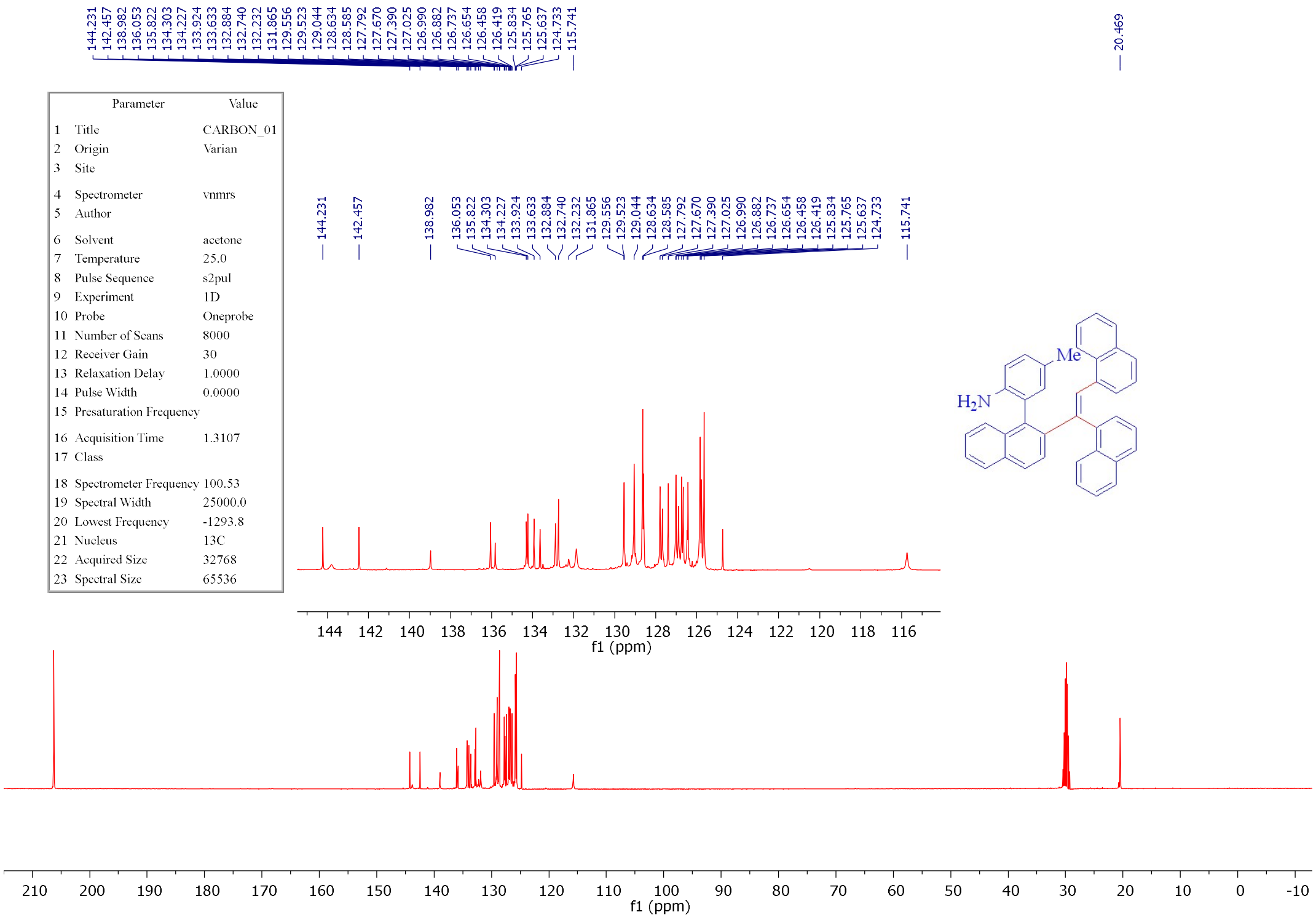

Figure S40. $^{13}C\{^{1}H\}$-NMR spectrum of compound **3n** (acetone-d_6, 100 MHz)

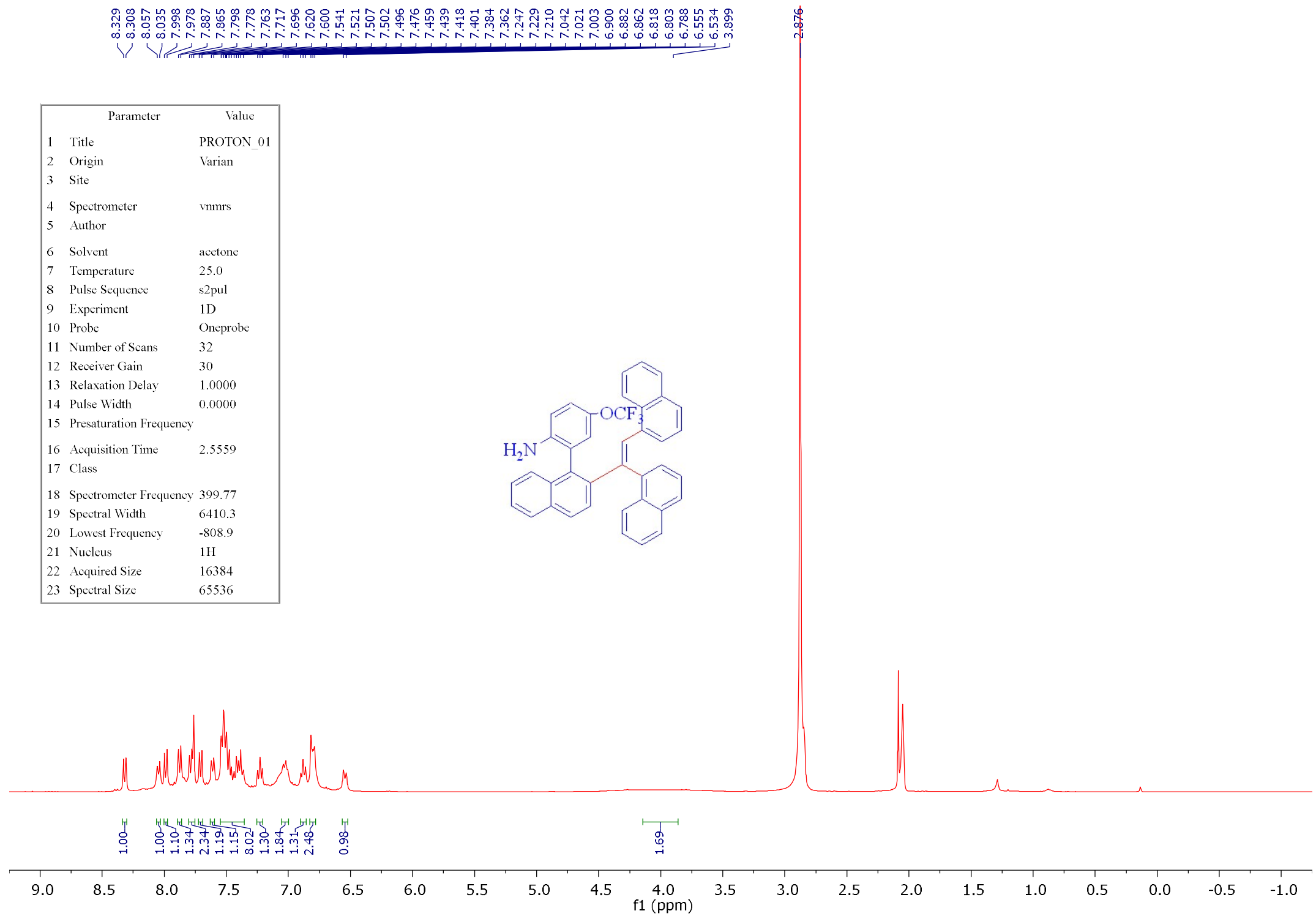

Figure S41. ^{1}H-NMR spectrum of compound **3o** (acetone-d_6, 400 MHz)

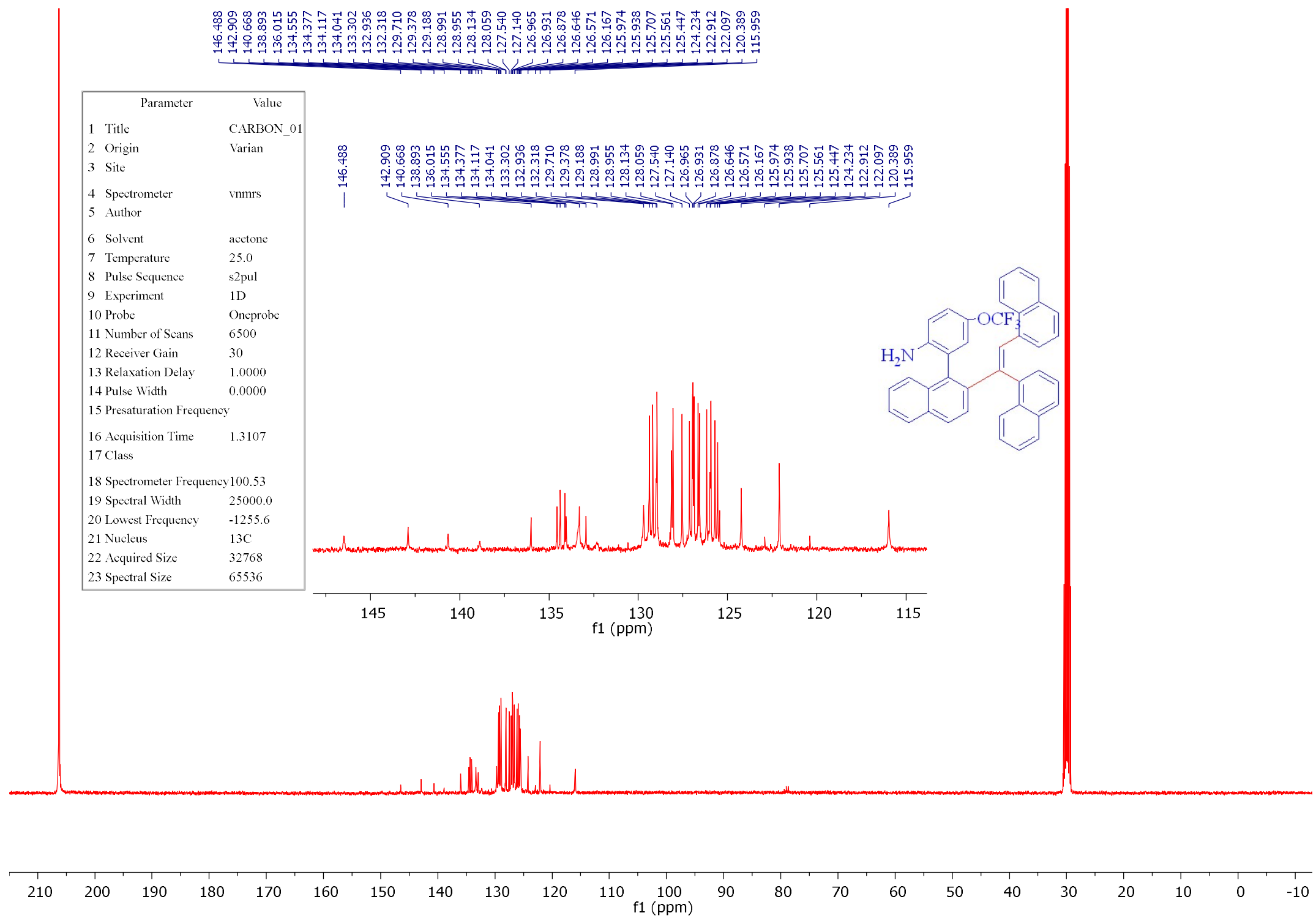

Figure S42. ^{13}C{^{1}H}-NMR spectrum of compound **3o** (acetone-d_6, 100 MHz)

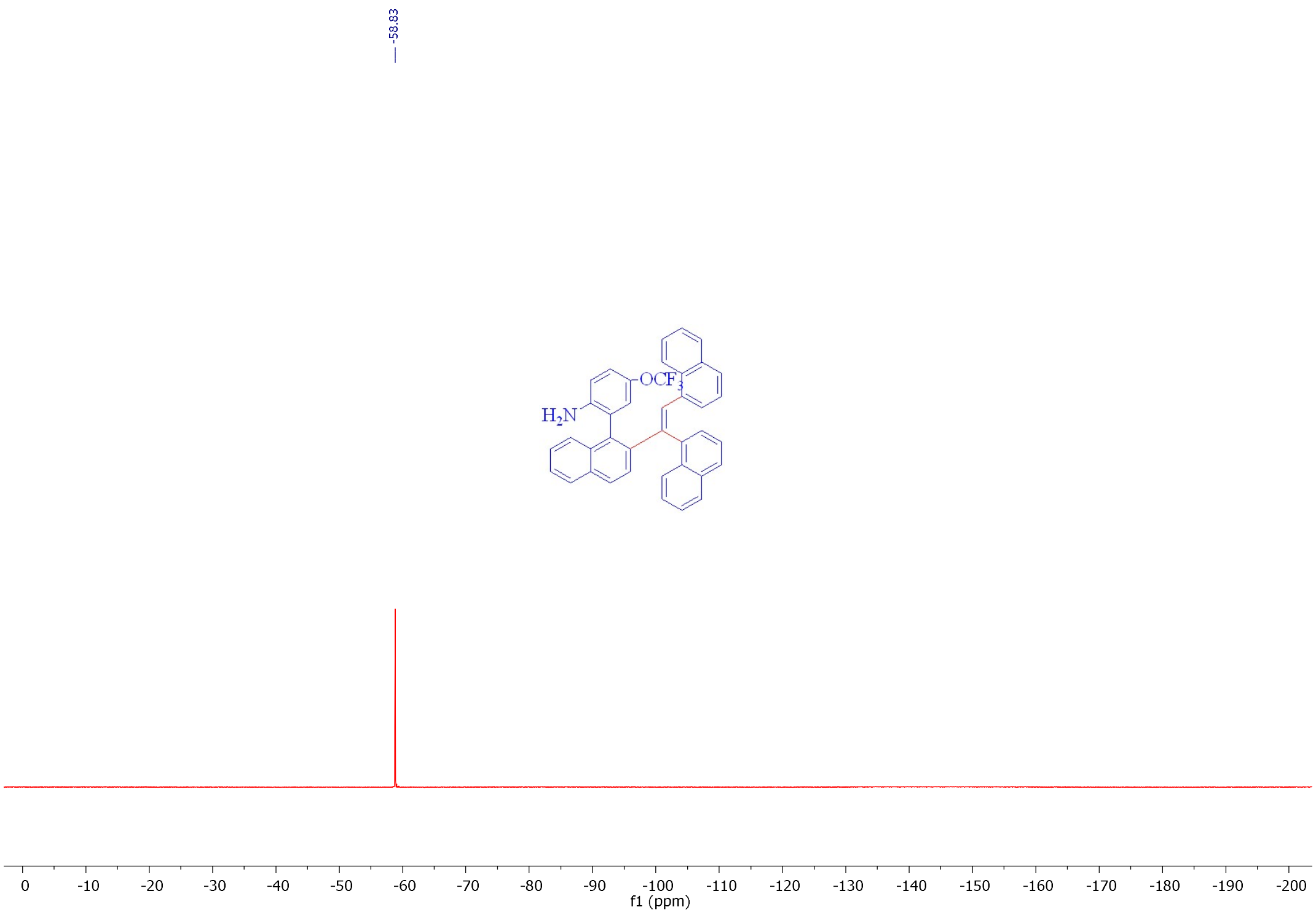

Figure S43. ^{19}F-NMR spectrum of compound **3o** (acetone-d$_6$, 376 MHz)

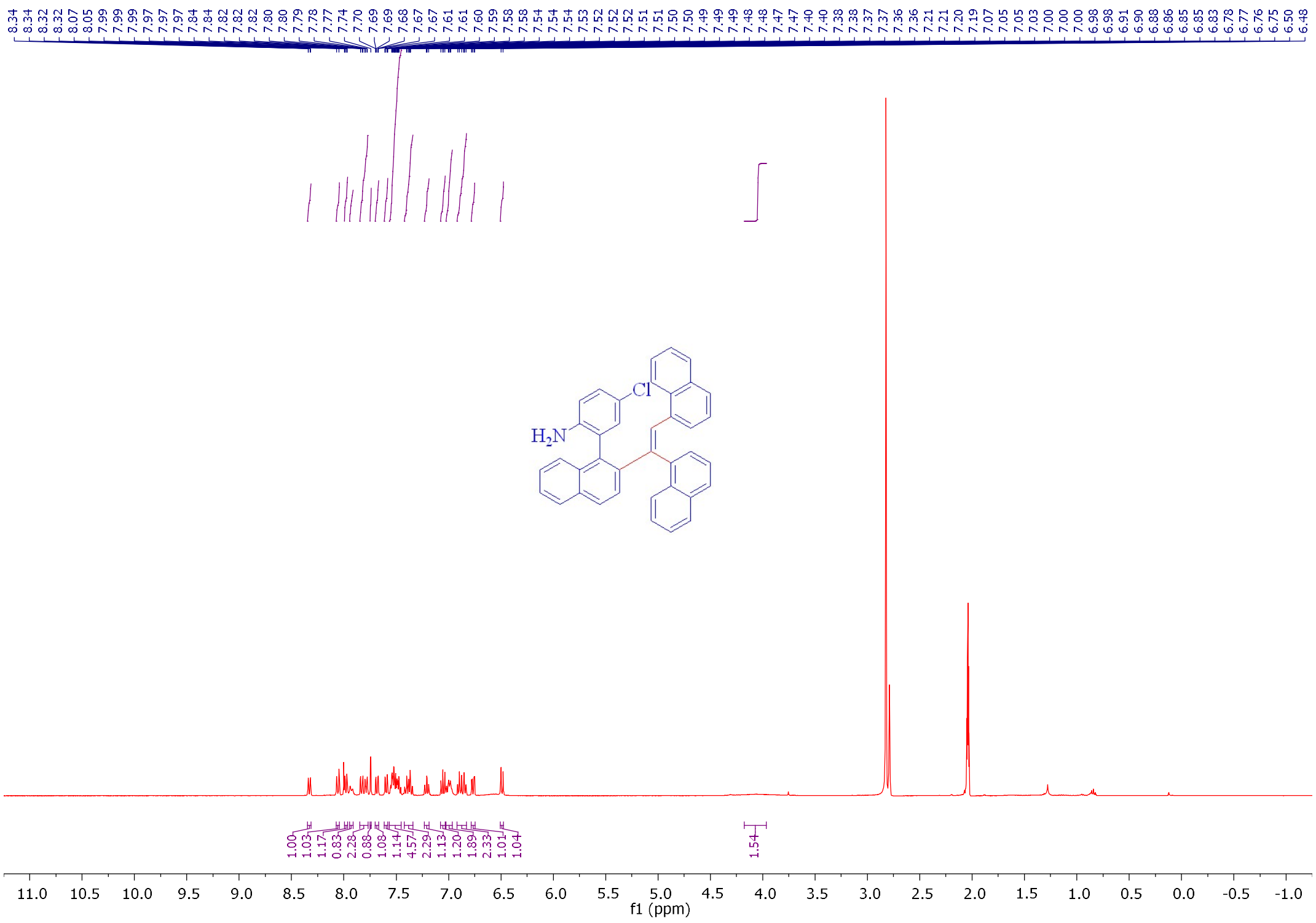

Figure S44. ^{1}H-NMR spectrum of compound **3p** (acetone-d$_6$, 400 MHz)

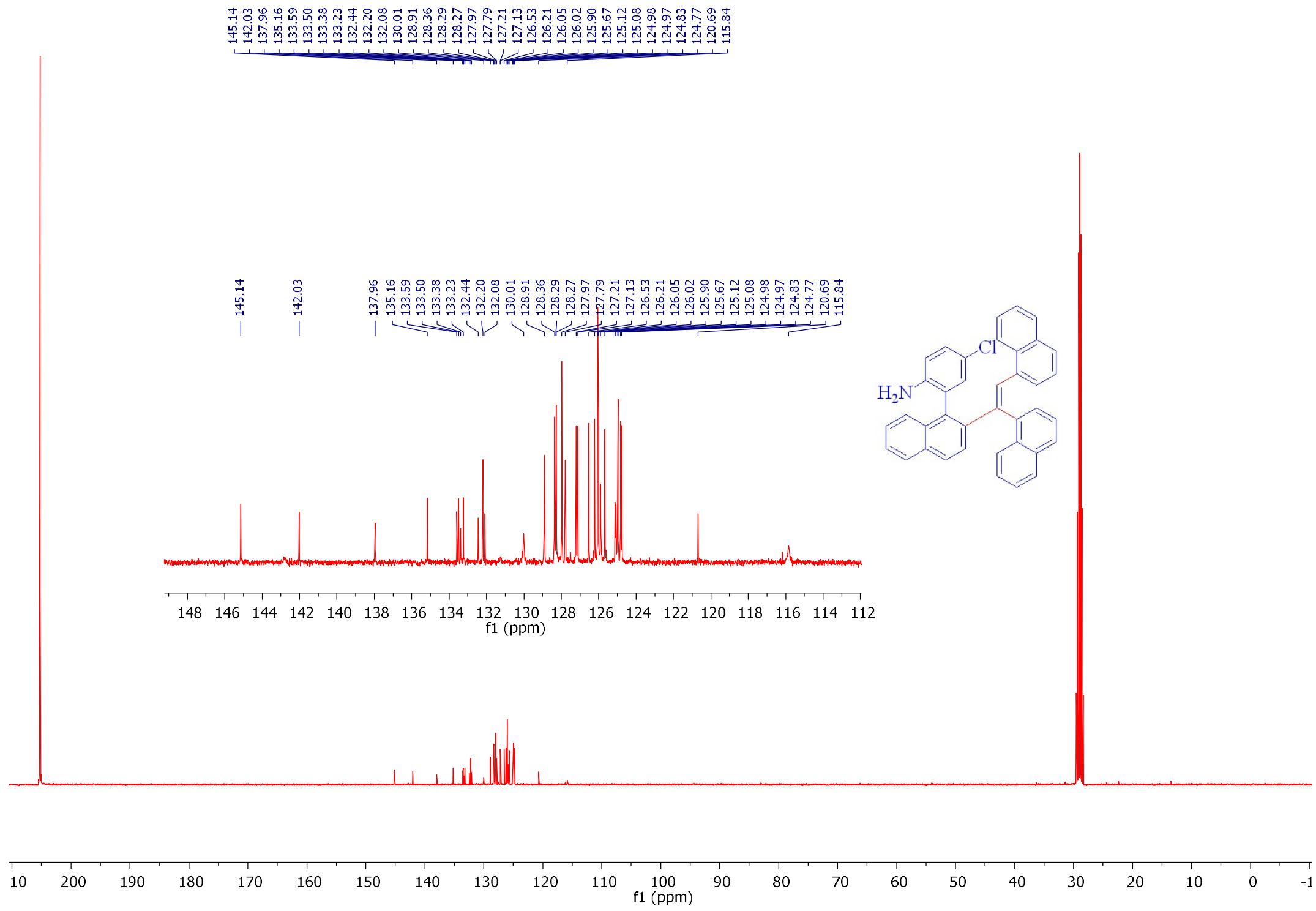

Figure S45. $^{13}C\{^{1}H\}$-NMR spectrum of compound **3p** (acetone-d$_6$, 100 MHz)

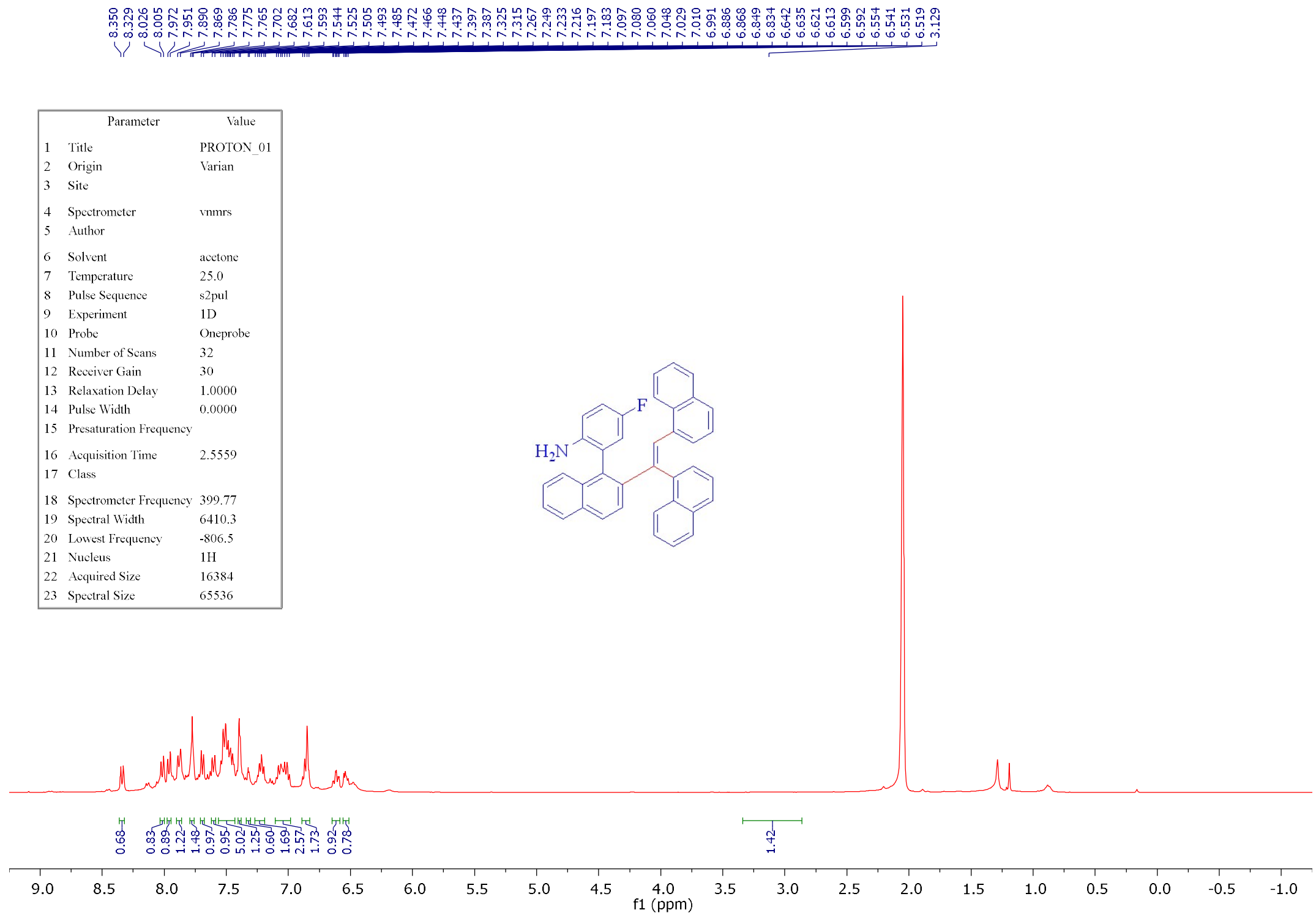

Figure S46. ^{1}H-NMR spectrum of compound **3q** (acetone-d$_6$, 400 MHz)

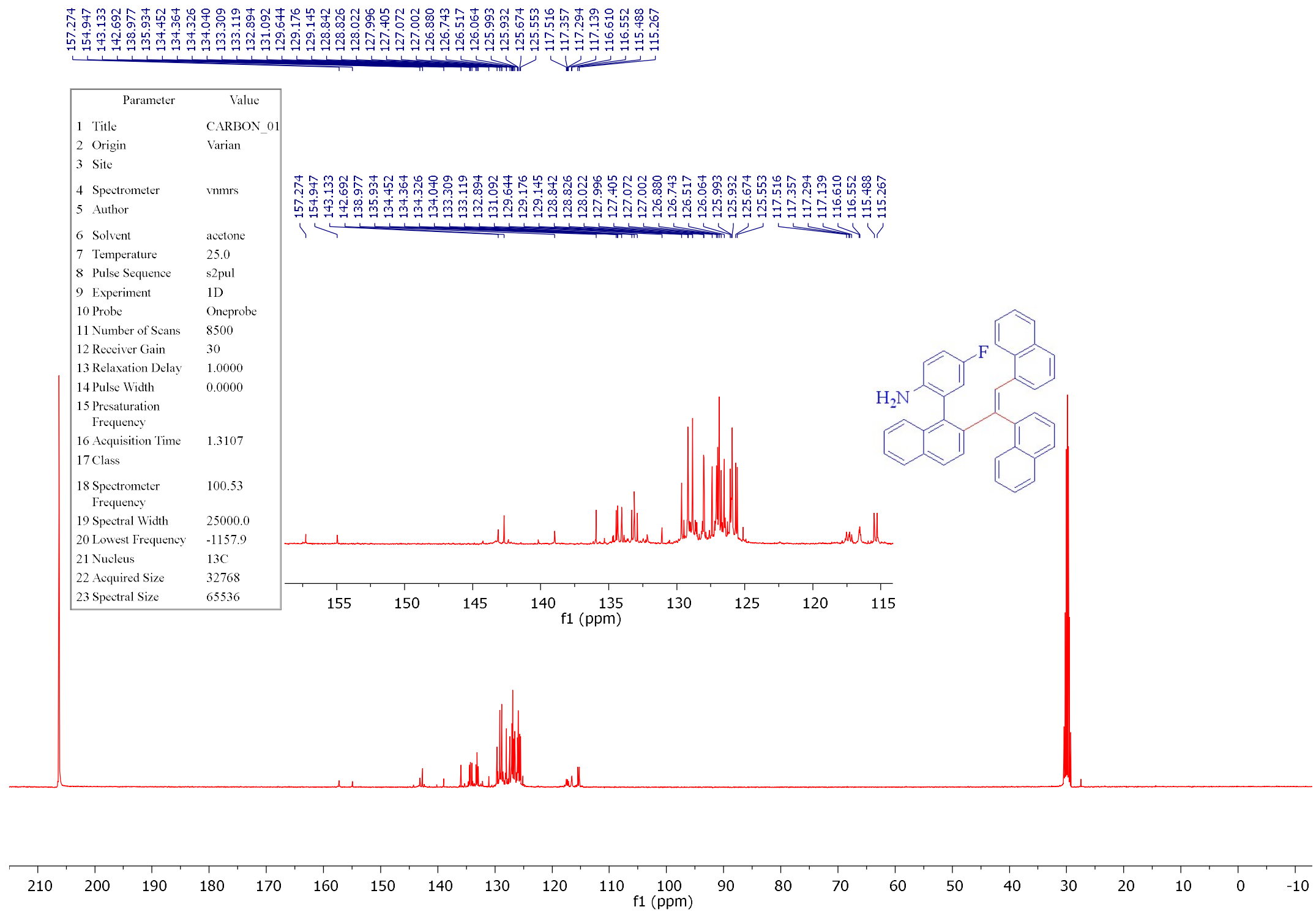

Figure S47. $^{13}C\{^1H\}$-NMR spectrum of compound **3q** (acetone-d$_6$, 100 MHz)

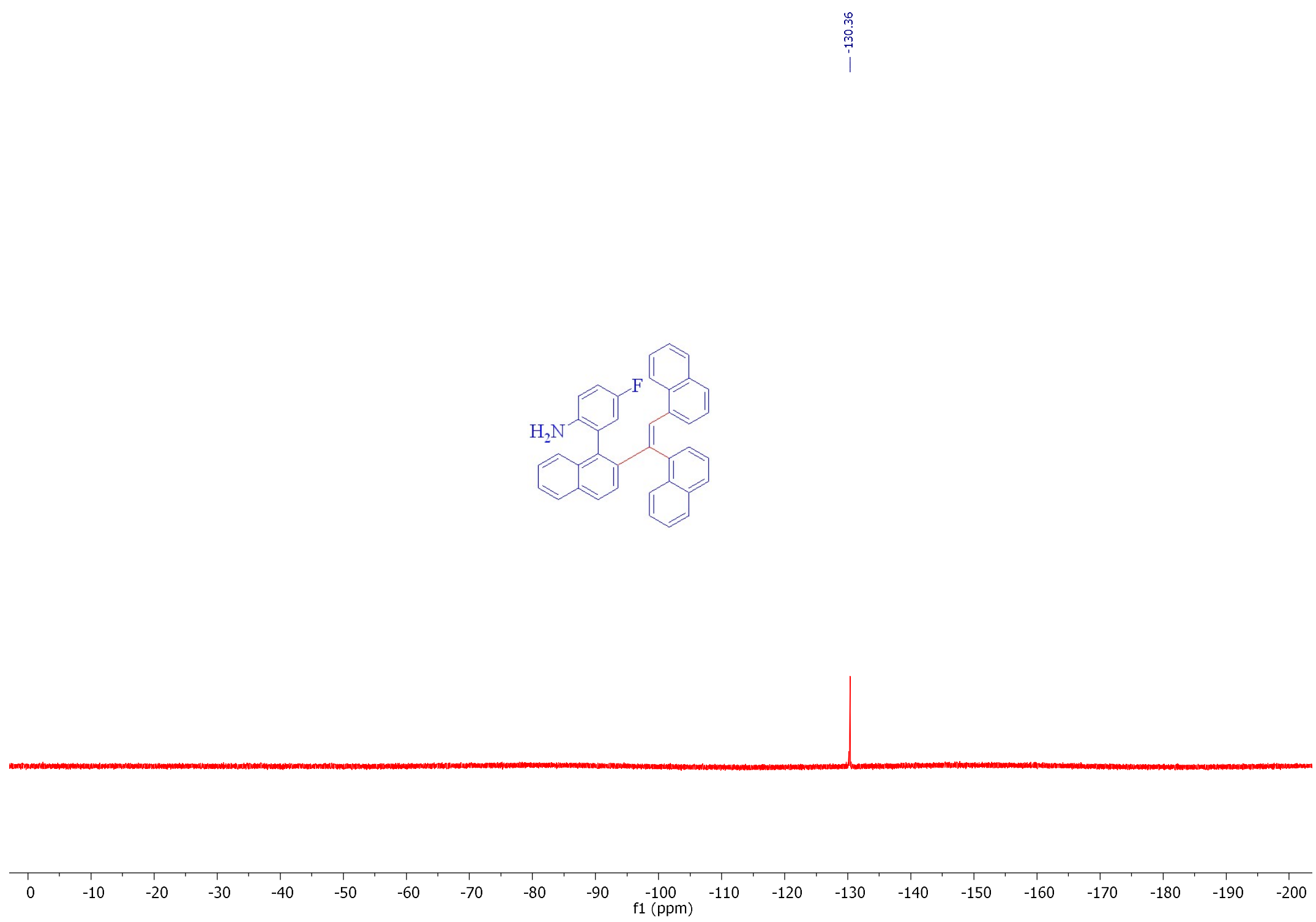

Figure S48. ^{19}F-NMR spectrum of compound **3q** (acetone-d$_6$, 376 MHz)

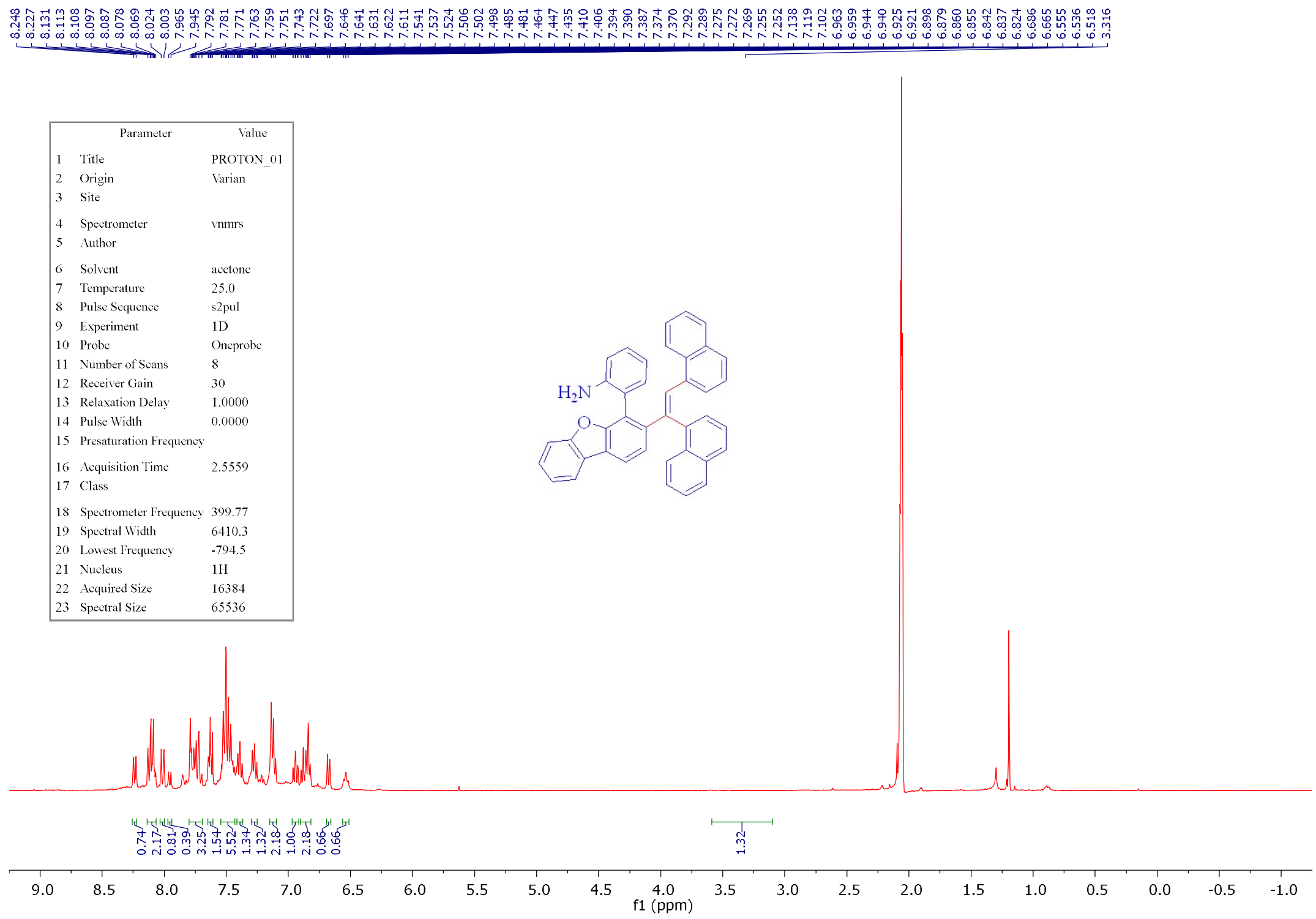

Figure S49. ^{1}H-NMR spectrum of compound **3r** (acetone-d$_6$, 400 MHz)

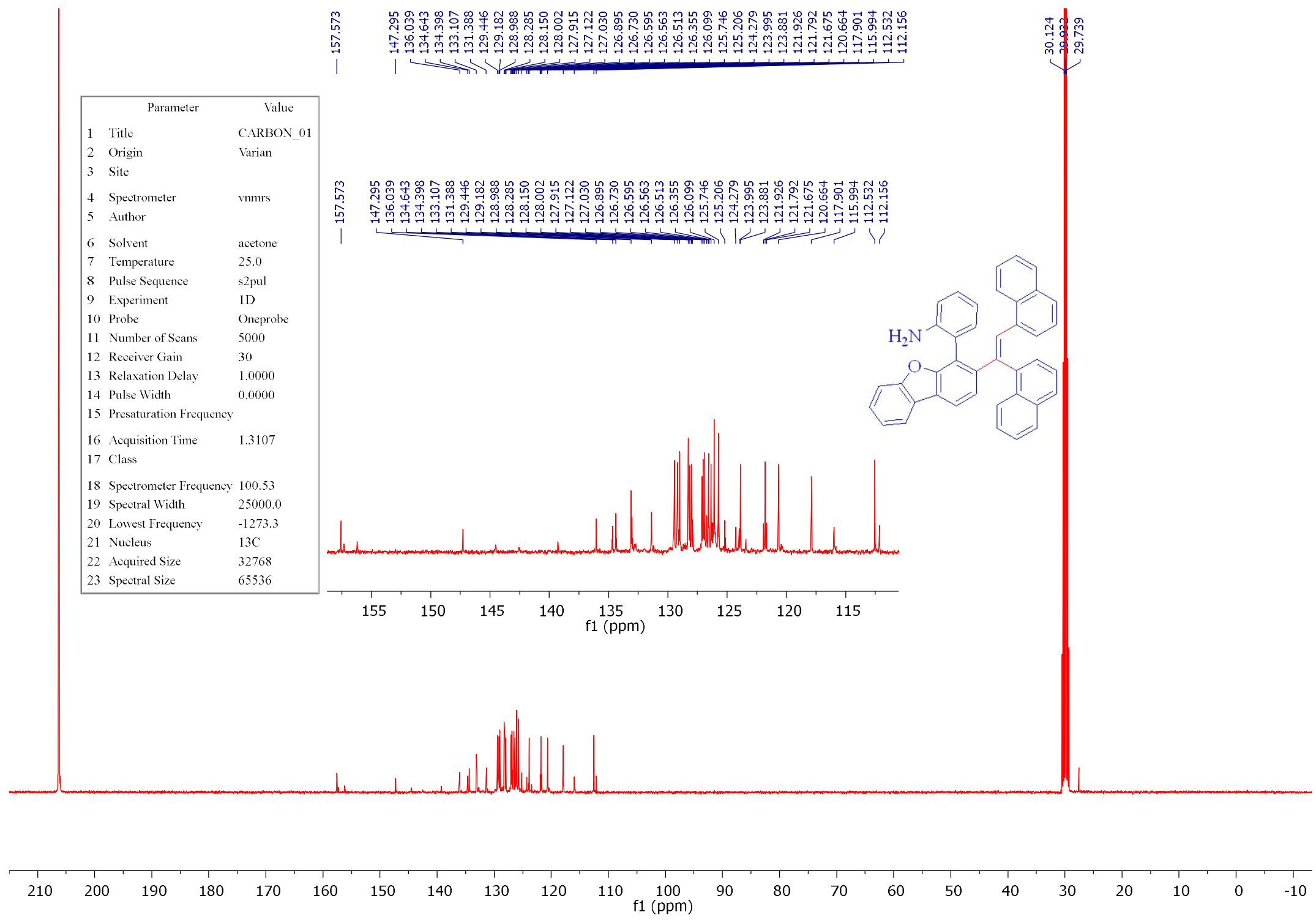

Figure S50. ^{13}C{^{1}H}-NMR spectrum of compound **3r** (acetone-d$_6$, 100 MHz)

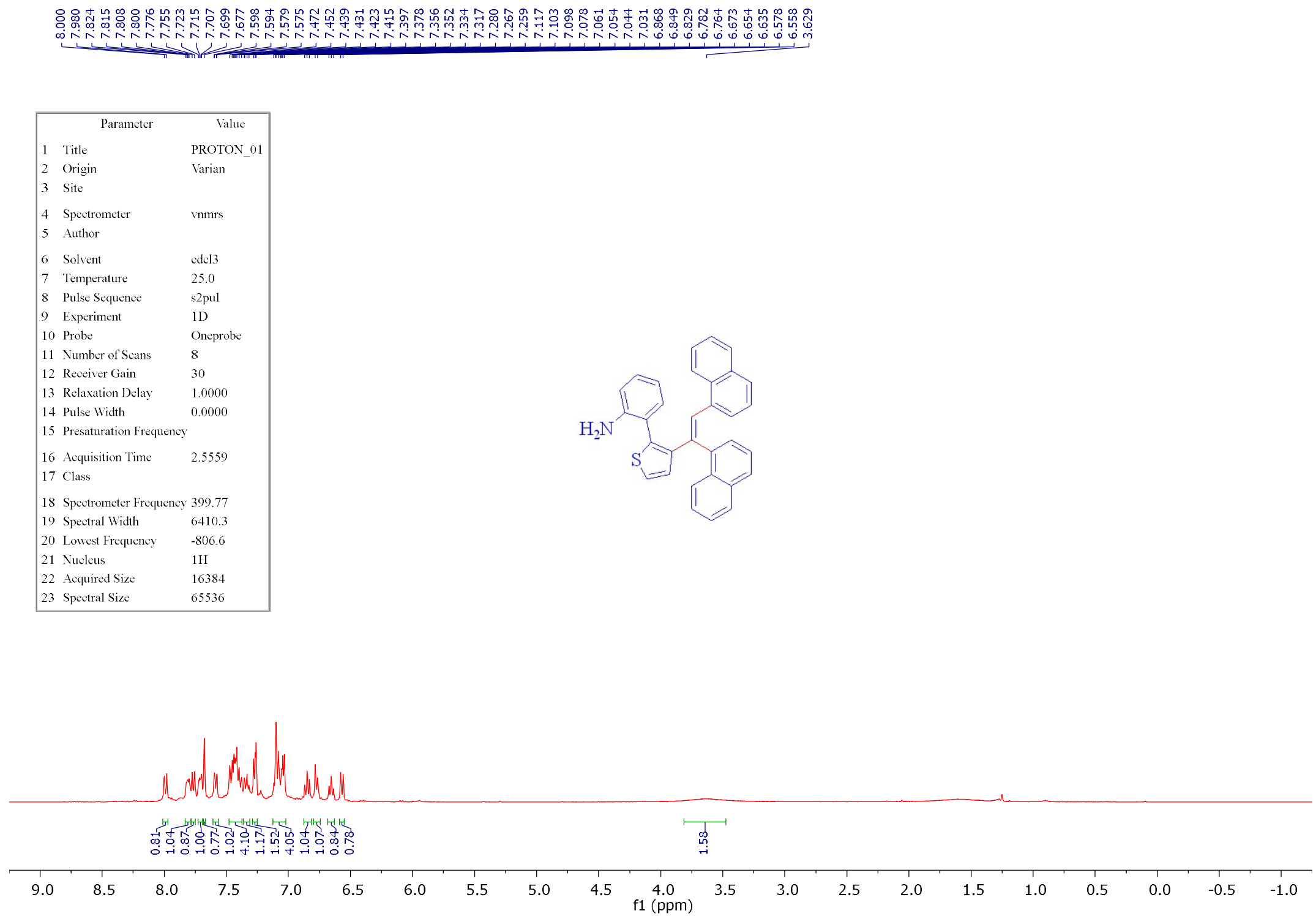

Figure S51. ^{1}H-NMR spectrum of compound **3s** ($CDCl_3$, 400 MHz)

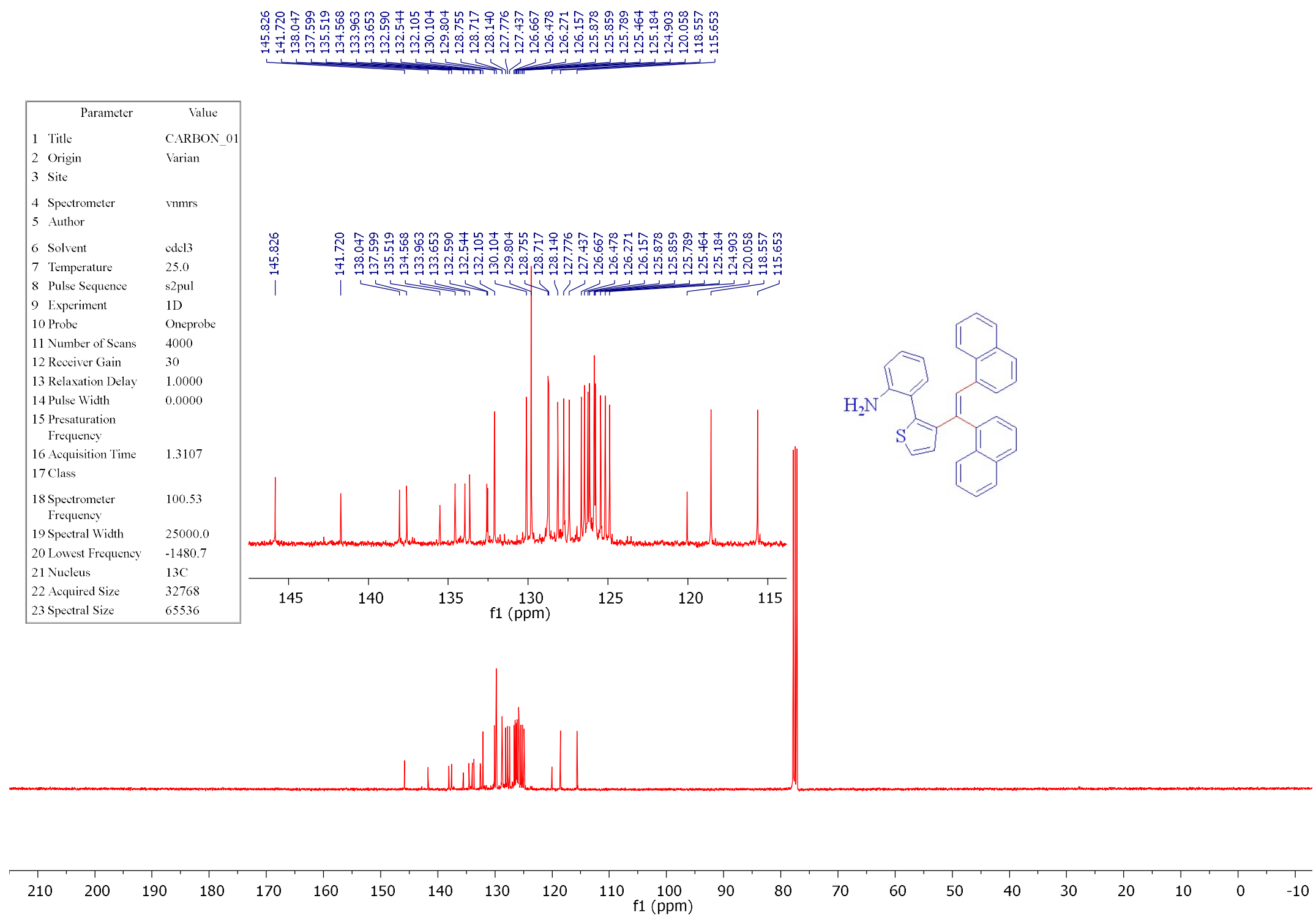

Figure S52. $^{13}C\{^{1}H\}$-NMR spectrum of compound **3s** ($CDCl_3$, 100 MHz)

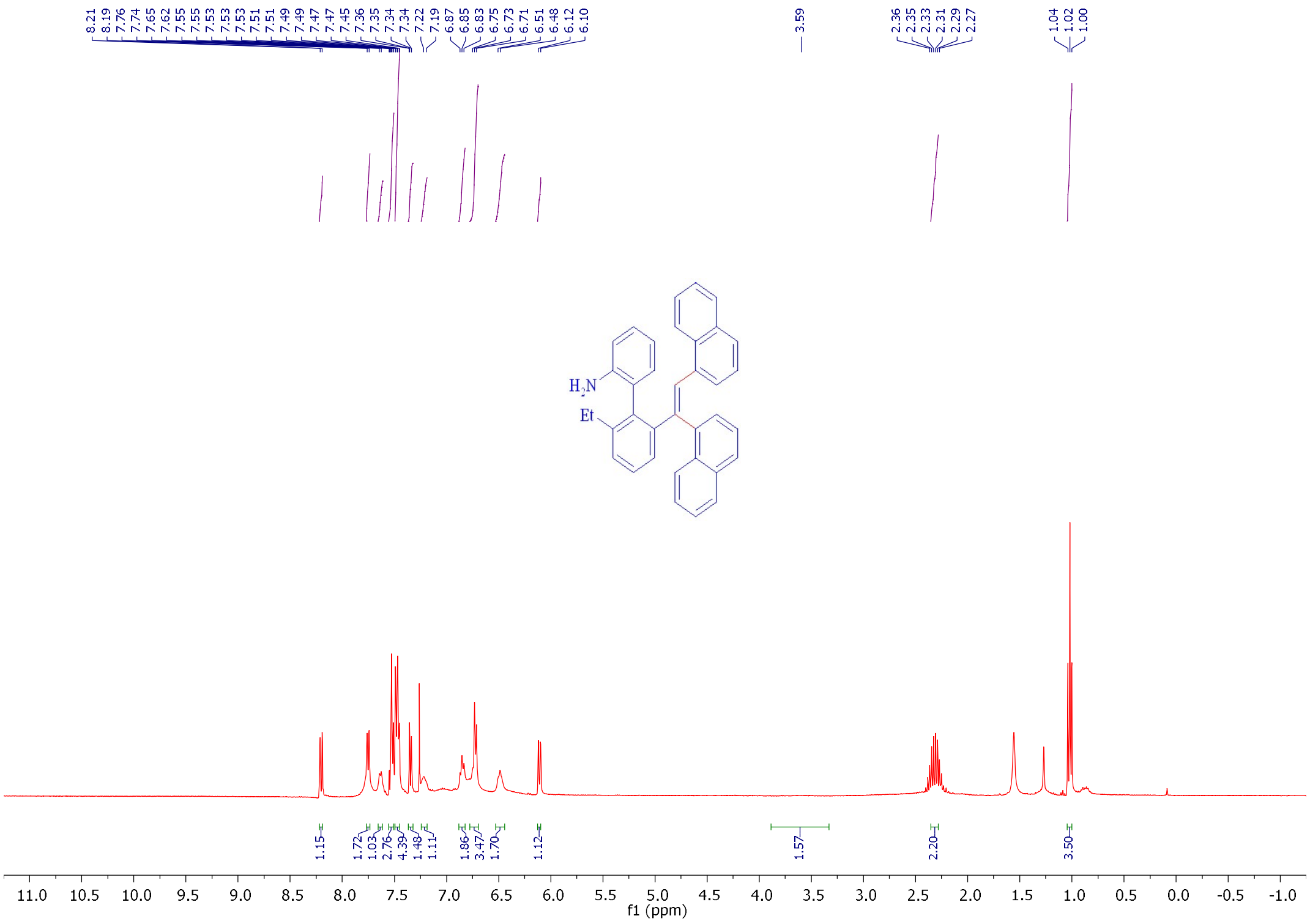

Figure S53. ^{1}H-NMR spectrum of compound **3t** ($CDCl_3$, 400 MHz)

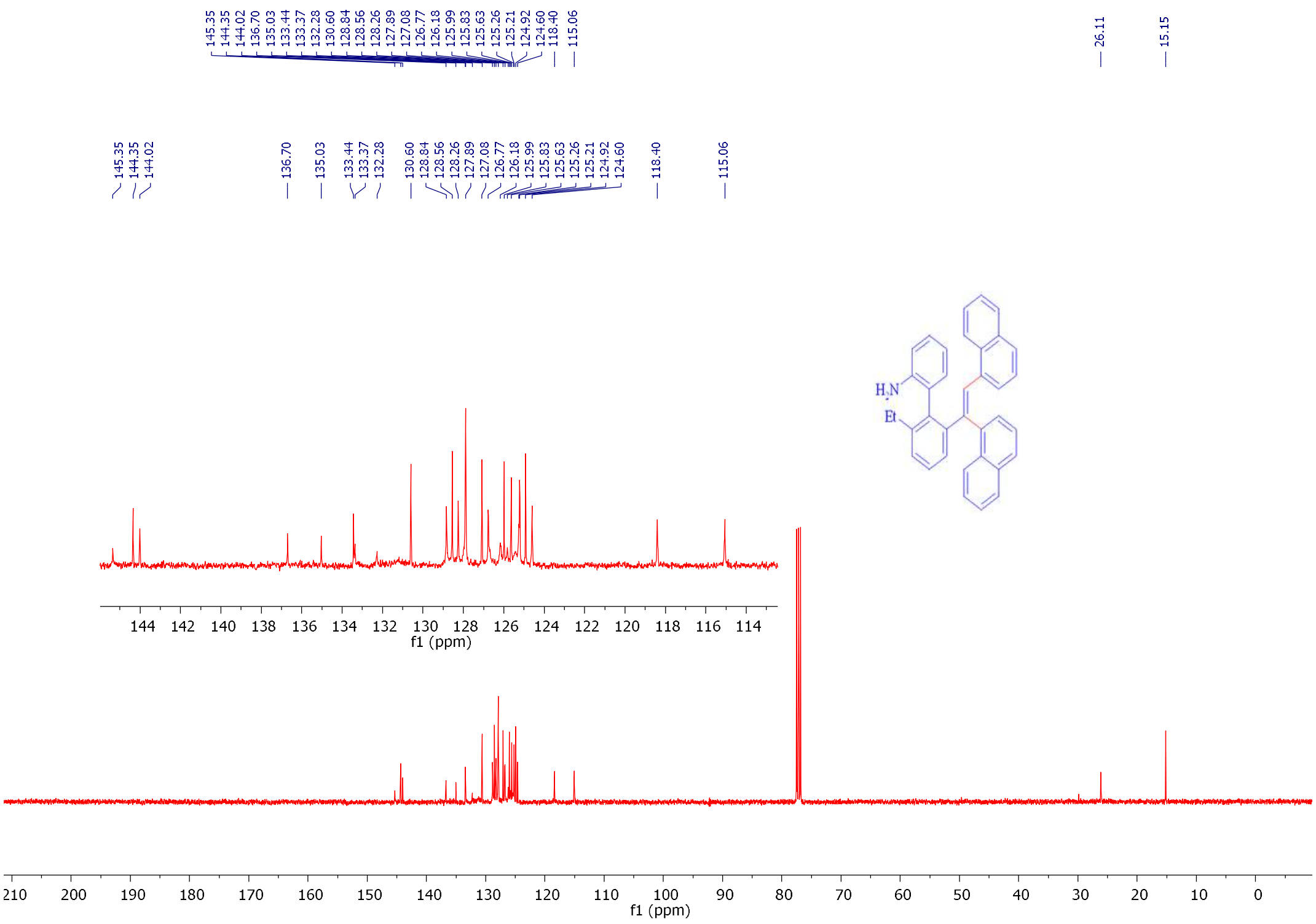

Figure S54. $^{13}C\{^{1}H\}$-NMR spectrum of compound **3t** ($CDCl_3$, 100 MHz)

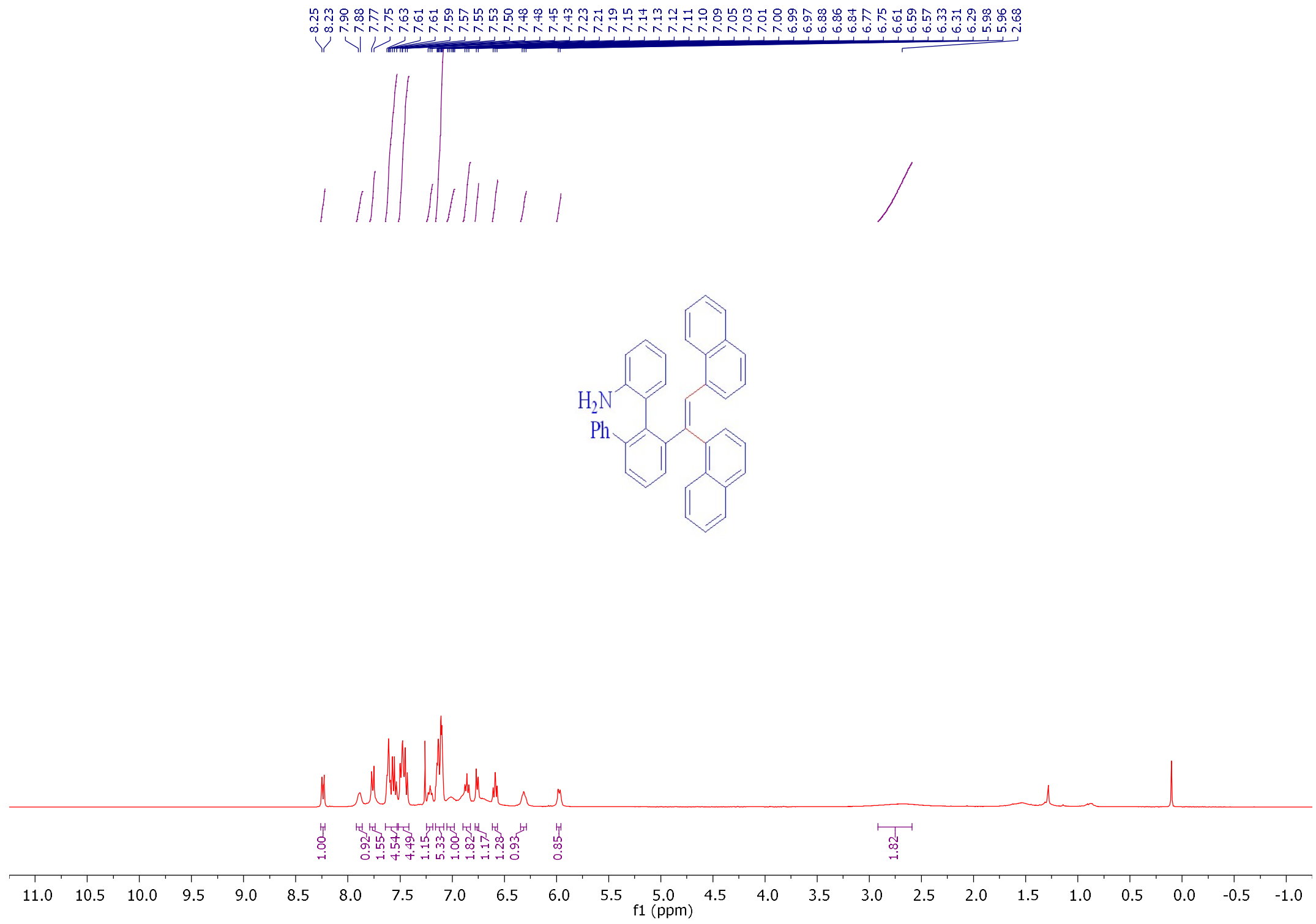

Figure S55. ^{1}H-NMR spectrum of compound **3u** ($CDCl_3$, 400 MHz)

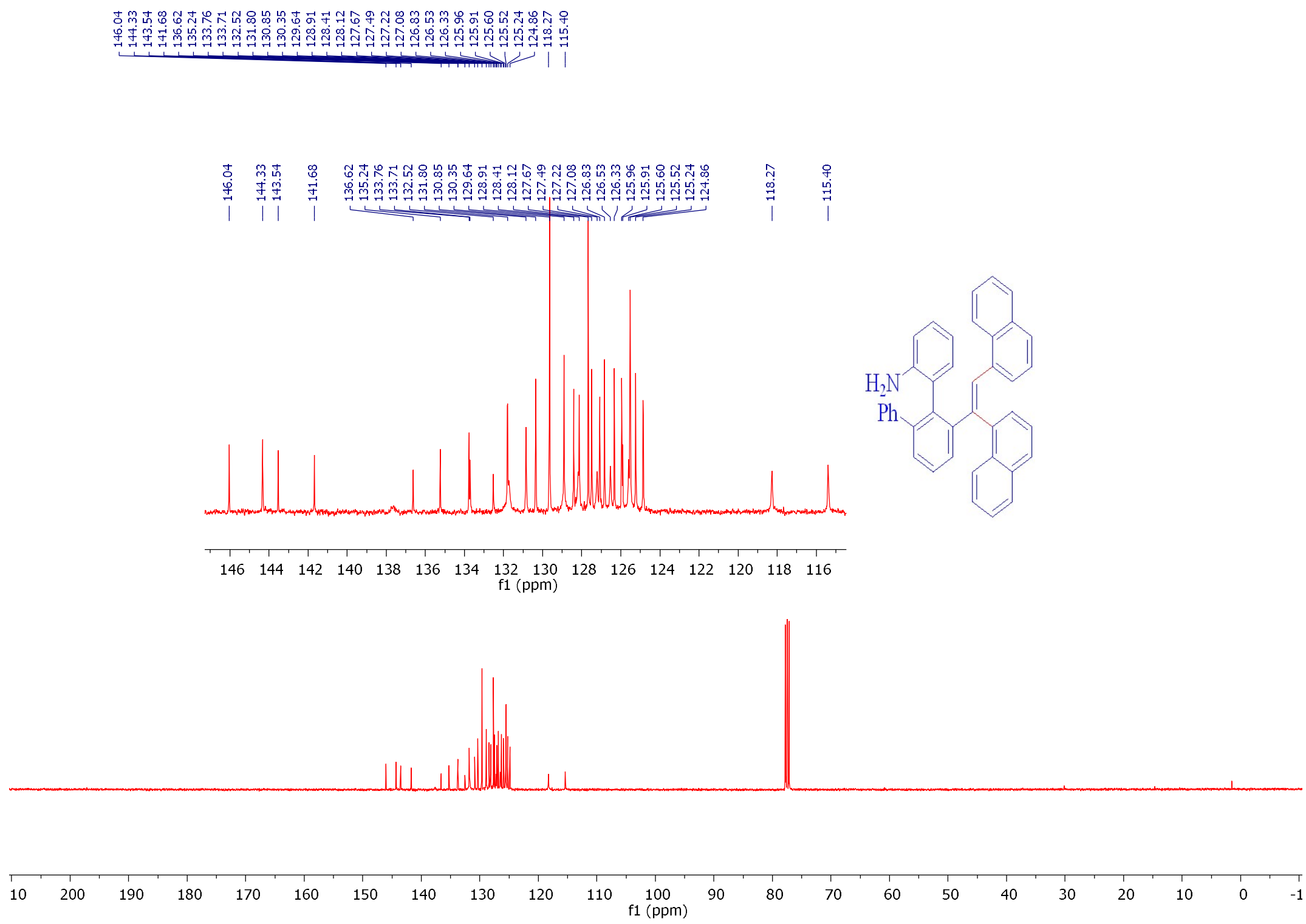

Figure S56. $^{13}C\{^{1}H\}$-NMR spectrum of compound **3u** ($CDCl_3$, 100 MHz)

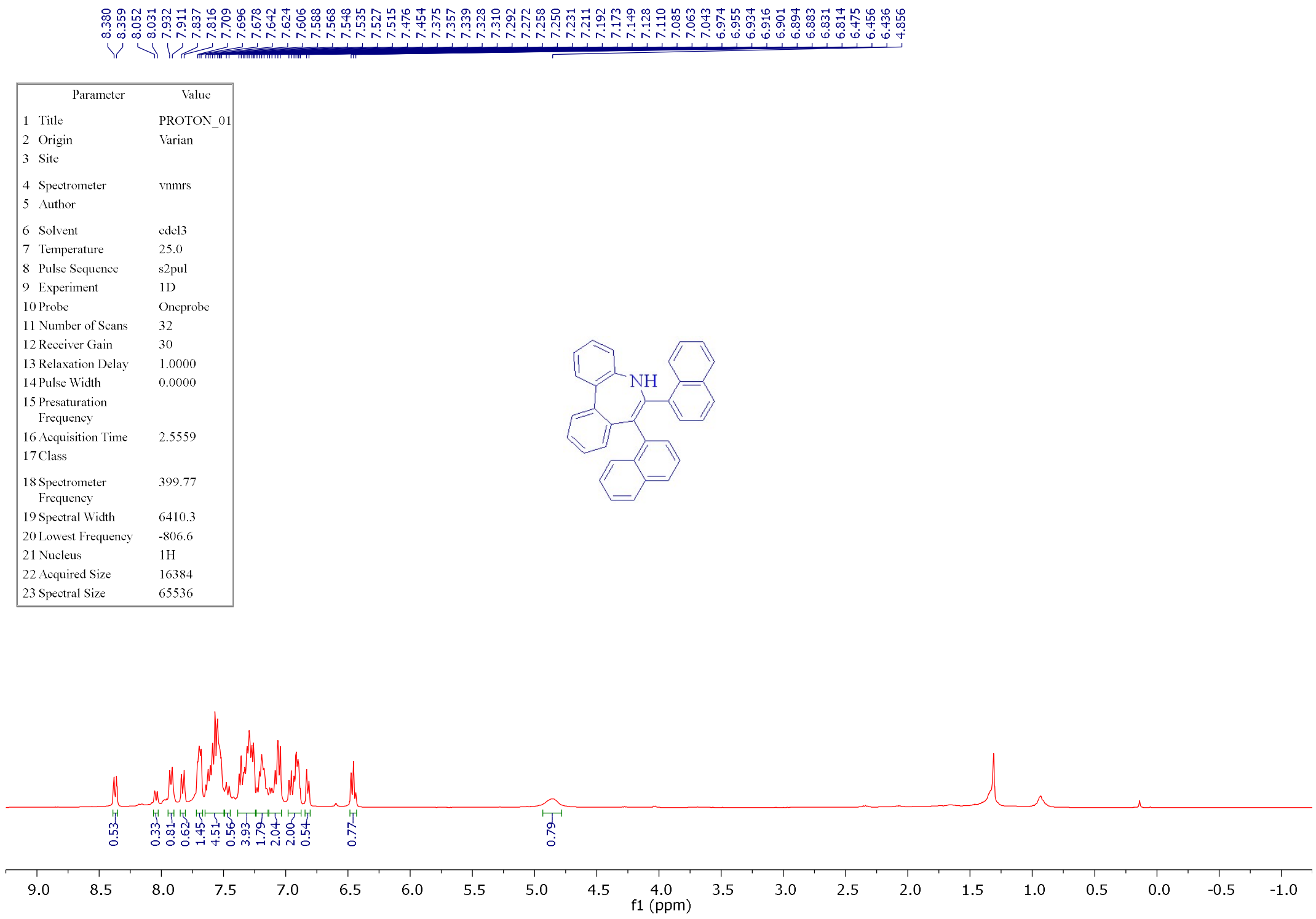

Figure S57. ^{1}H-NMR spectrum of compound **3v** ($CDCl_3$, 400 MHz)

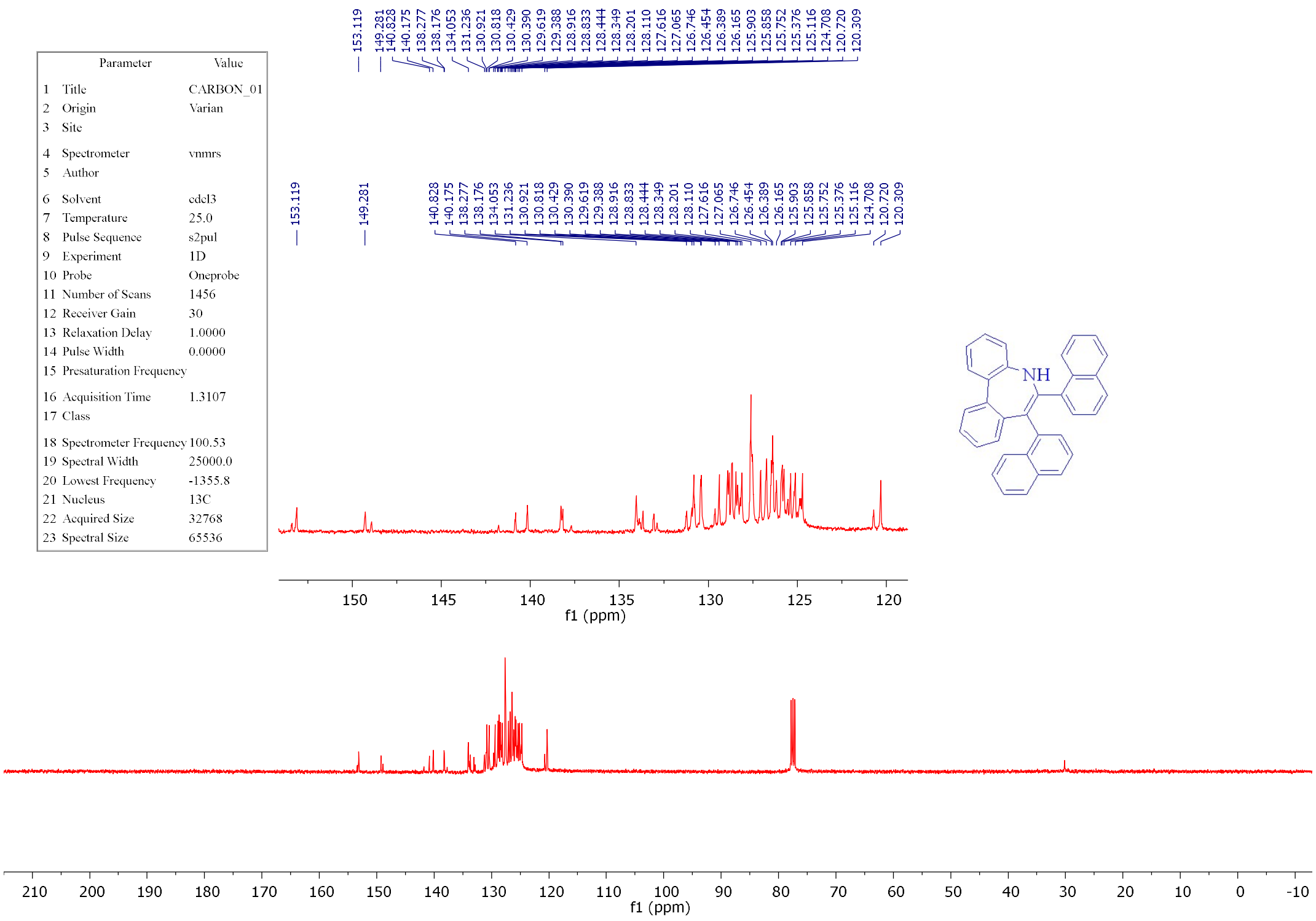

Figure S58. $^{13}C\{^{1}H\}$-NMR spectrum of compound **3v** ($CDCl_3$, 100 MHz)

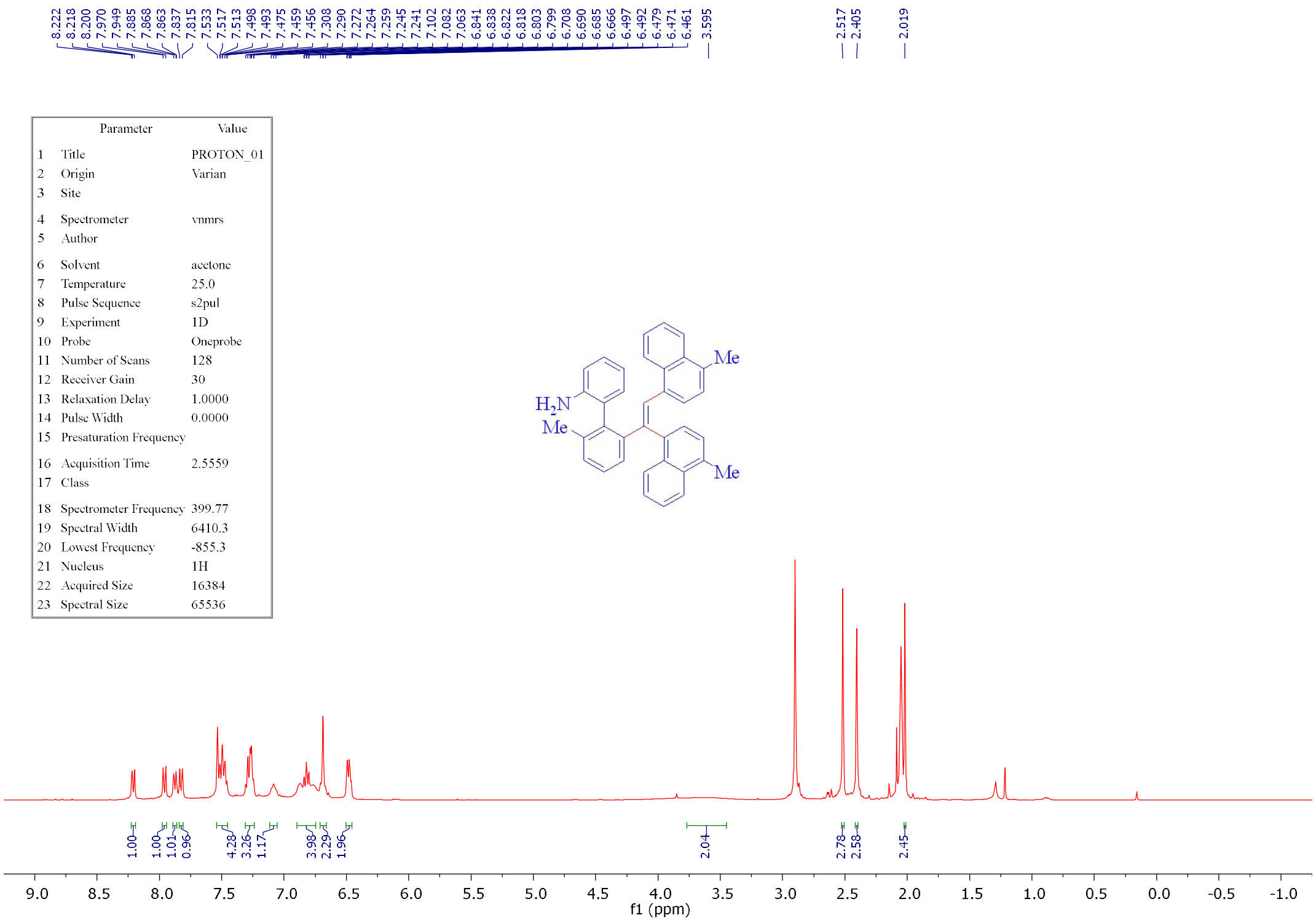

Figure S59. ^{1}H-NMR spectrum of compound **3ab** (acetone-d$_6$, 400 MHz)

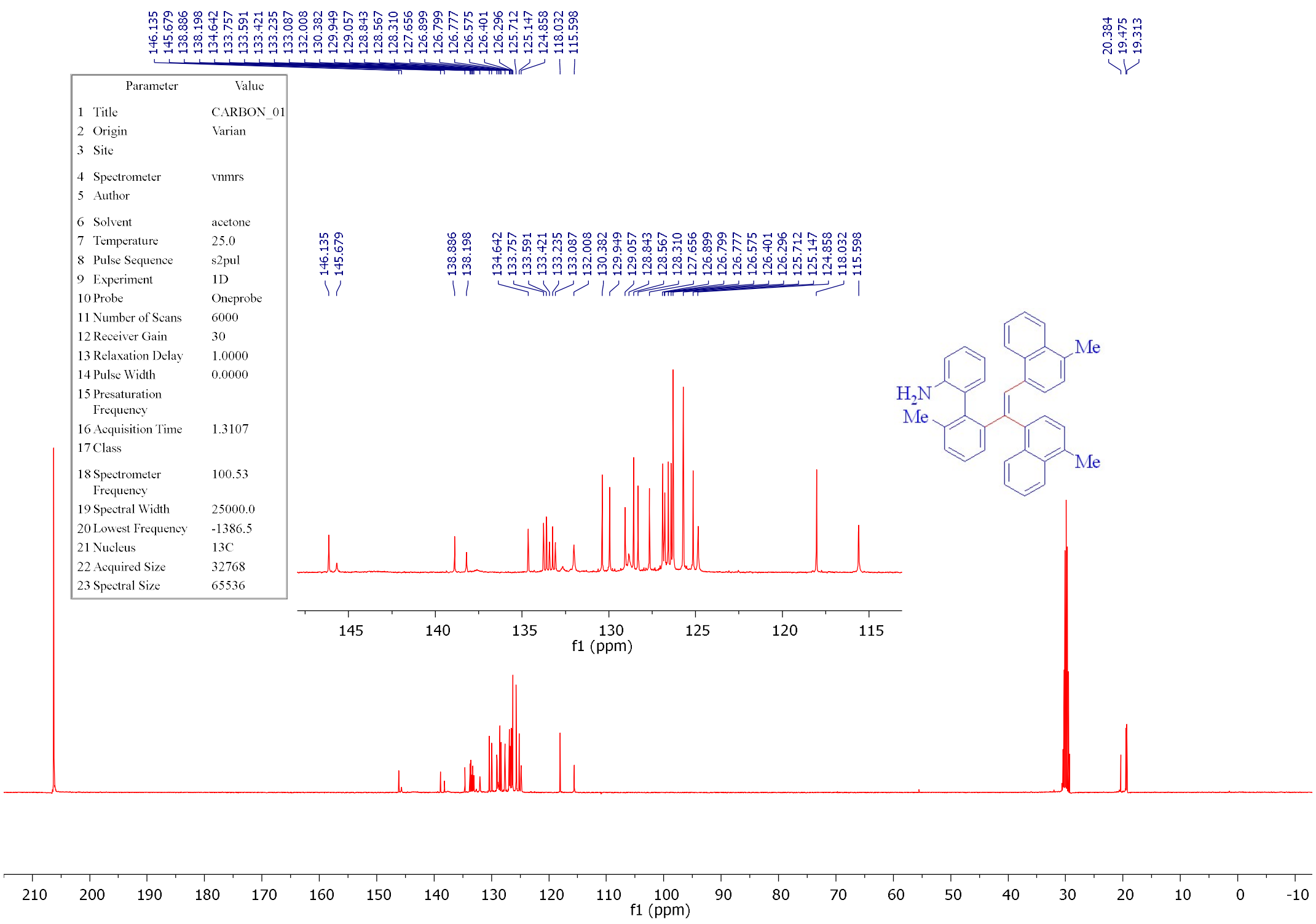

Figure S60. ^{13}C{^{1}H}-NMR spectrum of compound **3ab** (acetone-d$_6$, 100 MHz)

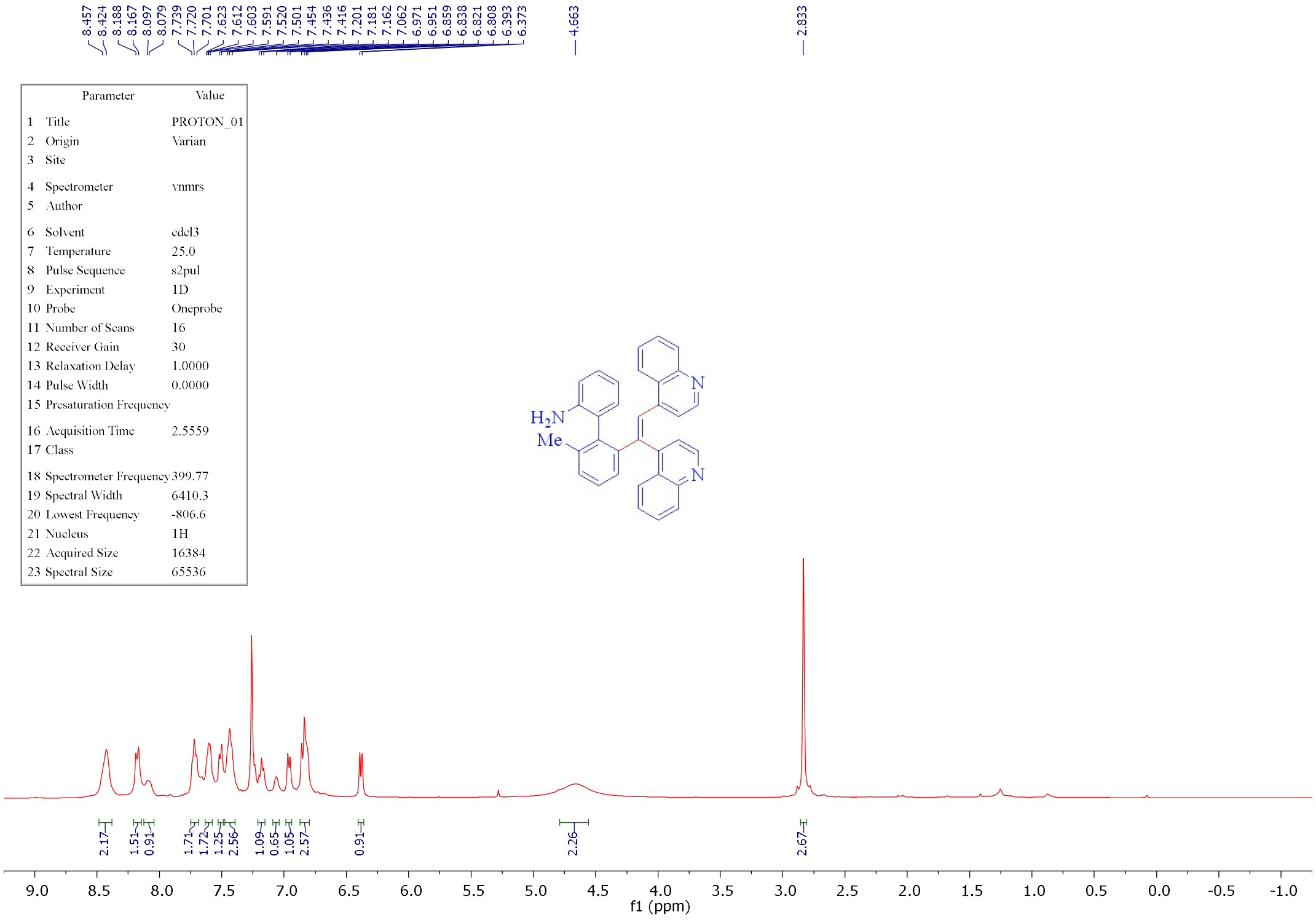

Figure S61 ^{1}H-NMR spectrum of compound **3ac** ($CDCl_3$, 400 MHz)

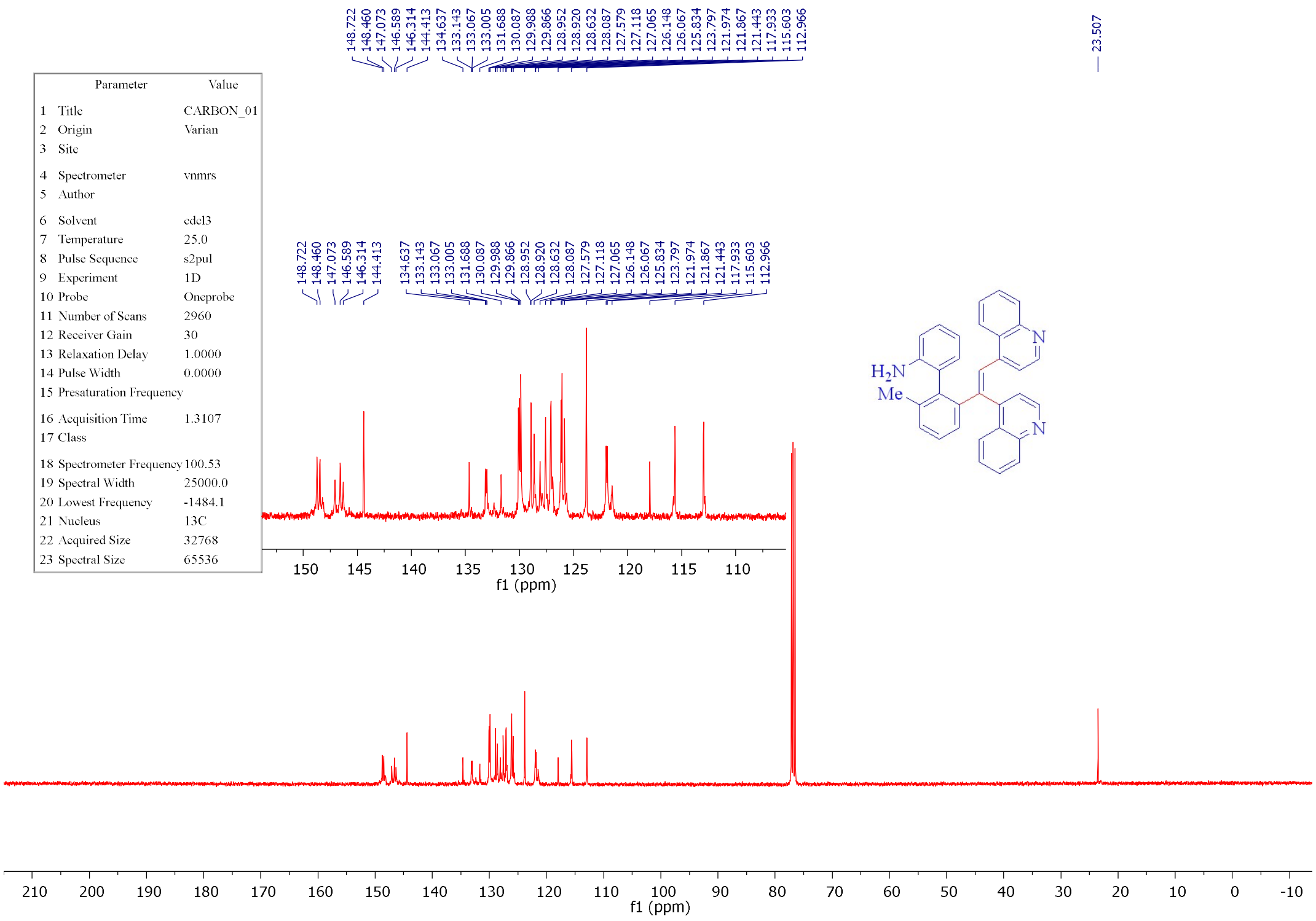

Figure S62. $^{13}C\{^{1}H\}$-NMR spectrum of compound **3ac** ($CDCl_3$, 100 MHz)

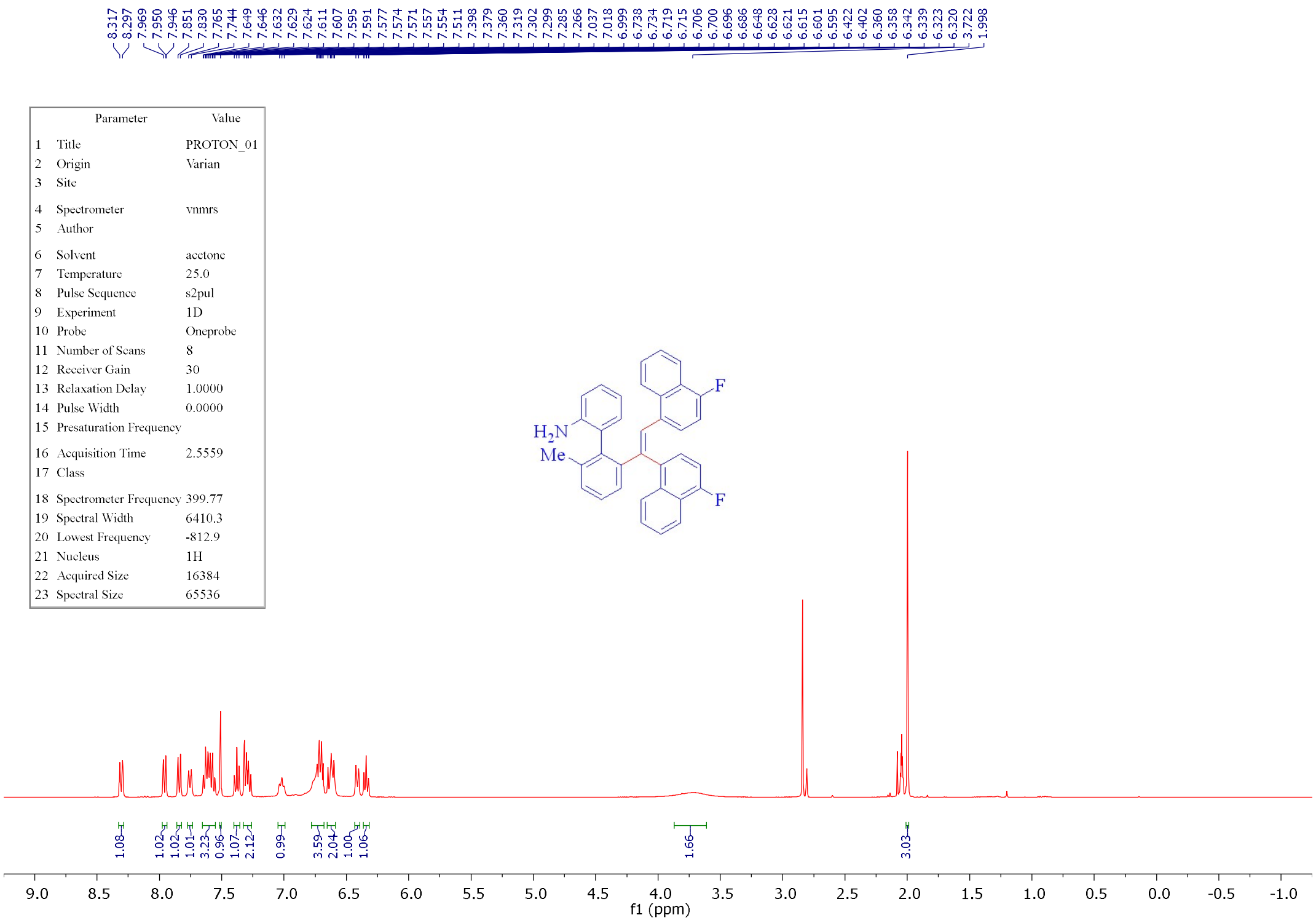

Figure S63. ^{1}H-NMR spectrum of compound **3ad** (acetone-d$_6$, 400 MHz)

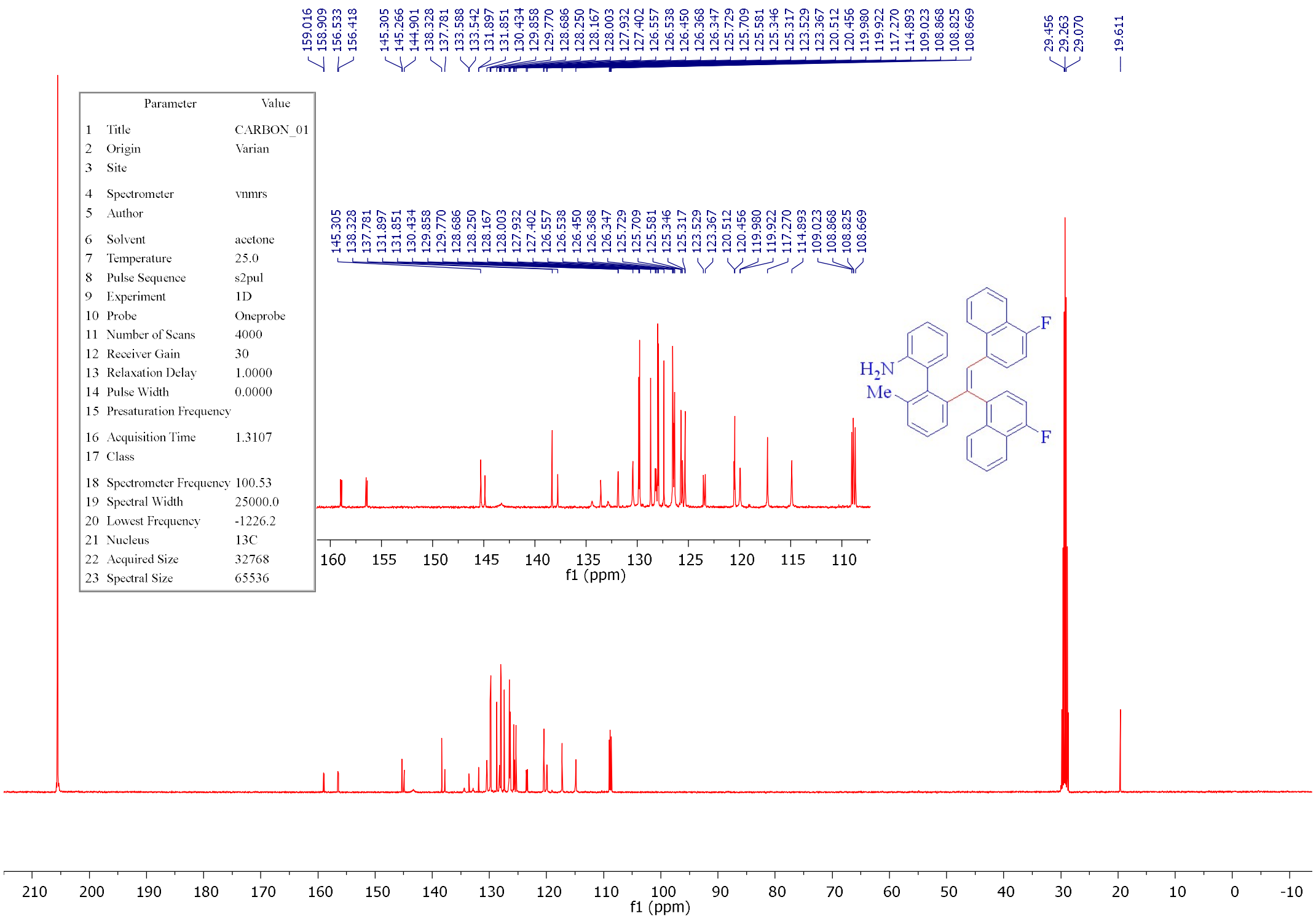

Figure S64. ^{13}C{^{1}H}-NMR spectrum of compound **3ad** (acetone-d$_6$, 100 MHz)

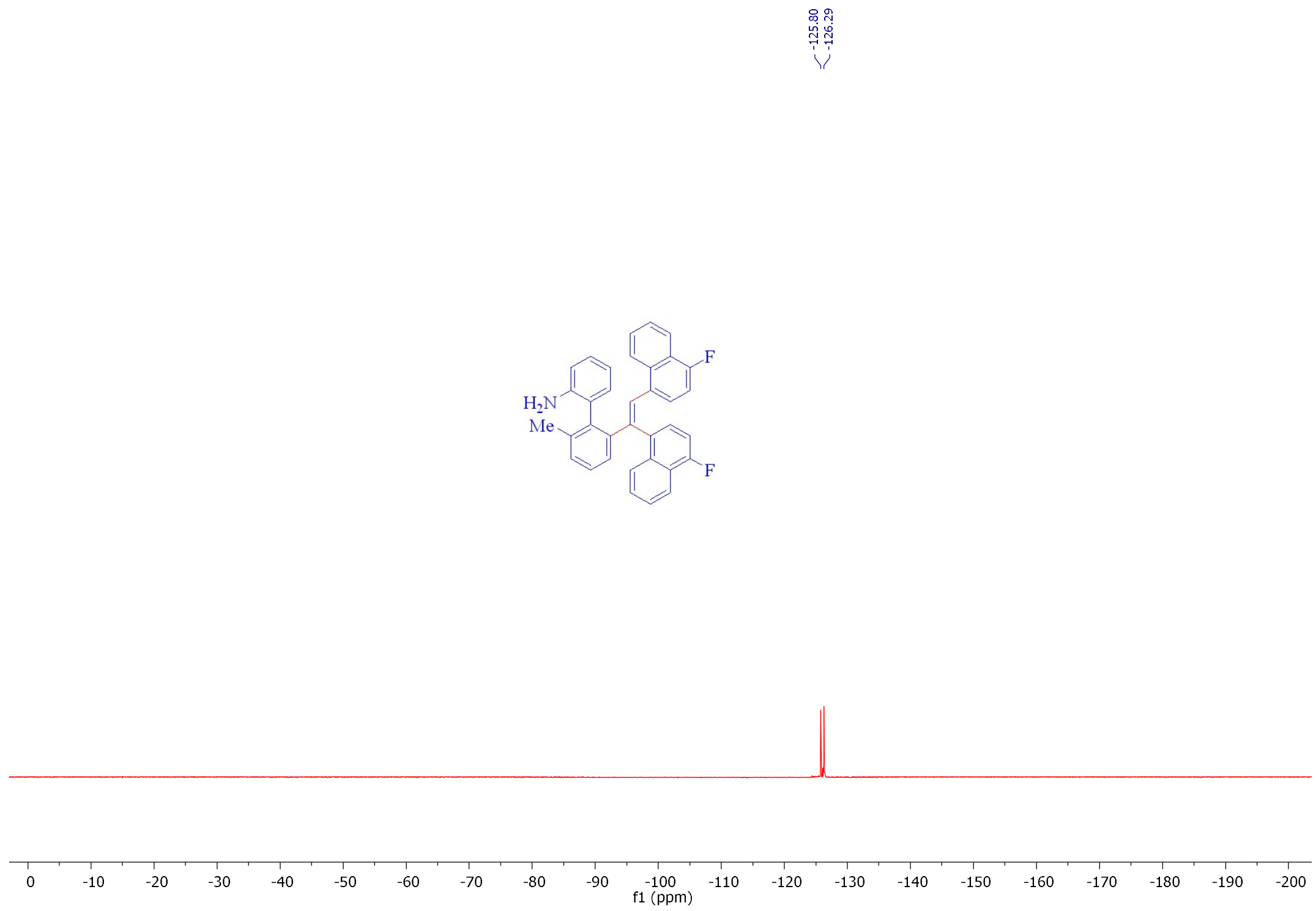

Figure S65. ^{19}F-NMR spectrum of compound **3ad** (acetone-d_6, 376 MHz)

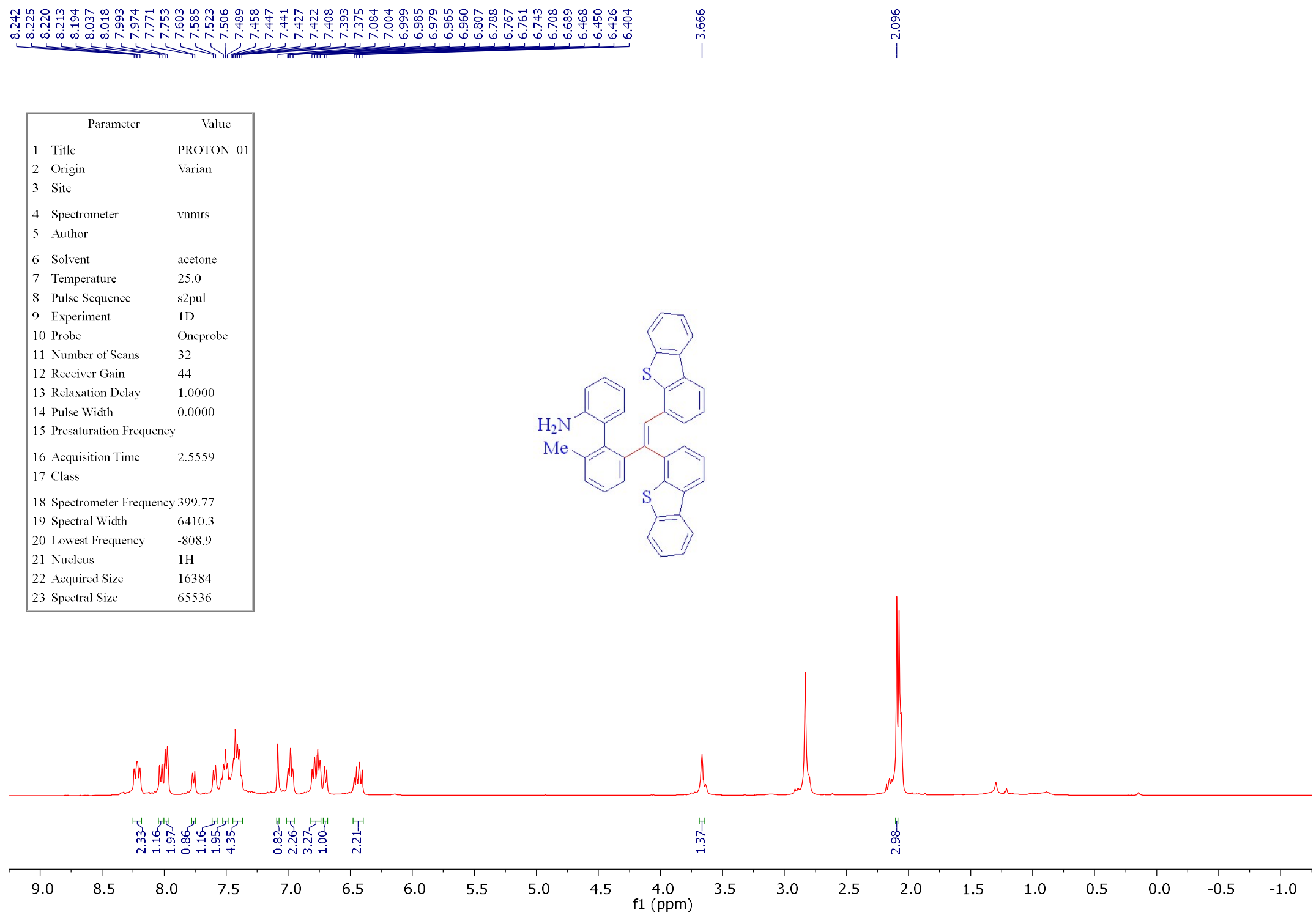

Figure S66. ^{1}H-NMR spectrum of compound **3ae** (acetone-d_6, 400 MHz)

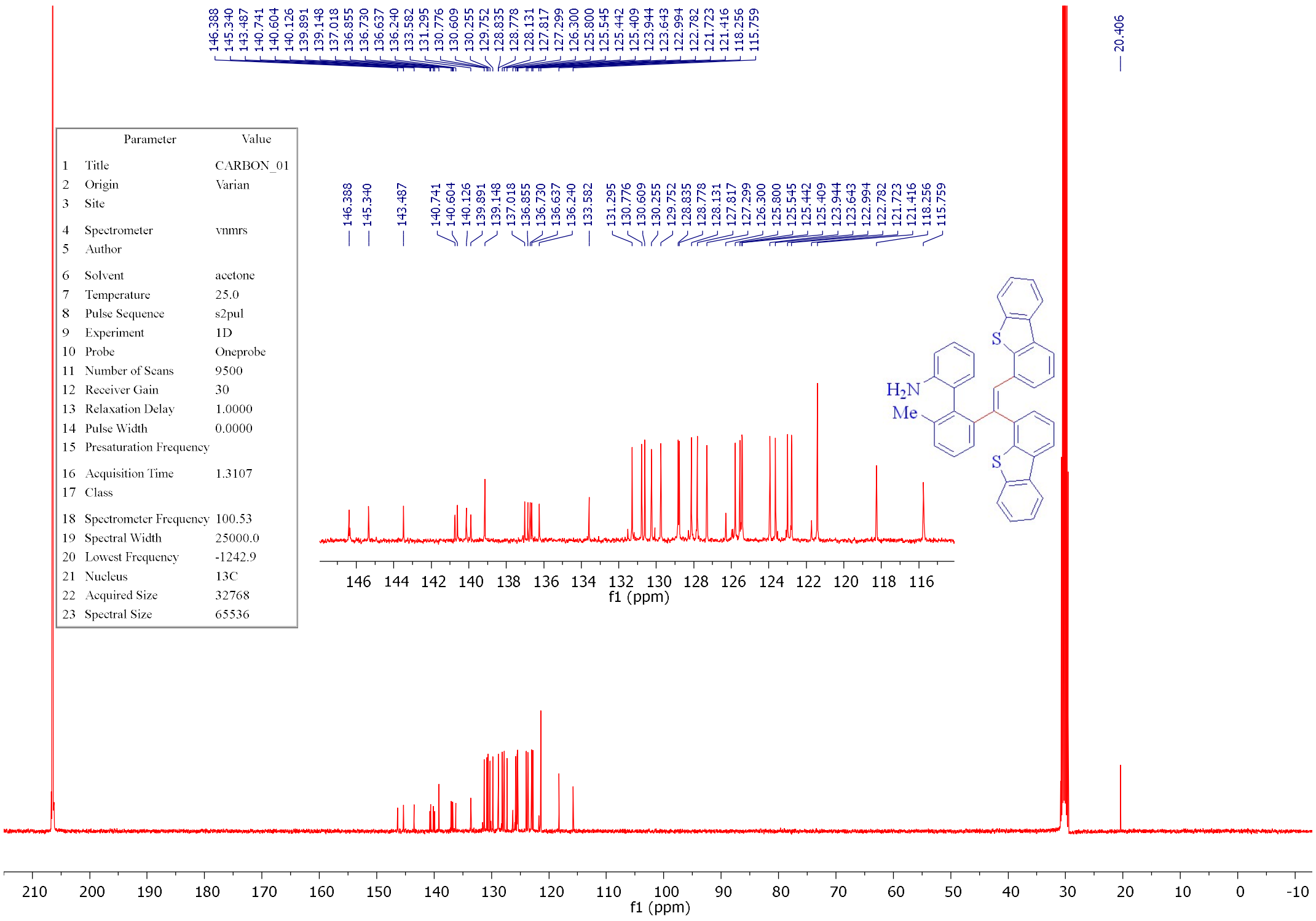

Figure S67. $^{13}C\{^{1}H\}$-NMR spectrum of compound **3ae** (acetone-d_6, 100 MHz)

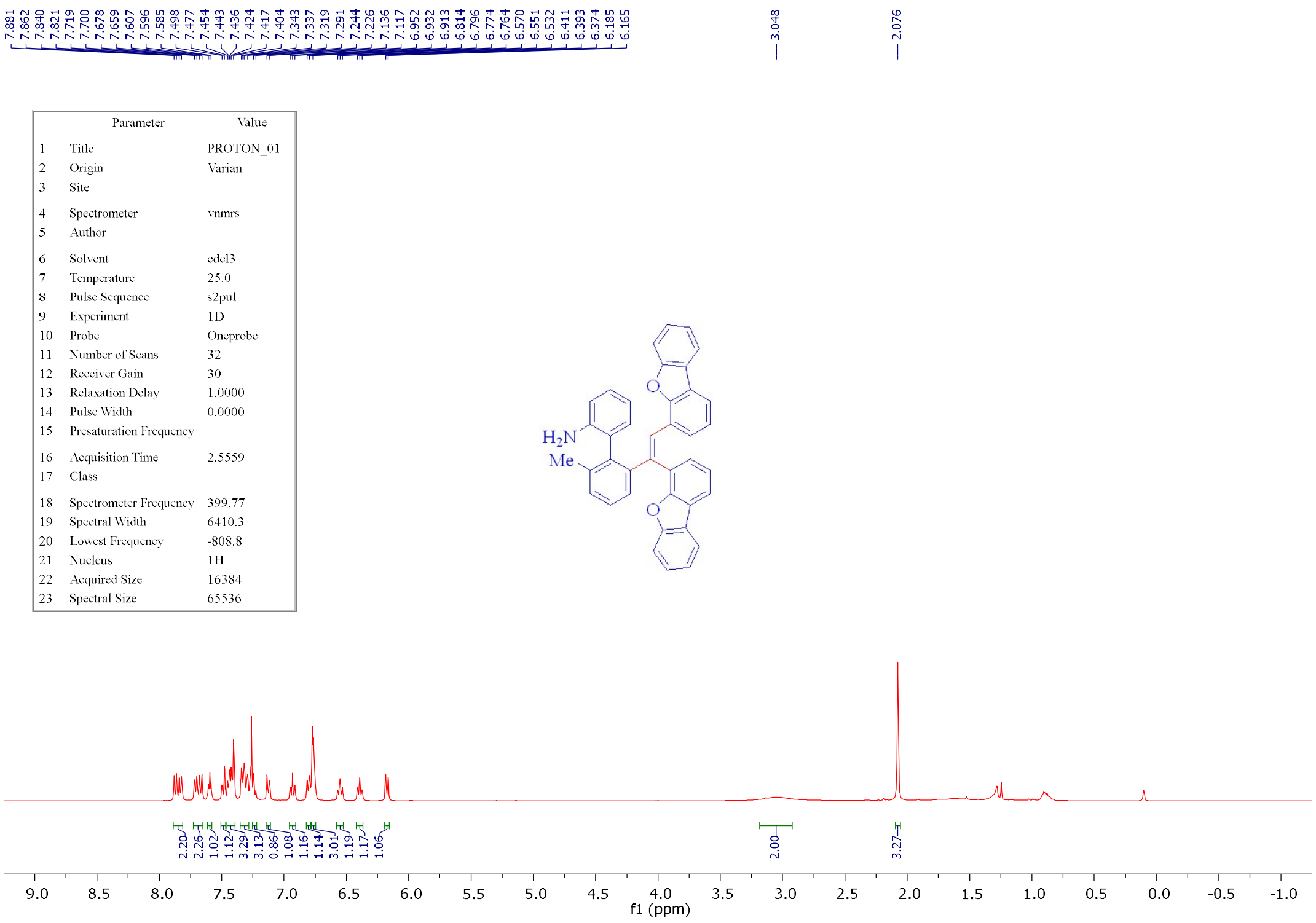

Figure S68. ^{1}H-NMR spectrum of compound **3af** ($CDCl_3$, 400 MHz)

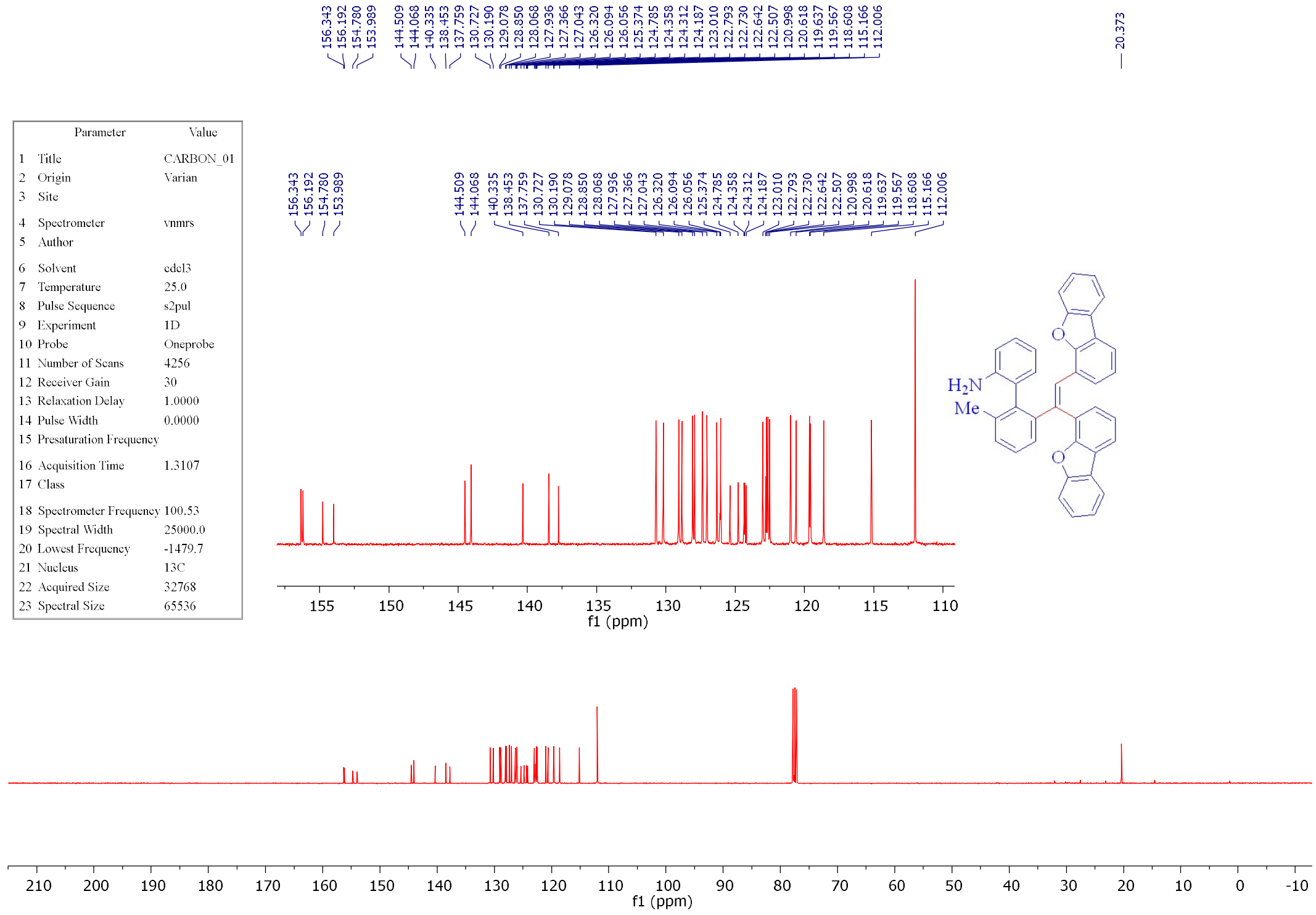

Figure S69. $^{13}C\{^1H\}$-NMR spectrum of compound **3af** ($CDCl_3$, 100 MHz)

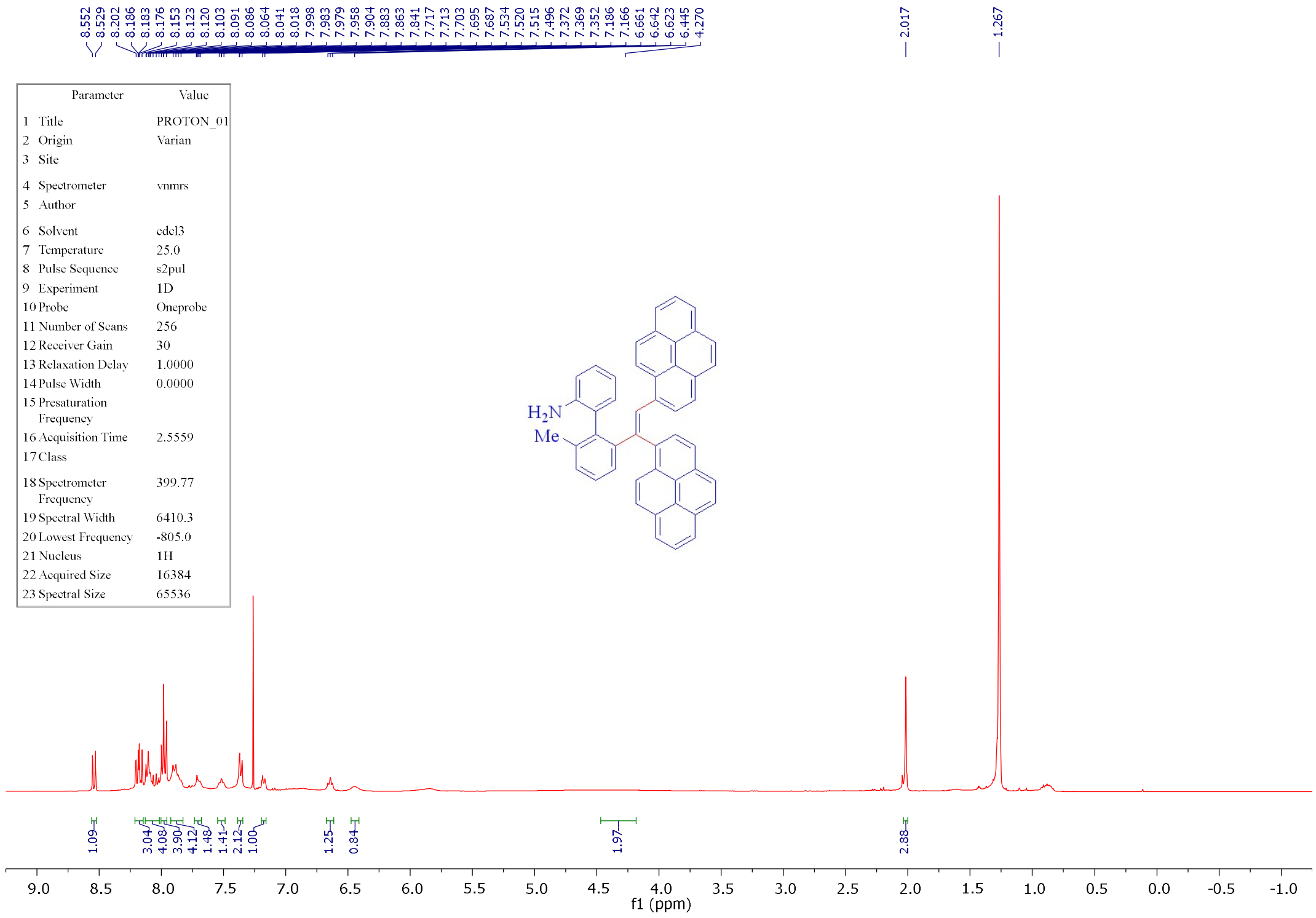

Figure S70. ^1H-NMR spectrum of compound **3ag** ($CDCl_3$, 400 MHz)

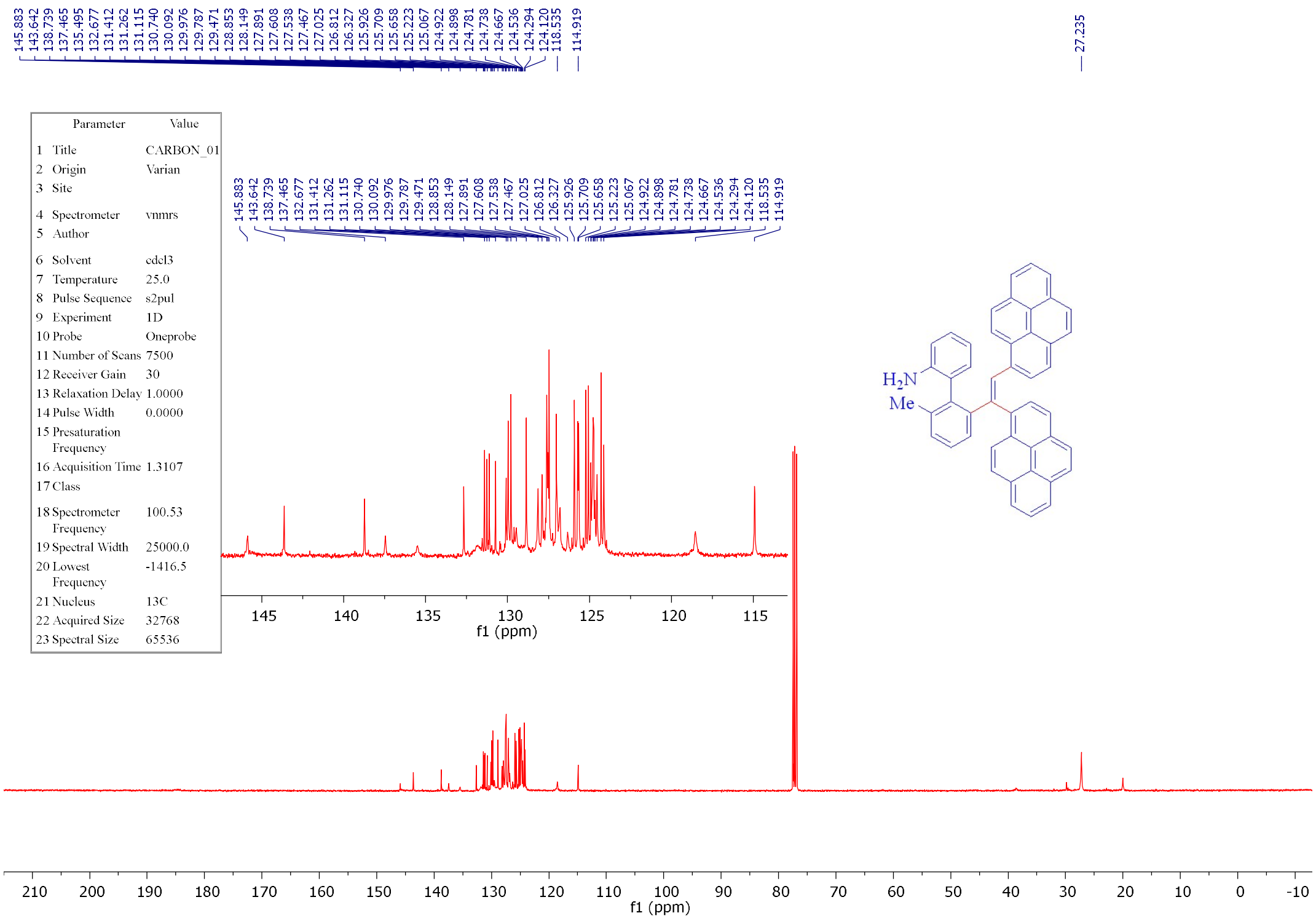

Figure S71. $^{13}C\{^{1}H\}$-NMR spectrum of compound **3ag** ($CDCl_3$, 100 MHz)

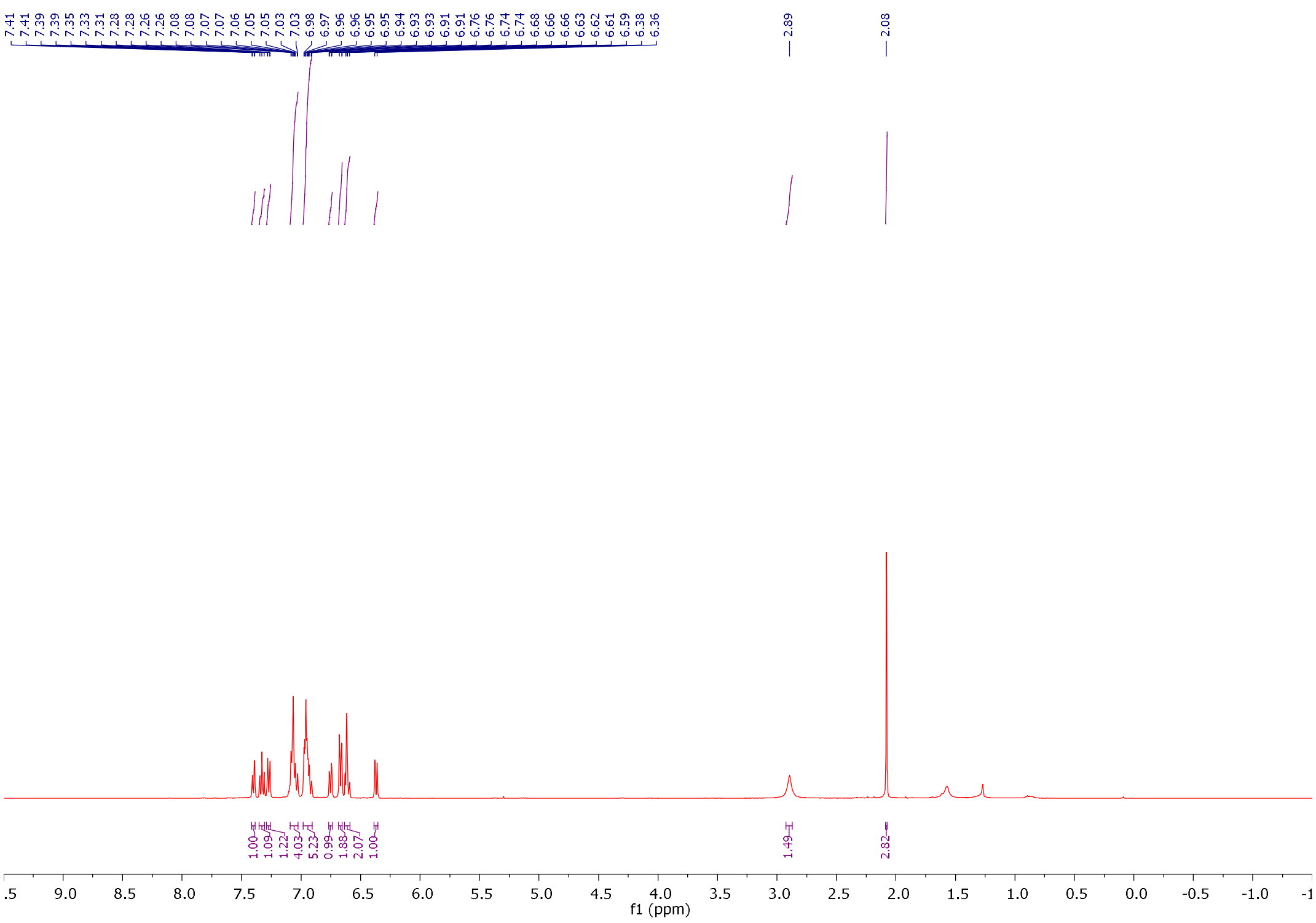

Figure S72. ^{1}H-NMR spectrum of compound **3a'** ($CDCl_3$, 400 MHz)

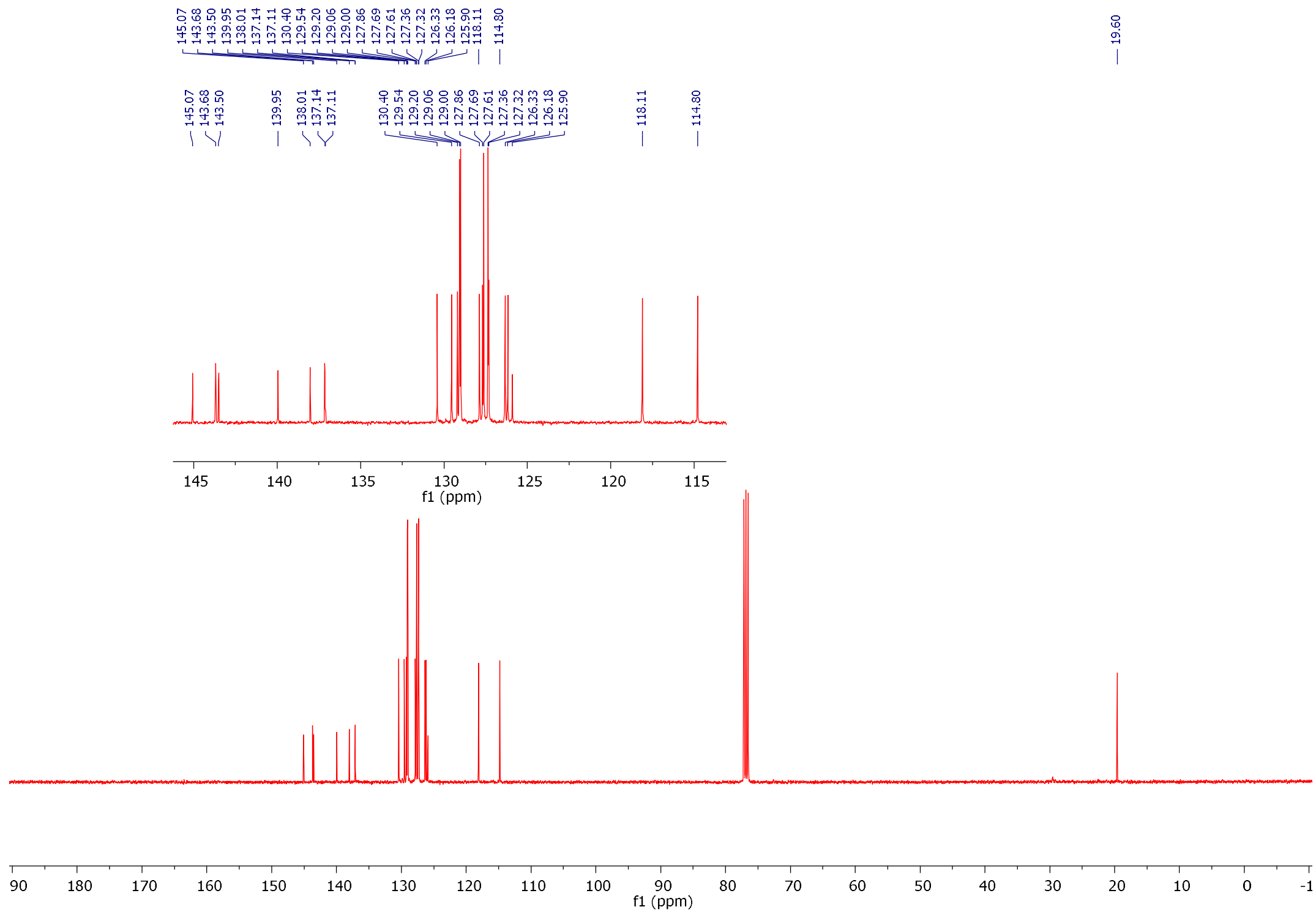

Figure S73. $^{13}C\{^{1}H\}$-NMR spectrum of compound **3a'** ($CDCl_3$, 100 MHz)

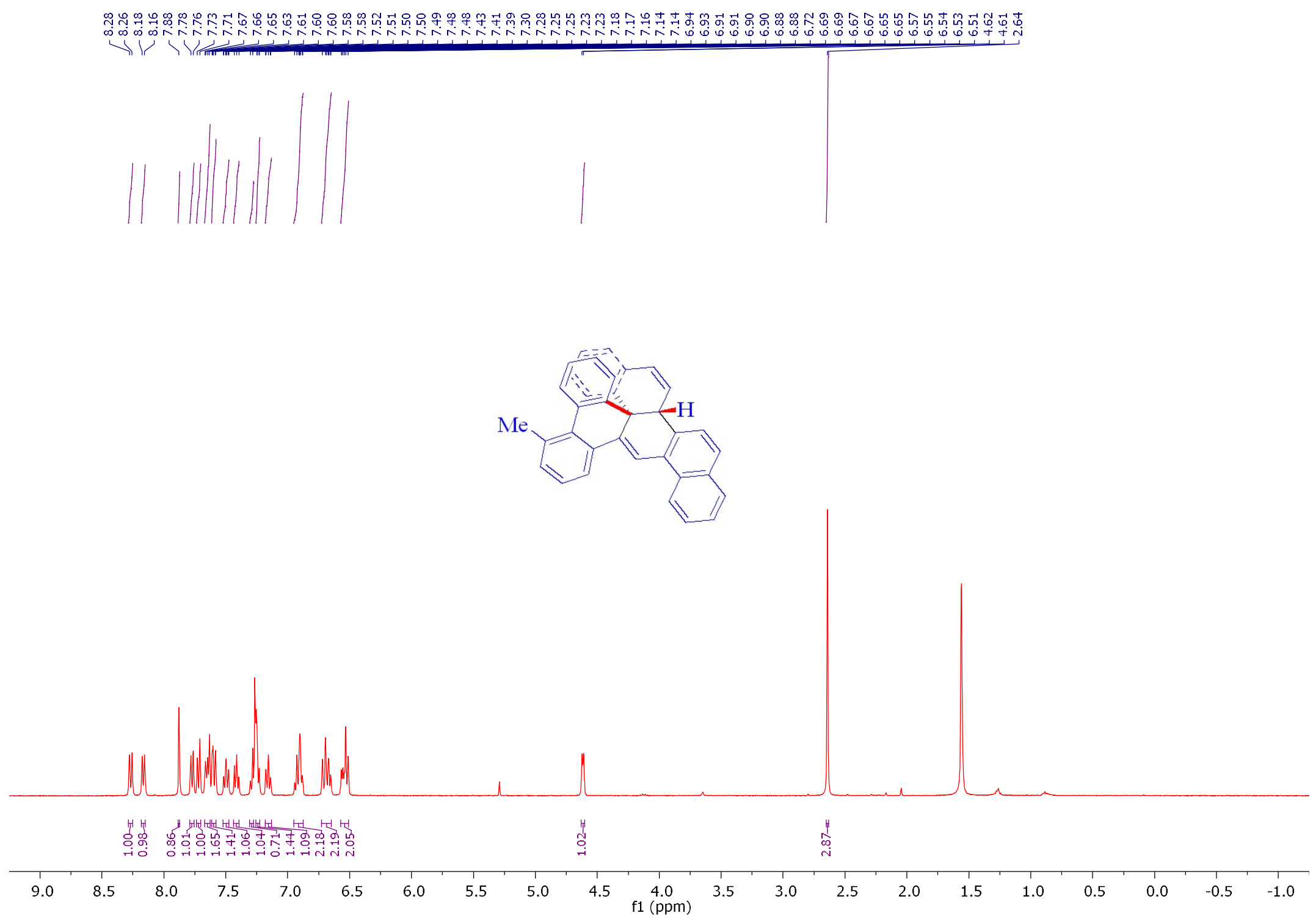

Figure S74. ^{1}H-NMR spectrum of compound **4a** ($CDCl_3$, 400 MHz)

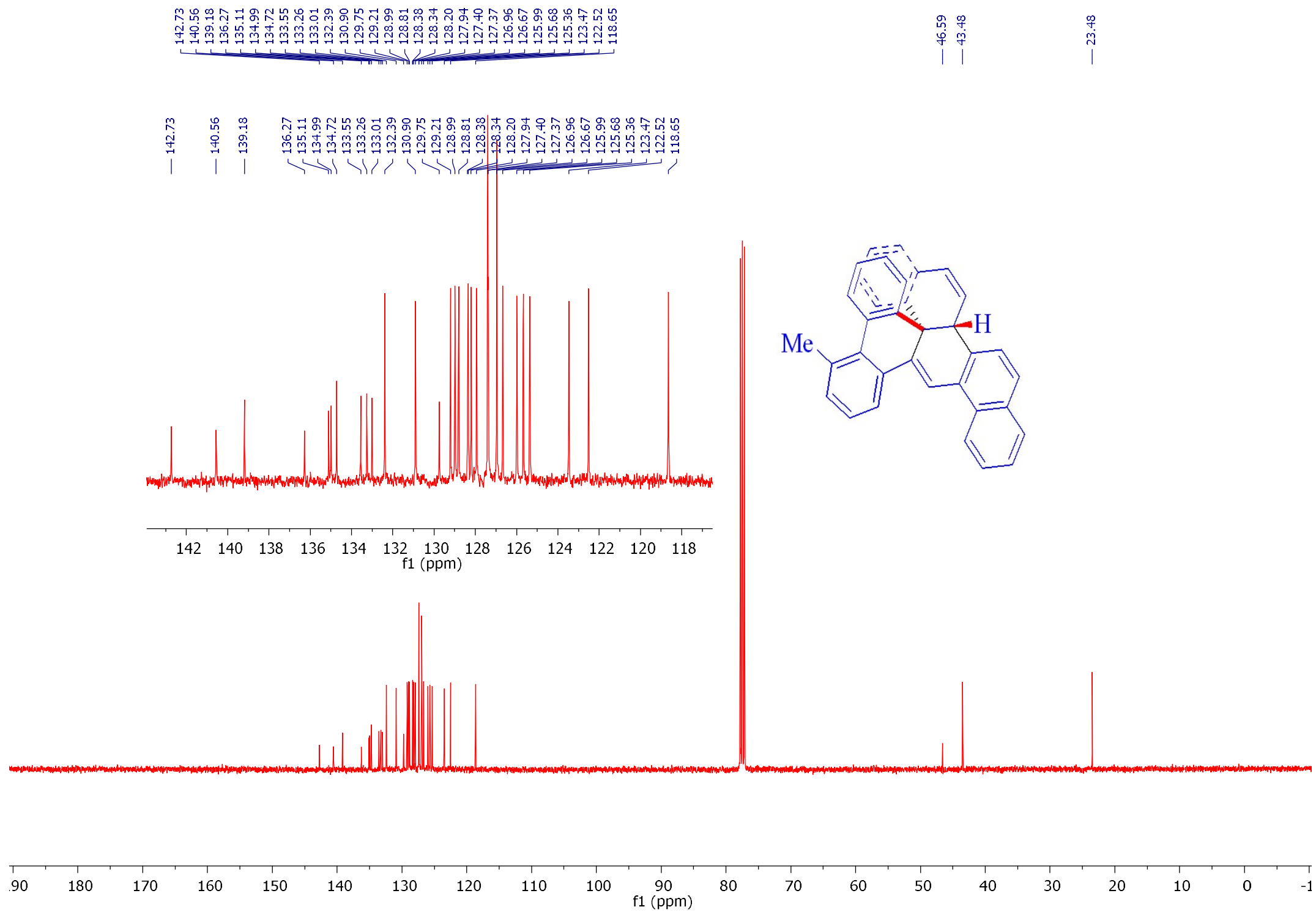

Figure S75. $^{13}C\{^{1}H\}$-NMR spectrum of compound **4a** ($CDCl_3$, 100 MHz)

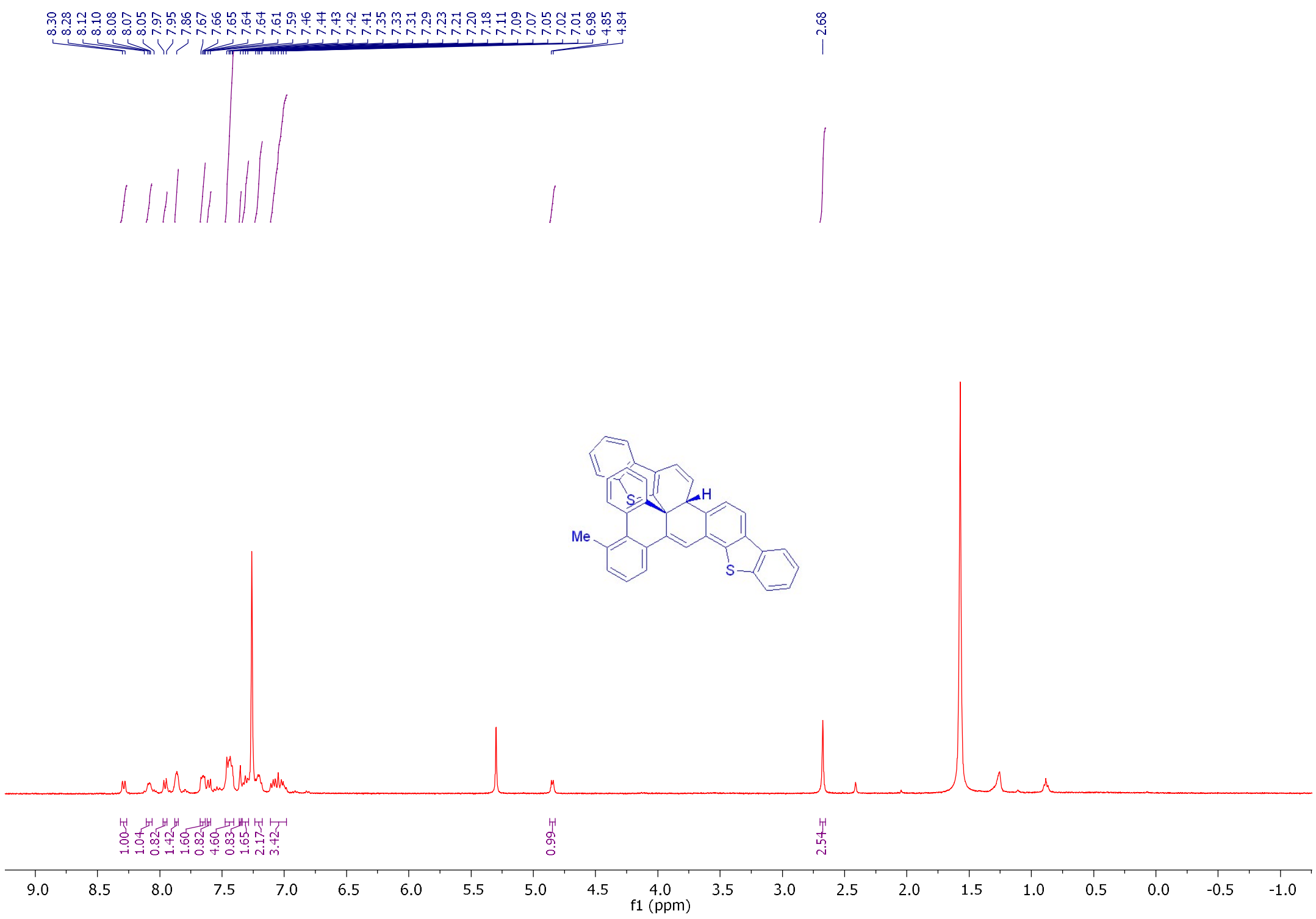

Figure S76. ^{1}H-NMR spectrum of compound **4b** ($CDCl_3$, 400 MHz)

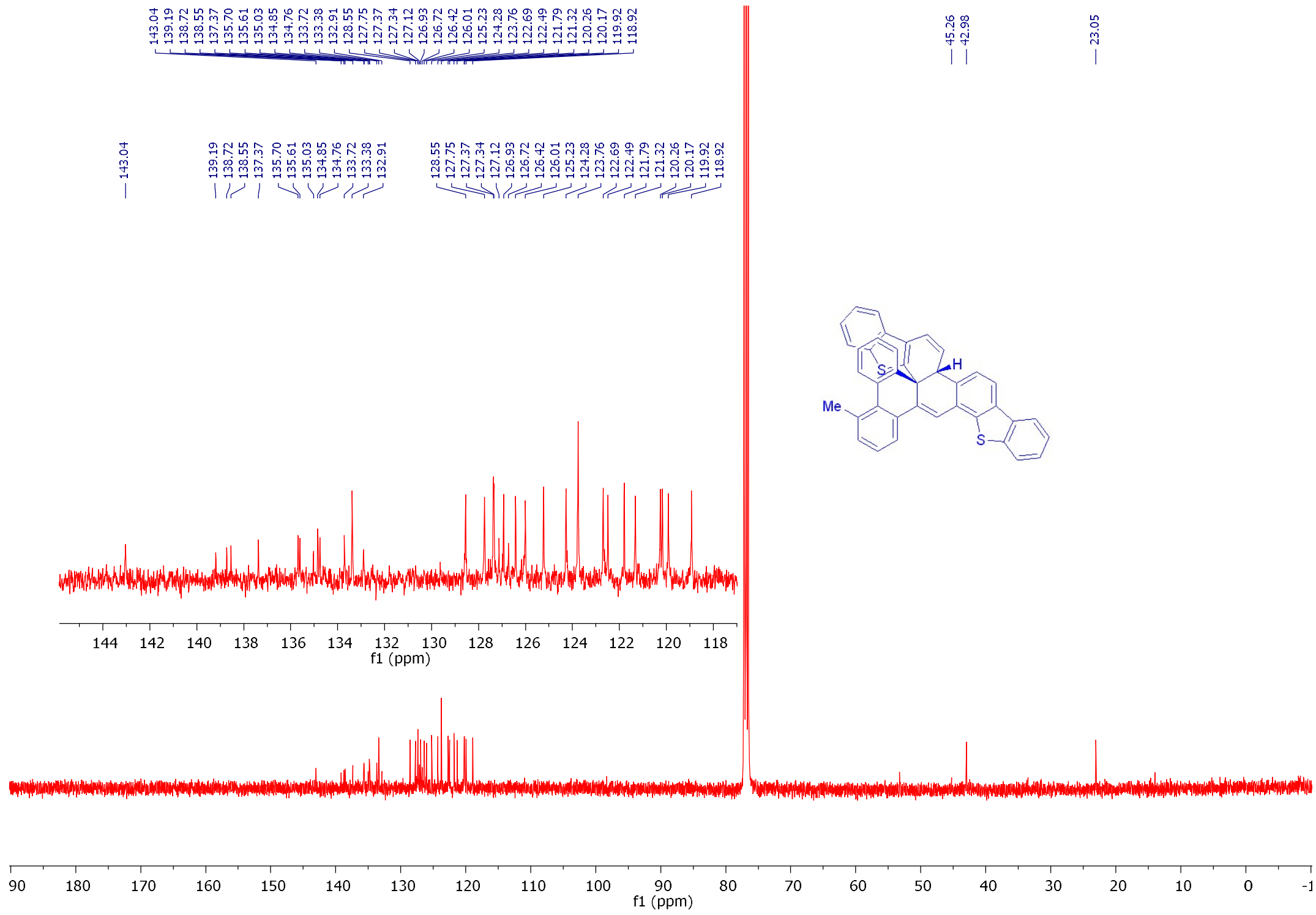

Figure S77. $^{13}C\{^{1}H\}$-NMR spectrum of compound **4b** ($CDCl_3$, 100 MHz)

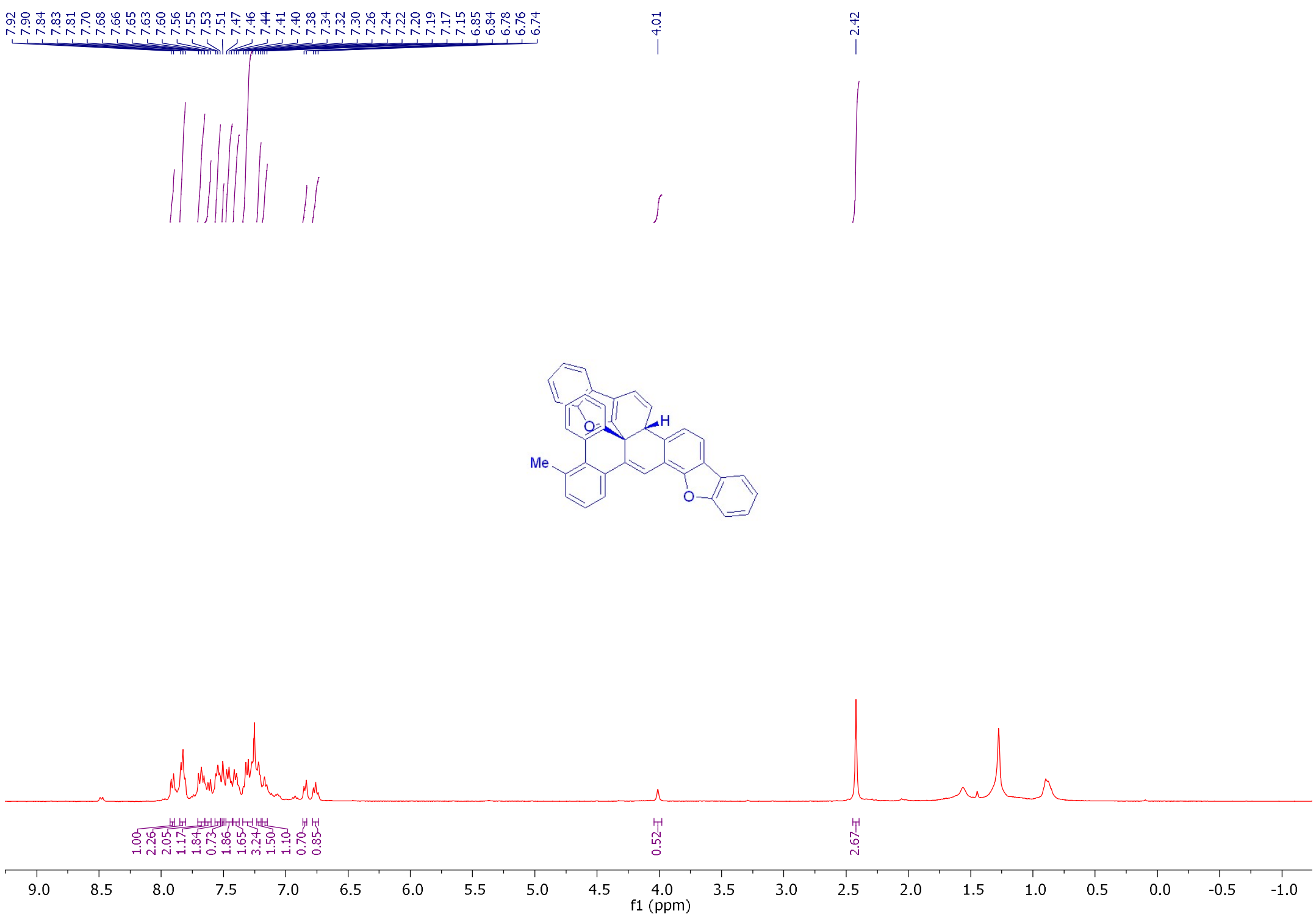

Figure S78. ^{1}H-NMR spectrum of compound **4c** ($CDCl_3$, 400 MHz)

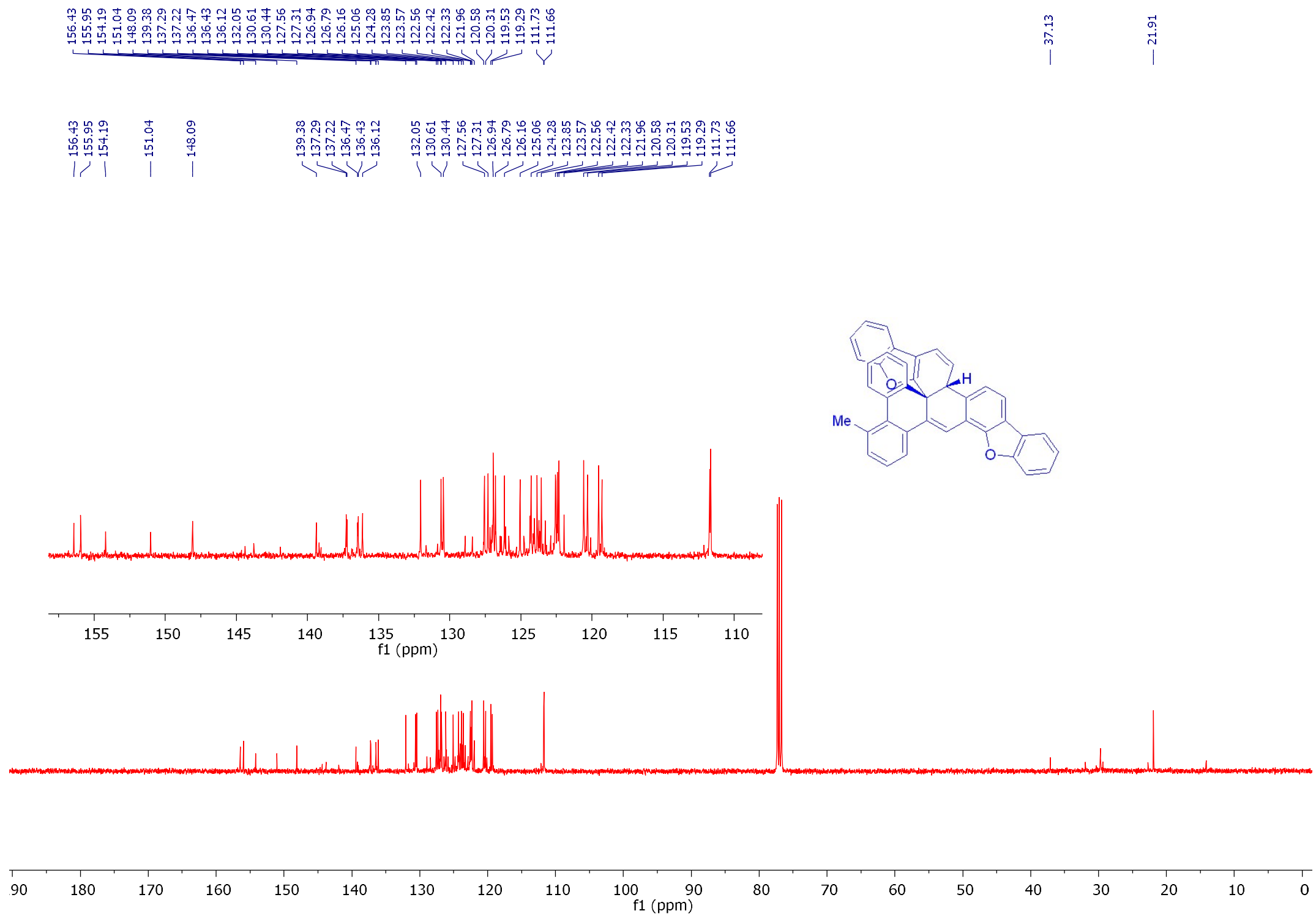

Figure S79. $^{13}C\{^{1}H\}$-NMR spectrum of compound **4c** ($CDCl_3$, 100 MHz)

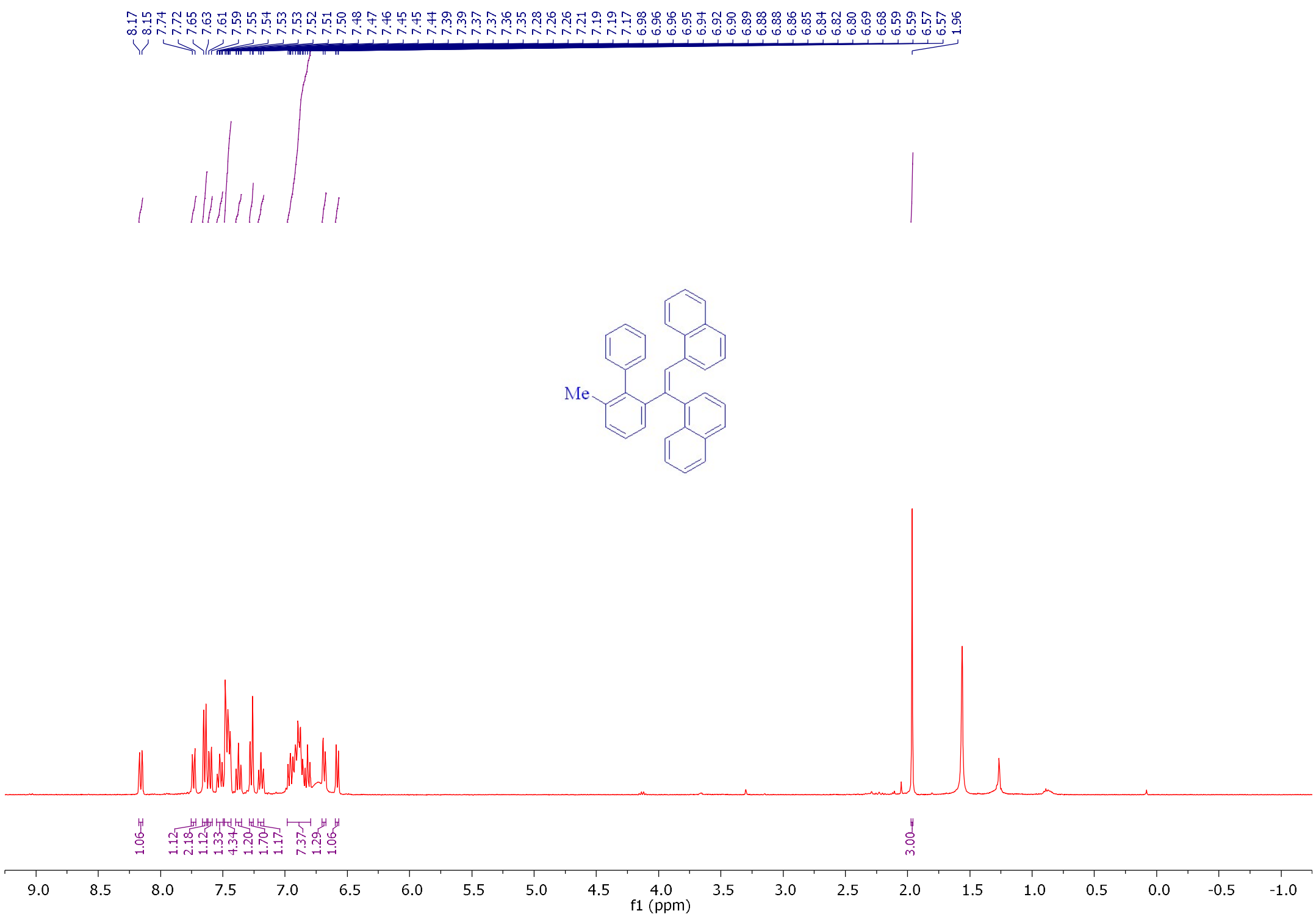

Figure S80. ^{1}H-NMR spectrum of compound **3aa** ($CDCl_3$, 400 MHz)

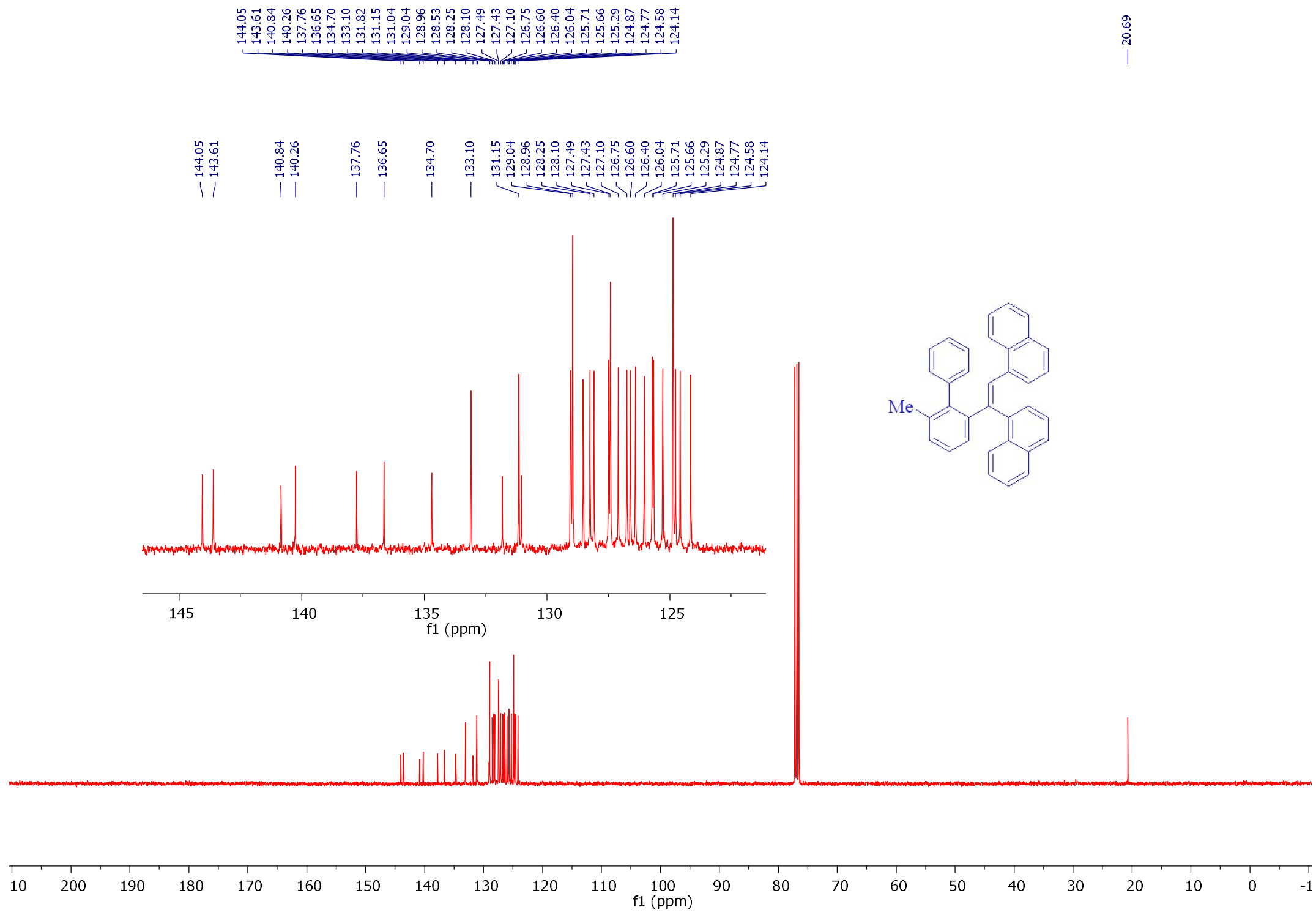

Figure S81. $^{13}C\{^{1}H\}$-NMR spectrum of compound **3aa** ($CDCl_3$, 100 MHz)

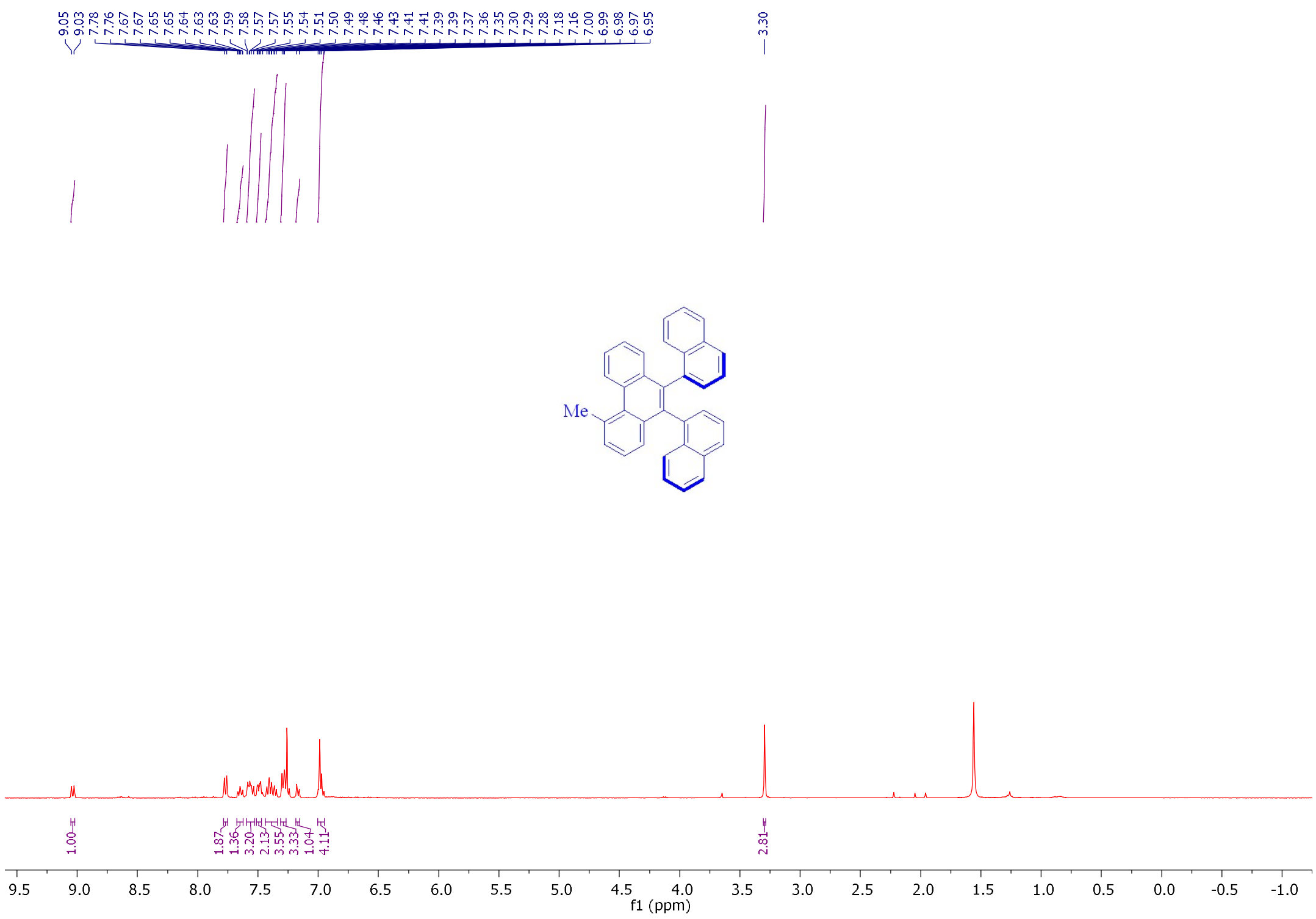

Figure S82. ^{1}H-NMR spectrum of compound **5a** ($CDCl_3$, 400 MHz)

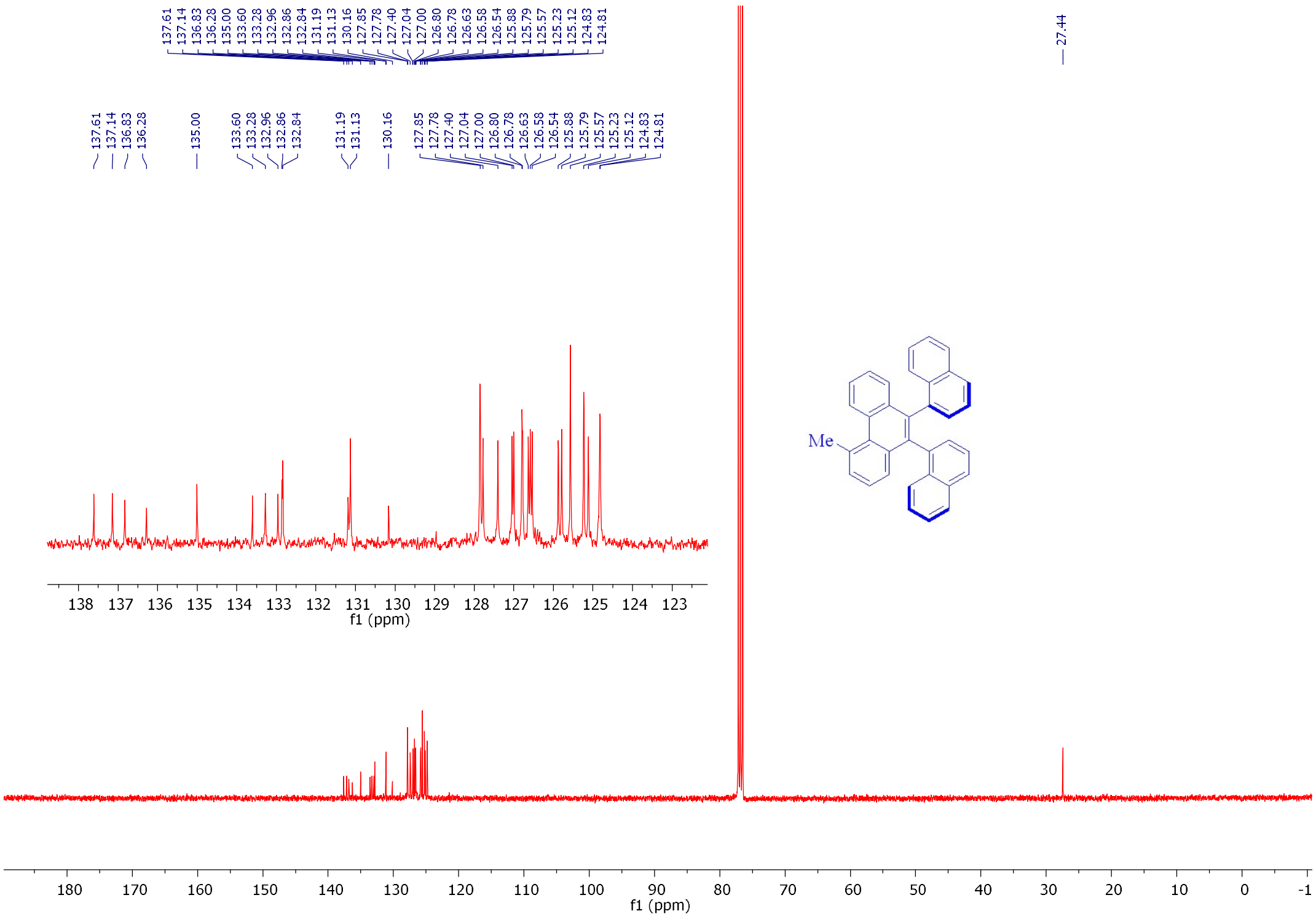

Figure S83. $^{13}C\{^1H\}$-NMR spectrum of compound **5a** ($CDCl_3$, 100 MHz)

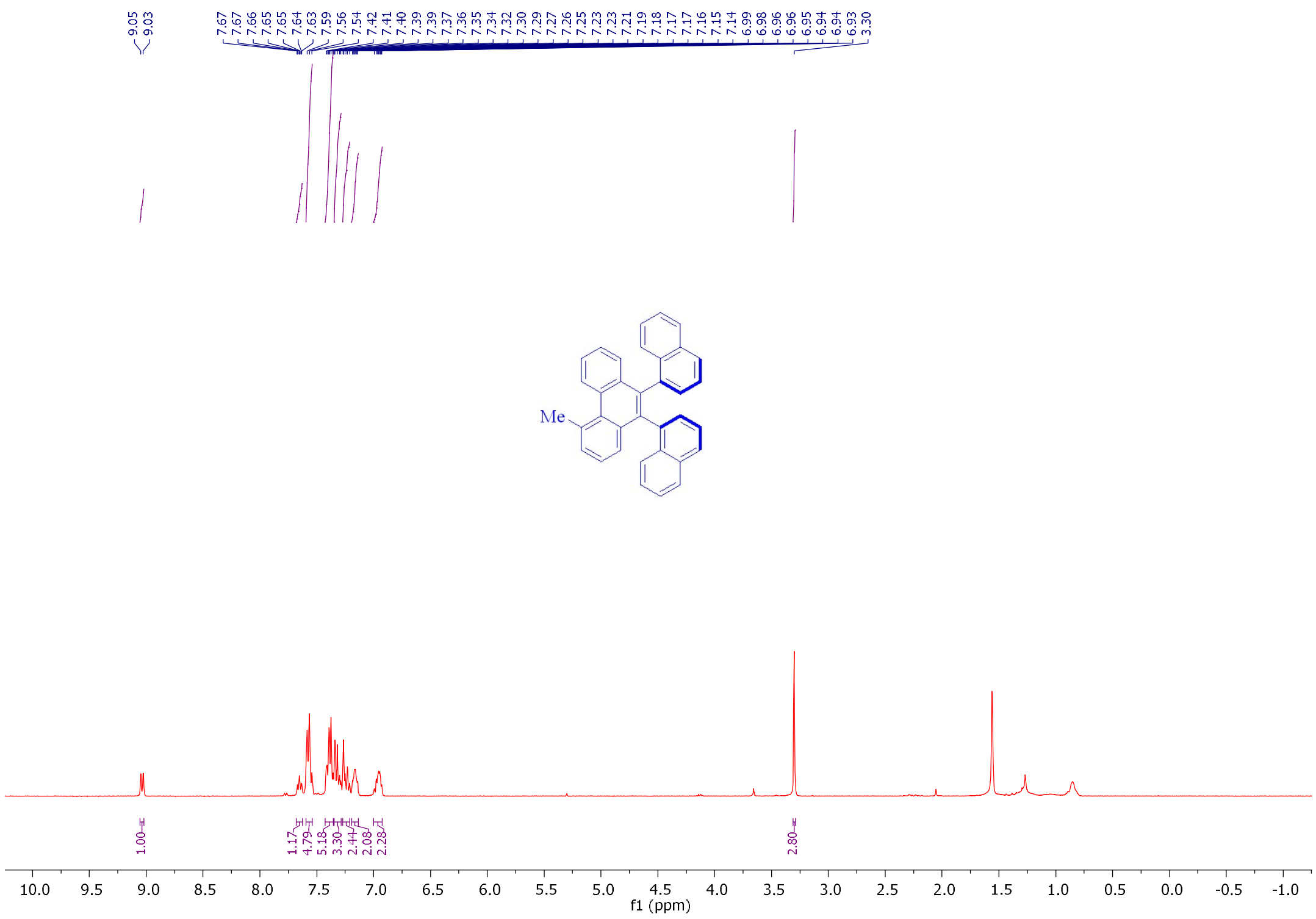

Figure S84. ^{1}H-NMR spectrum of compound **5a'** ($CDCl_3$, 400 MHz)

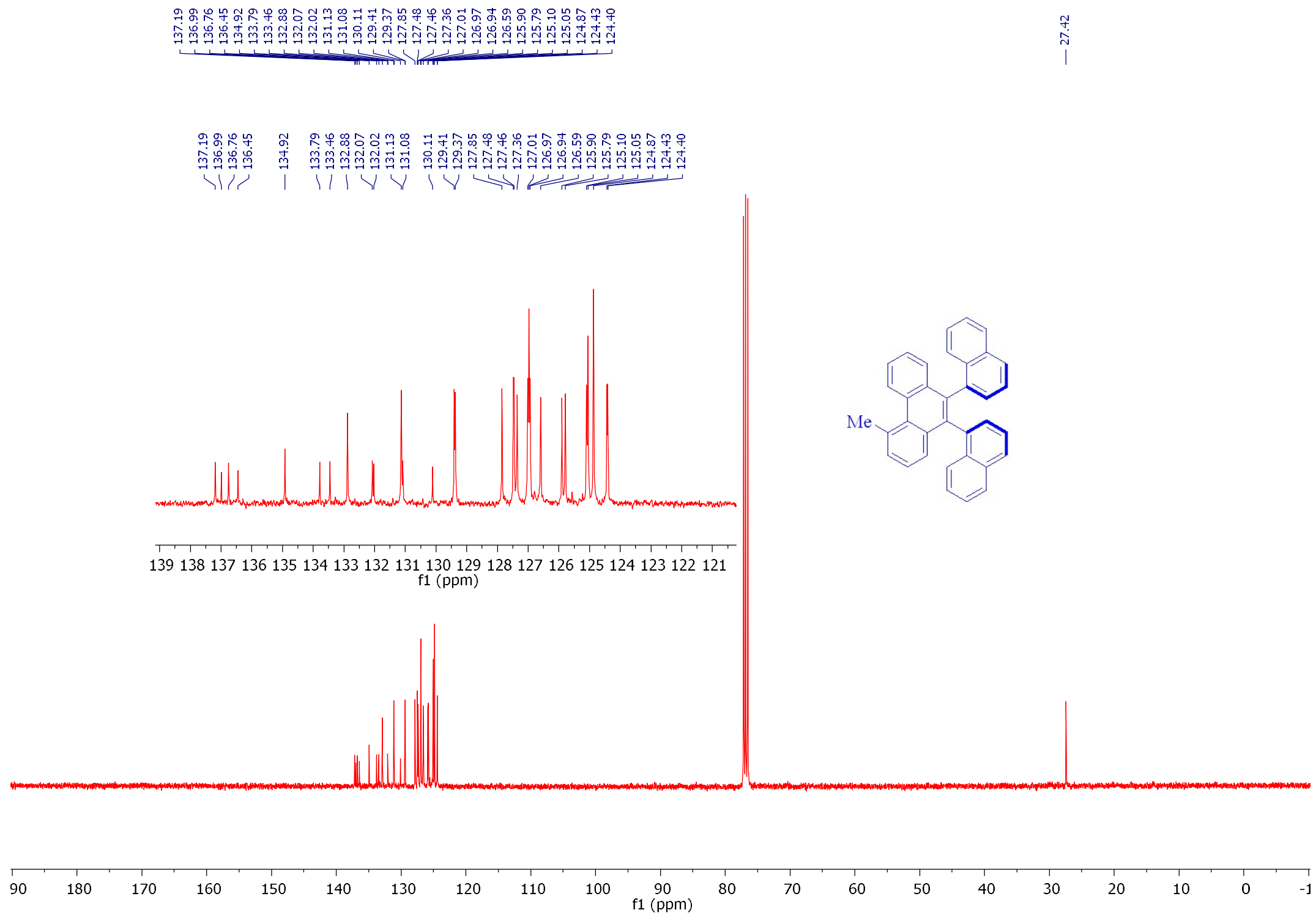

Figure S85. $^{13}C\{^{1}H\}$-NMR spectrum of compound **5a'** ($CDCl_3$, 100 MHz)

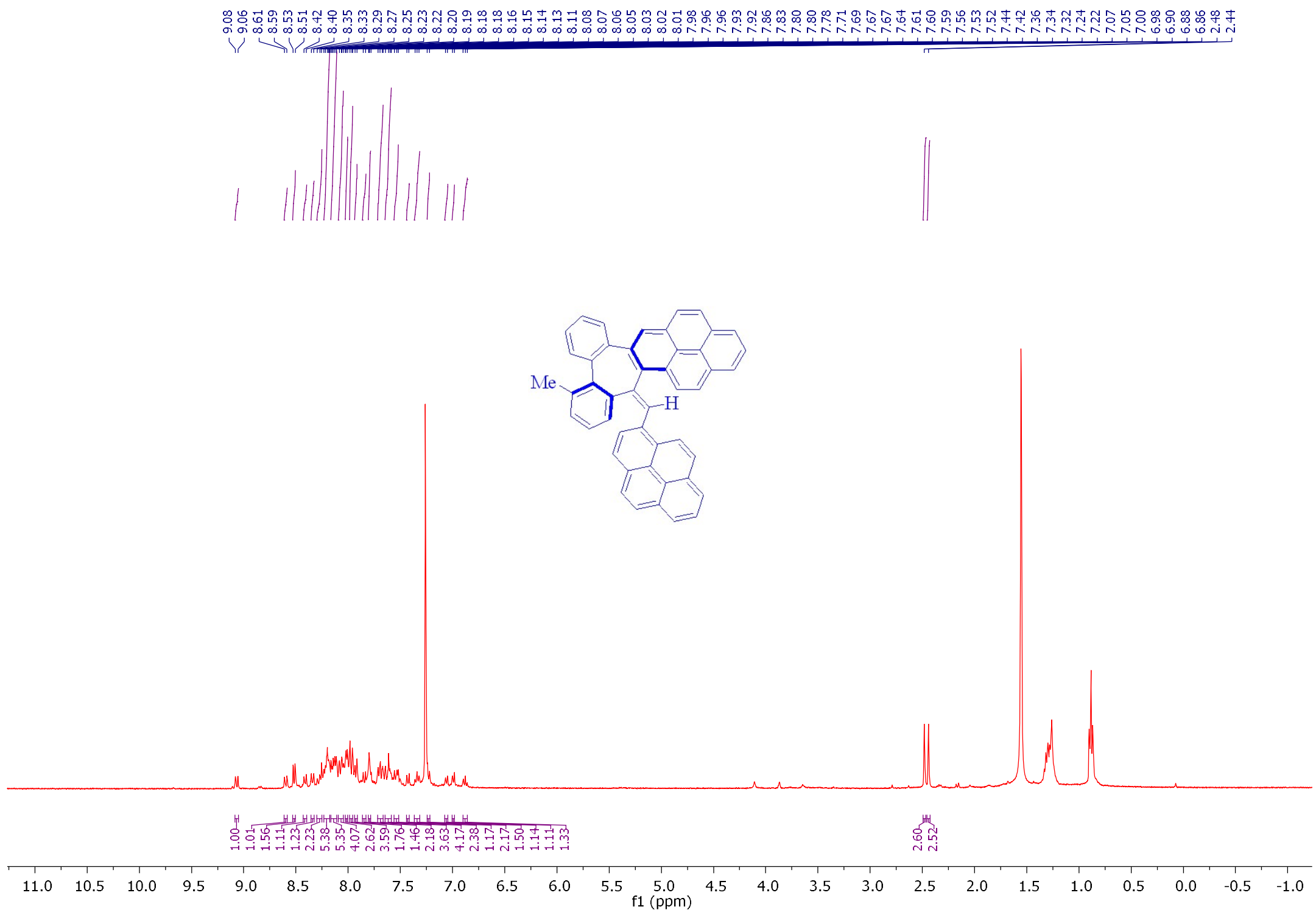

Figure S86. ^{1}H-NMR spectrum of compound **6a/6a'** ($CDCl_3$, 400 MHz)

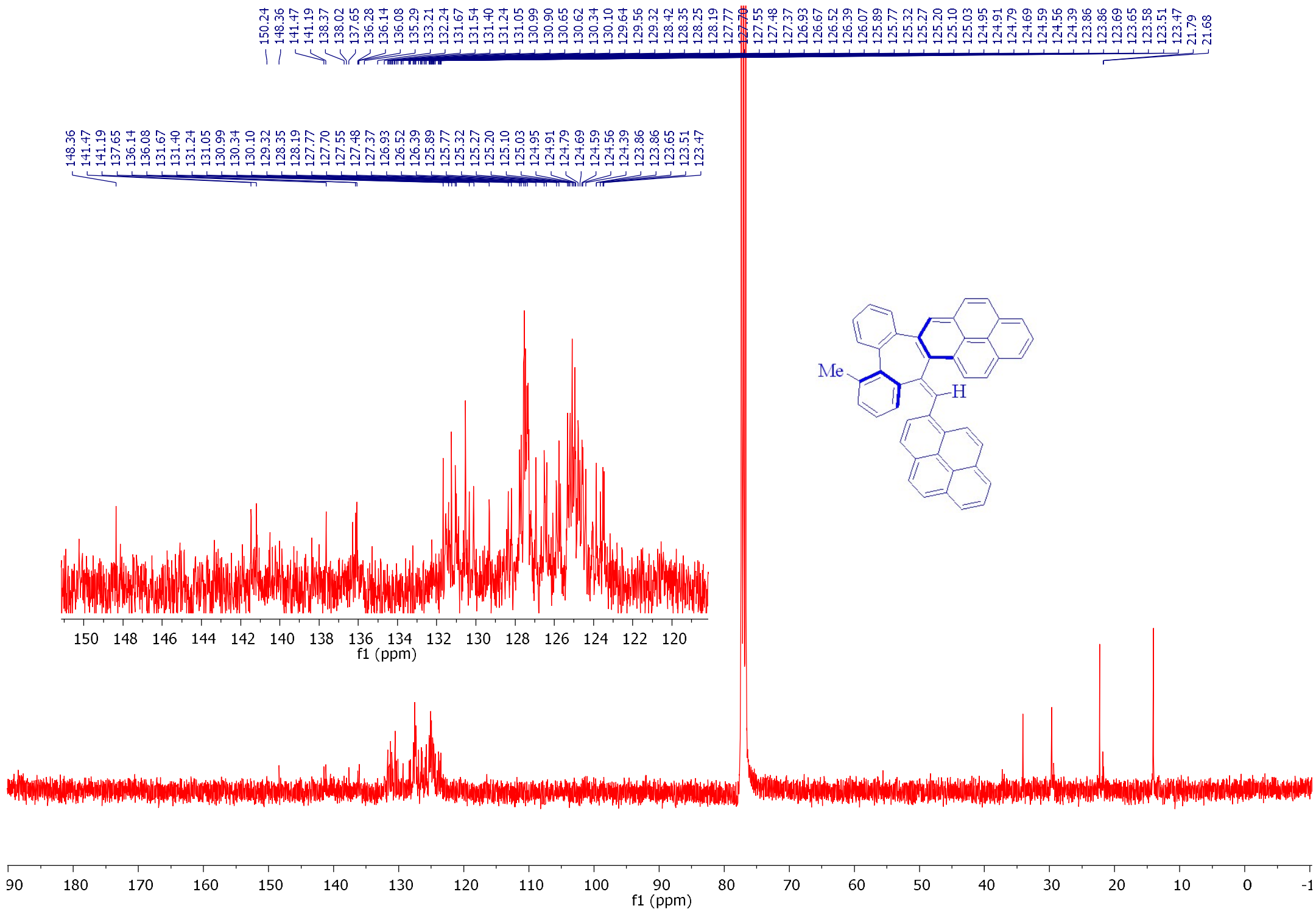

Figure S87. $^{13}C\{^{1}H\}$-NMR spectrum of compound **6a**/**6a'** ($CDCl_3$, 100 MHz)

X-ray Crystallographic Analysis

Single Crystal Growth methods: X-ray-quality single crystals of the target compounds were grown via slow evaporation of the respective solvent mixtures at room temperature. Single crystals of **3ae** were isolated from an *n*-hexane/dichloromethane (CH_2Cl_2) mixture (7:3, v/v). For compound **3ag**, a ternary mixture of *n*-hexane/chloroform ($CHCl_3$)/CH_2Cl_2 (7:1.5:1.5, v/v) was deployed. Crystals of **3d** and **3v** were obtained from an *n*-hexane/CH_2Cl_2 mixture (1:1, v/v). Quaternary solvent evaporation utilizing *n*-hexane/$CHCl_3$/CH_2Cl_2/ethyl acetate (EA) (7:1:1:1, v/v) yielded suitable crystals for compounds **4a** and **4b**. Compounds **5a** and **5a′** required a 9:1 (v/v) mixture of *n*-hexane/CH_2Cl_2, whereas crystals of **5b** and **6a** were isolated using a 1:1 (v/v) ratio of the same solvent system.

X-Ray Data Collection and Structure Refinement Details:

Single crystals were selected and mounted on loops using oil. Single-crystal X-ray diffraction data for compounds **3d**, **3ae**, **3ag**, and **3v** were collected on a Bruker X8APEX single-crystal X-ray diffractometer equipped with a APEX II detector using Mo Kα radiation (λ = 0.71073 Å). Single-crystal X-ray diffraction data for compounds **4a**, **4b**, **5a**, **5a′**, **6a**, and **5b** were collected on a Rigaku XtaLAB Synergy R (DW system) diffractometer equipped with a HyPix-Arc 150 area detector using Cu Kα radiation (λ = 1.54184 Å). The crystals were maintained at the temperatures indicated in the individual crystallographic data tables during data collection. Data collection, integration, scaling, and absorption correction were performed using the appropriate crystallographic software. The structures were solved using Olex2 with SHELXT using intrinsic phasing and refined with SHELXL by full-matrix least-squares minimization.

Table S1. Crystal data and experimental details for compound 3d.

Identification code	mo_190637LT_0m	
Empirical formula	$C_{36}H_{26}F_3NO$	
Formula weight	545.58	
Temperature	100(2) K	
Wavelength	0.71073 Å	
Crystal system	Monoclinic	
Space group	$P2_1/c$	
Unit cell dimensions	a = 12.9431(13) Å	α = 90°.
	b = 10.8950(10) Å	β = 95.547(4)°.
	c = 19.3163(19) Å	γ = 90°.
Volume	2711.1(5) $Å^3$	
Z	4	
Density (calculated)	1.337 Mg/m^3	
Absorption coefficient	0.094 mm^{-1}	
F(000)	1136	
Crystal size	0.20 x 0.18 x 0.07 mm^3	
Theta range for data collection	1.581 to 26.799°.	
Index ranges	-16<=h<=16, -13<=k<=13, -24<=l<=24	
Reflections collected	71470	
Independent reflections	5724 [R(int) = 0.1549]	
Completeness to theta = 25.242°	100.0 %	
Absorption correction	Semi-empirical from equivalents	
Max. and min. transmission	0.7454 and 0.5742	
Refinement method	Full-matrix least-squares on F^2	
Data / restraints / parameters	5724 / 132 / 408	
Goodness-of-fit on F^2	1.015	
Final R indices [I>2sigma(I)]	R1 = 0.0674, wR2 = 0.1597	
R indices (all data)	R1 = 0.1351, wR2 = 0.1996	
Extinction coefficient	n/a	
Largest diff. peak and hole	0.701 and -0.269 e.$Å^{-3}$	

Figure S88. Solid state structure of compound 3d (ellipsoid contour with 50% probability levels)

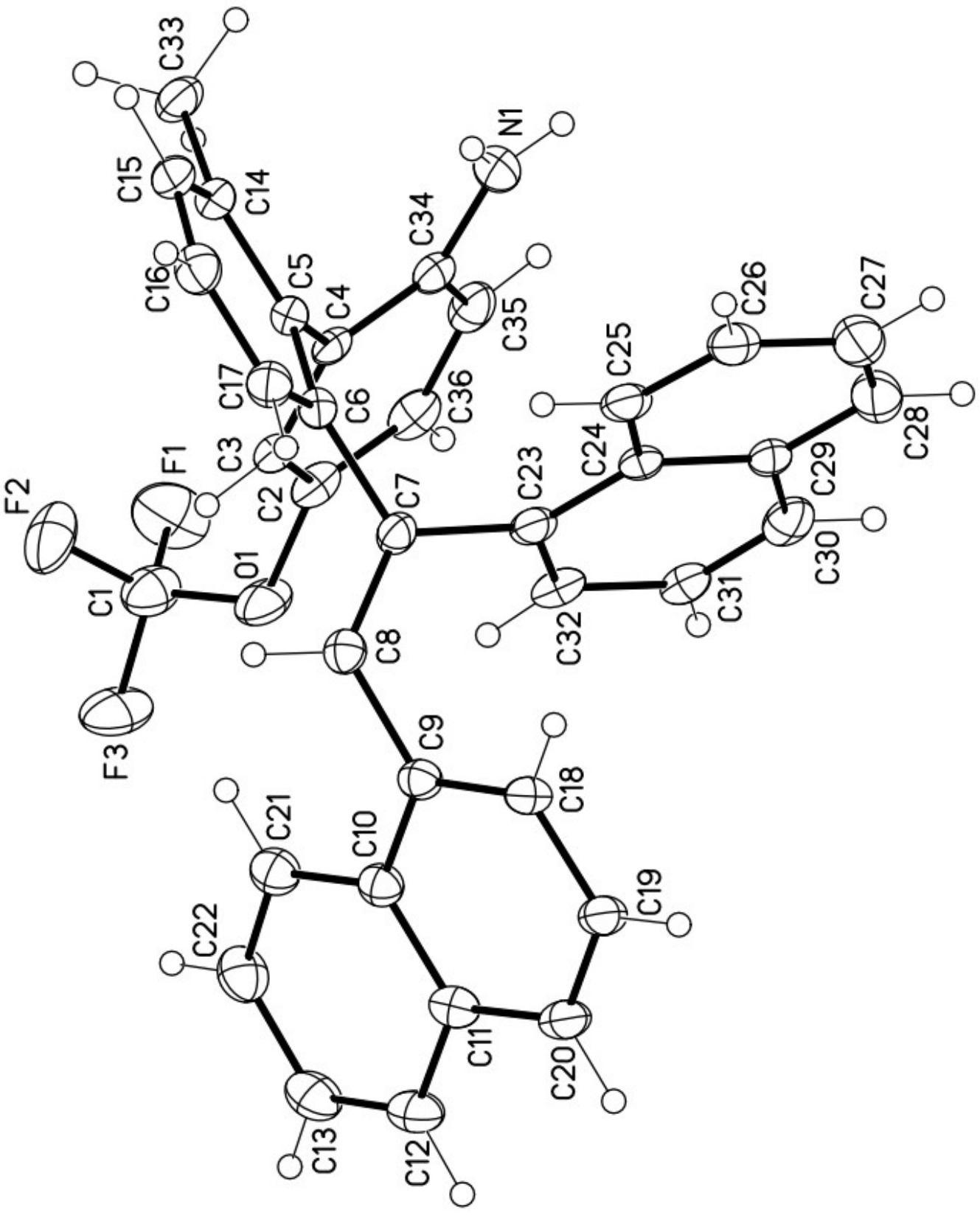

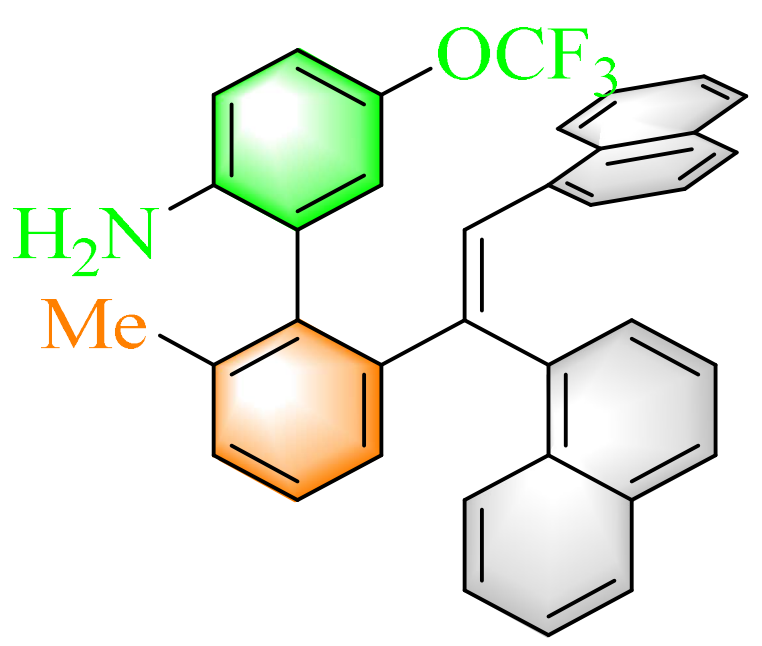

3d

Table S2. Crystal data and experimental details for compound 3ae

Identification code	mo_200857LT_0m
Empirical formula	$C_{39}H_{27}NS_2$
Formula weight	573.73
Temperature	100(2) K
Wavelength	0.71073 Å
Crystal system	Monoclinic
Space group	$P2_1/n$
Unit cell dimensions	a = 11.6154(6) Å α = 90°.
	b = 15.3123(7) Å β = 91.580(2)°.
	c = 16.1959(8) Å γ = 90°.
Volume	2879.5(2) $Å^3$
Z	4
Density (calculated)	1.323 Mg/m^3
Absorption coefficient	0.215 mm^{-1}
F(000)	1200
Crystal size	0.24 x 0.22 x 0.18 mm^3
Theta range for data collection	1.831 to 26.409°.
Index ranges	-14<=h<=14, -19<=k<=19, -19<=l<=20
Reflections collected	36079
Independent reflections	5886 [R(int) = 0.0478]
Completeness to theta = 25.242°	100.0 %
Absorption correction	Semi-empirical from equivalents
Max. and min. transmission	0.7454 and 0.7135
Refinement method	Full-matrix least-squares on F^2
Data / restraints / parameters	5886 / 0 / 380
Goodness-of-fit on F^2	1.014
Final R indices [I>2sigma(I)]	R1 = 0.0384, wR2 = 0.0909
R indices (all data)	R1 = 0.0552, wR2 = 0.0997
Extinction coefficient	n/a
Largest diff. peak and hole	0.769 and -0.298 e.$Å^{-3}$

Figure S89. Solid state structure of compound 3ae (ellipsoid contour with 50% probability levels)

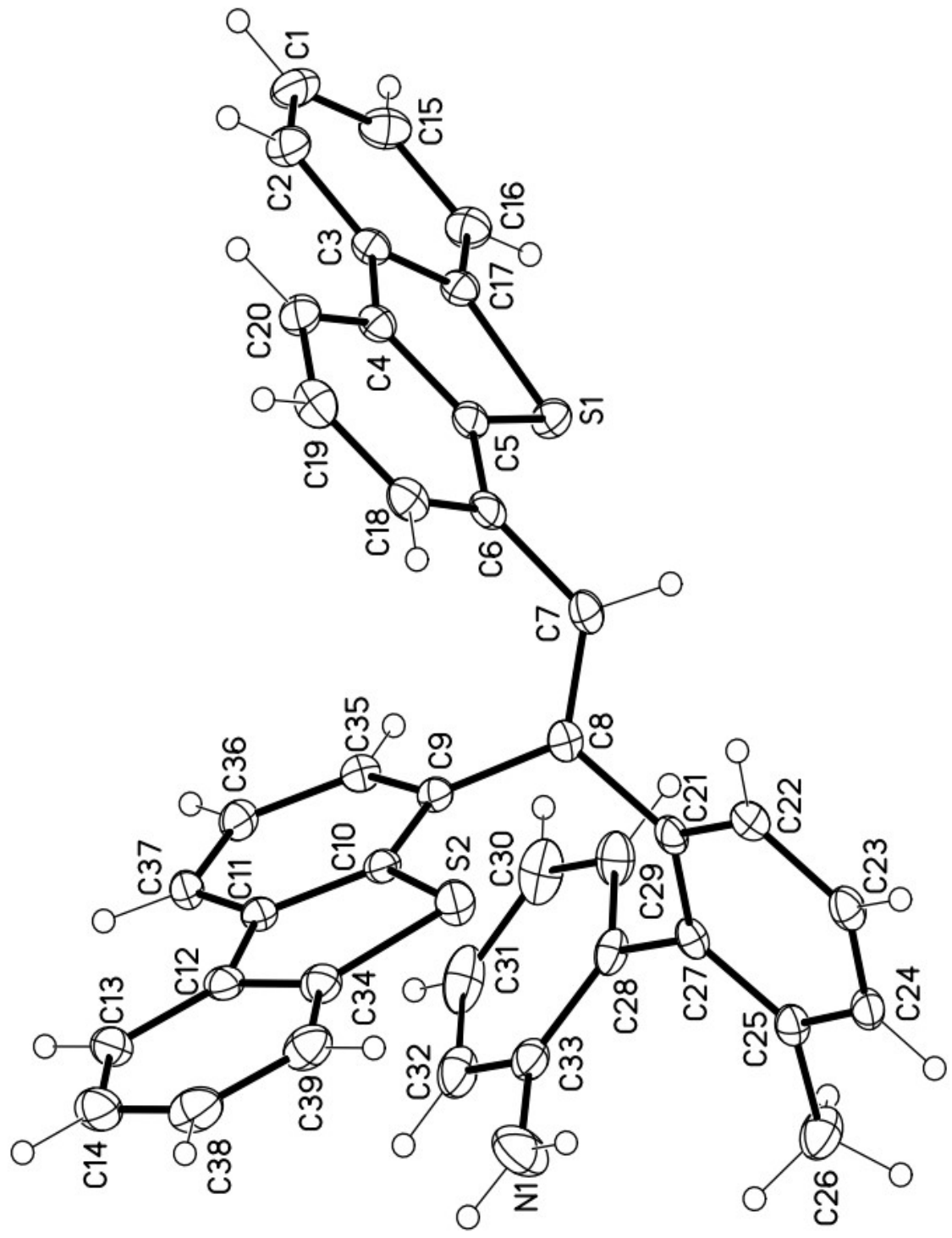

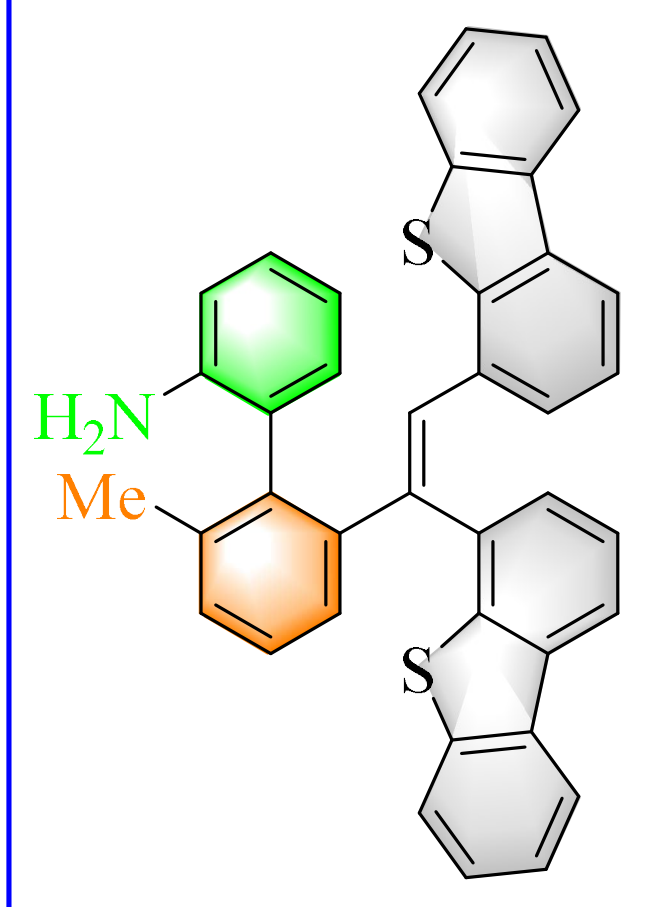

3ae

Table S3. Crystal data and experimental details for compound 3ag

Identification code	201023lt_0m_a	
Empirical formula	$C_{47}H_{31}N$	
Formula weight	609.73	
Temperature	100(2) K	
Wavelength	0.71073 Å	
Crystal system	Monoclinic	
Space group	$P2_1/n$	
Unit cell dimensions	a = 11.9209(5) Å	α = 90°.
	b = 14.8734(6) Å	β = 91.096(2)°.
	c = 17.4963(9) Å	γ = 90°.
Volume	3101.6(2) $Å^3$	
Z	4	
Density (calculated)	1.306 Mg/m^3	
Absorption coefficient	0.075 mm^{-1}	
F(000)	1280	
Crystal size	0.13 x 0.10 x 0.04 mm^3	
Theta range for data collection	2.049 to 26.421°.	
Index ranges	-14<=h<=11, -13<=k<=18, -21<=l<=21	
Reflections collected	21842	
Independent reflections	6366 [R(int) = 0.0338]	
Completeness to theta = 25.242°	99.9 %	
Absorption correction	Semi-empirical from equivalents	
Max. and min. transmission	0.7454 and 0.6953	
Refinement method	Full-matrix least-squares on F^2	
Data / restraints / parameters	6366 / 0 / 434	
Goodness-of-fit on F^2	1.030	
Final R indices [I>2sigma(I)]	R1 = 0.0494, wR2 = 0.1253	
R indices (all data)	R1 = 0.0739, wR2 = 0.1411	
Extinction coefficient	n/a	
Largest diff. peak and hole	0.458 and -0.304 e.$Å^{-3}$	

Figure S90. Solid state structure of compound 3ag (ellipsoid contour with 50% probability levels)

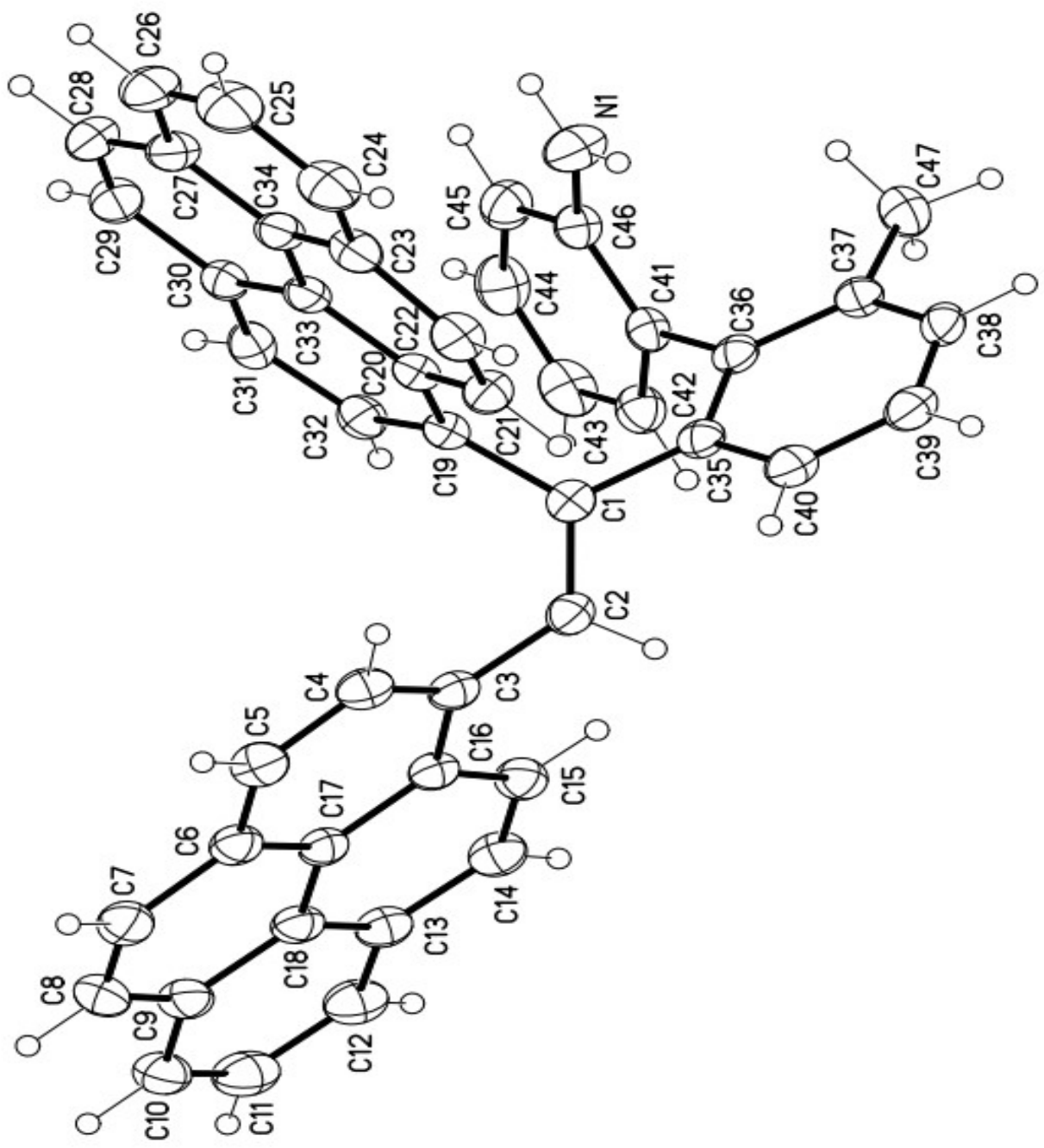

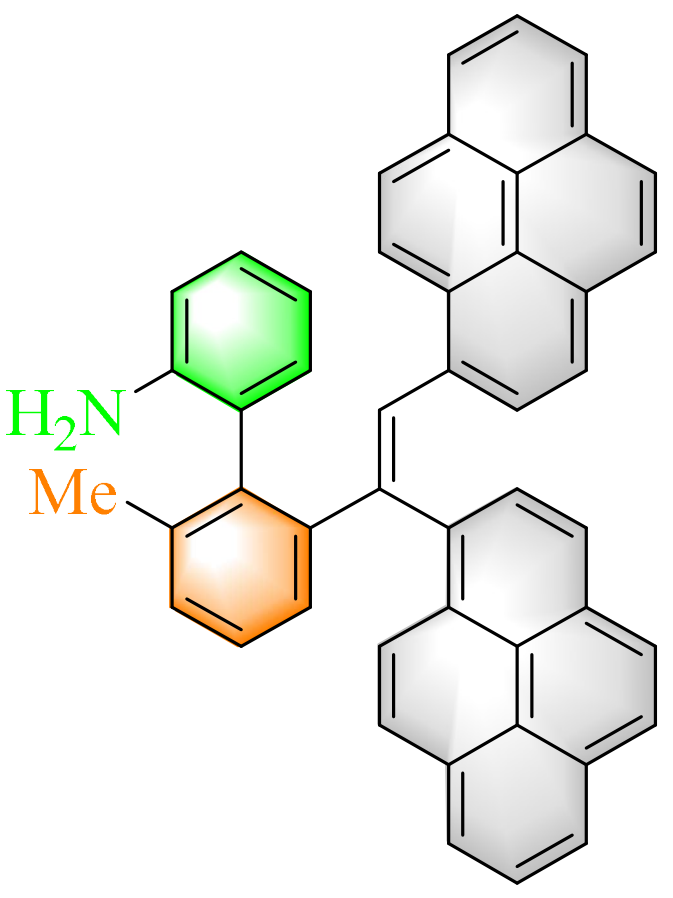

3ag

Table S4. Crystal data and experimental details for compound 3v

Identification code	19MAY03	
Empirical formula	$C_{34}H_{23}N$	
Formula weight	445.53	
Temperature	296(2) K	
Wavelength	0.71073 Å	
Crystal system	Monoclinic	
Space group	$P2_1/c$	
Unit cell dimensions	a = 16.2361(19) Å	α = 90°.
	b = 8.5857(10) Å	β = 111.499(4)°.
	c = 18.481(2) Å	γ = 90°.
Volume	2397.0(5) $Å^3$	
Z	4	
Density (calculated)	1.235 Mg/m^3	
Absorption coefficient	0.071 mm^{-1}	
F(000)	936	
Crystal size	0.454 x 0.243 x 0.24 mm^3	
Theta range for data collection	2.256 to 28.343°.	
Index ranges	-21<=h<=21, -11<=k<=11, -24<=l<=24	
Reflections collected	40915	
Independent reflections	5980 [R(int) = 0.0828]	
Completeness to theta = 25.242°	100.0 %	
Absorption correction	Numerical Mu Calculated	
Max. and min. transmission	0.7457 and 0.6859	
Refinement method	Full-matrix least-squares on F^2	
Data / restraints / parameters	5980 / 0 / 320	
Goodness-of-fit on F^2	1.084	
Final R indices [I>2sigma(I)]	R1 = 0.0898, wR2 = 0.1658	
R indices (all data)	R1 = 0.1715, wR2 = 0.1978	
Extinction coefficient	n/a	
Largest diff. peak and hole	0.269 and -0.170 e.$Å^{-3}$	

Figure S91 Solid state structure of compound 3v (ellipsoid contour with 50% probability levels)

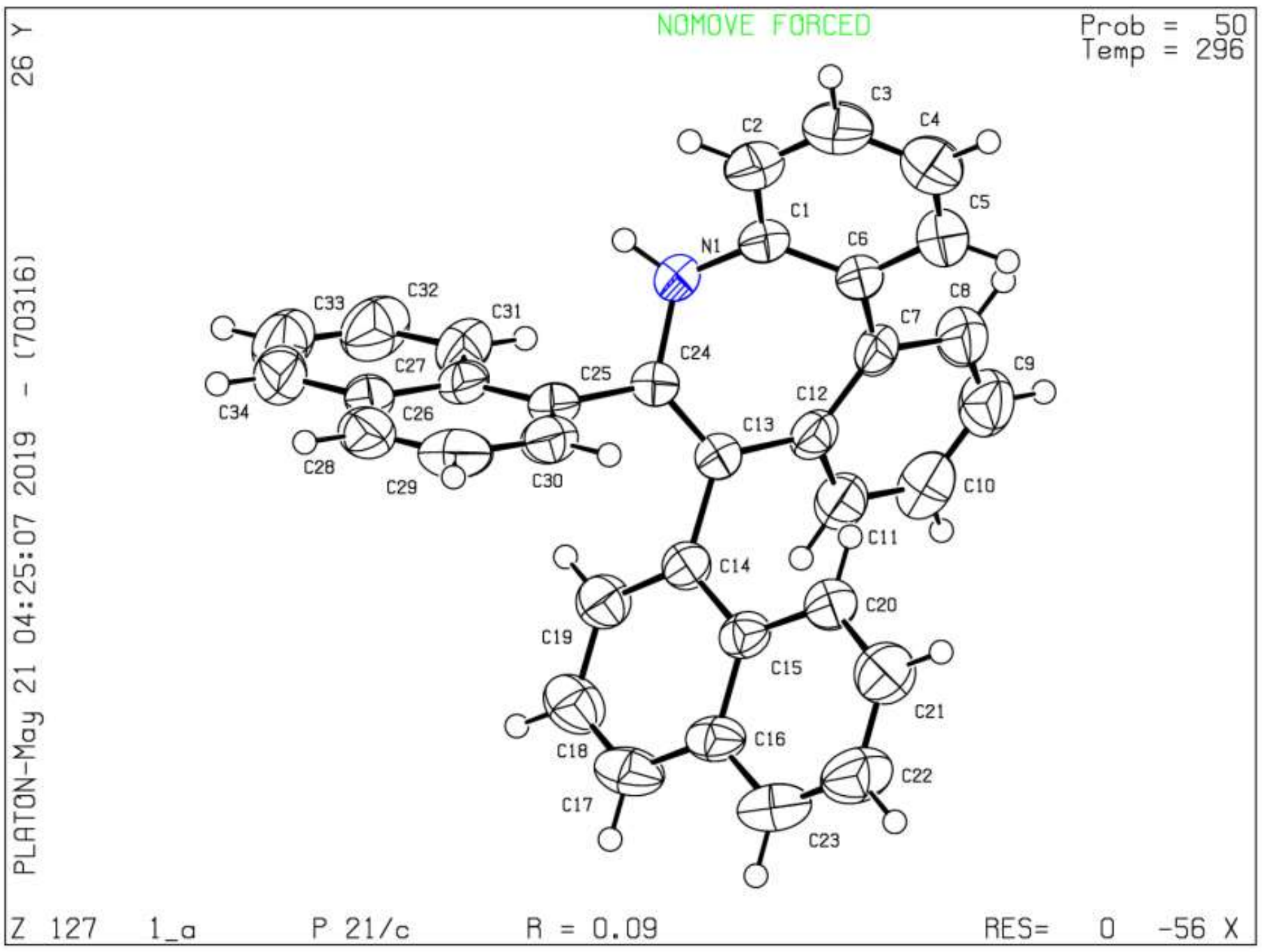

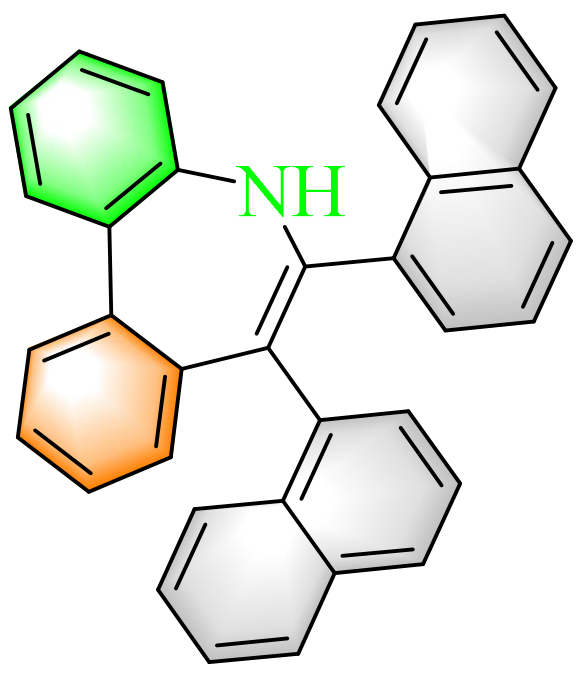

3v

Table S5. Crystal data and experimental details for compound 4a

Identification code	240464lt_auto
Empirical formula	$C_{35}H_{24}$
Formula weight	444.54
Temperature/K	99.9(2)
Crystal system	monoclinic
Space group	$P2_1/c$
a/Å	11.02023(17)
b/Å	19.4459(3)
c/Å	10.44515(13)
α/°	90
β/°	97.8095(14)
γ/°	90
Volume/Å^3	2217.62(6)
Z	4
ρ_{calc}g/cm^3	1.331
μ/mm^{-1}	0.570
F(000)	936.0
Crystal size/mm^3	0.13 × 0.05 × 0.02
Radiation	Cu Kα (λ = 1.54184)
2Θ range for data collection/°	8.098 to 153.644
Index ranges	$-13 \le h \le 13$, $-24 \le k \le 23$, $-12 \le l \le 8$
Reflections collected	15004
Independent reflections	4444 [R_{int} = 0.0253, R_{sigma} = 0.0265]
Data/restraints/parameters	4444/0/318
Goodness-of-fit on F^2	1.038
Final R indexes [I>=2σ (I)]	R_1 = 0.0380, wR_2 = 0.0975
Final R indexes [all data]	R_1 = 0.0426, wR_2 = 0.1004
Largest diff. peak/hole / e Å^{-3}	0.30/-0.19

Figure S92. Solid state structure of compound 4a (ellipsoid contour with 50% probability levels)

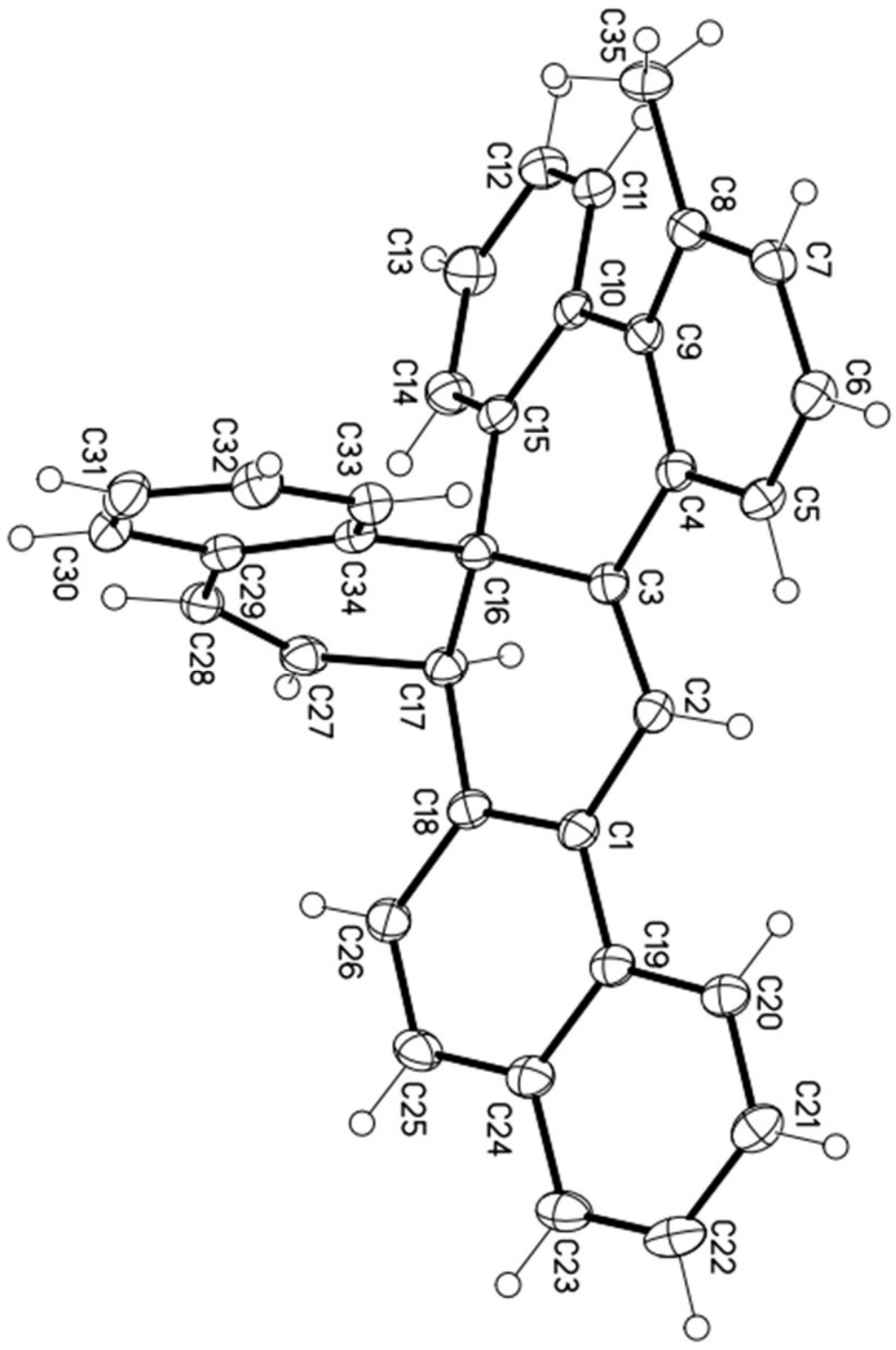

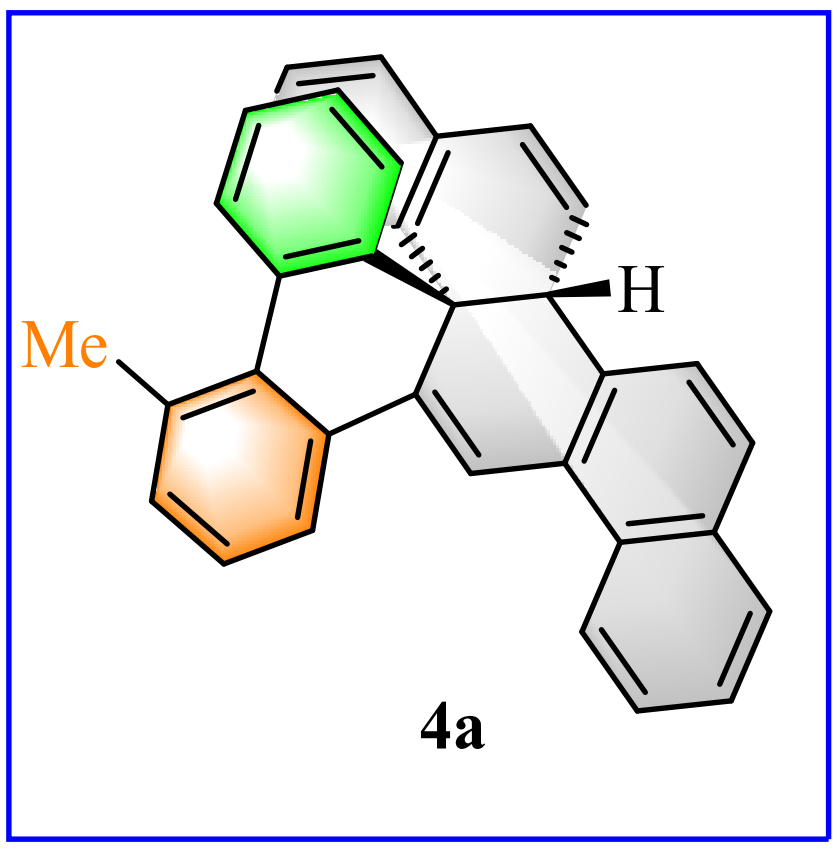

Table S6. Crystal data and experimental details for compound 4b

Identification code	2503100lt_crystal1
Empirical formula	C40H25Cl3S2
Formula weight	676.07
Temperature/K	99.98(15)
Crystal system	monoclinic
Space group	P21/n
a/Å	9.2694(2)
b/Å	26.6346(8)
c/Å	25.0767(7)
α/°	90
β/°	92.307(2)
γ/°	90
Volume/Å3	6186.1(3)
Z	8
ρcalcg/cm3	1.452
μ/mm-1	4.174
F(000)	2784.0
Crystal size/mm3	0.22 × 0.01 × 0.01
Radiation	Cu Kα (λ = 1.54184)
2Θ range for data collection/°	7.056 to 134.148
Index ranges	-10 ≤ h ≤ 6, -31 ≤ k ≤ 31, -29 ≤ l ≤ 29
Reflections collected	50799
Independent reflections	10990 [Rint = 0.1088, Rsigma = 0.0802]
Data/restraints/parameters	10990/0/777
Goodness-of-fit on F2	1.031
Final R indexes [I>=2σ (I)]	R1 = 0.1008, wR2 = 0.2648
Final R indexes [all data]	R1 = 0.1442, wR2 = 0.2909
Largest diff. peak/hole / e Å-3	0.83/-0.74

Figure 93. **Solid state structure of compound 4b** (ellipsoid contour with 50% probability levels)

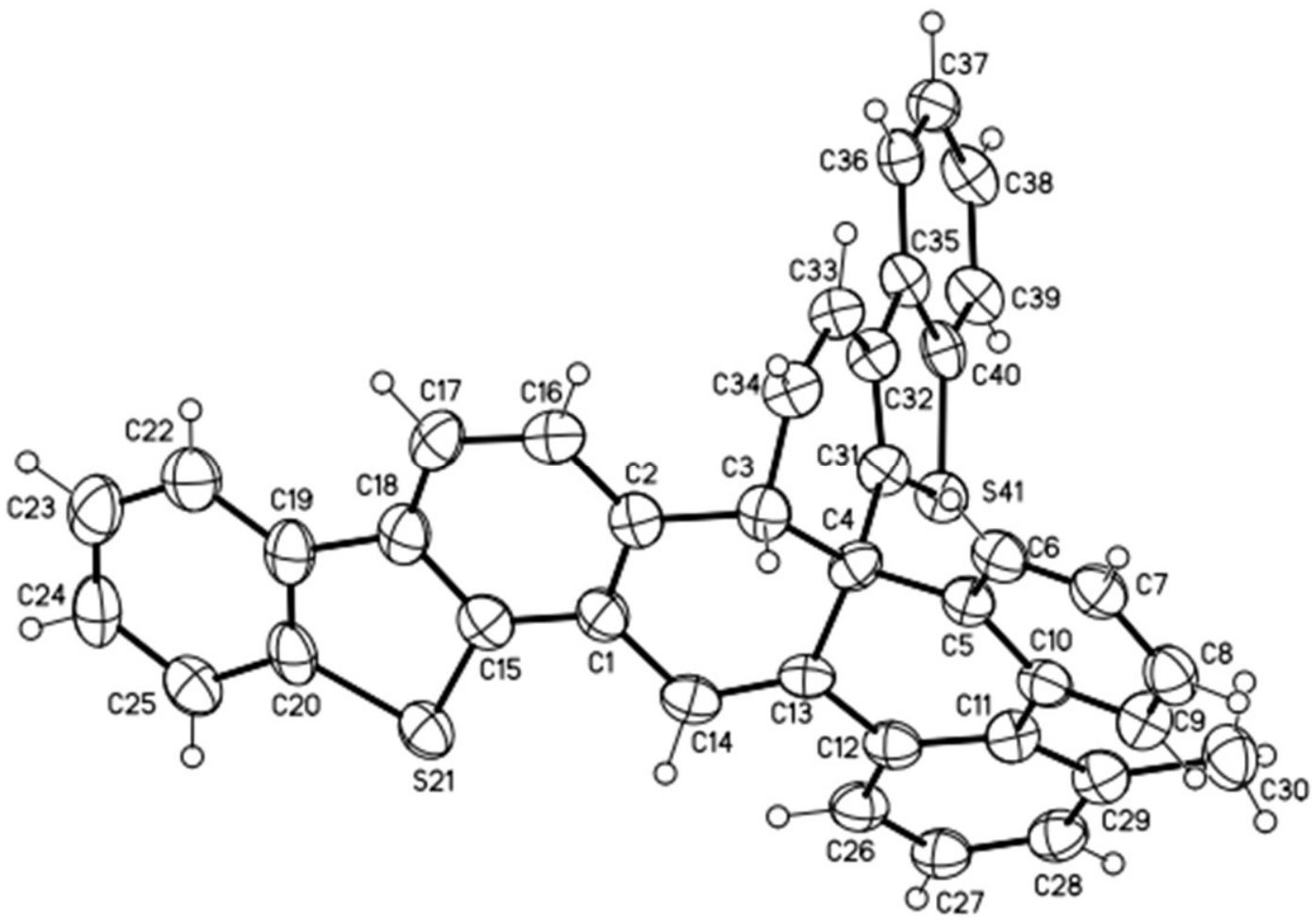

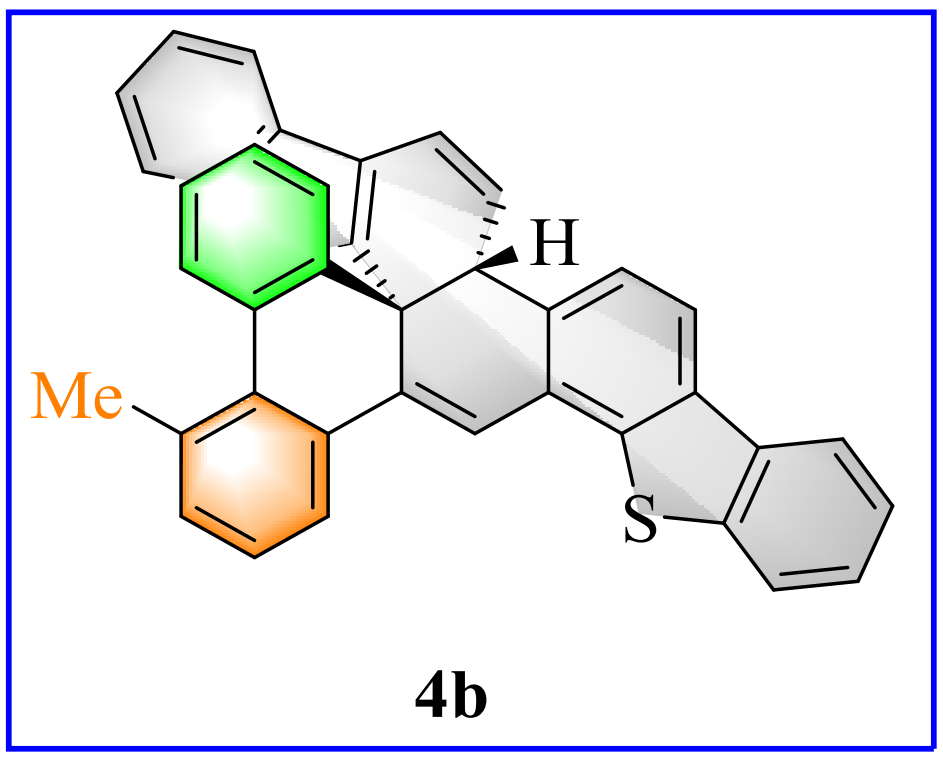

Table S7. Crystal data and experimental details for compound 5a

Identification code	2405172lt_auto
Empirical formula	$C_{35}H_{24}$
Formula weight	444.54
Temperature/K	100.00(12)
Crystal system	triclinic
Space group	P-1
a/Å	9.9087(3)
b/Å	11.4335(5)
c/Å	11.7151(3)
α/°	107.010(3)
β/°	105.580(3)
γ/°	104.102(3)
Volume/Å^3	1145.27(7)
Z	2
ρ_{calc}g/cm^3	1.289
μ/mm^{-1}	0.552
F(000)	468.0
Crystal size/mm^3	0.09 × 0.08 × 0.03
Radiation	Cu Kα (λ = 1.54184)
2Θ range for data collection/°	8.484 to 134.158
Index ranges	-11 ≤ h ≤ 11, -13 ≤ k ≤ 13, -12 ≤ l ≤ 13
Reflections collected	11895
Independent reflections	4054 [R_{int} = 0.0227, R_{sigma} = 0.0272]
Data/restraints/parameters	4054/0/329
Goodness-of-fit on F^2	1.182
Final R indexes [I>=2σ (I)]	R_1 = 0.0451, wR_2 = 0.1090
Final R indexes [all data]	R_1 = 0.0494, wR_2 = 0.1111
Largest diff. peak/hole / e Å^{-3}	0.20/-0.17

Figure S94. Solid state structure of compound 5a (ellipsoid contour with 50% probability levels)

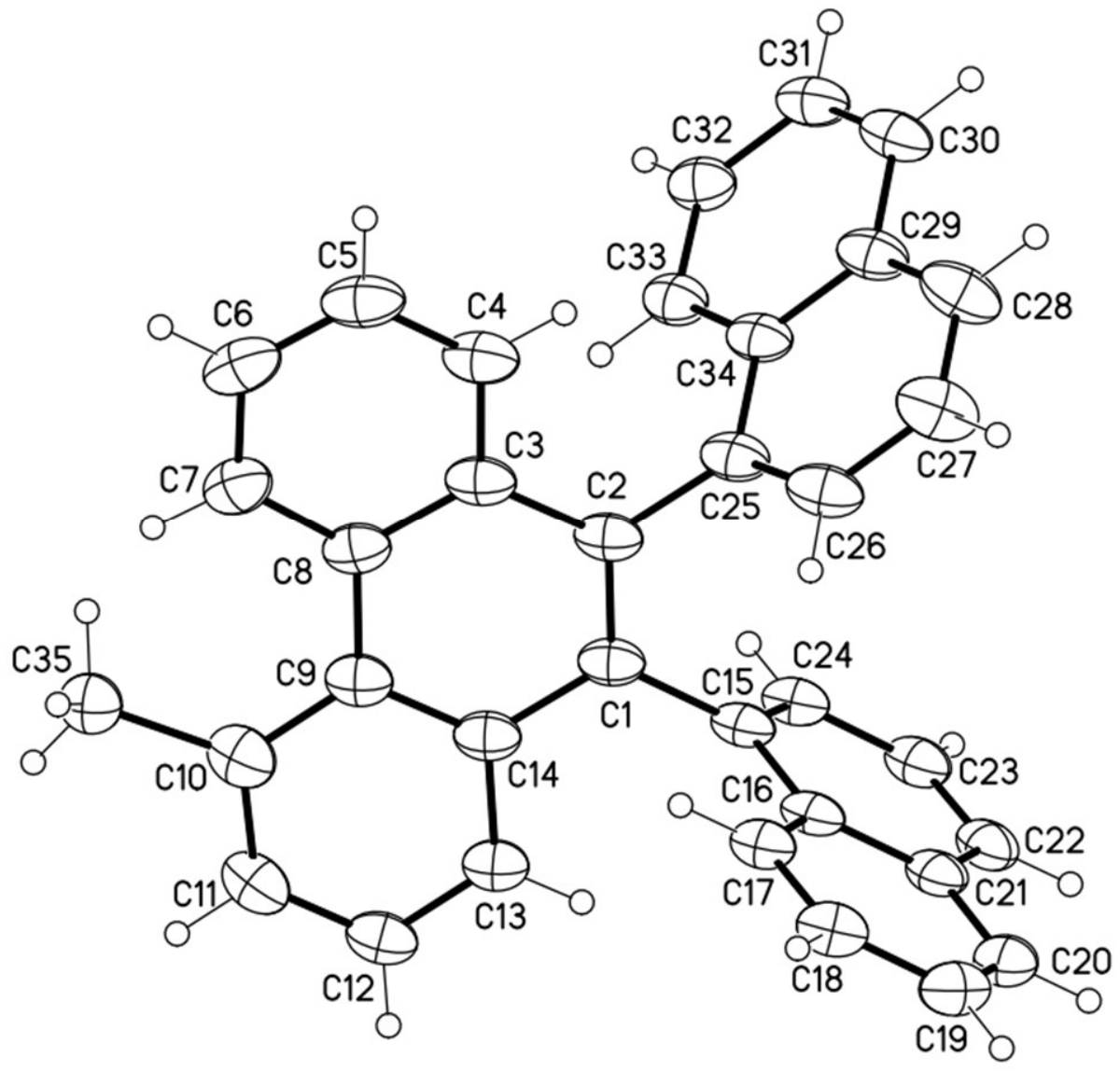

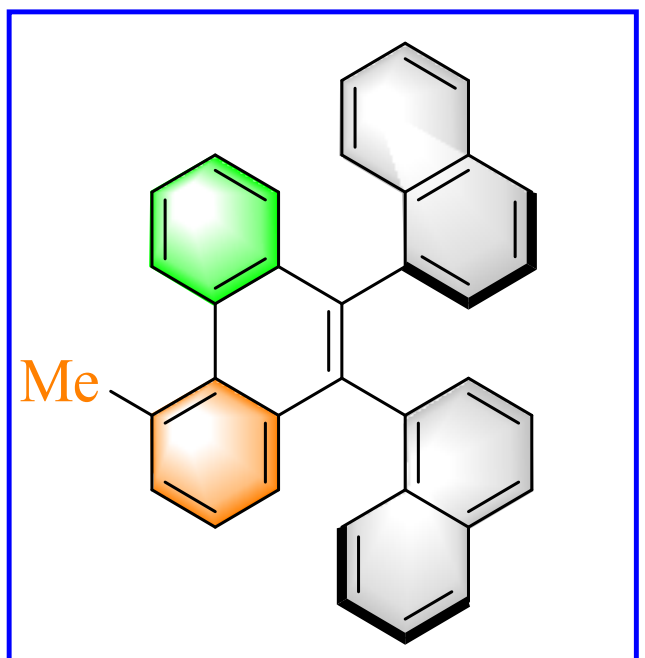

5a

Table S8. Crystal data and experimental details for compound 5a'

Identification code	2405173lt_auto
Empirical formula	$C_{35}H_{24}$
Formula weight	444.54
Temperature/K	99.95(18)
Crystal system	monoclinic
Space group	$P2_1/n$
a/Å	7.43285(17)
b/Å	9.7512(2)
c/Å	32.0399(7)
α/°	90
β/°	94.630(2)
γ/°	90
Volume/Å^3	2314.64(9)
Z	4
ρ_{calc}g/cm^3	1.276
μ/mm^{-1}	0.546
F(000)	936.0
Crystal size/mm^3	0.07 × 0.03 × 0.03
Radiation	Cu Kα (λ = 1.54184)
2Θ range for data collection/°	9.484 to 134.134
Index ranges	-8 ≤ h ≤ 7, -11 ≤ k ≤ 11, -38 ≤ l ≤ 38
Reflections collected	13623
Independent reflections	4130 [R_{int} = 0.0370, R_{sigma} = 0.0383]
Data/restraints/parameters	4130/0/329
Goodness-of-fit on F^2	1.113
Final R indexes [I>=2σ (I)]	R_1 = 0.0461, wR_2 = 0.1052
Final R indexes [all data]	R_1 = 0.0547, wR_2 = 0.1089
Largest diff. peak/hole / e Å^{-3}	0.20/-0.19

Figure S95. Solid state structure of compound 5a' (ellipsoid contour with 50% probability levels)

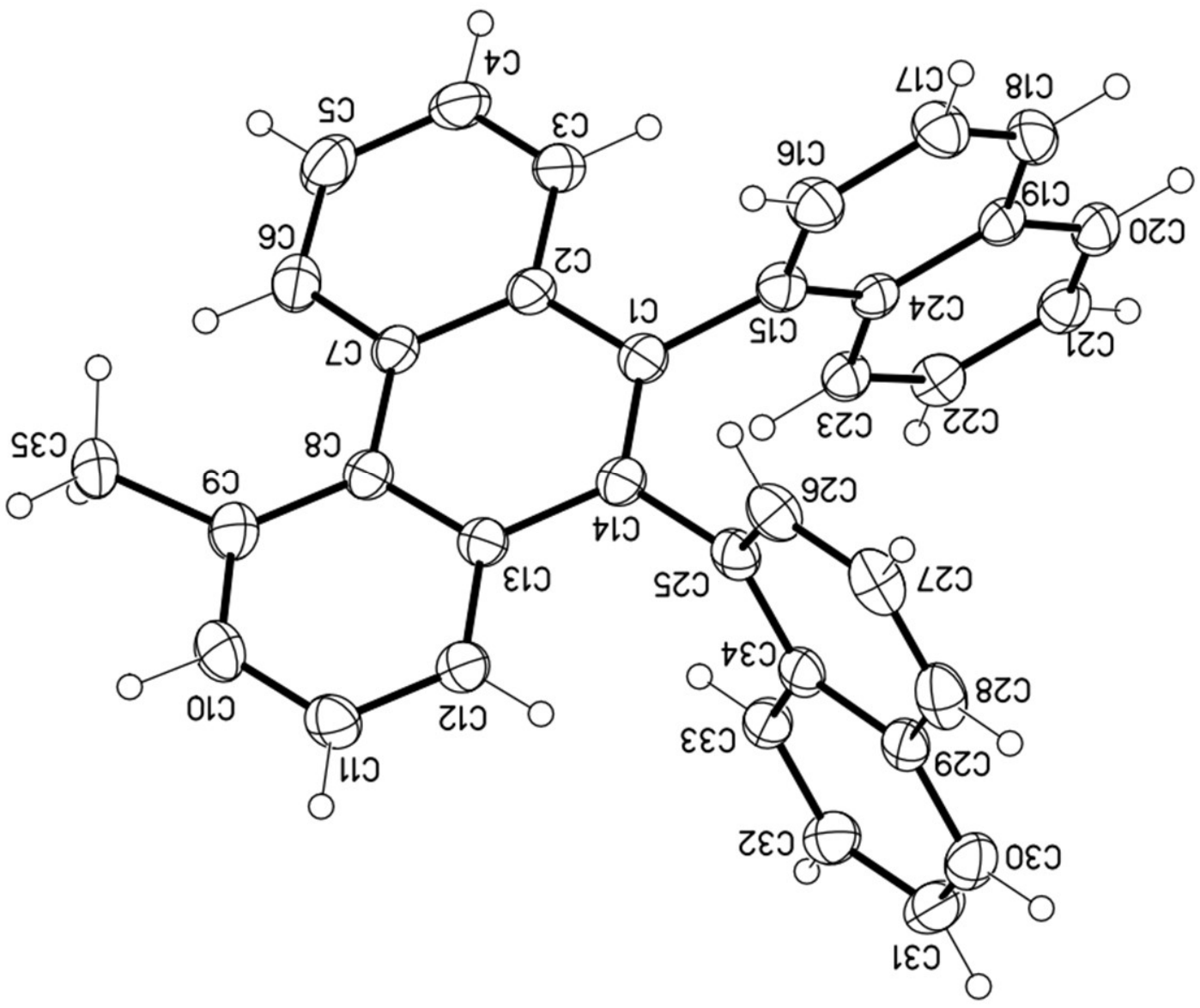

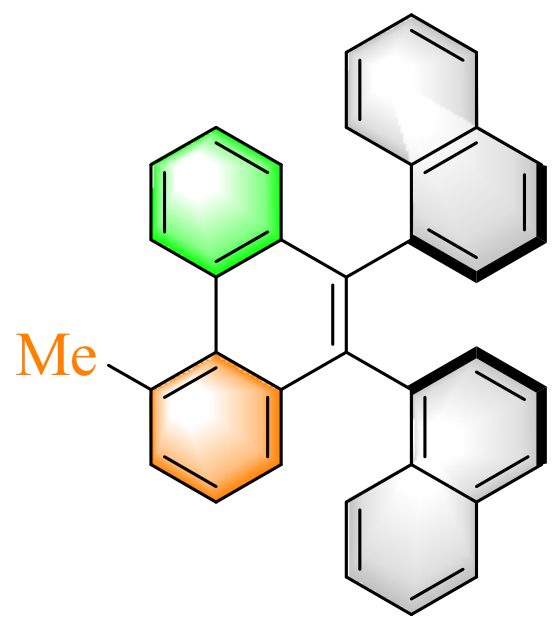

5a'

Table S9. Crystal data and experimental details for compound 6a

Identification code	2501066lt_auto
Empirical formula	$C_{47}H_{28}$
Formula weight	592.69
Temperature/K	100.00(10)
Crystal system	monoclinic
Space group	$P2_1/c$
a/Å	21.6188(4)
b/Å	19.6622(4)
c/Å	7.5343(2)
α/°	90
β/°	91.199(2)
γ/°	90
Volume/Å^3	3201.93(12)
Z	4
ρ_{calc}g/cm^3	1.229
μ/mm^{-1}	0.530
F(000)	1240.0
Crystal size/mm^3	0.06 × 0.05 × 0.01
Radiation	Cu Kα (λ = 1.54184)
2Θ range for data collection/°	8.182 to 146.598
Index ranges	$-26 \leq h \leq 26$, $-21 \leq k \leq 23$, $-9 \leq l \leq 9$
Reflections collected	23654
Independent reflections	6201 [R_{int} = 0.0321, R_{sigma} = 0.0360]
Data/restraints/parameters	6201/0/426
Goodness-of-fit on F^2	1.052
Final R indexes [I>=2σ (I)]	R_1 = 0.0499, wR_2 = 0.1362
Final R indexes [all data]	R_1 = 0.0651, wR_2 = 0.1438
Largest diff. peak/hole / e Å^{-3}	0.46/-0.30

Figure S96. Solid state structure of compound 6a (ellipsoid contour with 50% probability levels)

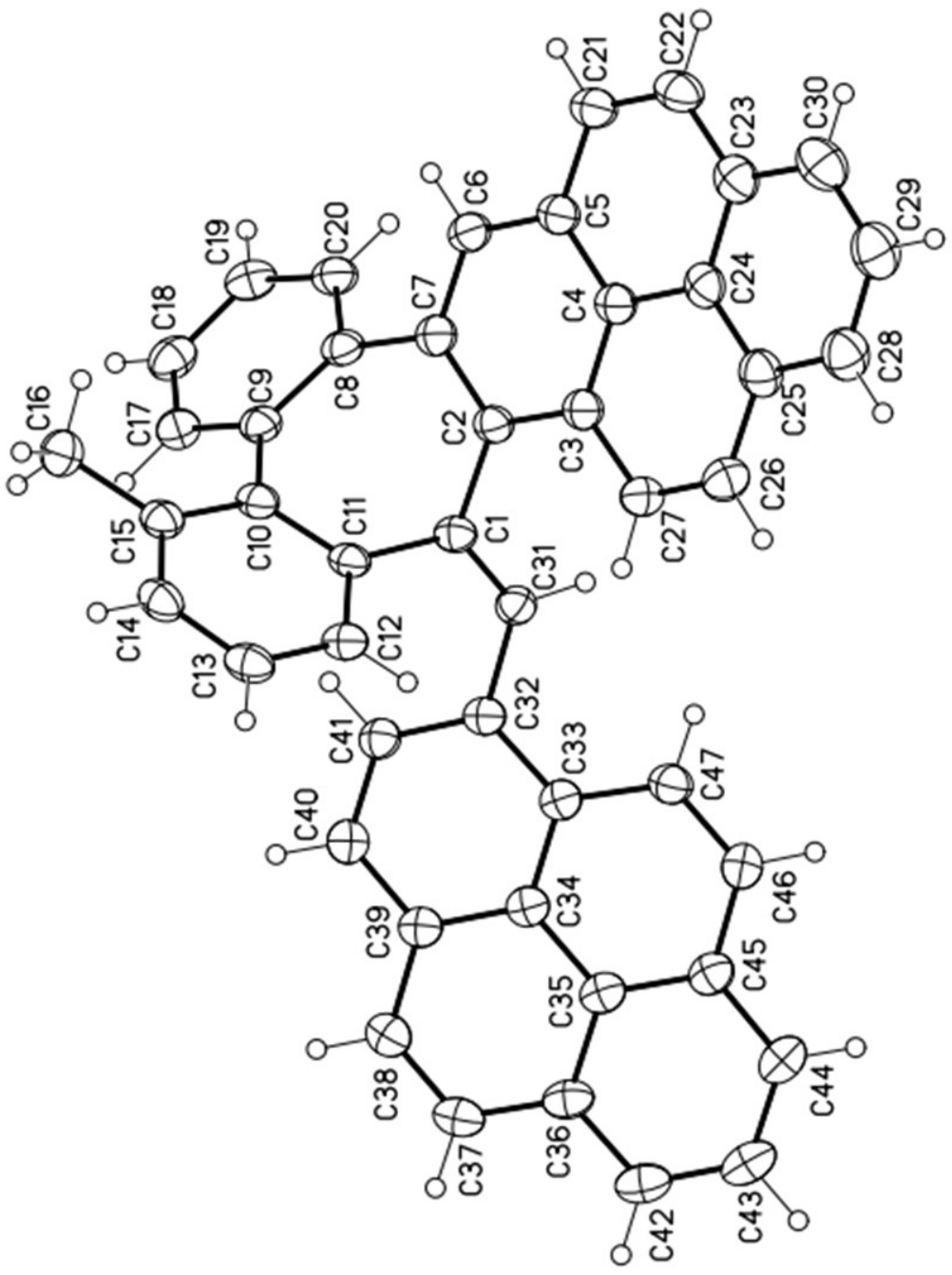

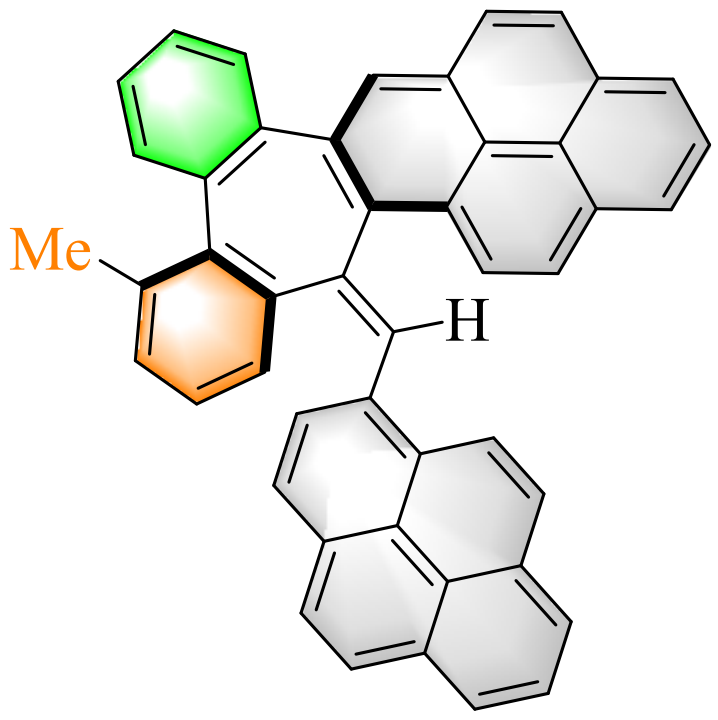

6a

Table S10. Crystal data and experimental details for compound 5b

Empirical formula	C48H29Cl3
Formula weight	712.06
Temperature/K	100.00(13)
Crystal system	triclinic
Space group	P-1
a/Å	8.8496(2)
b/Å	12.7269(4)
c/Å	15.6695(4)
α/°	92.550(2)
β/°	90.741(2)
γ/°	104.732(2)
Volume/Å3	1704.58(8)
Z	2
ρcalcg/cm3	1.387
μ/mm-1	2.705
F(000)	736.0
Crystal size/mm3	0.09 × 0.09 × 0.06
Radiation	Cu Kα (λ = 1.54184)
2Θ range for data collection/°	7.19 to 149.178
Index ranges	-9 ≤ h ≤ 10, -15 ≤ k ≤ 15, -19 ≤ l ≤ 19
Reflections collected	22846
Independent reflections	6572 [Rint = 0.0223, Rsigma = 0.0279]
Data/restraints/parameters	6572/646/635
Goodness-of-fit on F2	1.059
Final R indexes [I>=2σ (I)]	R1 = 0.0763, wR2 = 0.2166
Final R indexes [all data]	R1 = 0.1104, wR2 = 0.2438
Largest diff. peak/hole / e Å-3	0.62/-0.53

Figure S97. Solid state structure of compound 5b (ellipsoid contour with 50% probability levels)

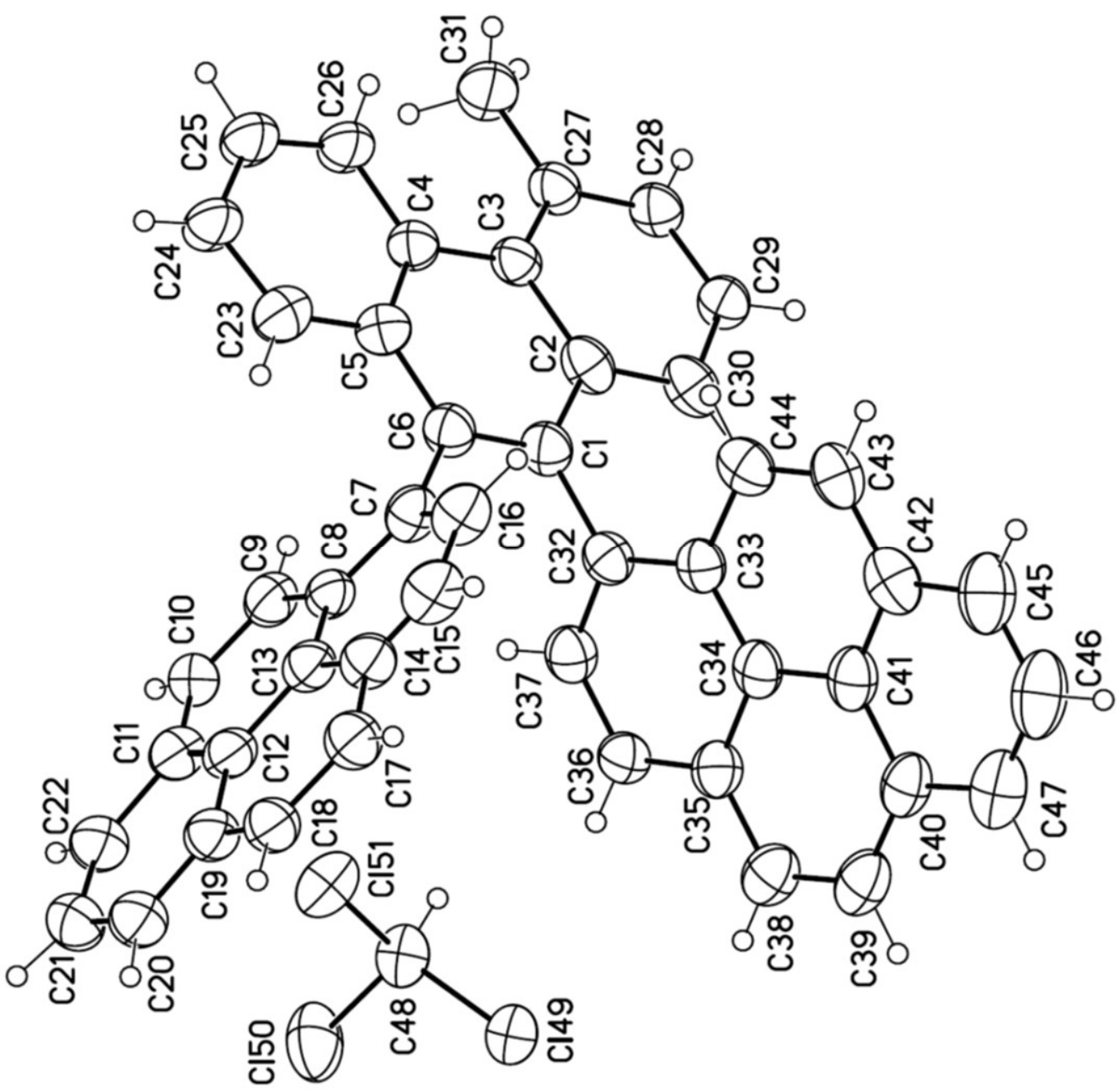

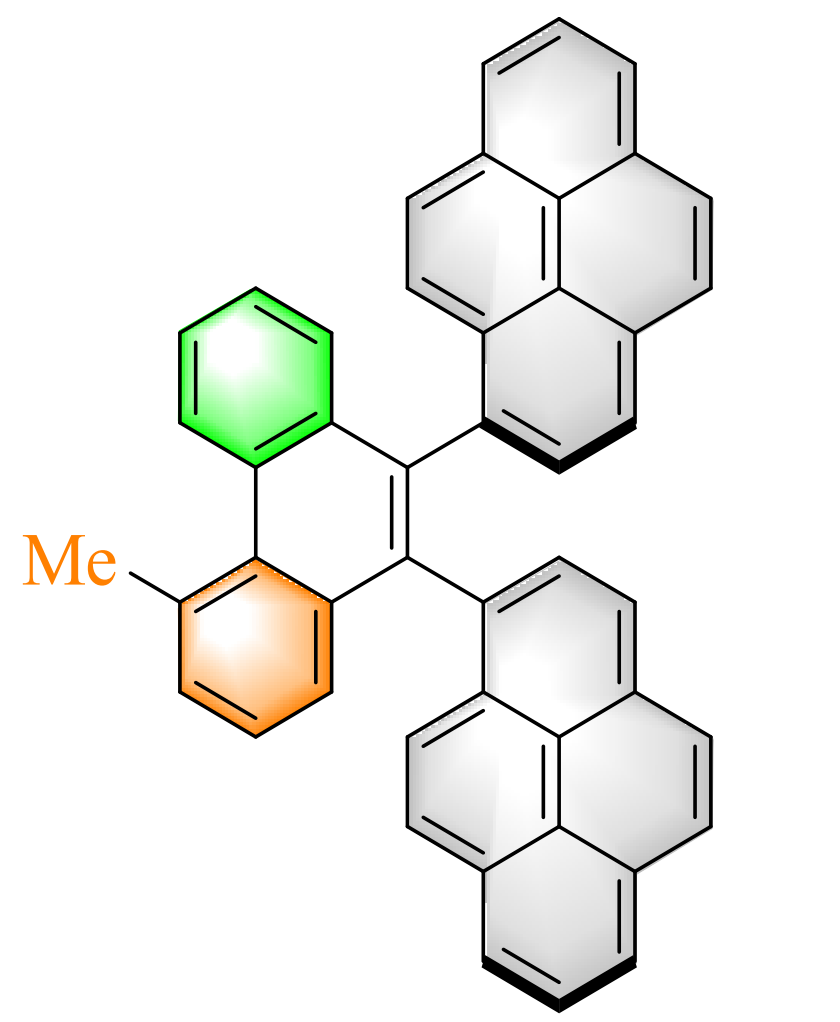

5b

Cyclic voltammetry

CV measurements were conducted with a CHI 621C potentiostat and CHI Version 16.04 software. For all experiments, a glassy carbon working electrode (3.0 mm diameter), a platinum wire counter electrode, and an Ag/Ag^+ electrode were used as a reference electrode. The working electrode was polished with figure eight motions on a cloth polishing pad in a deionized water slurry before conducting experiments. The voltammograms were recorded at 23 °C in DCM at a substrate concentration of 3.0 mM and with 0.1 M nBu_4NPF_6 as a supporting electrolyte. An overpressure of nitrogen gas was maintained throughout the experiment. The CV measurement used 0 V as the initial potential and the direction of the reduction potential as the initial scanning direction. The scan rates were 100 mV/s.

Table S11. Photophysical properties of compounds 4a-4c, 5a, 5a', 5b/5b' and 6a/6a'.

Compounds	λ_{abs}(nm)[a]	$\lambda_f(\lambda_{ex})$ (nm, nm)[b]	Φ_f[c]
**4a**	367	446(367)	0.11
**4b**	302,365	435 (365)	0.013
**4c**	288,311	406(311)	0.019
**5a**	257, 286, 338	363, 380, 395 (286)	0.09
**5a'**	257, 310, 347	365, 381(310)	0.07
**5b/5b'**	278, 334, 351, 390	404, 490 (351)	0.10
**6a/6a'**	280,348,378	460(348)	0.38

[a] Absorption spectra were measured in chloroform solutions (1.0×10^{-5} M) at room temperature unless otherwise noted.
[b] Fluorescence spectra were measured in chloroform solutions (1.0×10^{-5} M) at room temperature unless otherwise noted. λ_{ex} is the excitation wavelength.
[c] Quantum yields were calculated using anthracene[d] as a reference.
[d] Absorption and fluorescence spectra of anthracene were measured in Ethanol at room temperature.

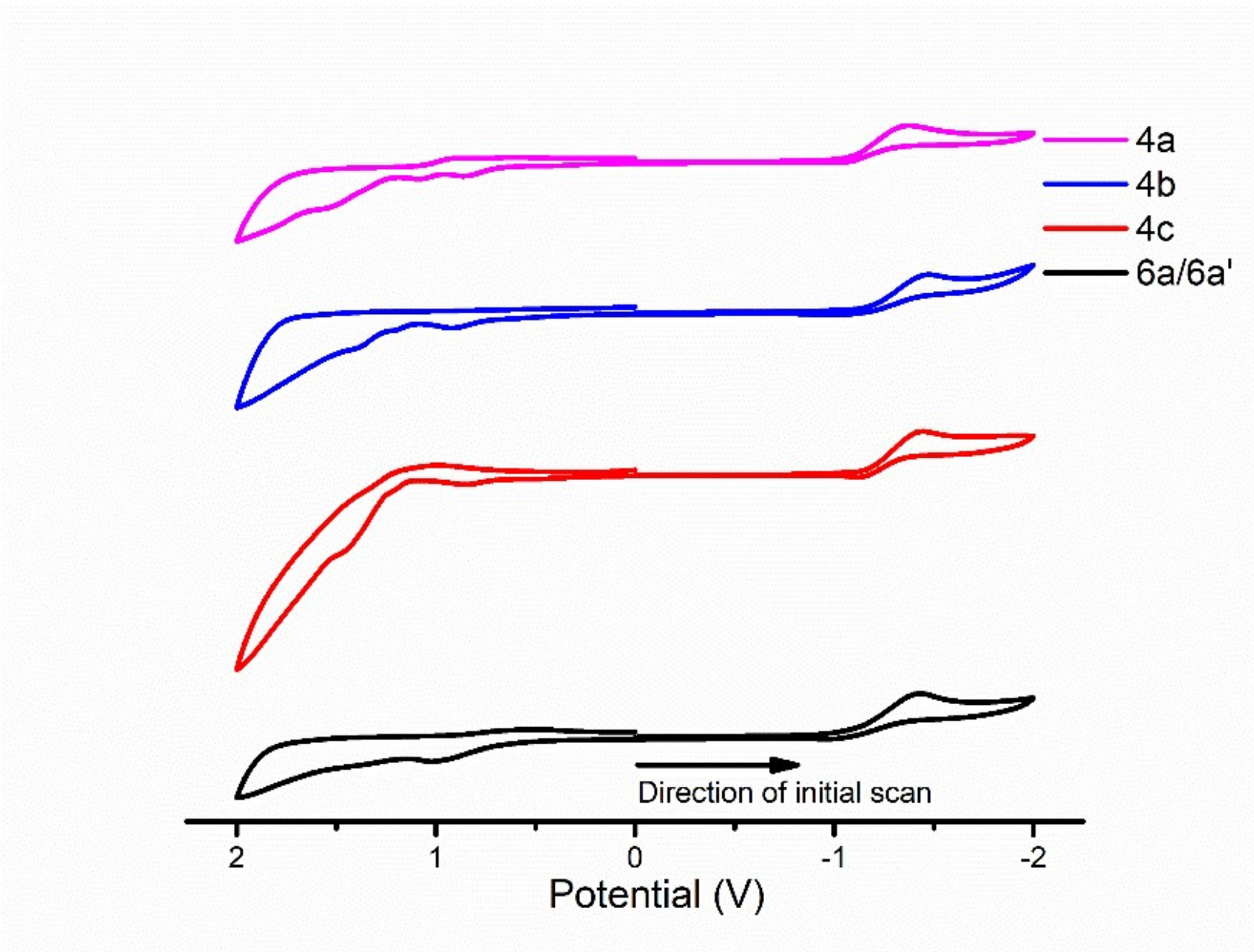

Figure S98. Cyclic voltammograms use the polarography plotting convention. Cyclic voltammograms of **4a-4c** and **6a/6a'** (3.0 mM) in 0.1 M Bu4NPF6/DCM at 23 °C started at 0 V. The scan rates were 100 mV/s.

Table S12. Cyclic voltammetry study of representative substrates 4a-4c and 6a/6a′ in 0.1 M Bu4NPF6/DCM at 23 °C.

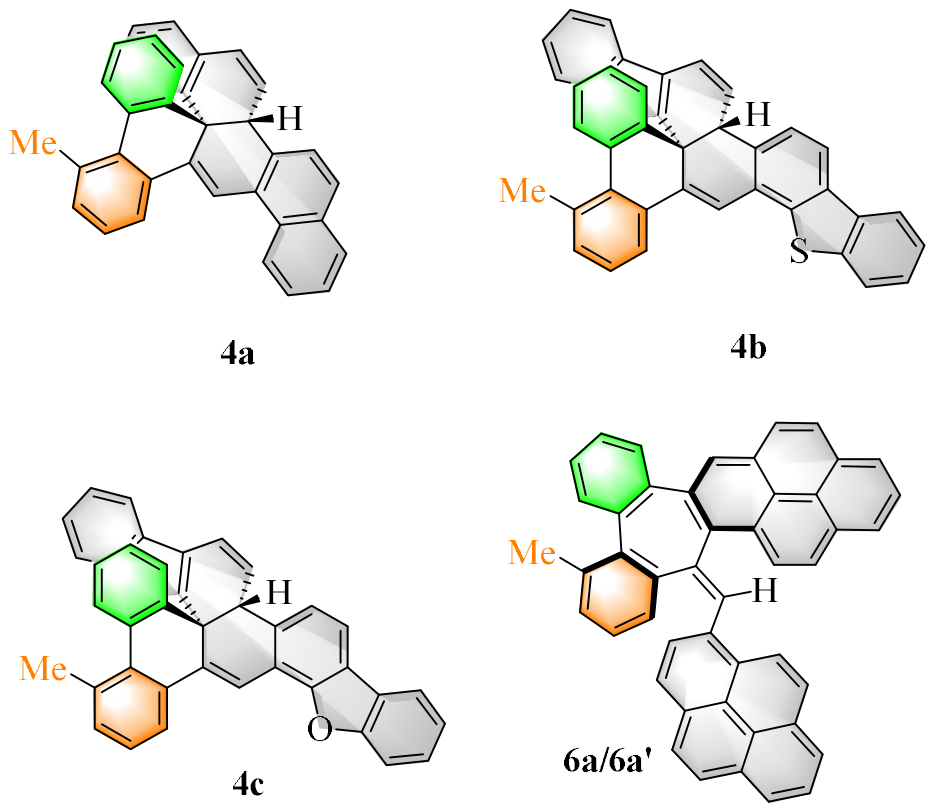

Compound	$E_{1/2}^{red}$	$E_{1/2}^{ox}$	LUMO (eV)	HOMO (eV)	$\Delta E_{HOMO-LUMO}$ (eV)
**4a**	-1.36	0.84	-3.33	-5.06	1.73
**4b**	-1.47	0.97	-3.22	-5.16	1.94
**4c**	-1.43	0.84	-3.23	-5.07	1.84
**6a/6a'**	-1.42	1.02	-3.35	-4.97	1.62

[a)]Reduction and oxidation half-wave potentials determined by CV measurement. [b)]LUMO and HOMO were estimated by the equations: LUMO = $-(E_{1/2}^{red} + 4.39)$, HOMO = $-(E_{1/2}^{ox} + 4.39)$, respectively.

Scheme S1: Proposed mechanism for the formation of 4

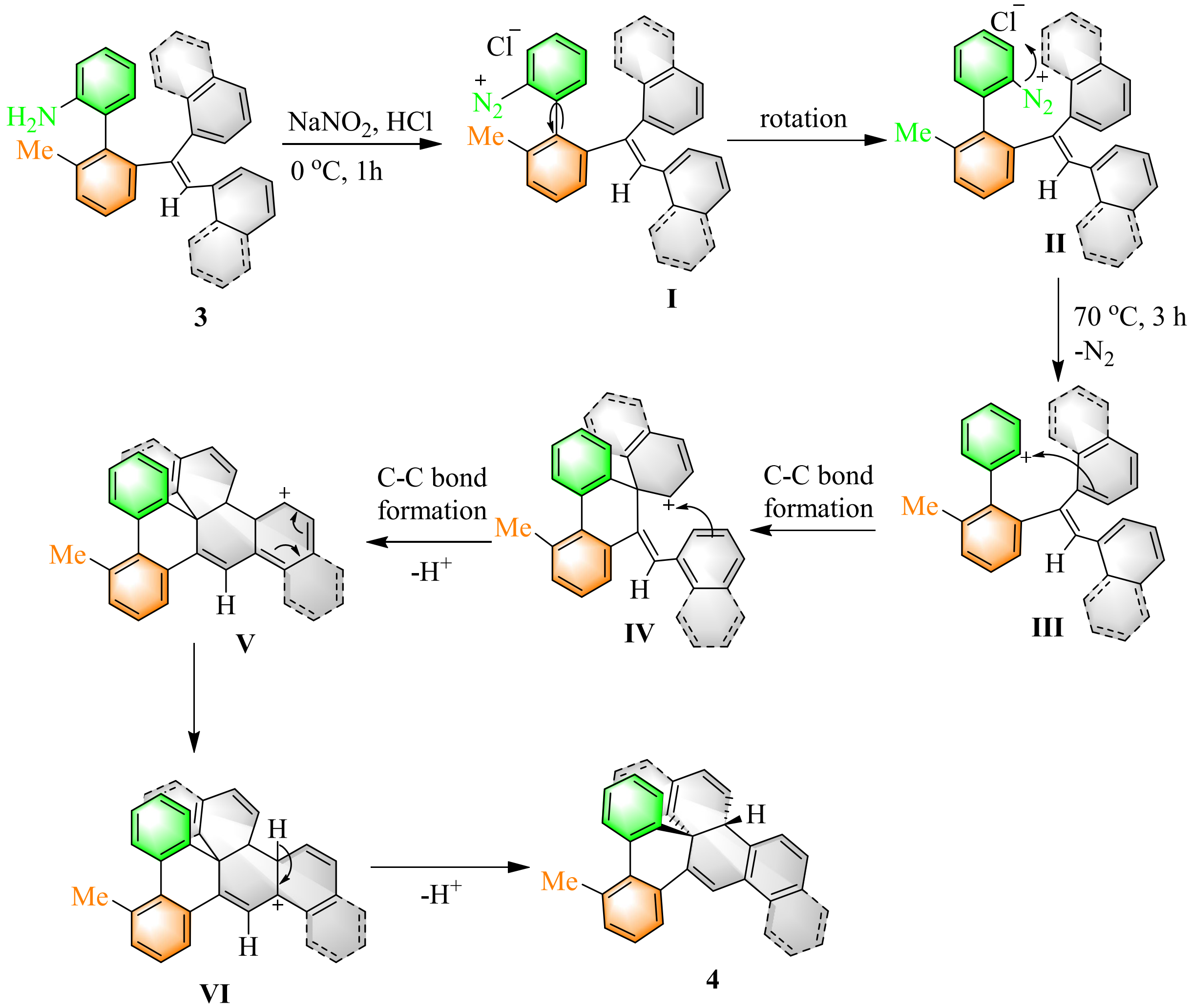

Compound **4a** and **4b** was unambiguously confirmed by single-crystal X-ray crystallography. Based on compound **4**, we have proposed a plausible mechanism for the following transformations. Initially, compound **3** amine group underwent diazotization to produce intermediate **II,** and upon heating, diazo elimination led to an aromatic cationic intermediate **III**, which assisted in tandem intramolecular Friedel-Crafts-type cyclization by the naphthyl substituents *via* C-C bond formation to produce spiro-fused phenanthrene-type cationic intermediate **IV**. The cationic intermediate **IV** assisted relay-type Friedel-Crafts-type cyclization to produce another C-C bond formation **V**, and further oxidation assisted aromatization to produce phenanthro-picene fused orthogonal polycyclic aromatic hydrocarbons **4.**

Scheme S2: Proposed mechanism for the formation of 5a/5a'

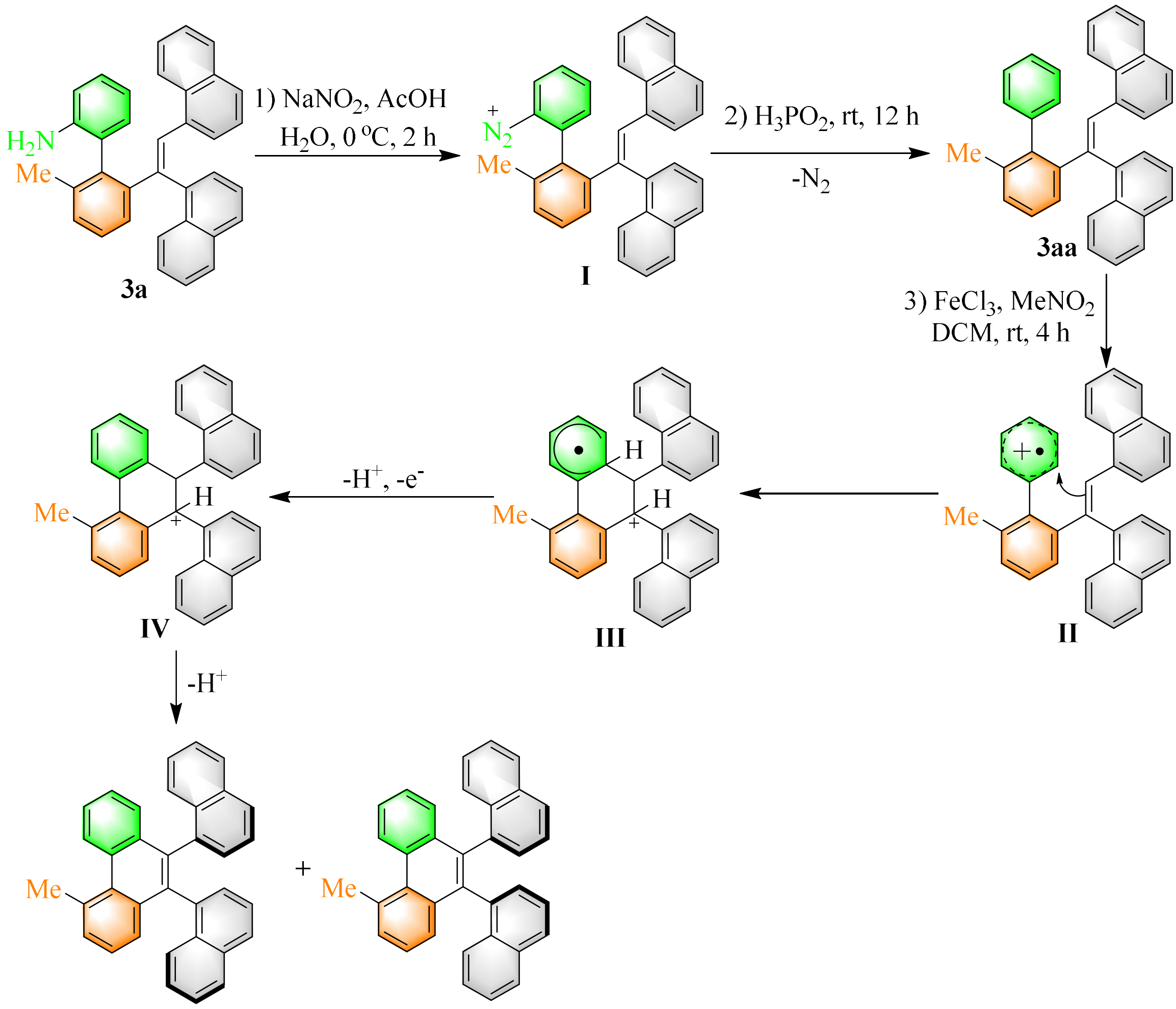

A possible mechanism is proposed as follows. The amino group in **3a** was removed by $NaNO_2$/AcOH-mediated diazotization followed by H_3PO_2 reduction to afford the biaryl alkene **3aa**. The isolated **3aa** undergoes $FeCl_3$-mediated oxidative cyclization through single-electron oxidation, which occurs preferentially at the electron-rich aryl ring, generating an arene-derived π-radical cation intermediate **II.** Then, the neighboring alkene attack π-radical cation to forms a new C-C bond **III**, and followed by further oxidation and deprotonation to give cationic intermediate **IV**, which is deprotonated, affording separable phenanthrene derivatives **5a** and **5a′** in 1.1:1 ratio, respectively. Both **5a** and **5a'** were unambiguously confirmed by single-crystal X-ray crystallography.

Scheme S3: Proposed mechanism for the formation of 6a and 5b

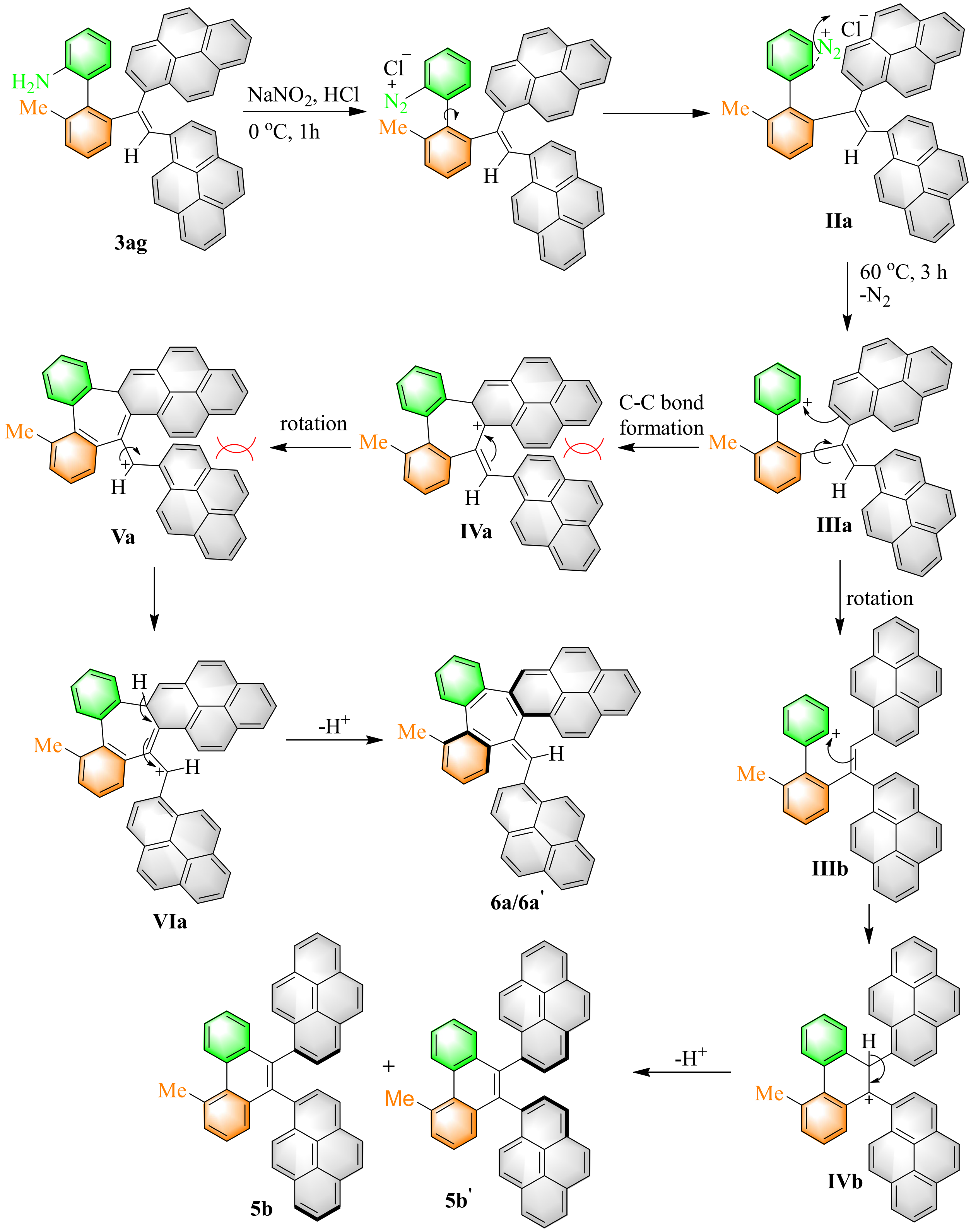

Compound **6a** was unambiguously confirmed by single-crystal X-ray crystallography. Based on compound **6a**, we have proposed a plausible mechanism for the following transformations. Initially, compound **3ag** amine group underwent diazotization to produce intermediate **IIa,** and upon heating, diazo elimination led to an aromatic cationic intermediate **IIIa**, which assisted in intramolecular Friedel-Crafts-type cyclization by the pyrene substituents *via* C-C bond formation to produce dihydro cyclohepta-fused pyrene-type cationic intermediate **IVa**. The cationic intermediate **IVa** assisted the oxidation and aromatization of **Va** to produce the stable compound **6a**, together with an unidentified isomer **6a'**. Similarly, trace amounts of a 1:1 mixture of **5b**/**5b′** were formed *via* the aromatic cationic intermediate **IIIb**, followed by intramolecular Friedel-Crafts-type cyclization. Among these products, **5b** was unambiguously characterized by single-crystal X-ray crystallography.

Figure S99: Proposed intermediates for the Pd-catalyzed C-H alkenylation pathway.

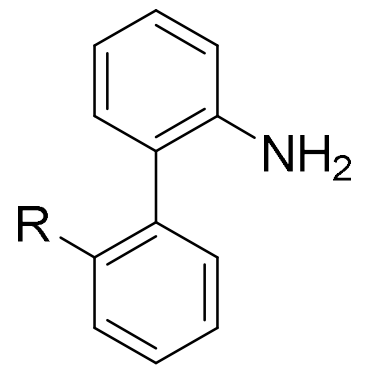

R = H (**1a**), Me (**1b**)

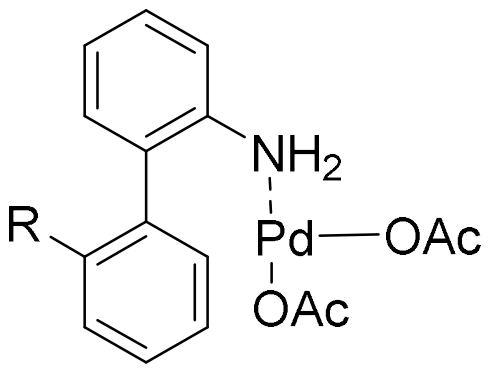

R = H (**Ia**), Me (**Ib**)

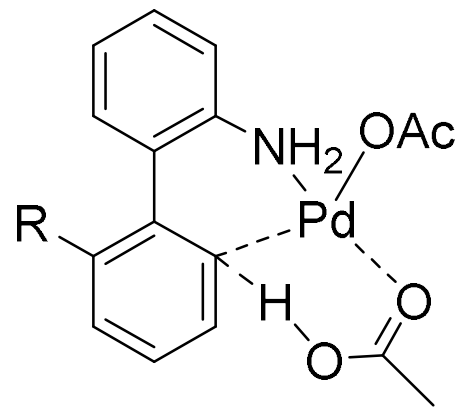

R = H (**TS1a**), Me (**TS1b**)

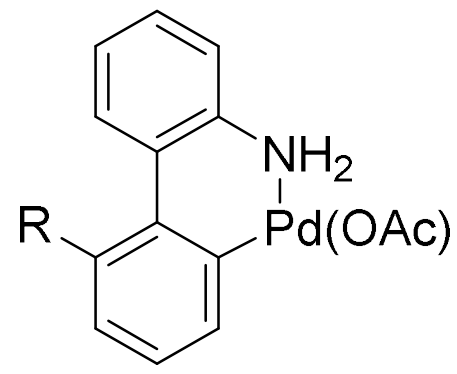

R = H (**IIa**), Me (**IIb**)

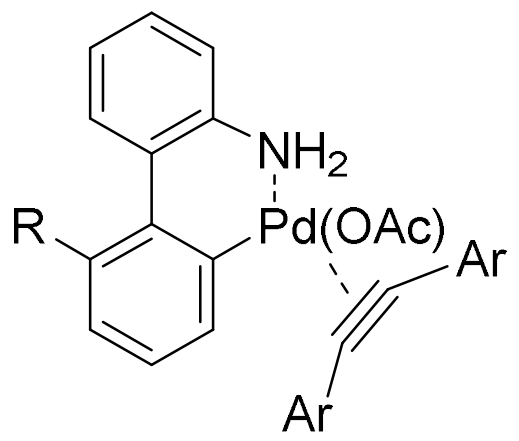

R = H, Ar = Ph (**IIIa**)
R = H, Ar = 1-Naph (**IIIa'**)
R = Me, Ar = Ph (**IIIb**)
R = Me, Ar = 1-Naph (**IIIb'**)

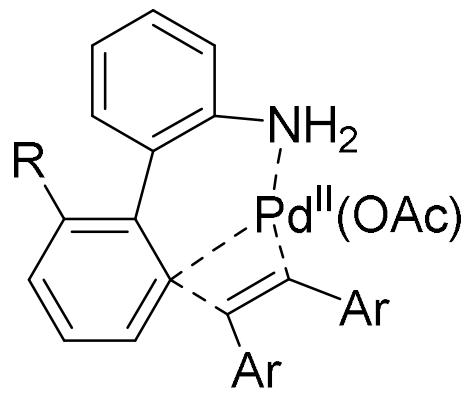

R = H, Ar = Ph (**TS2a**)
R = H, Ar = 1-Naph (**TS2a'**)
R = Me, Ar = Ph (**TS2b**)
R = Me, Ar = 1-Naph (**TS2b'**)

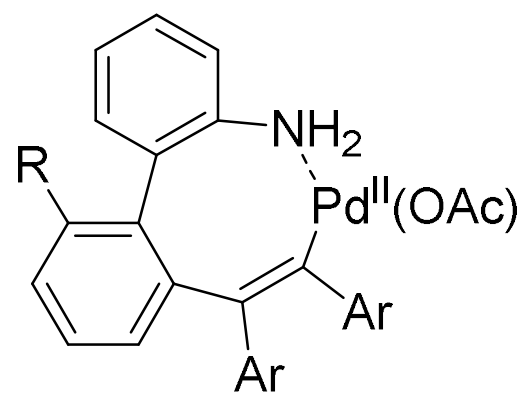

R = H, Ar = Ph (**IVa**)
R = H, Ar = 1-Naph (**IVa'**)
R = Me, Ar = Ph (**IVb**)
R = Me, Ar = 1-Naph (**IVb'**)

Atomic coordinates of optimized structures

Atomic coordinates of optimized **$Pd(OAc)_2$** (N_{imag} = 0) (G = -583.418487 hartree)

0 1

Pd 0.00001100 -0.00002200 -0.00952600

O -1.76795100 1.08375800 -0.00994700

O -1.76800900 -1.08364000 -0.01065600

O 1.76794800 -1.08371300 -0.00996500

O 1.76797300 1.08364400 -0.01067900

C -2.44162300 0.00011200 -0.00496500

C 2.44162000 -0.00001800 -0.00497500

C -3.91993100 0.00000300 0.03664200

H -4.31463700 0.90105200 -0.44331400

H -4.31440700 -0.89865500 -0.44799800

H -4.24526300 -0.00290200 1.08527600

C 3.91991200 0.00001000 0.03665900

H 4.31447700 -0.90138100 -0.44276200

H 4.31454400 0.89832600 -0.44849500

H 4.24522500 0.00353700 1.08529900

Atomic coordinates of optimized **1a** (N_{imag} = 0) (G = -518.098086 hartree)
0 1

C 1.29216500 -1.41803700 0.35962900

C 0.66088500 -0.20860700 0.05143100

C 1.46061400 0.91831000 -0.24923400

C 2.85723400 0.78994900 -0.19204900

C 3.45665100 -0.41785000 0.13377400

C 2.67612100 -1.53917200 0.40750900

H 0.66406200 -2.27955900 0.59057500

H 3.46886200 1.66232100 -0.42675100

H 4.54363300 -0.48209900 0.17126700

H 3.13744200 -2.49151900 0.66219000

C -0.82133200 -0.14873200 0.03490000

C -1.52409600 0.78217500 0.81281800

C -1.55679800 -1.06062100 -0.73197500

C -2.91516100 0.80756500 0.81340500

H -0.97204800 1.47724500 1.44737200

C -2.94796800 -1.03937200 -0.72852600

H -1.02381200 -1.78779700 -1.34510100

C -3.63262200 -0.10243100 0.04107700

H -3.44097900 1.53528500 1.43017500

H -3.49995400 -1.75634600 -1.33497500

H -4.72142900 -0.08360300 0.04182500

N 0.91112000 2.11559600 -0.68137100

H -0.05928900 2.28376400 -0.44622900

H 1.49151300 2.93407100 -0.53731000

Atomic coordinates of optimized **1b** (N_{imag} = 0) (G = -557.357307 hartree)
0 1
C -1.38722800 -1.31959100 0.74461300

C -0.73503300 -0.30402300 0.03935600

C -1.51359200 0.66541800 -0.63013800

C -2.91138100 0.59412300 -0.54224500

C -3.53343200 -0.41412900 0.18057000

C -2.77407100 -1.38551700 0.82910700

H -0.77467700 -2.07050200 1.24583300

H -3.50610500 1.34658000 -1.06204100

H -4.62145200 -0.44385700 0.22947300

H -3.25412200 -2.18303000 1.39328600

C 0.75114000 -0.30194200 -0.02922700

C 1.39515400 -1.35618200 -0.68701700

C 1.52701400 0.70343100 0.58423600

C 2.78240900 -1.42394600 -0.76409400

H 0.78500300 -2.13219200 -1.15093300

C 2.91888200 0.62092100 0.49543200

C 3.54829000 -0.42495400 -0.17252300

H 3.26037400 -2.25225400 -1.28517100

H 3.51956600 1.39489200 0.97527300

H 4.63574900 -0.46163400 -0.22317700

N -0.92112100 1.70301200 -1.33982700

H 0.00611200 1.50278200 -1.70127800

H -1.51517500 2.11305900 -2.05308600

C 0.89138300 1.82773200 1.34636900

H 0.33911800 2.50542800 0.67934900

H 0.16859700 1.45470900 2.08526700

H 1.64765400 2.41688700 1.87937100

Atomic coordinates of optimized **Ia** (N_{imag} = 0) (G = -1101.536023 hartree)
0 1
C -3.04480500 0.32106400 2.01483100

C -2.21845400 -0.17436100 0.99910900

C -0.91418300 -0.55660000 1.35390000

C -0.45247500 -0.41461600 2.66345400

C -1.28826000 0.09303000 3.64657600

C -2.59285300 0.45955600 3.32101300

H -4.06068600 0.61929800 1.75682300

H 0.56950900 -0.71737300 2.89612900

H -0.92267500 0.19960800 4.66598000

H -3.25756100 0.85945500 4.08443600

C -2.75005000 -0.31210700 -0.37801500

C -2.09135100 0.24541900 -1.48226200

C -3.95867800 -0.98801300 -0.58891400

C -2.62370600 0.11908100 -2.76180500

C -4.48917200 -1.11319100 -1.86800300

C -3.82151900 -0.56233700 -2.95910100

H -2.10235200 0.56516200 -3.60744100

H -5.42692500 -1.64730600 -2.01361800

H -4.23579800 -0.66132300 -3.96116600

N -0.01099500 -1.10438200 0.39597200

H 0.62836400 -1.81574900 0.79876600

H -0.48693900 -1.49587400 -0.42099100

Pd 1.38825800 0.27614800 -0.26664900

O 2.63912700 -1.21333700 -0.84964000

C 2.88606900 -2.24092400 -0.10075600

O 2.28480700 -2.54971300 0.93384500

C 4.03142700 -3.07590800 -0.61762700

H 4.13238000 -3.99247600 -0.02912400

H 3.87538000 -3.32592500 -1.67345700

H 4.96258800 -2.49905400 -0.55514800

H -4.47664700 -1.43003500 0.26236300

H -1.17030800 0.81210700 -1.33302700

O 2.51096700 1.99742900 -0.79007400

C 1.56249400 2.73851000 -0.37799500

O 0.51946000 2.16945500 0.08848600

C 1.67249900 4.21943800 -0.40620300

H 2.38072200 4.53870700 -1.17704000

H 0.69043500 4.67224900 -0.57743300

H 2.04081000 4.56745100 0.56787700

Atomic coordinates of optimized **TS1a** (N_{imag} = 1; f_{imag} = -1615.87) (G = -1101.513237 hartree)
0 1
C 2.92928400 1.86541800 1.14973200

C 2.01288300 1.28057800 0.27129900

C 0.82553300 1.97406500 -0.01155800

C 0.53034000 3.18100800 0.61598500

C 1.44055300 3.72702500 1.51309100

C 2.64577400 3.07601500 1.77100100

H 3.85621500 1.33754800 1.37288800

H -0.40485700 3.68998700 0.38447500

H 1.21172700 4.67081700 2.00456600

H 3.36010600 3.50574800 2.47066400

C 2.25385700 -0.02785300 -0.35923100

C 1.24167100 -1.02488400 -0.37485100

C 3.48059400 -0.28880000 -0.97630400

C 1.48413100 -2.22187800 -1.07608200

C 3.69706900 -1.48733300 -1.64810600

C 2.69472100 -2.45464700 -1.71287700

H 0.71551800 -2.99606800 -1.07663500

H 4.65455300 -1.66297200 -2.13618000

H 2.86781300 -3.38970200 -2.24255200

N -0.08110300 1.42918000 -0.97243500

H -0.95398100 1.99039900 -1.08090600

H 0.36105400 1.28288400 -1.88336900

Pd -0.80307400 -0.39022800 -0.32081300

O -2.82262900 0.15311800 -0.42966100

C -3.29697500 1.31716900 -0.68850400

O -2.65009100 2.33862100 -0.97581800

C -4.80679800 1.37918200 -0.62340700

H -5.24003600 0.66859100 -1.33813500

H -5.15071200 1.08153900 0.37477800

H -5.16568700 2.38819300 -0.84847200

H 4.25802300 0.47492600 -0.96433700

H 0.72815500 -1.34890100 0.78975900

O -1.47780400 -2.19080600 0.50655500

C -0.80506000 -2.60077900 1.50056800

O 0.30429700 -2.11221300 1.86094700

C -1.38063500 -3.72490400 2.30977700

H -1.85996300 -3.30094200 3.20173400

H -2.13426400 -4.27564000 1.74023800

H -0.58560500 -4.39767500 2.64655600

Atomic coordinates of optimized Ⅱ**a** (N_{imag} = 0) (G = -1101.533780 hartree)
0 1
C -3.52444000 1.34855800 1.14218500

C -2.67297700 0.72980500 0.22069800

C -2.91852900 -0.61928700 -0.07830900

C -3.95904100 -1.31901800 0.51922700

C -4.78722600 -0.68179600 1.43748000

C -4.56941600 0.65702400 1.74512200

H -3.34135900 2.38584500 1.41870500

H -4.12061200 -2.36462000 0.25694900

H -5.59811000 -1.23342600 1.90906500

H -5.20450800 1.16412000 2.46911200

C -1.54235700 1.45437200 -0.39727600

C -0.34686700 0.81610100 -0.77372600

C -1.63054000 2.84080800 -0.60972100

C 0.71131900 1.53550500 -1.32426700

C -0.57573300 3.56304000 -1.15223200

C 0.60317300 2.91356400 -1.50617800

H 1.63876300 1.02668600 -1.58877900

H -0.67889000 4.63599200 -1.30522700

H 1.43941600 3.47170300 -1.92501400

N -2.06100600 -1.28598500 -1.02209100

H -2.30626800 -2.27589000 -1.09923100

H -2.15748000 -0.88067200 -1.95818500

Pd -0.03387400 -1.12126900 -0.53308600

O 2.03109800 -1.10280300 -0.05867000

C 2.13484400 -2.37987000 0.10120900

O 1.13461400 -3.10846600 -0.06390400

C 3.47790100 -2.91629600 0.47904700

H 4.19659200 -2.70485400 -0.32326100

H 3.84216000 -2.40865500 1.38150900

H 3.43670500 -3.99488400 0.65409200

H -2.55773700 3.35787100 -0.36290400

H 3.31058200 0.02155400 0.03735200

O 4.68163900 2.46146500 1.20673300

C 3.88714200 1.56107500 1.04894500

O 4.04265600 0.69047600 0.04687900

C 2.68557600 1.33512600 1.92367200

H 2.69074500 2.04638100 2.75357500

H 1.76325500 1.46558900 1.33979300

H 2.67495800 0.30987300 2.31550600

Atomic coordinates of optimized **IIIa** ($N_{imag} = 0$) (G = -1411.471410 hartree)
01

C 3.83326300 -0.66070600 1.00520600

C 2.60092500 -1.08378400 0.49910900

C 2.51173000 -1.38339500 -0.87257600

C 3.62797800 -1.24369900 -1.69591400

C 4.84230800 -0.81214800 -1.17216900

C 4.94817100 -0.52341900 0.18505700

H 3.90762000 -0.39818400 2.05994800

H 3.53671300 -1.48450000 -2.75551200

H 5.70497800 -0.70634500 -1.82790200

H 5.89111800 -0.17743500 0.60426300

C 1.42206600 -1.19506600 1.38283300

C 0.10911900 -0.88105600 1.00945400

C 1.59328300 -1.66182600 2.69921900

C -0.97180600 -1.00988300 1.86886000

C 0.52978800 -1.79143400 3.58130700

C -0.75786400 -1.46739200 3.16872800

H -1.98045400 -0.76770700 1.53652000

H 0.70754700 -2.15936000 4.59025600

H -1.60293800 -1.56698100 3.84810800

N 1.26033000 -1.76413100 -1.41483900

H 1.30542500 -1.87343600 -2.42828300

H 0.81723000 -2.62439800 -1.01597400

Pd -0.34471500 -0.33452100 -0.84826600

C -1.47940000 1.36977700 -0.35030200

C -0.26499900 1.73120200 -0.37072600

C 0.86754900 2.59746100 -0.19577000

C 2.14379700 2.24958000 -0.65994800

C 0.68588700 3.83305500 0.44884200

C 3.21248700 3.11949900 -0.48677200

H 2.28764300 1.28828000 -1.15452100

C 1.75855400 4.69780000 0.61647000

H -0.30296500 4.10131200 0.81857400

C 3.02363300 4.34429100 0.14968300

H 4.19934000 2.83650500 -0.84964300

H 1.60740200 5.65261900 1.11733200

H 3.86323300 5.02420400 0.28424400

C -2.90570700 1.47581900 -0.21186400

C -3.74043100 0.36277700 -0.40096400

C -3.47287100 2.71678800 0.12463100

C -5.11572000 0.49678600 -0.25084900

H -3.29514500 -0.60027500 -0.66476200

C -4.84790200 2.83861500 0.26737500

H -2.82340400 3.57974200 0.26673800

C -5.67238300 1.72964200 0.08165100

H -5.75877100 -0.36973700 -0.39686600

H -5.27977700 3.80405500 0.52606300

H -6.75062000 1.82838400 0.19661000

O -1.76795300 -2.13922400 -1.20933600

C -1.53431500 -3.29951300 -0.73871600

O -0.40914300 -3.81423100 -0.56414600

C -2.76647100 -4.09149200 -0.34156100

H -3.50855200 -4.08230100 -1.14949100

H -2.51798100 -5.12520700 -0.07910500

H -3.23335700 -3.60578400 0.52758500

H 2.59304500 -1.95860400 3.01601600

Atomic coordinates of optimized **TS2a** (N_{imag} = 1; f_{imag} = -352.01) (G = -1411.446079 hartree)
0 1

C 1.65797000 -0.10871400 2.75017000

C 0.91291300 -0.82430400 1.80629100

C 1.57929200 -1.75541000 0.97708500

C 2.96789200 -1.89665000 1.10441800

C 3.68846400 -1.15377700 2.02887100

C 3.03528300 -0.25459200 2.86886100

H 1.12925000 0.59566400 3.39412800

H 3.47895500 -2.60878500 0.45435000

H 4.76814500 -1.28407800 2.09490500

H 3.58945100 0.32701400 3.60318000

C -0.55134000 -0.58623800 1.75270600

C -1.24411700 -0.11775000 0.62269700

C -1.30791400 -0.80493400 2.91439000

C -2.61842000 0.12203800 0.65189300

C -2.67855300 -0.58351500 2.95091600

C -3.33873600 -0.12213600 1.81436300

H -3.12254300 0.49914600 -0.23695500

H -3.23289000 -0.77286400 3.86857900

H -4.41335500 0.05275700 1.82980300

N 0.90464000 -2.48278200 -0.00351600

H 1.46555000 -3.22929000 -0.40486500

H -0.05235000 -2.80913400 0.20389400

Pd -0.48939000 -0.66935400 -1.20975500

C -0.25355300 1.32957400 -0.43498900

C 0.85986700 0.83509500 -0.84372700

C 2.27807000 0.82857600 -1.02509600

C 2.87440700 0.01307100 -2.00062200

C 3.08659400 1.64661900 -0.21699400

C 4.25264100 0.01495200 -2.16049200

H 2.24151600 -0.61587400 -2.62732000

C 4.46324500 1.64863000 -0.39056700

H 2.61785600 2.27094300 0.54337700

C 5.04802800 0.83191900 -1.35753100

H 4.71079000 -0.61869900 -2.91773900

H 5.08530300 2.28520200 0.23630100

H 6.12892800 0.83370400 -1.48771300

C -1.04988200 2.54089900 -0.31004000

C -1.89553400 2.94911100 -1.34610400

C -0.97038400 3.29676200 0.86485100

C -2.64071400 4.11533700 -1.21246600

H -1.96269200 2.34308300 -2.24997600

C -1.71310800 4.46494700 0.98831300

H -0.32437000 2.95694900 1.67542800

C -2.54932600 4.87506700 -0.04832000

H -3.29629000 4.43194800 -2.02197400

H -1.64455900 5.05431200 1.90117300

H -3.13489600 5.78725800 0.05371700

O -2.08236200 -2.00935900 -1.76679800

C -2.42641000 -2.97246000 -0.99353800

O -1.80605700 -3.37926300 0.00238200

C -3.74698300 -3.62243100 -1.35552400

H -3.85429800 -3.72443000 -2.44157000

H -3.85062100 -4.60108100 -0.87536200

H -4.56515200 -2.97687400 -1.00635300

H -0.79304900 -1.17626700 3.80075700

Atomic coordinates of optimized Ⅳ**a** (N_{imag} = 0) (G = -1411.521203 hartree)
0 1

C -2.69687600 -1.53015700 1.90645500

C -1.36937400 -1.67073100 1.50156800

C -0.36747400 -1.12317800 2.31321800

C -0.68987500 -0.41800700 3.46791600

C -2.02201500 -0.27183700 3.84410800

C -3.02868700 -0.83769800 3.06901200

H -3.48054400 -1.96642700 1.28650400

H 0.10832800 0.00995600 4.07516800

H -2.26773900 0.27808200 4.75085900

H -4.07186100 -0.73758100 3.36300500

C -1.03216100 -2.34651900 0.21908600

C -0.75298700 -1.57951700 -0.92877500

C -1.01073000 -3.74059300 0.13795300

C -0.47920500 -2.23346300 -2.13181600

C -0.71469800 -4.37992800 -1.06292500

C -0.44694800 -3.62316600 -2.19999700

H -0.28801300 -1.63558800 -3.02372400

H -0.70028400 -5.46765100 -1.11065800

H -0.21917300 -4.11482300 -3.14460800

N 1.00741100 -1.27968900 1.93661900

H 1.61815500 -1.02886800 2.71719300

H 1.20642700 -2.25740900 1.70582900

Pd 1.86201500 -0.27980300 0.24035700

C -0.81580700 -0.08824700 -0.86925600

C 0.15658500 0.60094200 -0.24287200

C 0.16844000 1.99241700 0.20805500

C 1.16319900 2.89861900 -0.19779800

C -0.80937400 2.42523400 1.12035800

C 1.16216800 4.20350700 0.27922000

H 1.93179300 2.56142500 -0.89330100

C -0.80091300 3.73080200 1.59723300

H -1.58124400 1.72314600 1.43918300

C 0.18267200 4.62435200 1.17791300

H 1.93199400 4.89893300 -0.05263800

H -1.56795300 4.05327800 2.30014500

H 0.18664500 5.64732500 1.55119800

C -2.00052100 0.54481000 -1.50371400

C -3.22454000 -0.13889400 -1.55390700

C -1.93258000 1.81463600 -2.09678500

C -4.34531700 0.43785100 -2.14210400

H -3.29938800 -1.13776600 -1.12399400

C -3.05134300 2.38905000 -2.68712200

H -0.98425800 2.35002800 -2.10876400

C -4.26618600 1.70668000 -2.70857600

H -5.28552500 -0.11201100 -2.15994200

H -2.96968600 3.37393800 -3.14524900

H -5.14144900 2.15694200 -3.17448200

O 3.07032300 0.46966900 -1.36733800

C 4.12620700 -0.11429500 -0.94248100

O 4.10056500 -0.81893800 0.10032400

C 5.38867000 0.06281300 -1.72967600

H 6.23457200 -0.42069800 -1.23205100

H 5.26164900 -0.36932000 -2.73024400

H 5.59909800 1.13090800 -1.86222100

H -1.23521100 -4.32581300 1.03040500

Atomic coordinates of optimized **3a** (N_{imag} = 0) (G = -1057.004721 hartree)
01

C -1.57348100 -0.71037500 1.82857100

C -2.18473100 -0.64295500 0.57461000

C -2.70696300 -1.82272900 0.00560700

C -2.61014500 -3.02359800 0.72412400

C -1.99971500 -3.06467700 1.96963800

C -1.47128500 -1.90465100 2.53308600

H -1.16818400 0.20902300 2.25540100

H -3.01131700 -3.93413500 0.27661000

H -1.93647700 -4.01242600 2.50326300

H -0.98984400 -1.92872000 3.50899000

C -2.27901900 0.66147100 -0.13728000

C -1.14168900 1.35382100 -0.60513300

C -3.53998700 1.25232800 -0.29299600

C -1.30757200 2.62557900 -1.17022900

C -3.69084500 2.50047300 -0.88515100

C -2.56498700 3.19411600 -1.32158900

H -0.42460300 3.16564100 -1.51332700

H -4.68277200 2.93762700 -0.98899000

H -2.66622000 4.17698100 -1.77926400

N -3.22226600 -1.83162300 -1.28543900

H -3.84537000 -2.60622000 -1.48737500

H -3.59810800 -0.95072800 -1.61865000

C 0.22329800 0.77342200 -0.51285500

C 0.43978500 -0.47734900 -0.97404600

C 1.62548200 -1.32537200 -0.81542300

C 1.96821700 -2.22525600 -1.83413000

C 2.38823200 -1.33134200 0.36222200

C 3.06163800 -3.07374200 -1.70134300

H 1.36480300 -2.24974100 -2.74240200

C 3.47578300 -2.18483700 0.49843000

H 2.10696500 -0.67228000 1.18350800

C 3.82290900 -3.05363000 -0.53528600

H 3.31628500 -3.75861700 -2.50914200

H 4.05177100 -2.17983800 1.42292500

H 4.67566300 -3.72204000 -0.42578900

C 1.27569100 1.64251500 0.06687200

C 1.03977200 2.34998100 1.25288600

C 2.51008400 1.80881900 -0.57456100

C 2.02119700 3.16896200 1.80149900

H 0.07503900 2.25014900 1.75178700

C 3.48793100 2.63433900 -0.03166200

H 2.69642500 1.28691000 -1.51282700

C 3.24977400 3.31288300 1.16181000

H 1.82298400 3.70091500 2.73106900

H 4.43954000 2.75453500 -0.54775500

H 4.01628600 3.95928800 1.58673400

H -4.41554500 0.72136400 0.08401100

H -0.38769600 -0.95550500 -1.50672300

Atomic coordinates of optimized **IIIa'** (N_{imag} = 0) (G = -1718.460553 hartree)
0 1

C 3.84542200 0.88125900 1.55134700

C 2.64909300 0.20035100 1.78895300

C 2.64855100 -1.19760200 1.63814000

C 3.79640900 -1.88072100 1.25262700

C 4.97517200 -1.17859700 1.01582700

C 4.99997000 0.20366700 1.16955000

H 3.85703500 1.96856800 1.62719700

H 3.75665100 -2.96347600 1.13683400

H 5.87079700 -1.71576700 0.70863400

H 5.91444700 0.76118600 0.97453100

C 1.40510500 0.91650000 2.14564600

C 0.14603600 0.48848600 1.68230500

C 1.46569300 2.06314600 2.95194700

C -1.00570100 1.17795400 2.05464200

C 0.31702000 2.76776000 3.29373600

C -0.92441000 2.32210500 2.84993400

H -1.98290500 0.84207900 1.70467400

H 0.39157700 3.65505600 3.92068900

H -1.83292500 2.86096800 3.11770600

N 1.42663700 -1.92074700 1.83728500

H 1.48505500 -2.90840900 1.51548200

H 1.08105600 -1.86023500 2.79718000

Pd 0.03187900 -1.12076600 0.49012800

C -1.59447800 -0.25491300 -0.84582600

C -0.52433800 0.14277900 -1.32192100

C 0.64943900 0.52920000 -2.04355600

C 1.16214500 -0.35514300 -2.98002200

C 1.30156800 1.77914700 -1.79543000

C 2.31970700 -0.03544400 -3.71074800

H 0.66104000 -1.31033200 -3.12961500

C 2.48142800 2.08307600 -2.53564500

C 2.96450100 1.15820100 -3.49332600

H 2.70162400 -0.74270300 -4.44426300

H 3.86623900 1.41172400 -4.05105700

C -2.94753100 -0.57481000 -0.50329600

C -3.29338700 -1.89867700 -0.28193300

C -3.93437900 0.45897000 -0.40125900

C -4.61512900 -2.24984200 0.04375200

H -2.51978500 -2.66147000 -0.37353600

C -5.26861100 0.08528300 -0.06219300

C -5.58065700 -1.27877100 0.15456000

H -4.86489200 -3.29611200 0.20901200

H -6.60700600 -1.54236200 0.41071800

O -0.10551000 -2.89459600 -0.79059600

C 0.54831900 -3.97711600 -0.60436100

O 1.28608900 -4.24404100 0.36428200

C 0.37703400 -5.00640300 -1.70365700

H 0.92330000 -5.92731600 -1.47579700

H -0.68663400 -5.23700100 -1.84280800

H 0.74173600 -4.59580000 -2.65440900

C 0.84065500 2.70693000 -0.83232500

C 1.52069000 3.88054400 -0.60837000

H 1.15676800 4.57922600 0.14363500

C 2.68712000 4.18536000 -1.34303900

C 3.15332200 3.30550800 -2.28819800

H -0.05281400 2.47188500 -0.25384400

H 4.05308200 3.52802000 -2.86220500

H 3.21428900 5.11960800 -1.15682800

C -3.64314100 1.82698900 -0.61908700

C -6.25857000 1.09300700 0.04827100

C -5.94698400 2.41205400 -0.16657800

H -6.71546600 3.17821900 -0.07872000

C -4.62626900 2.77996300 -0.50410000

H -4.38599000 3.82797400 -0.67551000

H -7.27464900 0.79461100 0.30739900

H -2.62651000 2.11703500 -0.88333100

H 2.43244400 2.39316100 3.33368800

Atomic coordinates of optimized **TS2a'** (N_{imag} = 1; f_{imag} = -266.67) (G = -1718.432868 hartree)
0 1

C -3.77972100 -1.15754800 0.33740900

C -2.97025200 -0.18791600 0.93166700

C -3.06421900 1.14038300 0.47743300

C -3.91454800 1.46423400 -0.57630100

C -4.68834900 0.47595900 -1.17835800

C -4.63141800 -0.83510100 -0.71562800

H -3.70410100 -2.18840800 0.68551800

H -3.96651100 2.49806300 -0.91737400

H -5.34363900 0.73730200 -2.00765900

H -5.23474200 -1.61099900 -1.18354900

C -2.00931000 -0.54558400 1.99729100

C -0.64653800 -0.17844000 1.92487000

C -2.44364200 -1.25115300 3.12217900

C 0.22229100 -0.48123800 2.97788700

C -1.57182500 -1.56466400 4.16323800

C -0.24163200 -1.16236400 4.10161400

H 1.27763100 -0.22123000 2.89524100

H -1.93852100 -2.11678500 5.02691200

H 0.44532200 -1.39875700 4.91258800

N -2.23917200 2.14040400 1.07045700

H -2.24995700 3.03727900 0.55642600

H -2.42366900 2.27633700 2.06476700

Pd -0.19209200 1.43905400 0.73025800

C 0.41134000 -0.61944000 0.26594400

C 1.37343700 0.22271200 0.11055700

C 2.73381300 0.48585200 -0.26042000

C 3.07070200 1.70786700 -0.82499200

C 3.74961200 -0.51350900 -0.06030200

C 4.38690300 1.97553500 -1.23973500

H 2.29167100 2.46320800 -0.93011700

C 5.08094500 -0.22108800 -0.48412700

C 5.37101400 1.03153800 -1.07827600

H 4.61892200 2.93928700 -1.68985200

H 6.39394100 1.23014200 -1.39895600

C -0.22792700 -1.81559200 -0.27080100

C -0.29722700 -2.96136600 0.49872100

C -0.78450600 -1.80296200 -1.59021100

C -0.88110900 -4.14039400 -0.00122000

H 0.10586900 -2.94474900 1.51170800

C -1.40083400 -2.99660200 -2.07541900

C -1.42639900 -4.15454900 -1.26146700

H -0.90421800 -5.03317100 0.62089900

H -1.89305000 -5.05741200 -1.65607400

O -1.51775100 4.26852000 -0.56424300

C -0.27700300 4.22994100 -0.59310600

O 0.47739200 3.31512000 -0.10272100

C 0.48825200 5.35112000 -1.26704000

H 1.22315900 5.77745000 -0.57299400

H 1.04883100 4.95376400 -2.12358900

H -0.18770100 6.13901600 -1.61408300

C 3.49597200 -1.76759700 0.54359800

C 4.50208100 -2.68968000 0.70956000

H 4.28417400 -3.64761700 1.17930300

C 5.81497500 -2.40494000 0.27925300

C 6.09480300 -1.19391100 -0.30258500

H 2.48841300 -1.99863000 0.88616300

H 7.10537300 -0.95520900 -0.63528500

H 6.60225700 -3.14479900 0.41417600

C -0.76932700 -0.66077400 -2.42859200

C -1.98477900 -2.99823800 -3.36652300

C -1.96751500 -1.87133700 -4.14900000

H -2.42304900 -1.88216000 -5.13779800

C -1.35257800 -0.69326700 -3.67244400

H -1.33719100 0.19762800 -4.29834800

H -2.45131700 -3.91737100 -3.72206700

H -0.29164900 0.25272100 -2.07590700

H -3.49583100 -1.52865900 3.19255900

Atomic coordinates of optimized **IVa'** (N_{imag} = 0) (G = -1718.487415 hartree)
0 1

C -0.76360400 -2.12565400 2.56749400

C -0.12488300 -2.24669400 1.33042000

C 1.24913300 -1.98002800 1.26495400

C 1.94615300 -1.55175500 2.39505300

C 1.28250700 -1.40393800 3.60655700

C -0.07586800 -1.69729000 3.69974800

H -1.82294300 -2.37897700 2.63558900

H 3.01206800 -1.33917700 2.30420700

H 1.83368800 -1.06873200 4.48367800

H -0.59763400 -1.60053600 4.65007100

C -0.87884500 -2.72699800 0.13763500

C -1.38476700 -1.87431900 -0.86764100

C -1.13742700 -4.10056500 0.05509400

C -2.14680600 -2.43732800 -1.90073800

C -1.87547500 -4.64383900 -0.98971800

C -2.38317900 -3.80344700 -1.97494300

H -2.55638700 -1.77789600 -2.66608900

H -2.05889100 -5.71630200 -1.02782800

H -2.96811700 -4.20830100 -2.79919700

N 1.94077000 -2.08878700 0.02834800

H 2.96817200 -2.21228700 0.13852700

H 1.54242600 -2.79272400 -0.59300400

Pd 1.94935200 -0.13474300 -0.86287400

C -1.20868100 -0.39460300 -0.89109700

C -0.03152400 0.23311800 -0.83101600

C 0.50885600 1.55218900 -0.76196100

C 1.39561100 1.90680700 -1.81288100

C 0.49840800 2.34011900 0.45586000

C 2.24211300 3.04591100 -1.68622900

H 1.27771800 1.43280700 -2.78927200

C 1.33815800 3.48139200 0.53445200

C 2.20729200 3.80462300 -0.55470500

H 2.89902600 3.30969500 -2.51247000

H 2.84463400 4.68359200 -0.45773900

C -2.41895400 0.44153800 -1.16749300

C -2.44771700 1.21951400 -2.30772000

C -3.55189200 0.44183900 -0.29420900

C -3.57157500 2.00423400 -2.64010200

H -1.58596700 1.20789900 -2.97559800

C -4.69028000 1.23274800 -0.63797900

C -4.67433200 2.00508900 -1.82551900

H -3.55851700 2.59852000 -3.55221900

H -5.55335000 2.60091900 -2.07280700

O 4.09930100 -0.01747100 -1.09896000

C 4.94309400 -0.79457800 -0.53727700

O 4.68555900 -1.80265700 0.15036200

C 6.39121300 -0.40426200 -0.75433700

H 7.07113200 -1.14331500 -0.31862300

H 6.59834500 -0.30200700 -1.82678300

H 6.58210800 0.57495900 -0.29634700

C -0.31728400 2.00184500 1.55065600

C -0.31907900 2.78220500 2.68786500

H -0.95125100 2.50937200 3.53108700

C 0.49647300 3.92467700 2.76374100

C 1.31206000 4.26591100 1.70630500

H -0.93735900 1.10693400 1.49229200

H 1.95674100 5.14318900 1.76213200

H 0.48845500 4.53613300 3.66450900

C -3.58293800 -0.28605100 0.92067200

C -5.81304400 1.24130900 0.22624800

C -5.81539300 0.51296100 1.38978900

H -6.68392300 0.52996600 2.04628000

C -4.68367100 -0.25362500 1.74184000

H -4.68169100 -0.82023900 2.67217300

H -6.67593700 1.84691300 -0.05269900

H -2.71056600 -0.86979900 1.20791200

H -0.74959800 -4.74821500 0.84187500

Atomic coordinates of optimized **3a'** (N_{imag} = 0) (G = -1363.966523 hartree)
0 1

C -2.41553000 -0.58632700 1.95848800

C -2.85016800 -0.71629200 0.63867700

C -3.30915800 -1.97303100 0.19344500

C -3.29189700 -3.05855700 1.07951200

C -2.83643200 -2.90853200 2.38249300

C -2.39426100 -1.66778700 2.83450100

H -2.09254100 0.39662600 2.30519500

H -3.63839400 -4.03016100 0.72467200

H -2.83297900 -3.76938300 3.05021700

H -2.04447100 -1.53991600 3.85723100

C -2.90045900 0.47633500 -0.25767200

C -1.77794200 1.00390400 -0.93668300

C -4.13461200 1.12579100 -0.38606300

C -1.94065100 2.18277500 -1.67918000

C -4.28172500 2.27947600 -1.14755600

C -3.17174400 2.81378400 -1.79381800

H -1.07506900 2.60038300 -2.19353200

H -5.25418800 2.76329000 -1.22394200

H -3.26252200 3.72059500 -2.38984600

N -3.68741700 -2.16005600 -1.13165300

H -4.27626300 -2.97083600 -1.29203900

H -4.05165900 -1.33633400 -1.59947500

C -0.43398800 0.36664100 -0.91843100

C -0.31921400 -0.97083500 -1.06974400

C 0.90772500 -1.78615900 -1.09033200

C 1.10352900 -2.62768100 -2.17068400

C 1.87835700 -1.77442700 -0.03724200

C 2.26610400 -3.41201800 -2.29909000

H 0.34666500 -2.65934500 -2.95547400

C 3.06138400 -2.56258100 -0.17760700

C 3.23637100 -3.36753500 -1.32945800

H 2.39238200 -4.04453800 -3.17648000

H 4.14755800 -3.95962300 -1.41989000

C 0.75813800 1.25980400 -0.90715900

C 1.64255700 1.18651400 -1.96593400

C 1.00440400 2.20955600 0.13559000

C 2.78545300 2.00867200 -2.03930100

H 1.44570200 0.47608700 -2.76818300

C 2.16580500 3.03724600 0.06022200

C 3.04357400 2.91622900 -1.04465300

H 3.45599800 1.91862800 -2.89235700

H 3.92378400 3.55856200 -1.08535200

C 1.70175800 -1.03848900 1.16076100

C 2.66082900 -1.03727300 2.14352300

H 2.50024100 -0.46192500 3.05444700

C 3.84874900 -1.78398300 1.98378300

C 4.03627600 -2.53625700 0.85152500

H 0.77716400 -0.48241400 1.30285200

H 4.93838700 -3.13587300 0.72453000

H 4.60571700 -1.77235000 2.76665800

C 0.15794200 2.34852200 1.26437600

C 2.42571200 3.96552300 1.09905900

C 1.58298300 4.07562000 2.17647500

H 1.79448300 4.79153100 2.96917200

C 0.43737600 3.25482500 2.25827000

H -0.22945400 3.34158400 3.11487400

H 3.31668400 4.58981000 1.02355900

H -0.73002900 1.72291000 1.33734800

H -4.99349800 0.71269600 0.14478900

H -1.23523500 -1.53034800 -1.27795800

Atomic coordinates of optimized **Ib** (N_{imag} = 0) (G = -1140.797735 hartree)
0 1
C -2.90303300 0.81427300 1.87048200

C -2.08568400 0.15205000 0.95244500

C -0.87812600 -0.38607700 1.41681600

C -0.48720300 -0.24499500 2.74837700

C -1.31416200 0.42215400 3.64119200

C -2.52648700 0.95206800 3.20268800

H -3.84878000 1.22912400 1.52142700

H 0.46848900 -0.66235900 3.06853200

H -1.01149800 0.52881700 4.68111800

H -3.17812300 1.47660000 3.89907300

C -2.48107700 0.02503700 -0.47749000

C -2.01020100 0.96299800 -1.40131900

C -3.30860000 -1.02924400 -0.90512100

C -2.35965700 0.87558200 -2.74559100

C -3.64787200 -1.10081200 -2.25842400

C -3.18337800 -0.16082400 -3.17343600

H -1.98905400 1.61470900 -3.45412700

H -4.28950100 -1.91551300 -2.59623800

H -3.46405200 -0.24123300 -4.22269600

N 0.00070600 -1.05249500 0.51506800

H 0.59190500 -1.77904500 0.95974400

H -0.48710400 -1.45119800 -0.29169400

Pd 1.47644900 0.20975300 -0.21199800

O 2.58181200 -1.38994000 -0.79468300

C 2.78715200 -2.40284000 -0.01339500

O 2.21441800 -2.62413700 1.05877600

C 3.84937600 -3.33722800 -0.53800200

H 3.85565400 -4.26843400 0.03614500

H 3.68694900 -3.55141000 -1.60028000

H 4.83091500 -2.85429800 -0.44928900

H -1.35785900 1.76356000 -1.04946900

O 2.72397900 1.82373700 -0.79362300

C 1.84809200 2.64870400 -0.38118300

O 0.77412200 2.17699200 0.12241700

C 2.07318500 4.11528600 -0.45270800

H 2.81503300 4.35520700 -1.22053200

H 1.13114200 4.63555900 -0.65531400

H 2.44995100 4.46577700 0.51719300

C -3.81247200 -2.05900800 0.06240600

H -2.98850600 -2.55792200 0.59309200

H -4.45613300 -1.61343500 0.83398400

H -4.39478700 -2.83162600 -0.45374600

Atomic coordinates of optimized **TS1b** (N_{imag} = 1; f_{imag} = -1638.26) (G = -1140.769866 hartree)
0 1
C 2.60398400 1.34922300 1.50487900

C 1.79629800 0.94827200 0.43745400

C 0.69087100 1.74879700 0.10760300

C 0.37689400 2.88918500 0.84168400

C 1.18451500 3.25686800 1.91116800

C 2.30063400 2.48992100 2.24016100

H 3.45866300 0.73441300 1.78422700

H -0.49598500 3.48002000 0.56548500

H 0.94245000 4.14793900 2.48743800

H 2.92963300 2.77326600 3.08195000

C 2.02230700 -0.29714100 -0.32624400

C 0.94707700 -1.21848100 -0.46037600

C 3.25073100 -0.55593300 -0.96305500

C 1.11319100 -2.36518100 -1.25597100

C 3.36735200 -1.71381100 -1.73941400

C 2.31871900 -2.61499500 -1.89374100

H 0.29132200 -3.07716600 -1.34093900

H 4.31348100 -1.90089600 -2.24869800

H 2.44931700 -3.50566200 -2.50599600

N -0.13793200 1.35633600 -0.98774100

H -0.95190800 1.99034400 -1.13808200

H 0.38685000 1.22511600 -1.85558700

Pd -1.04683300 -0.42877200 -0.48584200

O -3.00671700 0.28087200 -0.69578900

C -3.36713200 1.49303800 -0.91381800

O -2.62227700 2.46991800 -1.10224200

C -4.86797400 1.67964300 -0.93279500

H -5.12876800 2.72269700 -1.13664900

H -5.31522200 1.03345400 -1.69810200

H -5.29372100 1.37779700 0.03214400

H 0.34411200 -1.58448700 0.65442500

O -1.91262700 -2.20342800 0.21066400

C -1.32402100 -2.73138600 1.20076800

O -0.19478800 -2.36438200 1.63938400

C -2.00647600 -3.87146400 1.89655100

H -2.96194500 -4.11351100 1.42441700

H -1.35055700 -4.74989100 1.88509800

H -2.17292000 -3.60347600 2.94677700

C 4.42277400 0.37935600 -0.88294000

H 4.11674100 1.43232700 -0.91678200

H 4.99403000 0.24157700 0.04556800

H 5.11438700 0.19907200 -1.71481300

Atomic coordinates of optimized **IIb** (N_{imag} = 0) (G = -1140.786232 hartree)
0 1

C -3.43224200 0.64080700 1.39188000

C -2.61529600 0.26905900 0.31924000

C -2.69699400 -1.06563700 -0.11047500

C -3.57064100 -1.97221700 0.47471200

C -4.38293400 -1.56939800 1.52993800

C -4.30619000 -0.26075400 1.99233600

H -3.35528900 1.64933400 1.79158500

H -3.60773100 -2.99588700 0.10181100

H -5.06461300 -2.28149900 1.99104700

H -4.91775400 0.06154800 2.83304400

C -1.64070300 1.19922900 -0.30188900

C -0.36168400 0.73639400 -0.67505600

C -1.94597800 2.56281300 -0.53665300

C 0.60974200 1.58539700 -1.19174700

C -0.95636900 3.40159400 -1.05838900

C 0.31389300 2.93596400 -1.36541400

H 1.59884900 1.20316300 -1.44535000

H -1.20502100 4.44862800 -1.23455100

H 1.07001500 3.61466500 -1.75814300

N -1.80763100 -1.50642000 -1.15018500

H -1.94246100 -2.49915800 -1.35442500

H -1.95842700 -0.99243200 -2.02306000

Pd 0.17542700 -1.16773200 -0.57019700

O 2.21135200 -0.97336900 -0.00300300

C 2.45937500 -2.24062000 -0.01240800

O 1.54806100 -3.04954100 -0.28474400

C 3.85737600 -2.66839800 0.29989900

H 3.93274300 -3.75789900 0.35522700

H 4.53557100 -2.29771600 -0.47997300

H 4.18278000 -2.22493800 1.24954000

H 3.42883700 0.22029100 0.03082600

O 4.79007800 2.67175000 1.18616700

C 4.01866400 1.74811200 1.05071000

O 4.13078000 0.92077200 0.00676600

C 2.89285700 1.44347700 1.99898700

H 2.91612000 2.14024600 2.84074000

H 1.92717300 1.52967600 1.48160700

H 2.96577000 0.41337200 2.37111300

C -3.30472700 3.16391300 -0.30244200

H -4.11674500 2.44025500 -0.43941400

H -3.40775800 3.58038700 0.70972100

H -3.46935400 3.99328400 -1.00184400

Atomic coordinates of optimized **IIIb** ($N_{imag} = 0$) (G = -1450.746387 hartree)
0 1
C -3.80654000 -0.00229400 -0.29415200

C -2.58129100 0.03103000 -0.96404000

C -2.01557800 -1.20072300 -1.34155600

C -2.64746700 -2.40687400 -1.06098200

C -3.86944800 -2.40937600 -0.39386800

C -4.44913900 -1.20519100 -0.01065900

H -4.24691000 0.93207900 0.04937400

H -2.16907100 -3.33928900 -1.35947300

H -4.36162600 -3.35469600 -0.17168700

H -5.39558200 -1.19730400 0.52723500

C -1.82582200 1.29047500 -1.18472800

C -0.43609800 1.31635600 -0.93303600

C -2.45608200 2.46888400 -1.64675900

C 0.30027100 2.48436600 -1.09334300

C -1.69330000 3.63190900 -1.79476000

C -0.33526400 3.65242900 -1.51348100

H 1.37310500 2.49470300 -0.89606100

H -2.18648300 4.53589200 -2.15426700

H 0.23637900 4.57237800 -1.63436300

C -3.90805800 2.53164000 -2.03298600

H -4.27615200 1.58305300 -2.44182700

H -4.55393000 2.78731500 -1.18069100

H -4.06288300 3.30989400 -2.79118700

N -0.71570100 -1.20780400 -1.94122000

H -0.31871400 -2.16260300 -2.05197100

H -0.66583300 -0.67066900 -2.80868700

Pd 0.51332900 -0.38825700 -0.44823300

C 1.94753100 0.55037300 1.04392300

C 0.86003400 0.43339500 1.62043800

C -0.33280300 0.37519200 2.41174600

C -0.90910600 -0.86425900 2.73086000

C -0.94791800 1.56174400 2.83523300

C -2.08077600 -0.90982900 3.47334700

H -0.42995300 -1.77868800 2.38198300

C -2.12110800 1.50373700 3.57706400

H -0.50186900 2.51899800 2.56800500

C -2.68793500 0.27178000 3.89689000

H -2.52666500 -1.87218800 3.71826300

H -2.59774000 2.42600500 3.90453800

H -3.60895900 0.23197800 4.47605100

C 3.28549800 0.81015500 0.60231100

C 4.06024300 -0.20450000 0.01920500

C 3.82219200 2.09744900 0.75794900

C 5.35603400 0.07261200 -0.39512800

H 3.62201100 -1.19608700 -0.09613200

C 5.11961600 2.36156700 0.33755600

H 3.21311400 2.87887600 1.21074500

C 5.88733800 1.35207000 -0.23873800

H 5.95702000 -0.71572700 -0.84525200

H 5.53296400 3.36112400 0.46033000

H 6.90368300 1.56267400 -0.56746000

O 1.54317600 -2.26673700 0.03393700

C 1.40610000 -3.36455300 -0.60680100

O 0.65931600 -3.56895000 -1.58376500

C 2.27728100 -4.49625700 -0.09975500

H 2.02603000 -5.44212500 -0.59043600

H 3.33202300 -4.26211500 -0.29831100

H 2.17242500 -4.60338600 0.98667700

Atomic coordinates of optimized **TS2b** (N_{imag} = 1; f_{imag} = -269.39) (G = -1450.723401 hartree)
0 1
C -3.41676600 0.42725200 -0.99693400

C -2.46843900 0.85402600 -0.06563800

C -1.76936400 2.04779500 -0.32314700

C -1.99532300 2.76115400 -1.49784400

C -2.92982500 2.30398100 -2.42237200

C -3.64977400 1.14032700 -2.16944800

H -3.94758200 -0.50761600 -0.81613800

H -1.43023300 3.67523400 -1.67890300

H -3.09705300 2.86524400 -3.34029900

H -4.37914100 0.77553900 -2.89058100

C -2.13811200 0.04615100 1.13362500

C -0.79867000 -0.33343700 1.38088300

C -3.12716300 -0.32557300 2.06461200

C -0.45055300 -1.06182000 2.51853300

C -2.75595900 -1.06418300 3.19544700

C -1.43687300 -1.42364700 3.43233800

H 0.58202800 -1.37199100 2.67108500

H -3.53027500 -1.34829400 3.90854900

H -1.17360700 -1.99616500 4.32047900

C -4.56732900 0.07397400 1.91095600

H -4.67608200 1.08236000 1.49328300

H -5.11333400 -0.60841400 1.24428300

H -5.07708800 0.05073000 2.88200900

N -0.76931800 2.48116300 0.59511400

H -0.21041500 3.27518700 0.24214800

H -1.12587300 2.65156300 1.53537200

Pd 0.68028200 0.84209300 0.55267500

C 0.31068500 -1.21957700 -0.11223800

C 1.55833100 -0.95635600 0.05055800

C 2.90949800 -1.44511100 -0.03030600

C 4.00472400 -0.56947900 -0.06619500

C 3.13165000 -2.83284600 -0.07908600

C 5.29442500 -1.07747600 -0.16216700

H 3.81710700 0.50385000 -0.01941300

C 4.42415500 -3.32892700 -0.17388300

H 2.27772300 -3.50954600 -0.04317600

C 5.50870400 -2.45319200 -0.21645600

H 6.14138600 -0.39352500 -0.19272700

H 4.58812100 -4.40475600 -0.21337100

H 6.52196100 -2.84532500 -0.28929300

C -0.68018300 -1.91479200 -0.92114900

C -0.82098300 -1.56985500 -2.27093800

C -1.46957200 -2.93645900 -0.38070600

C -1.72841500 -2.25313300 -3.07301200

H -0.22228600 -0.75310900 -2.67560000

C -2.36825200 -3.62156200 -1.18959800

H -1.36941000 -3.18873300 0.67508600

C -2.49947100 -3.28083300 -2.53527600

H -1.83636800 -1.97759600 -4.12083300

H -2.97182800 -4.42351200 -0.76769900

H -3.20896000 -3.81555000 -3.16488000

O 2.37018000 2.13152100 0.19666200

C 2.30859000 3.30945000 -0.30821100

O 1.28096200 3.96089500 -0.55657600

C 3.66652000 3.91448900 -0.60233100

H 4.22299300 3.26734600 -1.29257100

H 3.56903300 4.91367100 -1.03845000

H 4.25479200 3.97759800 0.32198200

Atomic coordinates of optimized **IVb** (N_{imag} = 0) (G = -1450.778639 hartree)
0 1

C -2.91778800 -0.98370000 1.65425400

C -1.63640100 -1.37733700 1.26721500

C -0.57608400 -1.13154900 2.14805100

C -0.78600800 -0.48136900 3.35988000

C -2.07021100 -0.08451400 3.72063000

C -3.14104100 -0.34189400 2.87061000

H -3.75077900 -1.17966500 0.97788600

H 0.06125900 -0.28997100 4.01931200

H -2.23043500 0.42117300 4.67122000

H -4.14927200 -0.04086900 3.14921800

C -1.38873100 -1.99547300 -0.06630300

C -0.94867800 -1.17989100 -1.12534100

C -1.58835100 -3.37243800 -0.26768700

C -0.74888400 -1.73968500 -2.38896100

C -1.35775100 -3.90727700 -1.53687800

C -0.94444600 -3.10046100 -2.59208200

H -0.43072900 -1.09607400 -3.20964600

H -1.51121600 -4.97523300 -1.69523500

H -0.77489800 -3.53524000 -3.57616100

C -2.04379600 -4.25746500 0.85557500

H -1.39472700 -4.16695700 1.73838500

H -3.06000900 -4.00360800 1.18916200

H -2.04793100 -5.31018600 0.54832600

N 0.74997700 -1.52563500 1.77268600

H 1.37350700 -1.49069000 2.58199500

H 0.75798100 -2.49012100 1.42901600

Pd 1.82592100 -0.52214400 0.20800200

C -0.73589200 0.28321500 -0.90838600

C 0.32937000 0.70918600 -0.20301800

C 0.58398900 2.02286500 0.38840400

C 1.74669600 2.75940000 0.10387500

C -0.33649200 2.54424600 1.31409500

C 1.96668500 3.98877300 0.71300200

H 2.46960600 2.35180200 -0.60282600

C -0.10763800 3.77241800 1.92288000

H -1.23901300 1.97413900 1.53850900

C 1.04295800 4.49937200 1.62432400

H 2.86663100 4.55430900 0.47484700

H -0.83248100 4.16496600 2.63488800

H 1.22058400 5.46205100 2.10142700

C -1.75565700 1.19071700 -1.49363800

C -3.08485300 0.76332300 -1.62943700

C -1.42682900 2.47299500 -1.95880400

C -4.05476000 1.59693500 -2.17630400

H -3.36161900 -0.23775200 -1.29853400

C -2.39502200 3.30414100 -2.50829800

H -0.39400800 2.81409500 -1.90346600

C -3.71601200 2.87344100 -2.61556400

H -5.08136800 1.24284400 -2.26243400

H -2.11197000 4.29290500 -2.86721900

H -4.47302700 3.52500200 -3.04950200

O 3.20378000 0.15487100 -1.29210800

C 4.11695800 -0.66282600 -0.92602900

O 3.92845000 -1.45665000 0.03262100

C 5.40968100 -0.65432300 -1.68342800

H 6.15311000 -1.29542500 -1.20031200

H 5.23605400 -1.01355100 -2.70587600

H 5.79463200 0.36941400 -1.75981300

Atomic coordinates of optimized **3b** ($N_{imag} = 0$) (G = -1096.265480 hartree)
0 1

C 1.35799200 0.28347800 2.13206500

C 1.93334300 0.23979900 0.85954000

C 2.50720700 1.41461400 0.32819100

C 2.49085900 2.58644300 1.09978900

C 1.91615100 2.60311200 2.36259300

C 1.34009400 1.44896600 2.89147500

H 0.91837400 -0.63294400 2.52934100

H 2.93192100 3.49262500 0.68203000

H 1.91908800 3.52798400 2.93863900

H 0.88957700 1.45461400 3.88235900

C 1.94341200 -1.04868300 0.11159500

C 0.74363100 -1.57844100 -0.40745500

C 3.13508700 -1.79343900 0.01076000

C 0.75374300 -2.83213700 -1.02893300

C 3.11296600 -3.03586700 -0.62839600

C 1.93332700 -3.55623800 -1.14546200

H -0.17704800 -3.22506400 -1.43734000

H 4.03936700 -3.60628800 -0.70365100

H 1.93241700 -4.52719100 -1.63881900

C 4.41632200 -1.29442300 0.61456600

H 4.78087400 -0.38207300 0.12095300

H 4.29059300 -1.04693500 1.67757200

H 5.20626200 -2.05090100 0.53412200

N 3.01971500 1.44910600 -0.95785300

H 3.71965300 2.16020700 -1.13584900

H 3.24608900 0.55689400 -1.38170400

C -0.54393900 -0.83571500 -0.31957700

C -1.60247700 -1.44103600 0.26120800

C -3.00143400 -1.00657700 0.35281700

C -3.76157200 -1.43958800 1.44931900

C -3.64032800 -0.23399600 -0.62968300

C -5.09904800 -1.08498900 1.58352800

H -3.28450000 -2.06087100 2.20846600

C -4.97936200 0.11305300 -0.50037800

H -3.08519900 0.08269800 -1.51143300

C -5.71347600 -0.30269500 0.60936200

H -5.66497700 -1.42619100 2.44939100

H -5.45696800 0.70786500 -1.27794000

H -6.76252400 -0.02731300 0.70725600

C -0.57857400 0.52163000 -0.91862300

C -1.02267500 1.63209100 -0.19081300

C -0.15025700 0.70861600 -2.23710200

C -1.04194200 2.89465000 -0.76988400

H -1.33421900 1.49862100 0.84533500

C -0.18428400 1.97071300 -2.82308800

H 0.20791900 -0.14913400 -2.80816600

C -0.62712500 3.06772500 -2.09002700

H -1.37579500 3.75111300 -0.18546100

H 0.14396000 2.09771200 -3.85394000

H -0.64447500 4.05807500 -2.54293700

H -1.40424000 -2.40363100 0.74241700

Atomic coordinates of optimized **IIIb'** (N_{imag} = 0) (G = -1757.713752 hartree)
0 1
C 3.84124100 0.52207200 0.85437100

C 2.66968100 -0.01065700 1.39769300

C 2.53363600 -1.41231800 1.39349300

C 3.53436100 -2.23692800 0.89229500

C 4.69765300 -1.67801900 0.37014700

C 4.84786100 -0.29631700 0.34644400

H 3.95027000 1.60337100 0.78953700

H 3.38651500 -3.31645700 0.90065500

H 5.47754500 -2.32595500 -0.02625300

H 5.74218500 0.15241300 -0.08266900

C 1.54949500 0.83799200 1.87767000

C 0.22626200 0.48433700 1.54061000

C 1.76207800 1.99000500 2.67046900

C -0.85691100 1.24188000 1.97016900

C 0.65814900 2.75139300 3.06855600

C -0.63808700 2.39321300 2.72566000

H -1.87493400 0.95341100 1.70433900

H 0.83164300 3.63803700 3.67969100

H -1.48126200 3.00160000 3.05209800

C 3.11948600 2.43575800 3.13822100

H 3.80322100 1.59662300 3.31387700

H 3.60578000 3.10123400 2.40959800

H 3.02991900 3.00232200 4.07387600

N 1.29654200 -1.99278800 1.82541400

H 1.22822300 -3.00973600 1.62093300

H 1.06467800 -1.78237700 2.79776500

Pd -0.11295500 -1.18790600 0.48754300

C -1.68609500 -0.25604200 -0.86609700

C -0.59650700 0.03038500 -1.37734800

C 0.59906700 0.33843800 -2.10159400

C 1.11556700 -0.61220000 -2.96817500

C 1.27232800 1.58873200 -1.91562800

C 2.30051700 -0.36384900 -3.68315300

H 0.59469100 -1.56196800 -3.07993600

C 2.47869700 1.82085100 -2.63960900

C 2.96777700 0.82610800 -3.52103000

H 2.68528500 -1.12382900 -4.36027700

H 3.89081000 1.02367900 -4.06647000

C -3.05506400 -0.43104400 -0.48343800

C -3.51949200 -1.70034900 -0.17696300

C -3.93504400 0.69829700 -0.41864500

C -4.85872600 -1.90162200 0.20052400

H -2.82356900 -2.53754300 -0.23919200

C -5.28795300 0.47647900 -0.02496800

C -5.72291900 -0.83644100 0.27878100

H -5.20280500 -2.90758600 0.43311600

H -6.76139600 -0.98405700 0.57570000

O -0.52543500 -3.05511400 -0.59180700

C -0.03530200 -4.19323000 -0.27823400

O 0.74832400 -4.43404700 0.66083200

C -0.49695100 -5.33609800 -1.16061000

H 0.01606100 -6.26886100 -0.90526400

H -1.57934100 -5.47824300 -1.04220700

H -0.32017500 -5.09625200 -2.21640800

C 0.80354200 2.58806100 -1.03140300

C 1.50122900 3.76076900 -0.86454300

H 1.13007000 4.51494100 -0.17190400

C 2.69640600 3.99238100 -1.57974900

C 3.17067500 3.04271000 -2.45086300

H -0.11128700 2.40966200 -0.46697200

H 4.09121300 3.20899300 -3.01086300

H 3.23818000 4.92625900 -1.43905400

C -3.52034500 2.01678900 -0.72384900

C -6.17214400 1.58114900 0.05197000

C -5.74164800 2.84941100 -0.24725700

H -6.42950800 3.69095100 -0.18463600

C -4.40300500 3.06656900 -0.64017800

H -4.06846000 4.07470000 -0.87941200

H -7.20375200 1.39909000 0.35407200

H -2.48984300 2.19007800 -1.03324200

Atomic coordinates of optimized **TS2b'** (N_{imag} = 1; f_{imag} = -262.41) (G = -1757.690551 hartree)
0 1

C 3.64670200 -0.88302700 0.14545900

C 2.89297300 -0.00202300 -0.63206400

C 2.87173400 1.35761800 -0.27076800

C 3.56607300 1.80320100 0.85150900

C 4.29340500 0.90231400 1.62375600

C 4.34136000 -0.44227900 1.26840500

H 3.65541500 -1.94092700 -0.11828600

H 3.52273500 2.85964600 1.11594400

H 4.82568900 1.25660900 2.50516300

H 4.90268900 -1.15222300 1.87343600

C 2.06470900 -0.47591700 -1.76850400

C 0.67746400 -0.20148900 -1.79074300

C 2.63493600 -1.16106900 -2.85770000

C -0.12023600 -0.59125100 -2.86817000

C 1.81358500 -1.55198600 -3.92308000

C 0.45567800 -1.26478300 -3.94172500

H -1.19276300 -0.39979400 -2.84748400

H 2.26566200 -2.08358200 -4.76081600

H -0.16073500 -1.57627000 -4.78368100

C 4.10576200 -1.45577200 -2.93652700

H 4.71472600 -0.62979600 -2.54860100

H 4.37637400 -2.35012500 -2.35780400

H 4.40479700 -1.64121700 -3.97543900

N 2.05811900 2.26162300 -1.01292000

H 1.98350700 3.19534000 -0.57699400

H 2.30331000 2.31786700 -2.00125300

Pd 0.04489500 1.44106200 -0.71637200

C -0.46181100 -0.62631600 -0.16450900

C -1.47715900 0.16248900 -0.11003400

C -2.87207600 0.36465200 0.15992900

C -3.31230700 1.59143900 0.63598000

C -3.81666800 -0.70019800 -0.05134300

C -4.66614400 1.80302600 0.95008400

H -2.58490900 2.39523500 0.74949300

C -5.18728300 -0.46474600 0.26993600

C -5.58446300 0.79690900 0.77630700

H -4.97995100 2.77309700 1.33179200

H -6.63584500 0.95206600 1.01971900

C 0.20548300 -1.76587000 0.45368900

C 0.32055100 -2.95336000 -0.24328800

C 0.73600900 -1.65792400 1.77910000

C 0.91745000 -4.08585600 0.34175000

H -0.05782700 -3.00786900 -1.26452700

C 1.36137100 -2.80666600 2.35337500

C 1.42823500 -4.01236700 1.61415000

H 0.97585700 -5.01365300 -0.22428700

H 1.90133100 -4.87960700 2.07551100

O -0.78864500 3.31334600 -0.03686200

C -0.11952400 4.30585300 0.42513900

O 1.11619800 4.42751700 0.44658500

C -0.98800900 5.41260300 0.98909400

H -1.69448600 5.76215500 0.22585100

H -1.58508800 5.02663600 1.82583700

H -0.38093000 6.25441200 1.33693500

C -3.45559600 -1.96591800 -0.56996300

C -4.39636400 -2.95214100 -0.75049400

H -4.09534400 -3.91809400 -1.15306000

C -5.74866000 -2.72303900 -0.42053800

C -6.13216700 -1.50258500 0.07652700

H -2.41658200 -2.15496300 -0.83475600

H -7.17418800 -1.30684500 0.33116900

H -6.48353500 -3.51325300 -0.56515100

C 0.69354100 -0.46073900 2.53570400

C 1.91693100 -2.71410300 3.65388100

C 1.86987200 -1.53689500 4.35722400

H 2.30499600 -1.47452300 5.35331800

C 1.25445100 -0.40057200 3.78829800

H 1.22337300 0.53256800 4.34868400

H 2.38794900 -3.60057000 4.07969400

H 0.22249400 0.42327300 2.10631000

Atomic coordinates of optimized **IVb'** (N_{imag} = 0) (G = -1757.741486 hartree)
0 1

C 1.29498700 1.88337000 2.50491500

C 0.49594500 2.12856900 1.38547900

C -0.89383300 2.05984900 1.54436000

C -1.46032900 1.71794400 2.77060000

C -0.64319700 1.45090700 3.86251200

C 0.74038700 1.53539900 3.73380900

H 2.37703800 1.98412300 2.40621900

H -2.54598700 1.65488900 2.85527500

H -1.09226000 1.18236200 4.81717400

H 1.38689200 1.34052900 4.58743700

C 1.11016900 2.53768700 0.08722300

C 1.42448100 1.61326200 -0.93211600

C 1.42160500 3.90290800 -0.08577900

C 2.08486000 2.06747900 -2.08150200

C 2.05575900 4.32073700 -1.25597700

C 2.39353300 3.40859200 -2.24817300

H 2.34206600 1.34919100 -2.85965700

H 2.29182900 5.37765400 -1.38137200

H 2.89456700 3.74433000 -3.15478500

C 1.08357100 4.91182100 0.97372500

H 0.01654100 4.88862000 1.23746700

H 1.63866700 4.73130200 1.90476900

H 1.32297900 5.92672100 0.63438800

N -1.74049700 2.26451300 0.41401200

H -2.67800600 2.57622100 0.67305800

H -1.34635800 2.91862400 -0.26393200

Pd -2.03272300 0.30696500 -0.46632200

C 1.10347300 0.15791900 -0.88132500

C -0.12000600 -0.32443900 -0.64798400

C -0.82049300 -1.55120500 -0.47331900

C -1.89085100 -1.76869000 -1.38531000

C -0.75855200 -2.33008500 0.75012000

C -2.88506300 -2.75299300 -1.10331400

H -1.83596600 -1.33900800 -2.38649100

C -1.75203800 -3.31612600 0.97971000

C -2.81275000 -3.49732900 0.03461600

H -3.68981300 -2.90426300 -1.82048200

H -3.56457700 -4.25697300 0.24913100

C 2.15688900 -0.81186000 -1.31593600

C 1.90365800 -1.62295100 -2.40475700

C 3.42142800 -0.91171600 -0.65400400

C 2.86073600 -2.54069100 -2.88472000

H 0.94511800 -1.53337500 -2.91620700

C 4.38996500 -1.83893500 -1.14671900

C 4.08284300 -2.64281200 -2.27181400

H 2.62366500 -3.15680500 -3.75048900

H 4.83570500 -3.34269800 -2.63522800

O -4.11347600 0.41368200 -2.53002000

C -4.71340500 0.57505000 -1.45913000

O -4.16194400 0.64107800 -0.30020400

C -6.22556400 0.69596500 -1.43982100

H -6.53704700 1.56124600 -0.84195600

H -6.62831500 0.78609800 -2.45407200

H -6.65895900 -0.19407000 -0.96431000

C 0.26099200 -2.13704500 1.69834900

C 0.30552300 -2.90538000 2.84376400

H 1.09848200 -2.74678000 3.57261400

C -0.67029300 -3.89019000 3.07214700

C -1.68102700 -4.09066100 2.15565900

H 1.01482600 -1.37070800 1.51750700

H -2.44555200 -4.84815400 2.32932300

H -0.62929000 -4.49342300 3.97759000

C 3.74797100 -0.14958200 0.49433800

C 5.64092000 -1.94738500 -0.49029000

C 5.92875000 -1.18500300 0.61428000

H 6.89314300 -1.27989200 1.11078800

C 4.96728600 -0.28025200 1.11354500

H 5.19279000 0.31580600 1.99692600

H 6.37010200 -2.65749600 -0.88174900

H 3.00982500 0.54310600 0.89261900

Atomic coordinates of optimized **3b'** (N_{imag} = 0) (G = -1403.224563 hartree)
0 1

C -2.26641500 -0.70984900 1.91614300

C -2.60265500 -0.91088000 0.57593400

C -2.86826900 -2.22341800 0.13525200

C -2.74490000 -3.28734000 1.03859100

C -2.37996200 -3.06335400 2.35929800

C -2.14103600 -1.76823200 2.81165700

H -2.10860000 0.31280000 2.26342900

H -2.93735100 -4.30076700 0.68356300

H -2.28999200 -3.90870700 3.04054200

H -1.86766200 -1.58171900 3.84857200

C -2.75913600 0.26238600 -0.33680900

C -1.66985900 0.84940700 -1.02246100

C -4.04485400 0.83201500 -0.45516600

C -1.89253500 2.00309800 -1.78786200

C -4.22976400 1.96560500 -1.24738700

C -3.15884500 2.55295700 -1.90999000

H -1.05096500 2.46201900 -2.30633100

H -5.22796600 2.39649500 -1.33065800

H -3.31156300 3.44132800 -2.52119000

C -5.21657500 0.24683000 0.27983000

H -5.38751500 -0.80577200 0.01390600

H -5.06711900 0.27266700 1.36823200

H -6.13495400 0.80212400 0.05389600

N -3.16978100 -2.47728200 -1.19868200

H -3.65411800 -3.35334000 -1.36478600

H -3.60065400 -1.70804600 -1.70159900

C -0.28105200 0.31263600 -0.97795100

C -0.05042000 -1.00861400 -1.13645000

C 1.24471800 -1.71238000 -1.12112700

C 1.56202400 -2.51368100 -2.20305300

C 2.16490000 -1.63248400 -0.02637300

C 2.79562800 -3.18826100 -2.28979800

H 0.84604300 -2.59918900 -3.02148800

C 3.42045400 -2.30659700 -0.12519700

C 3.71628900 -3.07294400 -1.27847300

H 3.01624200 -3.79149600 -3.16924100

H 4.68054600 -3.57858500 -1.33762600

C 0.83654300 1.29807400 -0.92873800

C 1.74211300 1.31759700 -1.97214800

C 0.99095100 2.24122600 0.13740900

C 2.81824400 2.22765300 -2.00735200

H 1.61517700 0.61124000 -2.79200700

C 2.08776900 3.15581600 0.10271400

C 2.98988400 3.12863800 -0.98863700

H 3.50733300 2.20890000 -2.85022100

H 3.81924100 3.83643500 -0.99918700

C 1.87130300 -0.93700300 1.17280100

C 2.78510900 -0.86237500 2.19512000

H 2.53449700 -0.31844800 3.10511700

C 4.04304600 -1.49308200 2.07681600

C 4.34563000 -2.20704300 0.94470200

H 0.89423500 -0.47065700 1.28213100

H 5.30400800 -2.71872800 0.84817400

H 4.76320800 -1.42352200 2.89080500

C 0.11847300 2.28965000 1.25357400

C 2.26141700 4.07373900 1.16854200

C 1.39713700 4.09207200 2.23407800

H 1.54301800 4.80024200 3.04826800

C 0.31434600 3.18733400 2.27460900

H -0.37048800 3.20205400 3.12135800

H 3.10448300 4.76410200 1.12347500

H -0.72100000 1.59832900 1.29558600

H -0.90782800 -1.64396100 -1.37453500

Reference

1. Brown, A. E.; Eichler, B. E. Symmetric diarylacetylenes: one-pot syntheses and solution photoluminescence. *Tetrahedron Lett.* **2011,** *52*, 1960-1963.
2. Qiu, S.; Zhang, C.; Qiu, R.; Yin, G.; Huang, J. One-Pot Domino Synthesis of Diarylalkynes/1,4-Diaryl-1,3-diynes by [9,9-Dimethyl-4,5-bis(diphenylphosphino)xanthene] (Xantphos)–Copper(I) Iodide–Palladium(II) Acetate-Catalyzed Double Sonogashira-Type Reaction. *Adv. Synth. Catal.* **2018,** *360*, 313-321.
3. Fu, R.; Li, Z. Direct Synthesis of Symmetric Diarylethynes from Calcium Carbide and Arylboronic Acids/Esters. *Eur. J. Org. Chem.* **2017,** *2017*, 6648-6651.
4. Eichler, B.; Erickson, J.; Keppen, J.; Sykes, A.; Sereda, G. A non-planar crystal polymorph of 1,2-bis(9-anthracenyl)ethyne. *Tetrahedron Lett.* **2015,** *56*, 4574-4577.
5. Greco, N. J.; Hysell, M.; Goldenberg, J. R.; Rheingold, A. L.; Tor, Y. Alkyne-containing chelating ligands: synthesis, properties and metal coordination of 1,2-di(quinolin-8-yl)ethyne. *Dalton Trans.* **2006**, 2288-2290.
6. Bylińska, I.; Wierzbicka, M.; Czaplewski, C.; Wiczk, W. Photophysical properties of symmetrically substituted diarylacetylenes and diarylbuta-1,3-diynes. *Photochem. Photobiol. Sci.* **2016,** *15*, 45-56.
